# Canakinumab or emavusertib for anemia in lower-risk MDS: results of the CANFIRE and LUCAS phase 2, open-label, multicenter trials

**DOI:** 10.1038/s43856-026-01885-z

**Published:** 2026-09-16

**Authors:** Anne Sophie Platzbecker, Katharina Zoldan, Dominic Brauer, Philipp Kiewe, Daniel Sasca, Katja Sockel, Thomas Illmer, Aristoteles Giagounidis, Daniel Nowak, Kathrin Rieger, Friederike Wortmann, Katharina Götze, Martin Schmidt-Hieber, Karin Mayer, Ursula Vehling-Kaiser, Rudolf Weide, Stefan Mahlmann, Jens Przybilla, Susanne Melzer, Michael Cross, Marie Schneider, Dirk Hasenclever, Klaus H. Metzeler, Uwe Platzbecker

**Affiliations:** 1 Else Kroener Fresenius Center for Digital Health, Faculty of Medicine and University Hospital Carl Gustav Carus, TUD Dresden University of Technology, Dresden, Germany; 2 Medizinische Universität Lausitz – Carl Thiem, Cottbus, Germany; 3 Digital Health Cluster, Department for Digital Health, Economics & Policy, Hasso-Plattner Institute, University of Potsdam, Potsdam, Germany; 4 Department of Hematology, Cellular Therapy, Hemostaseology and Infectious Diseases, University Medical Center, Leipzig, Leipzig, Germany; 5 Medizinisches Versorgungszentrum “Onkologischer Schwerpunkt am Oskar-Helene-Heim”, Berlin, Germany; 6 Department of Hematology and Medical Oncology, University Medical Center of the Johannes Gutenberg University Mainz, Mainz, Germany; 7 Department of Internal Medicine I, University Hospital Dresden, TU Dresden, Dresden, Germany; 8 Outpatient Center, Haematology and Oncology, Dresden, Germany; 9 Department of Oncology, Hematology and Palliative Care, Marien-Hospital, Duesseldorf, Germany; 10 Department of Hematology and Oncology, Medical Faculty Mannheim, Heidelberg University, Mannheim, Germany; 11 Department of Hematology, Oncology and Cancer Immunity, Campus Benjamin Franklin, Charité - Universitätsmedizin Berlin, Berlin, Germany; 12 Department for Hematology and Oncology, Universitätsklinikum Schleswig-Holstein and University Cancer Center Schleswig-Holstein, Lübeck, Germany; 13 Department of Internal Medicine III, Klinikum rechts der Isar, School of Medicine and Health, Technical University of Munich, München, Germany; 14 Clinic of Haematology, Oncology, Pneumology, Nephrology and Diabetology, Medical University Lausitz - Carl Thiem (MUL-CT), Cottbus, Germany; 15 Clinic of Internal Medicine III, Oncology, Hematology, Immune-Oncology and Rheumatology, University Hospital Bonn, Bonn, Germany; 16 Outpatient Clinic, Landshut, Germany; 17 Institut für Versorgungsforschung in der Onkologie, Koblenz, Germany; 18 Hematology/Oncology and Nephrology Clinic, Friedrich-Ebert-Hospital Neumünster, Neumünster, Germany; 19 Clinical Trial Centre (ZKS), Leipzig, Germany; 20 Institute for Medical Informatics, Statistics & Epidemiology (IMISE), Leipzig, Germany; 21 Executive Board Office, University Hospital Carl Gustav Carus, TU Dresden, Dresden, Germany

**Keywords:** Myelodysplastic syndrome, Myelodysplastic syndrome

## Abstract

**Background:**

Dysregulated innate immune signaling promotes inflammatory suppression of hematopoietic stem and progenitor cell function, contributing to ineffective erythropoiesis in myelodysplastic neoplasms (MDS). The phase 2, open-label CANFIRE and LUCAS trials aimed to determine the efficacy and safety of canakinumab or emavusertib, respectively, in patients with lower-risk MDS (LR-MDS) or myelodysplastic/myeloproliferative neoplasms (MDS/MPN).

**Methods:**

Patients in CANFIRE were refractory, intolerant to, or ineligible for erythropoiesis-stimulating agent (ESA) treatment and received canakinumab 200 mg subcutaneously every 21 days for up to eight cycles within the first six months of treatment. Patients in LUCAS were either refractory or intolerant to ESAs (cohort A) or ESA-naïve with serum erythropoietin levels >200 U/L (cohort B); all patients received emavusertib 200–300 mg orally twice daily for 21 days in up to four 28-day cycles. The primary endpoint for both trials was hematologic improvement–erythroid per International Working Group 2018 criteria at the end of the planned treatment period. CANFIRE was registered at ClinicalTrials.gov (NCT05237713; registered January 21, 2022). LUCAS was registered in the EU Clinical Trials Register (EudraCT 2020-003986-20; registered March 15, 2021) and at ClinicalTrials.gov (NCT05178342; registered November 2, 2021).

**Results:**

Here we show that of 11 patients enrolled in CANFIRE and 36 patients enrolled in LUCAS, no patients in either trial achieved the primary endpoint. Canakinumab treatment was well-tolerated, with no reported serious adverse events (SAEs) and no patients died in the CANFIRE trial. In the LUCAS trial, 15 patients reported SAEs, and four patients died. CANFIRE and LUCAS were terminated early due to inadequate patient recruitment and after a pre-planned interim futility analysis, respectively.

**Conclusions:**

Inhibition of IL-1β or IRAK4 signaling with canakinumab or emavusertib did not result in hematologic improvement in patients with LR-MDS.

## Plain language summary

There is evidence that the drugs canakinumab and emavusertib could be useful for treating patients with certain blood cancers, specifically lower-risk myelodysplastic neoplasms (LR-MDS) or myelodysplastic/myeloproliferative neoplasms (MDS/MPN). The CANFIRE and LUCAS trials were carried out to see whether canakinumab or emavusertib are safe and effective when used for the treatment of patients with LR-MDS or MDS/MPN. Patients in the CANFIRE trial received canakinumab, and patients in the LUCAS trial received emavusertib. The primary endpoint of both trials was a reduction in the number of blood transfusions a patient needed and/or an increase in their hemoglobin level. No patients in either trial achieved the primary endpoint, and neither canakinumab nor emavusertib alone appears to be sufficient to treat patients with LR-MDS. Although neither drug was effective when used alone, future studies should investigate combination treatments, alternative treatment approaches, potentially longer treatment durations, and better selection of patients who are most likely to benefit.

## Abstract (web summary)

Platzbecker et al., evaluate the efficacy and safety of canakinumab or emavusertib in two phase II trials of patients with lower-risk myelodysplastic syndromes or myelodysplastic/myeloproliferative neoplasms who are ineligible for, refractory to, or intolerant of ESAs. Neither treatment achieves hematologic improvement–erythroid responses, canakinumab is well tolerated, and both trials are terminated early because of inadequate recruitment or futility.

## Introduction

Myelodysplastic neoplasms (MDS) and myelodysplastic/myeloproliferative neoplasms (MDS/MPN) are hematologic malignancies characterized by ineffective hematopoiesis and an increased risk of progression to acute myeloid leukemia (AML)^1–6^. There is increasing evidence to suggest that the pathogenesis of these disorders involves the upregulation of inflammatory signaling, particularly those pathways involving the proinflammatory cytokine interleukin-1β (IL-1β) and the toll-like receptor (TLR) signaling mediator interleukin-1 receptor-associated kinase 4 (IRAK4)^7–13^. The IL-1β pathway is upregulated in patients with MDS, and preclinical studies suggest that targeting the IL-1β inflammatory signaling pathway may rescue aberrant myeloid differentiation and ineffective erythropoiesis^7,14,15^. Similarly, a number of genetic lesions associated with MDS have been shown to induce alterations in IRAK signaling, and IRAK4 has been found to be the dominant alternatively spliced isoform in MDS and AML^10,11,16^.

Canakinumab is an anti-IL-1β monoclonal antibody that has been approved in the USA and Europe for the treatment of diseases, including periodic fever syndromes (including cryopyrin-associated periodic fever syndromes (CAPS) and tumor necrosis factor receptor-associated periodic syndromes (TRAPS)) and active Still’s disease (including adult-onset Still’s disease and systemic juvenile idiopathic arthritis)^17,18^. Canakinumab has been shown to increase the colony-forming activity of healthy donor HSPCs exposed to *SF3B1mut* MDS-derived monocytes in vitro and has been tested in patients with lower-risk MDS (LR-MDS)^7,8^. The phase 2 CANFIRE trial aimed to evaluate the efficacy and safety of canakinumab for the treatment of patients with LR-MDS or MDS/MPN with symptomatic anemia who are refractory to, intolerant to, or ineligible for treatment with erythropoiesis-stimulating agents (ESAs).

Emavusertib (CA-4948) is an oral small-molecule inhibitor of IRAK4 that has shown promising results in early-phase trials for hematologic malignancies^10,11^. Emavusertib has been shown to inhibit TLR and IL-1R pathway activity and has shown promising anti-cancer activity in patients with higher-risk MDS^10,11,19^. The phase 2 LUCAS trial aimed to evaluate the safety, tolerability, and efficacy of emavusertib monotherapy for the treatment of anemia in patients with LR-MDS who are not currently receiving ESAs.

Here we report efficacy and safety data from the CANFIRE and LUCAS trials. Of 11 patients enrolled in CANFIRE and 36 patients enrolled in LUCAS, no patients in either trial have achieved the primary endpoint, and both trials have been terminated early due to inadequate patient accrual, following a pre-planned futility analysis. The purpose of this joint report is comparative scientific interpretation rather than presentation of a unified interventional trial program. The rationale for joint reporting is to provide a comparative view of two independently conducted academic phase 2 trials targeting complementary inflammatory pathways in LR-MDS-associated anemia, thereby enabling a broader translational interpretation of negative efficacy findings.

## Methods

### Study design and oversight

The CANFIRE and LUCAS trials were independent investigator-initiated phase 2 studies conducted under separate protocols with different investigational agents and separate statistical analysis plans. They are presented together in this manuscript as complementary academic trials evaluating distinct anti-inflammatory strategies in a comparable LR-MDS setting. No pooled efficacy analysis was prespecified or performed.

**Fig. 1:**
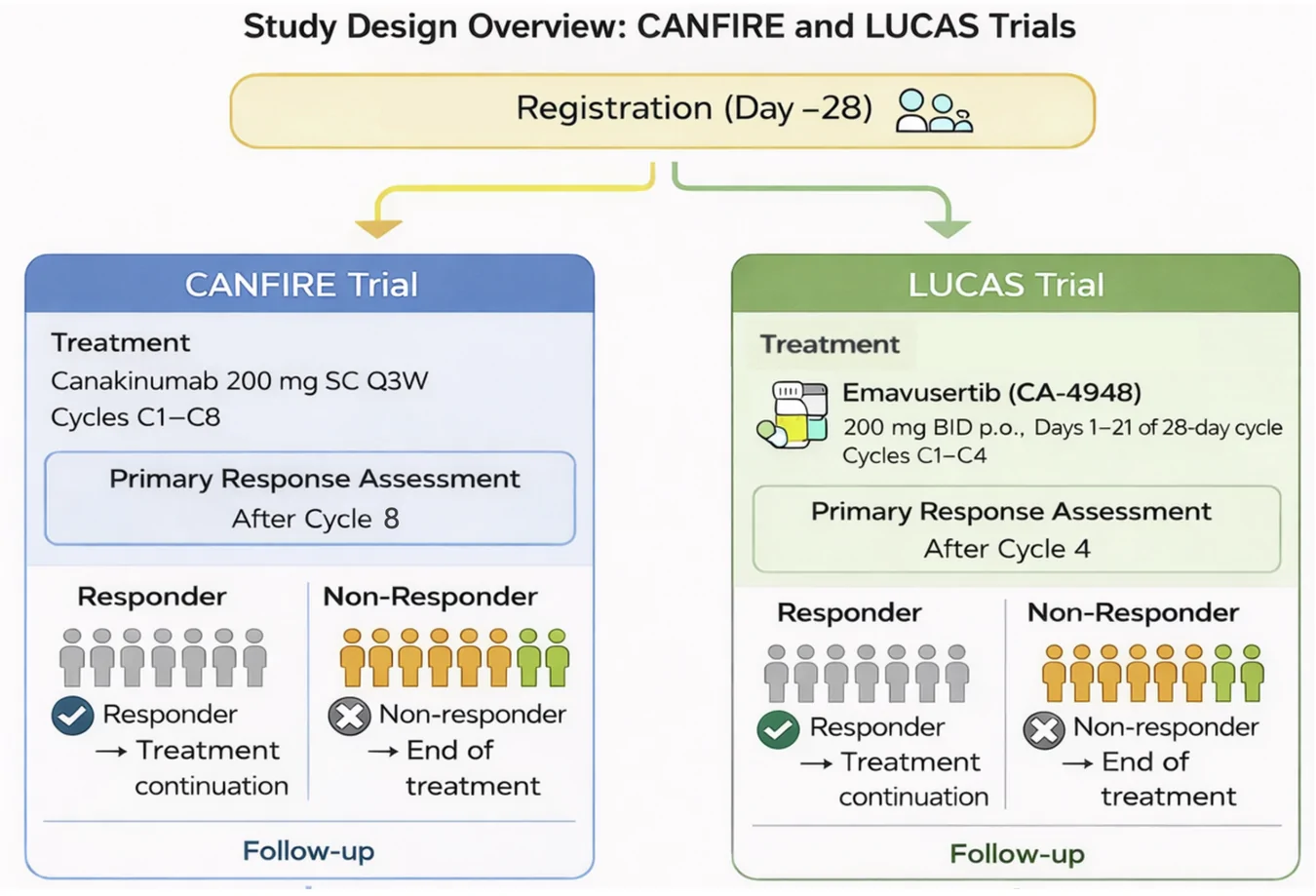
Study design of the CANFIRE and LUCAS trials. This figure summarizes key design elements of two independent studies for comparative purposes only and does not represent a basket trial, platform trial, or shared master protocol.

The CANFIRE trial was an investigator-initiated, phase 2, single-arm, open-label, multicenter study to evaluate the effects of canakinumab monotherapy on anemia in patients with revised international prognostic scoring system (IPSS-R) very low-, low-, or intermediate-risk MDS or MDS/MPN who are not currently receiving treatment with ESAs (Fig. 1).

The LUCAS trial was an investigator-initiated, phase 2, open-label, multicenter study to evaluate the effects of emavusertib monotherapy on anemia in patients with IPSS-R very low-, low-, or intermediate-risk MDS who are not currently receiving treatment with an ESA. Patients in the LUCAS trial were enrolled into two distinct cohorts: cohort A included patients who had prior exposure to and were refractory or intolerant to ESA, whereas cohort B included ESA-naive patients with serum erythropoietin levels greater than 200 U/L (Fig. 1).

Both the CANFIRE and LUCAS trials were conducted in accordance with the principles of the Declaration of Helsinki, all applicable national laws, and the ICH Guideline for Good Clinical Practice ICH E6 (R2). Ethical approval for both the LUCAS and CANFIRE trials was obtained from the Ethics Committee of the Medical Faculty of the University of Leipzig (reference nos. 183/21-ff and 448/21-ff, respectively), all patients provided written informed consent. Both the CANFIRE and LUCAS trials are registered at EudraCT (Lucas: 2020-003986-20, https://www.clinicaltrialsregister.eu/ctr-search/trial/2020-003986-20/DE, date of registration: March 15, 2021), CTIS (Transitional trial Canfire: 2024-515886-33-00, https://euclinicaltrials.eu/search-for-clinical-trials/?lang=en&EUCT = 2024-515886-33-00, date of registration: July 9, 2024) and ClinicalTrials.gov (Lucas: NCT05178342, https://clinicaltrials.gov/study/NCT05178342, date of registration: November 2, 2021; Canfire: NCT05237713, https://clinicaltrials.gov/study/NCT05237713, date of registration: January 21, 2022).

### Patients

Patients eligible for either study were aged ≥18 years and had a centrally confirmed diagnosis according to the 2016 WHO classification. Eligible disease categories comprised very low-, low-, or intermediate-risk MDS with an IPSS-R score ≤3.5, MDS/MPN with <10% bone marrow blasts, and CMML with a low or intermediate CPSS score. Eligible MDS/MPN entities included MDS/MPN-RS-T, MDS/MPN-u, aCML, and CMML. LUCAS specifically required de novo MDS or MDS/MPN. Symptomatic anemia had to be documented during the 16-week baseline period preceding formal inclusion and was defined as non-transfusion-dependent anemia (< 3 RBC transfusions and a mean hemoglobin level <10 g/dL), low transfusion burden (3–7 RBC transfusions in at least two transfusion episodes), or high transfusion burden (≥ 8 RBC transfusions). A defined transfusion strategy, including a documented transfusion trigger threshold, was required.

In CANFIRE, patients additionally had to have relapsed after, be refractory or intolerant to, or be ineligible for ESA treatment; ESA ineligibility was defined as an endogenous serum erythropoietin level ≥200 U/L. Key exclusion criteria included ECOG performance status >2; prior allogeneic or autologous stem cell transplantation; a history of AML; ANC ≤ 0.5 Gpt/L; platelet count <30 Gpt/L; protocol-defined renal, hepatic, infectious, cardiovascular, immunologic, or malignant comorbidities; prior canakinumab treatment; and receipt of prohibited cytotoxic, immunosuppressive, hematopoietic growth factor, disease-modifying, or experimental treatments within 14 days before registration or during study treatment.

In LUCAS, patients also had to have no available option for an approved MDS therapy, as determined by the treating physician. Cohort A comprised patients previously exposed to ESAs who were refractory or intolerant and had received no ESA during the 28 days preceding formal inclusion. Cohort B comprised ESA-naive patients with a serum erythropoietin level >200 U/L. Key exclusion criteria included ECOG performance status ≥3; protocol-defined unacceptable organ function; prior treatment with azacitidine or decitabine; exposure within 14 days before registration to ESA, luspatercept, G-CSF, GM-CSF, lenalidomide, or another prohibited investigational treatment; iron chelation initiated <56 days before registration; and protocol-defined surgical, infectious, cardiovascular, malignant, reproductive, or regulatory exclusions. The complete eligibility criteria are provided in the study protocols (Supplementary Appendix).

### Study treatment

Participants enrolled in the CANFIRE trial received canakinumab, a human monoclonal antibody that targets IL-1β, at a dose of 200 mg administered subcutaneously every 21 days for either eight cycles (6 months) for non-responders or up to 3 years for responders. For patients who experienced tolerability problems, the dose of canakinumab could be reduced to 150 mg or, if necessary, 100 mg in the following cycle. Treatment would be stopped in the event of disease progression (as determined by the investigator per 2018 IWG Revised Response Criteria for MDS^20^), withdrawal of consent, loss of contact, physician decision, or grade 3–4 toxicity that was unresponsive to dose reduction.

Patients enrolled in the LUCAS trial were assigned to one of two cohorts: ESA-exposed patients who were refractory or intolerant to ESAs were enrolled in cohort A, whereas ESA-naive patients with serum erythropoietin level >200 U/L were enrolled in cohort B. Patients in both cohorts received emavusertib (CA-4948), an IRAK4 inhibitor, administered orally at a starting dose of 300 mg twice daily (for a total daily dose of 600 mg) for 21 consecutive days in 28-day cycles. Following the occurrence of a rhabdomyolysis event, a protocol amendment was implemented, and the dose of emavusertib was reduced to 200 mg twice daily (total daily dose 400 mg) as a safety measure. Patients were treated for four cycles (16 weeks) until the primary endpoint assessment. In the event of progressive disease (as determined by the investigator per 2018 IWG^20^), intolerable toxicity, withdrawal from the trial, or study termination, treatment was discontinued. In the event of grade ≥3 non-hematologic toxicity (excluding grade ≥3 nausea or vomiting that resolves to grade <2 within 3 days with appropriate treatment, grade ≥3 fatigue lasting ≤7 days, or grade ≥3 electrolyte disturbance resolving to grade ≤1 or baseline within 3 days), or grade 4 neutropenia or thrombocytopenia in the absence of pre-existing condition of disease progression, treatment was held until recovery to grade ≤2, with subsequent dosing reduced by one level (150 mg for dose level −1 and 100 mg for dose level −2). In the event of other adverse events, treatment could be withheld for up to 28 days, with treatment discontinued if the adverse event had not resolved by the end of this time; treatment could be resumed (with dose reduced by one dose level) after more than 28 days where there was evidence of clinical benefit and if continuing treatment is judged by the investigator to be in the best interest of the patient.

### Endpoints

The primary endpoint for both trials was the proportion of patients with a hematologic response–erythroid (HI-E) per IWG 2018 criteria^20^. Secondary endpoints in both trials included HI-E response duration, time to HI-E response, changes in hemoglobin level during the course of treatment versus baseline, RBC transfusion independence ≥8 weeks and ≥12 weeks, and safety. RBC transfusion rates during the screening period (“before inclusion”, up to a maximum of 130 days before inclusion) and the treatment period (“after inclusion”, up to the end of treatment visit) were calculated by dividing the number of RBC transfusions received by the length of the respective period. Safety was systematically assessed in both trials using the NCI Common Terminology Criteria for Adverse Events.

In the CANFIRE trial, the primary endpoint was assessed 21 days ( ± 3 days) after patients received their eighth dose of canakinumab. The end of treatment visit was performed 28 days ( ± 3 days) after the last dose of canakinumab. A follow-up visit was carried out by telephone 56 days ( ± 14 days) after the end of the treatment visit.

In the LUCAS trial, the primary endpoint was assessed after four treatment cycles. The end of treatment visit was performed 7 days after the last dose of emavusertib. A safety follow-up visit was carried out 28 days ( ± 3 days) after the last dose of emavusertib, with further survival follow-up assessments performed by telephone in 3- to 6-month intervals for up to 2 years to determine survival status and treatment-related adverse events.

Longitudinal health-related quality of life (HRQoL) was assessed using validated patient-reported outcome measures. In CANFIRE, HRQoL was evaluated using the EORTC QLQ-C30 and the disease-specific QUALMS questionnaire, whereas in LUCAS quality of life was assessed using the EORTC QLQ-C30 and EORTC QLQ-FA12 instruments. HRQoL analyses were exploratory and descriptive. No formal confirmatory statistical testing was prespecified for HRQoL endpoints.

### Translational analysis

In both studies, translational biospecimen collection was prospectively specified in the study protocols and included scheduled bone marrow (BM) and peripheral blood (pB) sampling at predefined study visits. The cytokine and growth factor analyses presented here were exploratory and hypothesis-generating. In both studies, BM and pB samples were collected from consenting patients (LUCAS, *n* = 32; CANFIRE, *n* = 6) during the scheduled screening and response assessments. Inflammatory cytokine and growth factor concentrations in plasma were quantified using Luminex xMAP technology (Milliplex human cytokine/chemokine/growth factor panel A 38 plex, Merck, Darmstadt, Germany). Results below the detection limit were adjusted to 0 pg/ml to permit their inclusion in the analysis. We acknowledge that assigning values below the lower limit of detection to zero may lead to underestimation of low-level cytokine expression and reduced variability at the lower end of the distribution. However, this potential bias is systematic and non-differential, as all samples were processed uniformly, and therefore unlikely to account for the group-specific or longitudinal patterns.

### Statistics and reproducibility

In the CANFIRE trial, the planned sample size was calculated based on the null hypothesis that HI-E response probability is ≤5% and the alternative hypothesis that HI-E response is ≥20%; using a one-sided significance level of 2.5% and requiring a power of 85% provides a sample size of 41 patients with the exact binomial test. Assuming approximately nine patients fail to start treatment, a target sample size of 50 was determined. The CANFIRE full analysis set was defined as all patients who started treatment. The primary endpoint was to be evaluated using the exact binomial test, with the HI-E response rate to be estimated with an exact two-sided 95% confidence interval (CI), and duration of response to be evaluated by Kaplan–Meier plot and two-sided 95% CI.

In the LUCAS trial, each cohort was determined to constitute an independent sub-study, with each sub-study using the optimal Simon 2-stage phase 2 design^21^, with up to 42 patients to be enrolled in each of the two cohorts. Sample sizes were calculated based on a null hypothesis that the HI-E response probability is ≤5% and an alternative hypothesis that HI-E response is ≥20%. For each cohort, patients were enrolled in two stages with the option to stop enrollment early for futility based on interim analysis of the first 21 patients; enrollment was to be discontinued if fewer than two responses were documented in the first 21 patients included. The LUCAS full analysis set was defined as all patients who received any treatment. Response was estimated with an exact two-sided 95% CI and described based on initial transfusion dependency. No data were imputed in these statistical analyses.

For graphical visualization and statistical analyses of plasma measurements, GraphPad Prism 8 software (GraphPad Software, San Diego, USA) was used. Paired data were analyzed using the Wilcoxon matched-pairs signed rank test. Spider plots were generated using Microsoft Excel (Microsoft Corporation, Redmond, USA). Heatmaps were generated with R Studio (https://www.r-project.org) applying the ComplexHeatmap package^22^.

### Ethics

Ethical approval for both the LUCAS and CANFIRE trials was obtained from the Ethics Committee of the Medical Faculty of the University of Leipzig (reference nos. 183/21-ff and 448/21-ff, respectively).

## Results

For the purpose of clarity, results for each trial are presented separately below, followed by an integrated interpretation in the “Discussion”.

### CANFIRE: patients

As of data cutoff (1 December 2025 for CANFIRE), 11 patients had been enrolled in the CANFIRE trial between 26 April 2022 and 21 March 2024. The CANFIRE trial was terminated early due to an inadequate rate of recruitment.

**Fig. 2:**
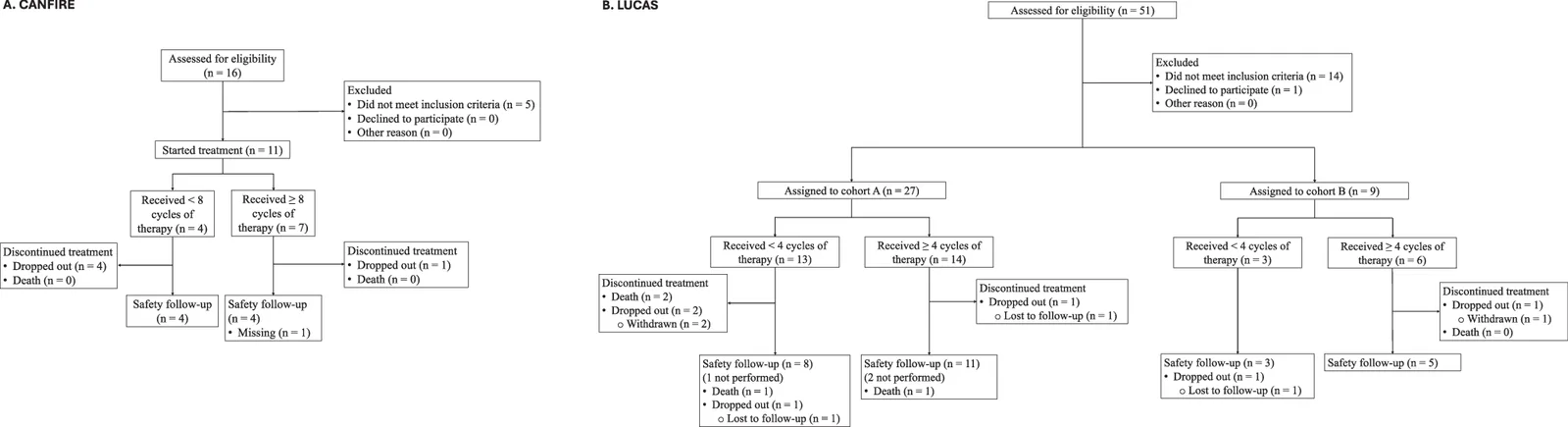
CONSORT study design and patient disposition. **A** Patient flow in the CANFIRE trial. **B** Patient flow in the LUCAS trial.

In CANFIRE at baseline, four patients (36%) had high transfusion burden (HTB, defined as requiring ≥8 RBC transfusions within the prior 16 weeks), two patients (18%) had low transfusion burden (LTB, defined as requiring 3–7 RBC transfusions within the prior 16 weeks in at least two transfusion episodes), and five patients (45%) were non-transfusion dependent (NTD, defined as requiring <3 RBC transfusions and mean hemoglobin level <10 g/dl within the prior 16 weeks). Of 11 patients enrolled in the CANFIRE trial, seven patients (64%) completed ≥ eight cycles of treatment within the first 6 months (Fig. 2A).

Baseline characteristics for patients in the CANFIRE trial are shown in Table 1 and Supplementary Table 1; additional baseline characteristics for individual patients, including IPSS-R karyotype details of mutated genes at screening, can be found in Supplementary Table 3. Despite the lack of objective clinical efficacy in the CANFIRE trial, two patients continued treatment beyond eight cycles due to perceived clinical benefits. A summary of concomitant medication received can be found in Supplementary Table 5.

**Table 1:** Baseline characteristics of patients in the CANFIRE and LUCAS trials

Patient characteristics	CANFIRE (*N* = 11)	LUCAS		
		Cohort A ESA-exposed (*N* = 27)	Cohort B ESA-naive (*N* = 9)	Total (*N* = 36)
Age (years), mean (SD)	65.3 (17.5)	72.3 (8.1)	66.4 (13.1)	70.9 (9.7)
Sex, *n* (%)				
Female	4 (36.4)	12 (44.4)	1 (11.1)	13 (36.1)
Male	7 (63.6)	15 (55.6)	8 (88.9)	23 (63.9)
Transfusion burden, *n* (%)^a^				
NTD	5 (45.5)	2 (7.4)	1 (11.1)	3 (8.3)
LTB	2 (18.2)	6 (22.2)	2 (22.2)	8 (22.2)
HTB	4 (36.4)	19 (70.4)	6 (66.7)	25 (69.4)
Disease, *n* (%)				
MDS	10 (90.9)	21 (77.8)	7 (77.8)	28 (77.8)
MDS/MPN	1 (9.1)	4 (14.8)	1 (11.1)	5 (13.9)
CMML	0	2 (7.4)	1 (11.1)	3 (8.3)
Disease subtype, *n* (%)				
MDS-MLD	2 (18.2)	5 (18.5)	3 (33.3)	8 (22.2)
MDS-MLD-RS	4 (36.4)	5 (18.5)	0	5 (13.9)
MDS-SLD	2 (18.2)	2 (7.4)	0	2 (5.6)
MDS-SLD-RS	1 (9.1)	6 (22.2)	1 (11.1)	7 (19.4)
MDS with excess blasts-1	0	0	1 (11.1)	1 (2.8)
MDS-EB2	1 (9.1)	0	0	0
MDS (unclassified)	–	3 (11.1)	2 (22.2)	5 (13.9)
MDS/MPN-RS-T	1(9.1)	3 (11.1)	1 (11.1)	4 (11.1)
MDS/MPN	–	1 (3.7)	0	1 (2.8)
CMML type I	–	2 (7.4)	0	2 (5.6)
CMML type II	–	0	1 (11.1)	1 (2.8)
IPSS-R risk category, *n* (%)				
Very low	0	2 (7.4)	0	2 (5.6)
Low	8 (72.7)	17 (63.0)	2 (22.2)	19 (52.8)
Intermediate	2 (18.2)	5 (18.5)	3 (33.3)	8 (22.2)
High	1 (9.1)	0	0	0
Missing	0	3 (11.1)	4 (44.4)	7 (19.4)
Bone marrow blasts, *n* (%)				
≤2%	8 (72.7)	18 (66.7)	2 (22.2)	20 (55.6)
>2–5%	1 (9.1)	5 (18.5)	2 (22.2)	7 (19.4)
5–10%	1 (9.1)	1 (3.7)	1 (11.1)	2 (5.6)
>=10%	1 (9.1)	0	0	0
Missing	0	3 (11.1)	4 (44.4)	7 (19.4)
Cytogenetics, *n* (%)				
Very good	0	3 (11.1)	0	3 (8.3)
Good	11 (100)	18 (66.7)	6 (66.7)	24 (66.7)
Intermediate	0	3 (11.1)	0	3 (8.3)
Missing	0	3 (11.1)	3 (33.3)	6 (16.7)
Hemoglobin, *n* (%)				
≥10 g/dL	0	2 (7.4)	0	2 (5.6)
≥8–<10 g/dL	5 (45.5)	14 (51.8)	3 (33.3)	17 (47.2)
<8 g/dL	6 (54.5)	9 (33.3)	3 (33.3)	12 (33.3)
Missing	0	2 (7.4)	3 (33.3)	5 (13.9)
Thrombocytes, *n* (%)				
≥100/nL	9 (81.8)	21 (77.8)	5 (55.6)	26 (72.2)
≥50/nL to <100/nL	2 (18.2)	2 (7.4)	0	2 (5.6)
<50/nL	0	2 (7.4)	1 (11.1)	3 (8.3)
Missing	0	2 (7.4)	3 (33.3)	5 (13.9)
Neutrophils				
≥0.8/nL	9 (81.8)	24 (88.9)	6 (66.7)	30 (83.3)
<0.8/nL	2 (18.2)	1 (3.7)	0	1 (2.8)
Missing	0	2 (7.4)	3 (33.3)	5 (13.9)

*CPPS* CMML prognostic scoring system, *MDS-MLD* MDS with multilineage dysplasia, *MDS-MLD-RS* MDS-MLD with ring sideroblasts, *MDS-SLD* MDS with single-lineage dysplasia, *MDS-SLD-RS* MDS-SLD with ring sideroblasts, *MDS/MPN* myelodysplastic/myeloproliferative neoplasm, *MDS/MPN-RS-T* MDS/MPN with ring sideroblasts and thrombocytosis.

^a^HTB is defined as requiring ≥8 RBC transfusions in the prior 16 weeks, LTB as requiring 3 to 7 RBC transfusions in the prior 16 weeks in at least two transfusion episodes, and NTD as requiring <3 RBC transfusions and a mean hemoglobin level <10 g/dl in the prior 16 weeks.

A complete listing of baseline characteristics for both studies can be found in Supplementary Table 1.

### CANFIRE: primary endpoint

**Fig. 3:**
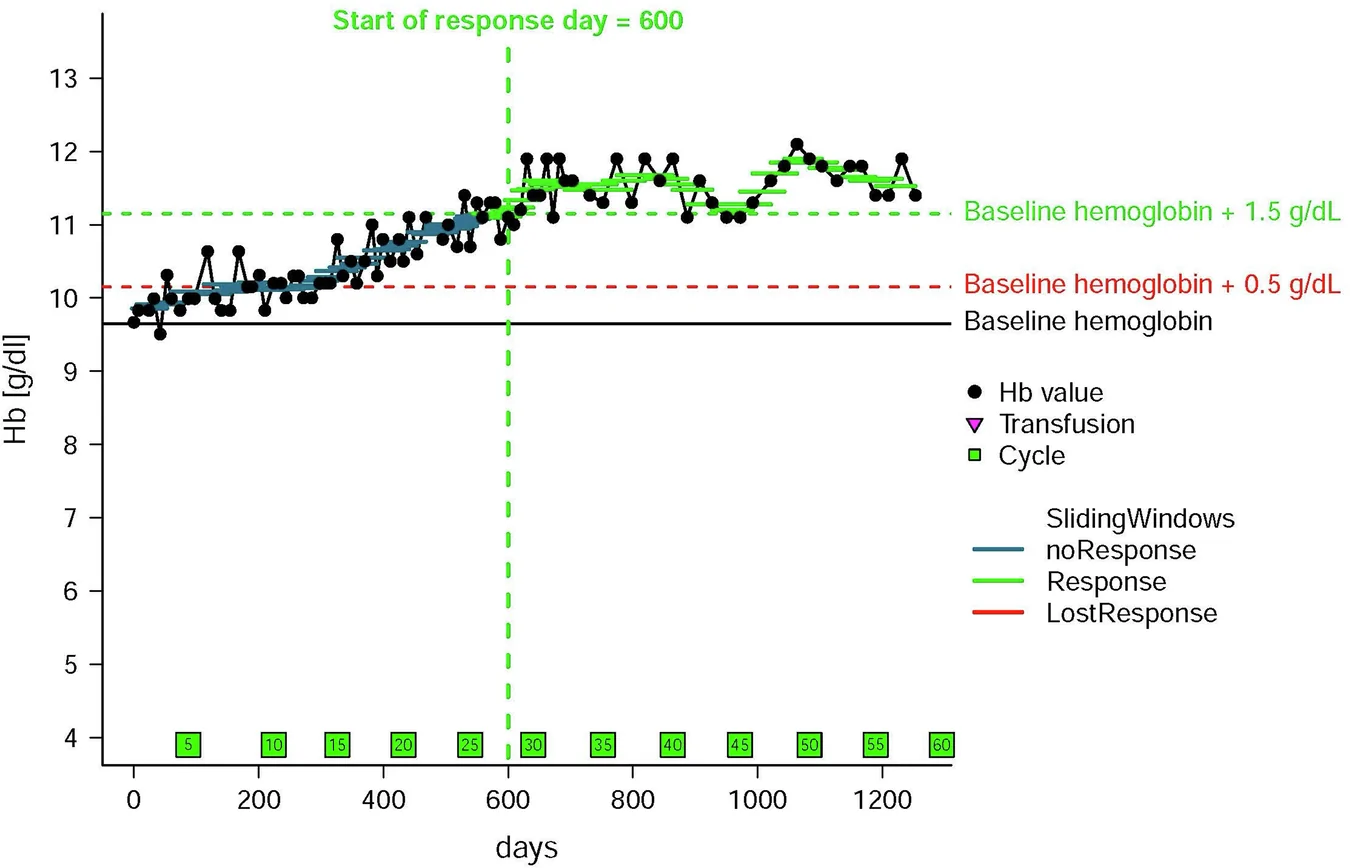
Achievement of hematologic improvement–erythroid response among 1 patient after cycle 28 in the CANFIRE trial.

None of the 11 fully evaluable patients in the CANFIRE trial achieved HI-E within the first 8 cycles of treatment. Notably, one patient with *ASXL1*, *SETBP1*, and *U2AF1* mutations achieved HI-E after 28 cycles (19.6 months) and maintained a response for over 5 months as of data cutoff (Fig. 3).

### CANFIRE: secondary endpoints

**Fig. 4:**
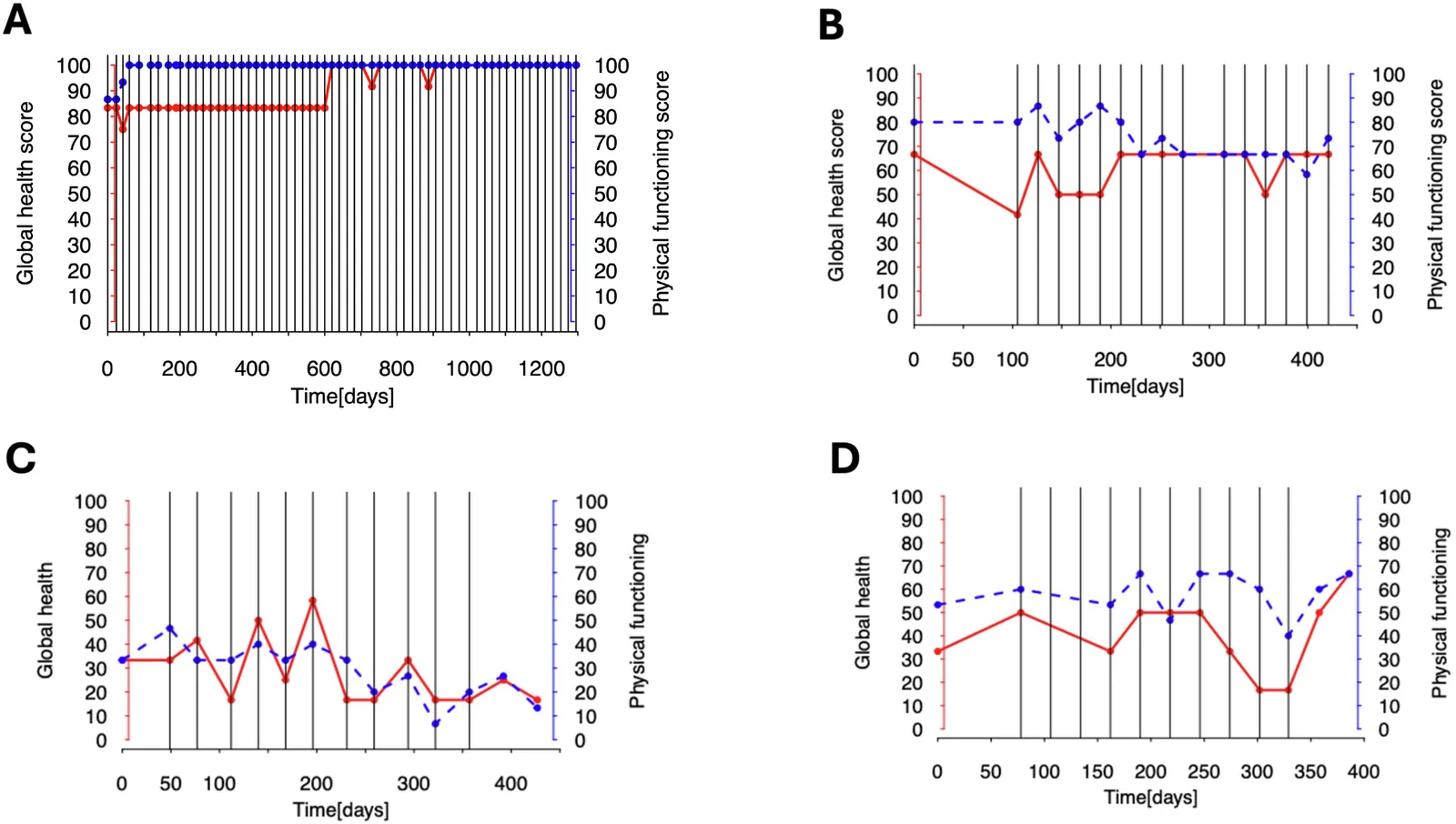
Quality-of-life trajectories in representative CANFIRE and LUCAS patients with perceived clinical benefit. **A**, **B** show representative CANFIRE patients who continued treatment based on perceived clinical benefit despite the absence of hematologic improvement–erythroid (HI-E). **C**, **D** depict representative LUCAS patients. Health-related quality of life was assessed using the EORTC QLQ-C30, with global health status (red solid line) and physical functioning (blue dashed line) shown over time. Individual trajectories demonstrate maintenance of patient-reported quality of life despite lacking objective erythroid response. Vertical lines indicate protocol-defined assessment time points.

Duration of HI-E response and time to HI-E response could not be evaluated in the CANFIRE trial. In the CANFIRE trial, two patients continued to receive treatment after the eighth cycle due to perceived clinical benefit. Figure 4 depicts representative individual patient trajectories illustrating HRQoL over time in patients reporting a perceived clinical benefit despite the absence of HI-E; observed HRQoL trajectories are descriptive and illustrate individual patient-reported outcomes over time. In CANFIRE, EORTC QLQ-C30 global health status and physical functioning scores showed no sustained deterioration, and QUALMS total and physical domain scores were preserved over time, with only minor early fluctuations followed by stabilization.

### CANFIRE: cytokine and growth factor dynamics

**Fig. 5:**
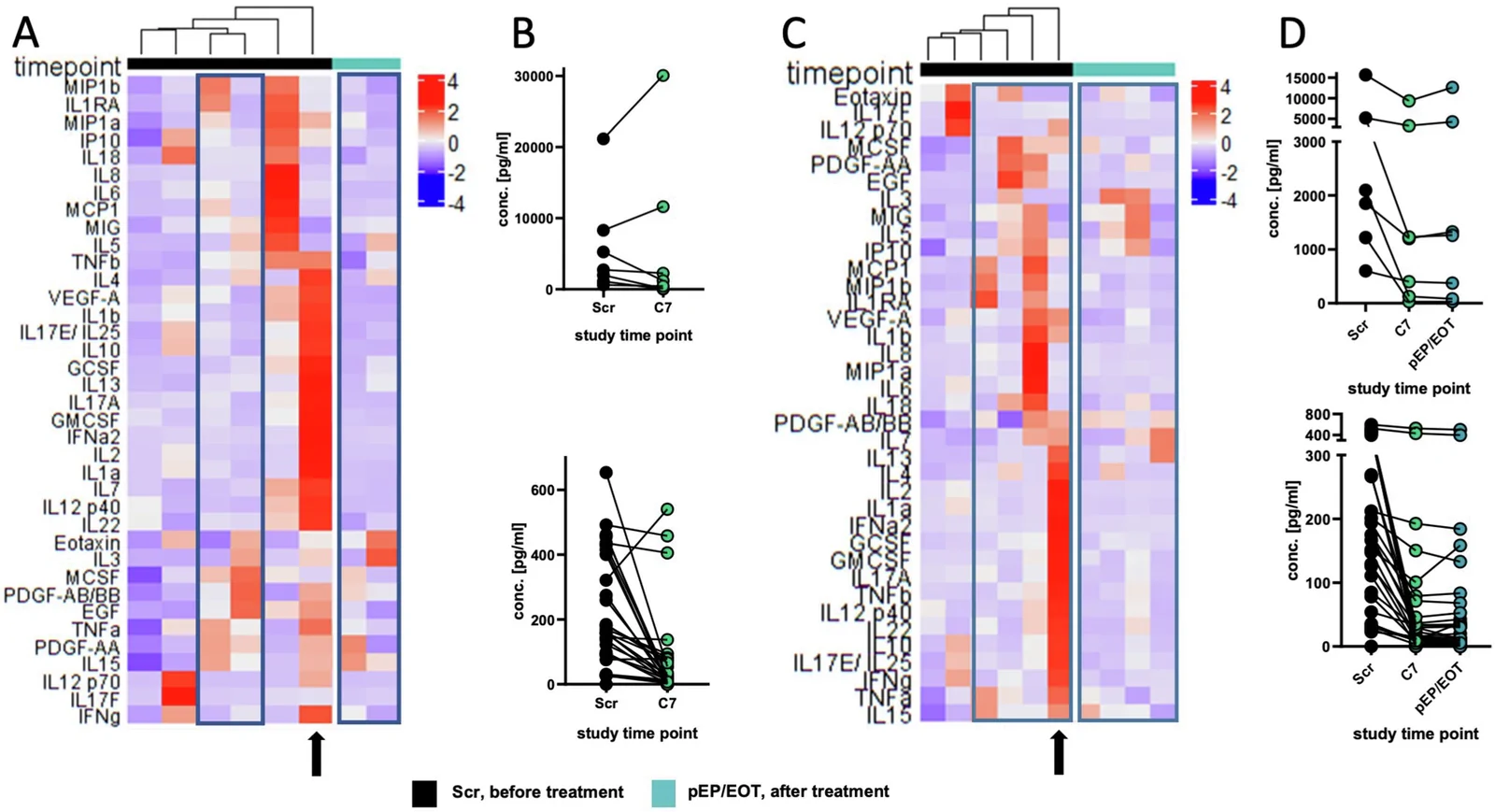
Plasma concentrations of cytokines and growth factors before and after treatment with canakinumab. Heatmaps of z scores of plasma concentrations in BM (**A**) and pB (**C**) at screening and after treatment. Each column represents one patient at the respective time point. The sequence of patients is based on unbiased clustering at screening and was then applied to pEP/EOT to facilitate comparison. Patients from whom both Scr and pEP/EOT samples were analyzed are framed on the heatmaps. The arrow beneath the heatmaps marks the patient for whom longitudinal samples of BM (**B**) and pB (**D**) were available. Each symbol in (**B**, **D**) represents a single analyzed parameter at the respective time point. Markers detected at high concentrations are shown collectively in the upper graphs, and those at lower concentrations in the lower graphs. Scr screening (before treatment), C7 treatment cycle 7, pEP/EOT primary endpoint assessment/end of treatment. *n* = 6 with longitudinal sampling available from 2 and 4 patients in BM and pB, respectively.

Plasma concentrations of cytokines and growth factors were analyzed in a subset of six patients. A trend towards lower cytokine levels in both BM and pB was observed in four patients from whom longitudinal samples were available (Fig. 5). For the one patient with HI-E response after 28 cycles, samples for cytokine measurements were not available.

### CANFIRE: safety

The incidence of adverse events occurring in ≥10% of patients in the CANFIRE trial is summarized in Supplementary Table 2. Canakinumab treatment was well-tolerated with no reported serious adverse events (SAEs), and no patient died in the CANFIRE trial.

### LUCAS: patients

As of data cutoff (April 14, 2025 for LUCAS), 36 patients had been enrolled in the LUCAS trial between January 19, 2022 and December 29, 2023. The LUCAS trial was terminated early following a pre-planned interim futility analysis.

Of the 36 patients enrolled in the LUCAS trial, 27 ESA-refractory/intolerant patients were enrolled in cohort A and nine ESA-naive patients were enrolled in cohort B (Fig. 2B). At baseline, 25 patients (70%; 19 in cohort A and six in cohort B) had HTB, eight (22%; 6 in cohort A and 2 in cohort B) had LTB, and three (8%; two in cohort A and one in cohort B) were NTD. Of 36 patients treated with emavusertib, 20 (55%) completed ≥ four cycles of treatment (*n* = 14 in cohort A, *n* = 6 in cohort B). One patient in cohort A withdrew early from the study before treatment could be documented but was considered as having started therapy and included in the full analysis set as adverse events and physical examinations on cycle 1, days 1, 8, and 15 were documented for this patient. Baseline characteristics for patients in the LUCAS trial are shown in Table 1 and Supplementary Table 1; additional baseline characteristics for individual patients, including IPSS-R karyotype details of mutated genes at screening, can be found in Supplementary Table 4. Despite the lack of objective clinical efficacy in the LUCAS trial, three patients continued treatment beyond four cycles due to perceived clinical benefits. A summary of concomitant medication received can be found in Supplementary Table 5.

### LUCAS: primary and secondary endpoint

None of the 36 patients in the LUCAS trial achieved the primary endpoint of HI-E. Duration of HI-E response and time to HI-E response could not be evaluated in the LUCAS trial. One patient in cohort A and two patients in cohort B continued to receive treatment after the fourth cycle due to perceived clinical benefit. Fig. 4 depicts representative individual patient trajectories illustrating HRQoL over time in patients reporting a perceived clinical benefit despite the absence of HI-E; observed HRQoL trajectories are descriptive and illustrate individual patient-reported outcomes over time.

### LUCAS: cytokine and growth factor dynamics

**Fig. 6:**
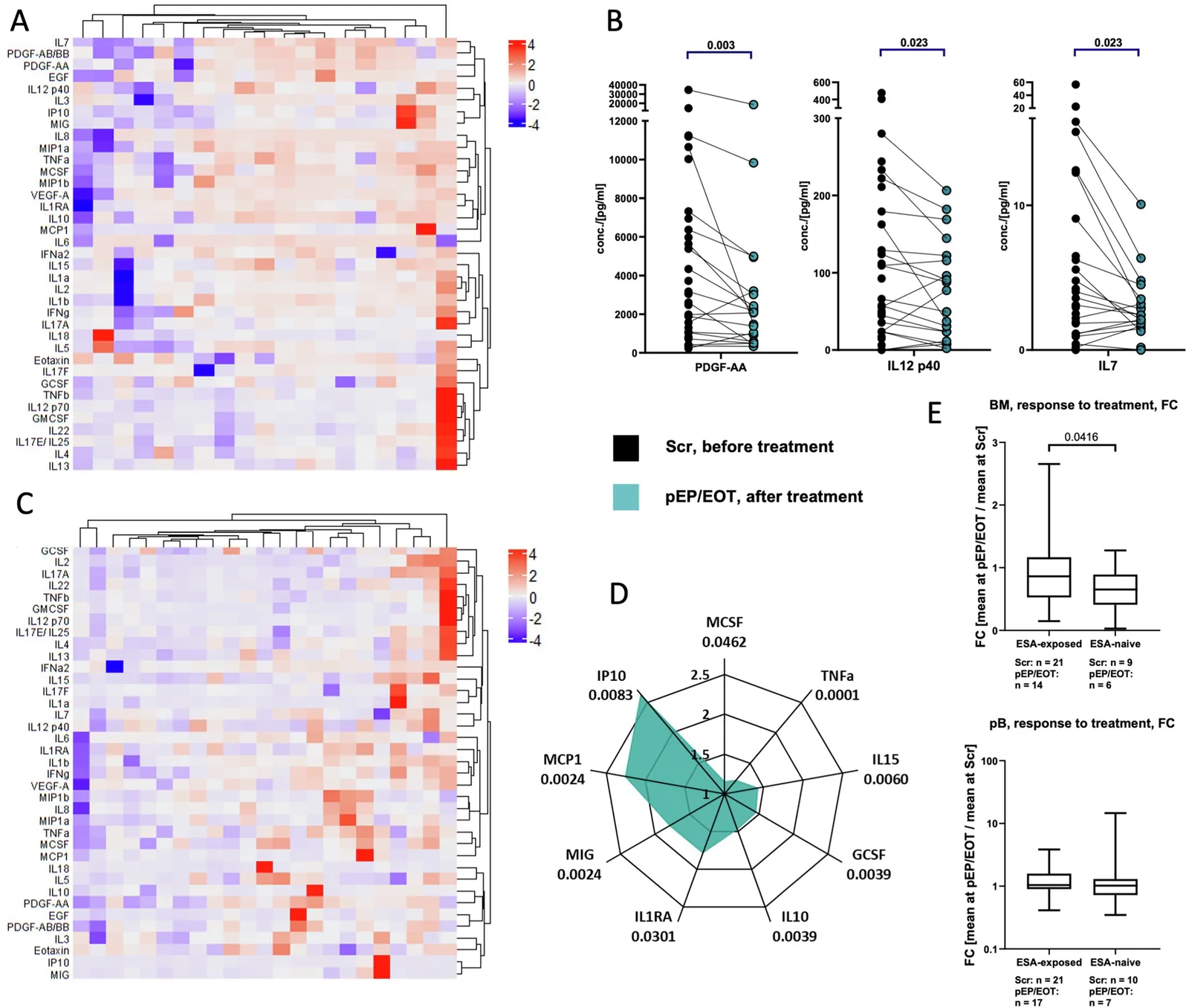
Plasma concentrations of cytokines and growth factors before and after treatment with emavusertib. Heatmap of z scores of plasma concentrations (difference: pEP/EOT-Scr) in BM (*n* = 19, **A**) and pB (*n* = 23, **C**). Each column represents one patient. **B** Plasma concentrations in BM were compared before and after treatment using the Wilcoxon matched-pairs signed rank test (32 patients analyzed, 19 patients at both time points). Each symbol represents one patient at the respective time point. Plots are shown only for significantly affected parameters. *P* values are indicated above the plots. **D** Plasma concentrations in pB were compared before and after treatment using the Wilcoxon matched-pairs signed rank test (32 patients analyzed, 23 patients at both time points). The spider plot summarizes the mean fold changes in significantly affected parameters. *P* values are indicated below the parameters. **E** Treatment-induced FC (pEP/EOT/Scr) of the mean of each parameter in BM (above) and pB (below) in ESA-exposed versus ESA-naive cohorts. The two cohorts were compared using Mann–Whitney rank sum test. *P* values are indicated above the plots. The number of analyzed patients (*n*) is indicated below the graphs. Before treatment = screening: Scr, after treatment = primary endpoint assessment/end of treatment: pEP/EOT, fold change: FC.

Plasma concentrations of cytokines and growth factors were analyzed in a subset of 32 patients. In the BM, anti-inflammatory modulation was evidenced by an overall reduction in cytokine and growth factor levels over 1–5 months of treatment, achieving statistical significance for IL-7, IL-12p40, and PDGF-AA (*n* = 19, Fig. 6A, B). Conversely, a range of pro- and anti-inflammatory markers in pB, including TNF-α, IP10, GCSF, MCSF, IL-15, IL-10, MCP1, MIG, and IL1RA, were significantly elevated during emavusertib treatment (*n* = 23, Fig. 6C, D). The changes of cytokine and growth factor levels in the BM tended to be more prominent in the ESA-naive cohort, whereas no difference between the cohorts was observed in pB (Fig. 6E).

### LUCAS: safety

The incidence of adverse events occurring in ≥10% of patients in the LUCAS trial is summarized in Supplementary Table 2. SAEs were reported in 15 patients, with nine of the 28 SAEs being potentially related to the study medication. One patient developed grade 2 rhabdomyolysis during the first treatment cycle while receiving emavusertib at a dose of 300 mg twice daily, which led to a dose reduction and consecutive treatment discontinuation. This adverse event prompted a protocol amendment; the daily dose was reduced to 200 mg twice daily. Four deaths occurred in the LUCAS trial, all in cohort A. Two deaths occurred during treatment: one patient died due to neutropenia that was judged to be possibly related to the study drug and was complicated by pneumonia, and one patient died due to gastroischemia that was not related to the study drug. Two additional patient deaths occurred outside treatment: one patient died due to cardiogenic shock, and the cause of death for one patient is unknown. No deaths occurred in cohort B. In addition, emavusertib treatment was accompanied by dose-dependent hematologic toxicity, most prominently a reduction in neutrophil counts during on-treatment periods, which appeared reversible during therapy (i.e., with treatment interruption and/or dose modification) (Supplementary Fig. 1A). In addition, we observed a dose-dependent decrease in platelet counts while patients were on treatment, consistent with an exposure-related myelosuppressive effect (Supplementary Fig. 1B). Although no HI-E responders were documented in LUCAS, these dose-dependent cytopenic effects nevertheless support a biological on-target effect of emavusertib in this patient population, even in the absence of objective erythroid responses.

## Discussion

Here we report the clinical and translational results of two phase 2 studies, CANFIRE and LUCAS, testing selective inhibition of IL-1β with canakinumab and inhibition of IRAK4 with emavusertib in patients with LR-MDS and MDS/MPN overlap syndromes suffering from symptomatic anemia after failure of, intolerance to, or ineligibility for ESA. Despite a strong mechanistic rationale and an acceptable safety profile in our cohorts, neither study met its primary efficacy endpoint, with no protocol-defined HI-E according to IWG 2018 criteria^20^ observed within the prespecified assessment windows. Although one patient achieved HI-E after 28 cycles, the delayed hemoglobin improvement was observed beyond the formal endpoint window and thus should be interpreted cautiously and considered hypothesis-generating only. However, the absence of HI-E should not be interpreted as synonymous with the absence of biological activity or as evidence against a pathogenic role of inflammation in LR-MDS. Rather, these findings suggest that inhibition of a single signaling node may be insufficient within a distributed and partially redundant inflammatory network. Furthermore, immunomodulatory interventions may influence marrow inflammatory tone, cytokine architecture, cellular stress signaling, stromal interactions, clonal fitness, or iron availability without producing immediate protocol-defined erythroid responses. These negative results provide important information for the field, because they challenge the assumption that targeting a single inflammatory node is sufficient to reverse ineffective erythropoiesis in transfusion-burdened lower-risk disease, and they help refine where and how anti-inflammatory strategies should be deployed in myeloid neoplasms. Although conducted independently, the two studies collectively inform the ongoing debate regarding the therapeutic relevance of inflammatory pathway inhibition in unselected LR-MDS.

A large body of work supports a causal role of innate immune activation and inflammasome signaling in the pathogenesis of MDS, linking inflammatory cytokine production to pyroptotic cell death, clonal expansion, and ineffective hematopoiesis^23^. In particular, activation of the NLRP3 inflammasome has been described as a hallmark feature in MDS that can drive the MDS phenotype across genotypes, with downstream IL-1 family cytokine signaling contributing to cytopenias and marrow dysfunction^9,24^. These observations are consistent with broader frameworks positioning chronic inflammation as a continuum from clonal hematopoiesis to overt myeloid neoplasia and AML, and as a determinant of disease evolution, symptom burden, and therapeutic vulnerability^12^. Within LR-MDS, more recent multi-omic analyses from our group indicate that inflammatory states are not uniform but cluster into distinct patterns that intersect with genotype and clinical phenotype^8^. We demonstrated that monocytes from LR-MDS patients can suppress colony formation of healthy HSPCs in co-culture and that this suppression can be mitigated ex vivo by IL-1β neutralization with canakinumab, supporting a plausible IL-1β-driven microenvironmental component, at least for defined subsets^8^. In parallel, mechanistic studies have identified genetically driven, therapeutically targetable activation of innate immune signaling through IRAK4. Mutations in spliceosome genes, particularly *U2AF1*, can induce expression of an oncogenic long isoform of IRAK4 (IRAK4-L), resulting in chronic innate immune signaling, NF-κB activation, and leukemic cell survival^16^. Inhibition of IRAK4 in these models reduces leukemic growth, providing a direct molecular link between splicing mutations and innate immune pathway dependence^16^.

Against this background, our CANFIRE findings should be interpreted in the context of the most relevant prospective clinical experience with canakinumab in LR-MDS. Rodriguez-Sevilla and colleagues recently reported a phase 2 trial of canakinumab in previously treated and transfusion-dependent LR-MDS in which the majority of patients (approximately 80%) had received prior disease-modifying therapy, most commonly hypomethylating agents^7^. Overall efficacy was limited, with an overall response rate of 17.4%, consisting of HI-E and platelet hematological improvement (HI-P) in three patients (13%) and one patient (4%), respectively^7^. Importantly, translational analyses revealed that clinical benefit was strongly associated with clonal architecture; however, given the limited number of evaluable paired samples, translational findings should be interpreted as exploratory signals rather than validated pharmacodynamic biomarkers. While canakinumab effectively suppressed IL-1β– and TNF-driven inflammatory programs in hematopoietic stem and progenitor cells, erythroid recovery occurred only in patients with low genetic complexity. In contrast, patients with multiple coexisting mutations or advanced clonal evolution showed no hematologic improvement despite biological target engagement^7^. These data underscore that IL-1β inhibition may confer clinical benefit only in biologically defined subgroups of LR-MDS, providing a mechanistic explanation for the absence of responders in also more advanced, genetically complex cohorts such as those enrolled in CANFIRE. Our CANFIRE results extend this observation by showing an absence of protocol-defined HI-E in our population and underscore that, under real-world academic trial constraints and in ESA-refractory, transfusion-burdened patients, IL-1β blockade as monotherapy is unlikely to deliver a robust or predictable anemia benefit. Importantly, the mechanistic heterogeneity described in LR-MDS suggests that IL-1β may be central only in a subset of patients characterized by specific inflammatory profiles and/or genotypes, and that enrolling biologically unselected cohorts risks diluting any benefit confined to such subgroups. Future studies should more comprehensively integrate iron biology, including serial assessment of IL-6, hepcidin, ferritin, transferrin saturation, soluble transferrin receptor, and related markers of iron-restricted erythropoiesis, as inflammatory anemia in LR-MDS may be mediated not only by progenitor suppression but also by dysregulated iron homeostasis. This may be particularly relevant in biologically defined subgroups such as *SF3B1*-mutated disease, where ex vivo erythroid colony restoration has been observed after IL-1β blockade.

The LUCAS results similarly require contextualization against emerging clinical data for emavusertib. Emavusertib (CA-4948) is a selective, orally bioavailable inhibitor of IRAK4 and FLT3 that has been developed across lymphoid and myeloid malignancies, with early-phase trials reporting antileukemic activity in relapsed/refractory AML and higher-risk MDS, particularly among patients with *FLT3* alterations or spliceosome mutations^11,25^. In contrast, our lower-risk, anemia-focused population did not show HI-E benefit, emphasizing that the therapeutic objective and disease biology in LR-MDS differ fundamentally from blast-driven AML or higher-risk MDS.

Several non-mutually exclusive biological and methodological explanations may account for the absence of responders in CANFIRE and LUCAS. One central concept is disease stage and irreversibility: our cohorts were ESA-refractory and mostly transfusion dependent, often reflecting prolonged disease biology with cumulative clonal evolution and microenvironmental damage. Inflammation can be an initiating and shaping force early in clonal hematopoiesis and early MDS, but later stages may become progressively dominated by cell-intrinsic differentiation failure and genetically encoded resistance, rendering single-node anti-inflammatory interventions insufficient to restore effective erythropoiesis^12^. Inflammation is described as a continuum and as a selective pressure in early myeloid neoplasia, while also acknowledging that inflammatory networks become entrenched, redundant, and increasingly coupled to malignant cell programs as disease progresses^12^. Consistent with this, the Rodriguez-Sevilla canakinumab trial^7^ reported that genetic complexity influenced clinical benefit despite demonstrable immunologic modulation, implying that once clonal architecture becomes complex, cytokine pathway inhibition alone may not be sufficient to translate into hematologic improvement^7^. A second major explanation is the lack of biomarker-driven enrichment. For IL-1β blockade, mechanistic work suggests that IL-1β-dependent suppression of hematopoiesis may be particularly relevant in defined inflammatory clusters and possibly in subtypes such as *SF3B1*-mutant disease^8^, in which ex vivo canakinumab can alleviate suppression in co-culture systems. For IRAK4 inhibition, the most compelling biological link is the spliceosome mutation–IRAK4-L axis, especially in *U2AF1-*mutant disease, where IRAK4-L expression is induced and therapeutically targetable^16^. The clinical emavusertib activity signals reported in AML and higher-risk MDS appear enriched in precisely these molecular contexts, including spliceosome-mutated and *FLT3*-altered disease^26^. In an unselected lower-risk cohort powered for an erythroid endpoint rather than antileukemic cytoreduction, any benefit confined to a molecularly defined subgroup would be statistically difficult to detect, particularly if the pathway dependence is more relevant to blast survival than to erythroid maturation. Third, redundancy and compensatory signaling within innate immune networks may blunt the clinical impact of single-node inhibition. NLRP3 inflammasome activation and IL-1 family cytokines interact with a broad inflammatory web that includes IL-18, alarmins, S100A8/A9-driven myeloid-derived suppressor cell loops, and multiple TLR-dependent inputs; suppressing one cytokine (IL-1β) may therefore be bypassed by other inflammatory mediators that continue to enforce ineffective hematopoiesis^9^. Similarly, inhibiting IRAK4 may not fully silence TLR/IL-1 receptor signaling because downstream signaling can be maintained through parallel adapters and kinases or through pathway rewiring; the broader emavusertib literature emphasizes both the centrality of IRAK4 within the myddosome and the complexity of innate immune survival signaling, supporting the plausibility of compensatory escape^11^. This redundancy concept is clinically relevant because it implies that single-agent IL-1β or IRAK4 blockade may require either combination approaches or a more precise selection of cases where the inhibited node is truly rate-limiting. However, although rational combination strategies may help overcome pathway redundancy and multifactorial erythroid failure, their development must carefully consider safety and feasibility in addition to biological rationale. In LUCAS, dose-dependent neutrophil and platelet reductions indicate that IRAK4 inhibition may narrow the therapeutic window when combined with other marrow-active therapies. Rare non-hematologic events such as muscle toxicity should also be considered in future regimen design. Combinations with erythroid maturation agents, hypomethylating agents, or other targeted therapies should therefore be explored in stepwise dose-finding studies with close safety monitoring and prospective biomarker integration. Such approaches may help identify combinations that are both biologically effective and clinically tolerable in selected LR-MDS populations. Fourth, endpoint kinetics and exposure duration may matter for an erythroid endpoint. Here, we assessed cytokine concentration in bone marrow plasma and peripheral blood 7 days after the last dose of emavusertib in the LUCAS trial and found divergent changes between the two tissues. This may be a result of differences between bone marrow and blood in terms of either drug penetration and turnover; the source and functional interaction of cytokines, or a difference in the capacity of the two tissues to compensate for an IRAK4 block, for instance by IRAK1^27^.

Anti-inflammatory niche remodeling could plausibly require prolonged exposure before translating into erythroid recovery, while formal IWG HI-E criteria impose a stringent threshold that may miss modest but clinically relevant shifts in transfusion interval or fatigue. The canakinumab experience in LR-MDS suggests that biologic modulation is detectable, yet clinical responses remain limited and appear dependent on host and clonal context; thus, it remains possible that a subset of patients could exhibit delayed benefit that is not captured within early fixed windows, particularly in small studies terminated for futility^7^. However, the consistent lack of any clear response signal across two different anti-inflammatory strategies in our cohorts argues that timing alone is unlikely to fully explain the negative results.

Other limitations include the relatively small size of the study population; the CANFIRE trial in particular was substantially limited by premature closure and low accrual, with only 11 treated patients included compared with the originally planned sample size. Consequently, estimates of clinical activity are imprecise and should be interpreted as descriptive rather than definitive. Similarly, correlative laboratory analyses were feasible in only six evaluable patients. These exploratory findings should therefore be interpreted cautiously and cannot be considered confirmatory evidence of mechanism or resistance biology. Importantly, the absence of protocol-defined HI-E responses in this small cohort does not exclude potential activity in biologically selected subgroups, which were not prospectively enriched in the present study. Given the limited sample size and exploratory analysis, HRQoL findings should be interpreted cautiously as supportive patient-centered observations rather than definitive treatment effects. Longitudinal assessment of variant allele frequencies was also not systematically available and therefore potential effects on clonal dynamics could not be evaluated in the present analysis. The conduct of CANFIRE and LUCAS also highlights structural challenges for academic trials of LR-MDS. Many patients with chronic anemia are managed long-term in community-based outpatient practices and may not be routinely referred to tertiary centers for trial participation. In addition, selective eligibility criteria, competing clinical studies, logistical burden of repeated study visits, and disruptions related to the COVID-19 pandemic and its aftermath may all negatively affect accrual and study feasibility.

Finally, our findings reinforce the idea that LR-MDS anemia is multifactorial and that “inflammation” is not a single actionable pathway but a layered phenotype. The seminal NLRP3 inflammasome work demonstrates a driver role for pyroptosis and inflammatory death in MDS, yet it also implies that effective therapeutic intervention may need to target the upstream inflammasome machinery, broader inflammatory circuits, or simultaneously address lineage-specific maturation failure with erythroid-directed agent^9^. Similarly, the spliceosome-IRAK4-L mechanism provides a compelling target in defined molecular subsets. In conclusion, CANFIRE and LUCAS demonstrate that, in unselected, ESA-refractory, transfusion-dependent LR-MDS/MDS-MPN with anemia, monotherapy with canakinumab or emavusertib is insufficient to induce clinically meaningful erythroid responses as defined by standard criteria. Moreover, the heterogeneous inflammatory landscape of LR-MDS strongly argues for biomarker-guided patient selection. Rather than applying anti-inflammatory therapies to unselected populations, future trials should incorporate composite immune or inflammatory scores integrating cytokine profiles, transcriptional inflammatory signatures, and clonal architecture to identify patients in whom innate immune activation represents a dominant disease driver. Such an immunologically informed stratification strategy may be essential to unlock the therapeutic potential of anti-inflammatory interventions in MDS. Future studies would require biomarker-guided patient selection, different endpoints, larger cohorts, and prospective translational integration. Future development of anti-inflammatory strategies in MDS should also prioritize biomarker-driven cohorts enriched for IL-1β-dependent inflammatory states or for spliceosome mutation–associated IRAK4-L biology, incorporate direct marrow pharmacodynamic readouts, and evaluate rational combinations that simultaneously address inflammatory network redundancy and erythroid maturation failure.

## Supplementary information

Supplementary Information

Description of Additional Supplementary Data

Supplementary Data 1

Supplementary Data 2

Supplementary Data 3

Supplementary Data 4

## Data Availability

Individual participant data that underlie the results reported in this article after deidentification (text, tables, figures, and appendices) and the study protocol, statistical analysis plan, informed consent form, will be made available immediately following publication of this article, with no end date, to investigators whose proposed use of the data has been approved by an institutional review board/ethics committee for individual patient meta-analysis; public registration of the meta-analysis is also required. Proposals should be directed to annesophie.platzbecker@ukdd.de. The source data underlying Fig. 3 can be found in Supplementary Data 1, the source data underlying Fig. 4 can be found in Supplementary Data 2, the source data underlying Fig. 5 can be found in Supplementary Data 3, and the source data underlying Fig. 6 can be found in Supplementary Data 4.
