# Supplementary material for: Canakinumab or emavusertib for anemia in lower-risk MDS: results of the CANFIRE and LUCAS phase 2, open-label, multicenter trials
Source: Commun Med (Lond). 2026 Sep 16;6:486. doi: 10.1038/s43856-026-01885-z (PMC13583006; doi:10.1038/s43856-026-01885-z)
Supplement: Supplementary file 1 [file 43856_2026_1885_MOESM1_ESM.pdf]

Supplementary Information

Canakinumab or Emavusertib for Anemia in Lower-Risk MDS: Results of the CANFIRE and LUCAS Phase 2, Open-Label, Multicenter Trials

Anne Sophie Platzbecker, Dominic Brauer, Katharina Zoldan, Philipp Kiewe, Daniel Sasca, Katja Sockel, Thomas Illmer, Aristoteles Giagounidis, Daniel Nowak, Kathrin Rieger, Friederike Wortmann, Katharina Götze, Martin Schmidt-Hieber, Karin Mayer, Ursula Vehling-Kaiser, Rudolf Weide, Stefan Mahlmann, Jens Przybilla, Susanne Melzer, Michael Cross, Marie Schneider, Dirk Hasenclever, Klaus H Metzeler, Uwe Platzbecker

Contents

Supplementary Tables………………………….……………………………………………………2

CONSORT 2025 checklist…………………….......…………………………………………………..17

Study locations…………………………………….……………………………………………………..21

Trial protocols and SAPs………………………. ……………………………………………………..28

Supplementary Table 1. Baseline characteristics for patients in the CANFIRE and LUCAS trials.

Patient characteristics	CANFIRE (N = 11)	LUCAS		
		Cohort A ESA-exposed (N = 27)	Cohort B ESA-naïve (N = 9)	Total (N = 36)
Age (years), mean (SD)	65.3 (17.5)	72.3 (8.1)	66.4 (13.1)	70.9 (9.7)
Sex, n (%)				
Female	4 (36.4)	12 (44.4)	1 (11.1)	13 (36.1)
Male	7 (63.6)	15 (55.6)	8 (88.9)	23 (63.9)
Transfusion burden, n (%)[a]				
NTD	5 (45.5)	2 (7.4)	1 (11.1)	3 (8.3)
LTB	2 (18.2)	6 (22.2)	2 (22.2)	8 (22.2)
HTB	4 (36.4)	19 (70.4)	6 (66.7)	25 (69.4)
Disease, n (%)				
MDS	10 (90.9)	21 (77.8)	7 (77.8)	28 (77.8)
MDS/MPN	1 (9.1)	4 (14.8)	1 (11.1)	5 (13.8)
CMML	0	2 (7.4)	1 (11.1)	3 (8.3)
Disease subtype, n (%)				
MDS-MLD	2 (18.2)	5 (18.5)	3 (33.3)	8 (29.6)
MDS-MLD-RS	4 (36.4)	5 (18.5)	0	5 (18.5)
MDS-SLD	2 (18.2)	2 (7.4)	0	2 (7.4)
MDS-SLD-RS	1 (0.09)	6 (22.2)	1 (11.1)	7 (25.9)
MDS with excess blasts-1	0	0	1 (11.1)	1 (3.7)
MDS with excess blasts-2	1 (9.1)	0	0	0
MDS (unclassified)	-	3 (11.1)	2 (22.2)	5 (18.5)
MDS/MPN-RS-T	1(9.1)	3 (11.1)	1 (11.1)	4 (14.8)
MDS/MPN	-	1 (3.7)	0	1 (3.4)
CMML type I	-	2 (7.4)	0	2 (7.4)
CMML type II	-	0	1 (11.1)	1 (3.4)
IPSS-R risk category, n (%)				
Very low	0	2 (7.4)	0	2 (5.6)
Low	8 (72.7)	17 (63.0)	2 (22.2)	19 (52.8)
Intermediate	2 (18.2)	5 (18.5)	3 (33.3)	8 (22.2)
High	1 (9.1)	0	0	0
Missing	0	3 (11.1)	4 (44.4)	7 (19.4)

Supplementary Table 1. Baseline characteristics for patients in the CANFIRE and LUCAS trials.

Patient characteristics	CANFIRE (N = 11)	LUCAS		
		Cohort A ESA-exposed (N = 27)	Cohort B ESA-naïve (N = 9)	Total (N = 36)
Bone marrow blasts, n (%)				
≤ 2%	8 (72.7)	18 (66.7)	2 (22.2)	20 (55.6)
> 2 to 5 %	1 (9.1)	5 (18.5)	2 (22.2)	7 (19.4)
5% to 10%	1 (9.1)	1 (3.7)	1 (11.1)	2 (5.6)
≥10%	1 (9.1)	0	0	0
Missing	0	3 (11.1)	4 (44.4)	7 (19.4)
Cytogenetics, n (%)				
Very good	0	3 (11.1)	0	3 (8.3)
Good	11 (100)	18 (66.7)	6 (66.7)	24 (66.7)
Intermediate	0	3 (11.1)	0	3 (8.3)
Missing	0	3 (11.1)	3 (33.3)	6 (16.7)
Hemoglobin, n (%)				
≥ 10 g/dL	0	2 (7.4)	0	2 (5.6)
≥ 8 to < 10 g/dL	5 (45.5)	14 (51.8)	3 (33.3)	17 (47.2)
< 8 g/dL	6 (54.5)	9 (33.3)	3 (33.3)	12 (33.3)
Missing	0	2 (7.4)	3 (33.3)	5 (13.9)
Thrombocytes, n (%)				
≥100/nL	9 (81.8)	21 (77.8)	5 (55.6)	26 (72.2)
≥50/nL to <100/nL	2 (18.2)	2 (7.4)	0	2 (5.6)
<50/nL	0	2 (7.4)	1 (11.1)	3 (8.3)
Missing	0	2 (7.4)	3 (33.3)	5 (13.9)
Neutrophils				
≥0.8/nL	9 (81.8)	24 (88.9)	6 (66.7)	30 (83.3)
<0.8/nL	2 (18.2)	1 (3.7)	0	1 (2.8)
Missing	0	2 (7.4)	3 (33.3)	5 (13.9)
CPSS risk category, n/N (%)				
Low	-	1/2 (50.0)	0	1/4 (25.0)
Intermediate 1	-	1/2 (50.0)	0	1/4 (25.0)
Intermediate 2	-	0	2/2 (100)	2/4 (50.0)

Supplementary Table 1. Baseline characteristics for patients in the CANFIRE and LUCAS trials.

Patient characteristics	CANFIRE (N = 11)	LUCAS		
		Cohort A ESA-exposed (N = 27)	Cohort B ESA-naïve (N = 9)	Total (N = 36)
WHO subtype, n (%)				
CMML-1	-	2/2 (100)	1/2 (50.0)	3/4 (75.0)
CMML-2	-	0	1/2 (50.0)	1/4 (25.0)

[a] HTB was defined as requiring ≥ 8 RBC transfusions within the prior 16 weeks; LTB was defined as requiring 3 to 7 RBC transfusions within the prior 16 weeks in at least two transfusion episodes, and NTD was defined as requiring < 3 RBC transfusions and mean hemoglobin level <10 g/dl within the prior 16 weeks. CMML, chronic myelomonocytic leukemia; CPPS, CMML prognostic scoring system; ESA, erythropoiesis-stimulating agent; HTB, high transfusion burden; IPSS-R, Revised International Prognostic Scoring System; LTB, low transfusion burden; MDS, myelodysplastic syndromes; MDS-MLD, MDS with multilineage dysplasia; MDS-MLD-RS, MDS-MLD with ring sideroblasts; MDS-SLD, MDS with single-lineage dysplasia; MDS-SLD-RS, MDS-SLD with ring sideroblasts; MDS/MPN, myelodysplastic/myeloproliferative neoplasm; MDS/MPN-RS-T; MDS/MPN with ring sideroblasts and thrombocytosis; NTD, non-transfusion dependent; RBC, red blood cell; WHO, World Health Organization.

Supplementary Table 2. Incidence of adverse events in the CANFIRE and LUCAS trials.

n (%)	CANFIRE (N = 11)	LUCAS Cohort A ESA-exposed (N = 27)	LUCAS Cohort B ESA-naïve (N = 9)
Patients with any adverse event	8 (72.7)	27 (100)	9 (100)
Number of patients with adverse event			
Fatigue	1 (9.1)	13 (48.1)	2 (22.2)
Diarrhea	1 (9.1)	12 (44.4)	3 (33.3)
Nausea	1 (9.1)	11 (40.7)	1 (11.1)
Anemia	1 (9.1)	10 (37.0)	0
Dizziness	0	7 (25.9)	1 (11.1)
Headache	0	6 (22.2)	1 (11.1)
Constipation	0	5 (18.5)	2 (22.2)
Myalgia	0	5 (18.5)	1 (11.1)
Neutrophil count decreased	1 (9.1)	5 (18.5)	0
Pain in extremity	0	5 (18.5)	1 (11.1)
Blood creatine phosphokinase increased	0	4 (14.8)	1 (11.1)
Decreased appetite	0	4 (14.8)	1 (11.1)
Night sweats	1 (9.1)	4 (14.8)	0
Syncope	0	4 (14.8)	0
Abdominal pain upper	0	3 (11.1)	2 (22.2)
Alanine aminotransferase increased	0	3 (11.1)	0
Coronavirus infection	0	3 (11.1)	0
Dyspepsia	0	3 (11.1)	0
Dyspnea	0	3 (11.1)	1 (11.1)
Muscle spasms	0	3 (11.1)	1 (11.1)
Myoglobin blood increased	0	3 (11.1)	0
Platelet count decreased	1 (9.1)	3 (11.1)	1 (11.1)
Pyrexia	1 (9.1)	3 (11.1)	2 (22.2)

Supplementary Table 2. Incidence of adverse events in the CANFIRE and LUCAS trials.

n (%)	CANFIRE (N = 11)	LUCAS	
		Cohort A ESA-exposed (N = 27)	Cohort B ESA-naïve (N = 9)
White blood cell count decreased	0	3 (11.1)	0
Pneumonia	0	1 (3.7)	3 (33.3)
COVID-19	0	1 (3.7)	2 (22.2)
Pruritis	0	1 (3.7)	2 (22.2)
Respiratory tract infection	1 (9.1)	0	2 (22.2)
Abdominal discomfort	0	0	1 (11.1)
Anxiety	0	0	1 (11.1)
Aortic aneurysm	0	0	1 (11.1)
Back pain	0	2 (7.4)	1 (11.1)
Blood bilirubin increased	2 (18.2)	1 (3.7)	0
Blood loss anemia	0	0	1 (11.1)
Cardiac failure	0	0	1 (11.1)
Cardiovascular disorder	0	1 (3.7)	1 (11.1)
Disease progression	0	0	1 (11.1)
Duodenitis	0	0	1 (11.1)
Factor II mutation	0	0	1 (11.1)
Gastrointestinal hemorrhage	0	0	1 (11.1)
Hematoma	1 (9.1)	0	1 (11.1)
Hematotoxicity	0	0	1 (11.1)
Hypoglycemia	0	0	1 (11.1)
Influenza	0	0	1 (11.1)
Intervertebral disc protrusion	0	0	1 (11.1)
Lower gastrointestinal hemorrhage	0	0	1 (11.1)
Malaise	0	0	1 (11.1)
Nasopharyngitis	2 (18.2)	0	1 (11.1)
Nervousness	0	1 (3.7)	1 (11.1)
Edema peripheral	0	2 (7.4)	1 (11.1)
Parathesia	0	2 (7.4)	1 (11.1)

Supplementary Table 2. Incidence of adverse events in the CANFIRE and LUCAS trials.

n (%)	CANFIRE (N = 11)	LUCAS Cohort A ESA-exposed (N = 27)	LUCAS Cohort B ESA-naïve (N = 9)
Peripheral sensorimotor neuropathy	0	0	1 (11.1)
Pharyngeal swelling	0	0	1 (11.1)
Rib fracture	0	0	1 (11.1)
Sacroilliac joint dysfunction	0	0	1 (11.1)
Stent-graft endoleak	0	0	1 (11.1)
Thrombocytopenia	0	1 (3.7)	1 (11.1)
Tinnitus	0	1 (3.7)	1 (11.1)
Visual impairment	0	0	1 (11.1)
Patients with ≥ 1 grade ≥ 3 adverse event	2 (1.8)	14 (51.9)	6 (66.7)
Patients with ≥ 1 serious adverse event	0	0	1 (11.1)
Patients with adverse events leading to death	0	2 (7.4)	0

Supplementary Table 3. Baseline characteristics of patients in the CANFIRE trial.

	Central morphology (WHO 2016) at screening	Transfusion dependency before inclusion[a]	Karyotype category (IPSS-R) at inclusion	IPSS-R risk category at inclusion	Mutated genes at screening (VAF/%, if available)
Patient 1	MDS-MLD-RS	HTB	good	low	ASXL1 (1.4), JAK2(1.9), SF3B1 (30.8)
Patient 2	MDS-SLD-RS	LTB	good	low	ASXL1 (1.9, 1.1, 1.7, 1.1), SF3B1 (47.2), STAG2 (94), TET2 (42.7)
Patient 3	MDS-MLD-RS	HTB	good	low	SF3B1 (43.8), TET2 (41.4)
Patient 4	MDS-MLD-RS	HTB	good	low	SF3B1 (45.8), MPL (13.7)
Patient 5	MDS-SLD	HTB	good	intermediate	TP53 (34.2)
Patient 6	MDS-SLD	LTB	good	low	NF1 (2.5, 5.8)

[a] HTB was defined as requiring ≥ 8 RBC transfusions within the prior 16 weeks; LTB was defined as requiring 3 to 7 RBC transfusions within the prior 16 weeks in at least two transfusion episodes, and NTD was defined as requiring < 3 RBC transfusions and mean hemoglobin level <10 g/dl within the prior 16 weeks. HTB, high transfusion burden; IPSS-R, Revised International Prognostic Scoring System; LTB, low transfusion burden; MDS, myelodysplastic syndromes; MDS-MLD, MDS with multilineage dysplasia; MDS-MLD-RS, MDS-MLD with ring sideroblasts; MDS-SLD, MDS with single-lineage dysplasia; MDS-SLD-RS, MDS-SLD with ring sideroblasts; NTD, non-transfusion dependent; VAF, variant allele frequency; WHO, World Health Organization.

Supplementary Table 4. Baseline characteristics of patients in the LUCAS trial.

	Cohort[a]	Central morphology (WHO 2016) at screening	Transfusion dependency before inclusion[c]	Karyotype category (IPSS-R) at inclusion	IPSS-R risk category at inclusion	Mutated genes at screening (VAF/%, if available)	Mutations at other time point (VAF/% if available)
Patient 1	A	MDS-MLD-RS[b]	LTB	good	low	ASXL1 (2), JAK2 (2), SF3B1 (41)	at pEP: ASXL1 (3), PRPF8 (32), SF3B1 (33)
Patient 2	A	MDS/MPN-RS-T	HTB	good	low	SF3B1 (48), BRCC3 (2), TET2 (46)	at EOT: SF3B1 (47), BRCC3 (2), TET2 (48)
Patient 3	A	MDS-MLD	LTB	good	low	ASXL1 (43), ETV6 (25), GATA2 (46), SETBP1 (3, 2)	–
Patient 4	B	MDS-MLD	HTB	good	intermediate	ASXL1 (41), SETBP1 (49)	at pEP: ASXL1 (40), SETBP1 (45)
Patient 5	B	MDS-SLD-RS	HTB	good	low	–	–
Patient 6	B	MDS-MLD	HTB	good	intermediate	ASXL1 (2)	–
Patient 7	A	MDS-SLD-RS	HTB	good	low	SF3B1 (44), CBL (34)	at pEP: SF3B1 (40), CBL (33)
Patient 8	A	MDS-SLD	HTB	good	intermediate	–	at pEP: ASXL1 (3), ETNK1, U2AF1 (3)
Patient 9	A	MDS-MLD-RS	LTB	good	low	DNMT3A (36), SF3B1 (36), TET2 (32)	at EOT: DNMT3A (31), SF3B1 (32), TET2 (25)

Supplementary Table 4. Baseline characteristics of patients in the LUCAS trial.

	Cohort[a]	Central morphology (WHO 2016) at screening	Transfusion dependency before inclusion[c]	Karyotype category (IPSS-R) at inclusion	IPSS-R risk category at inclusion	Mutated genes at screening (VAF/%, if available)	Mutations at other time point (VAF/% if available)
Patient 10	B	MDS/MPN-RS-T	LTB	good	intermediate	JAK2, SF3B1 (45)	–
Patient 11	A	MDS/MPNu[b]	HTB	very good	very low	SF3B1 (44), JAK2 (3), RAD21 (8)	–
Patient 12	B	MDS-MLD[b]	NTD	good	low	U2AF1 (2), SMC3	–
Patient 13	A	MDS-MLD	HTB	good	low	no mutations within documented genes	–
Patient 14	A	MDS-SLD-RS	LTB	good	low	ANKRD26 (51), DNMT3A (38), SF3B1 (38), TET2 (38)	–
Patient 15	A	MDS-MLD-RS	NTD	good	low	JAK2, SF3B1 (3)	–
Patient 16	A	MDS-MLD-RS	HTB	intermediate	intermediate	–	–
Patient 17	B	MDS-MLD	HTB	–	–	ASXL1 (1)	–
Patient 18	A	MDS/MPN-RS-T	HTB	good	low	–	–
Patient 19	B	MDS-MLD	LTB	good	intermediate	no mutations within documented genes	–

Supplementary Table 4. Baseline characteristics of patients in the LUCAS trial.

	Cohort[a]	Central morphology (WHO 2016) at screening	Transfusion dependency before inclusion[c]	Karyotype category (IPSS-R) at inclusion	IPSS-R risk category at inclusion	Mutated genes at screening (VAF/%, if available)	Mutations at other time point (VAF/% if available)
Patient 20	A	CMML – Type I	NTD	–	–	–	–
Patient 21	A	CMML – Type I	HTB	–	–	SF3B1 (43)	–
Patient 22	A	MDS-SLD-RS	LTB	good	very low	ETNK1 (3), GATA2 (3), SF3B1 (38)	at pEP: ETNK1 (4), GATA2 (4), SF3B1 (24)
Patient 23	A	MDS-SLD	HTB	good	intermediate	–	–
Patient 24	A	MDS-MLD	LTB	–	–	ASXL1 (37), EZH2 (29), JAK2, TET2 (38), U2AF1 (40)	–
Patient 25	A	MDS-SLD-RS	HTB	good	low	SRSF2 (47), TET2 (38)	–
Patient 26	A	MDS-MLD	HTB	very good	low	TET2 (3)	–
Patient 27	A	MDS-MLD	HTB	intermediate	intermediate	DNMT3A (35), GNB1 (25), SF3B1 (36), SRSF2 (30)	at EOT: DNMT3A (28), GNB1 (24), SF3B1 (31), SRSF2 (27), STAT5B (1)
Patient 28	B	MDS-SLD[b]	HTB	very good	intermediate	MPL, TET2 (3), ZRSR2 (91)	–

Supplementary Table 4. Baseline characteristics of patients in the LUCAS trial.

	Cohort[a]	Central morphology (WHO 2016) at screening	Transfusion dependency before inclusion[c]	Karyotype category (IPSS-R) at inclusion	IPSS-R risk category at inclusion	Mutated genes at screening (VAF/%, if available)	Mutations at other time point (VAF/% if available)
Patient 29	A	MDS-SLD[b]	HTB	intermediate	low	CBL (2), EZH2 (5), GNAS (35), PHF6 (12), SF3B1 (32)	–
Patient 30	B	MDS-EB1	HTB	good	intermediate	ASXL1 (27), NRAS (28), U2AF1 (28)	–
Patient 31	B	CMML – Type II	LTB	–	–	TET2 (42), ZRSR2 (87)	at pEP: TET2 (38), ZRSR2 (86)
Patient 32	A	MDS-SLD-RS	HTB	good	low	SF3B1 (30), TET2 (8), PHF6	at pEP: SF3B1 (25), TET2 (9, 1), PHF6 (4), KMT2A-PTD (Ratio 0.336)

[a]Cohort A included ESA exposed (and refractory or intolerant) patients, Cohort B included patients who were ESA naive and had a serum erythropoietin level > 200 U/L. [b]Diagnosis not from central morphology. [c]HTB was defined as requiring ≥ 8 RBC transfusions within the prior 16 weeks; LTB was defined as requiring 3 to 7 RBC transfusions within the prior 16 weeks in at least two transfusion episodes, and NTD was defined as requiring < 3 RBC transfusions and mean hemoglobin level <10 g/dl within the prior 16 weeks. CMML, chronic myelomonocytic leukemia; CPPS, CMML prognostic scoring system; HTB, high transfusion burden; IPSS-R, Revised International Prognostic Scoring System; LTB, low transfusion burden; MDS, myelodysplastic syndromes; MDS-MLD, MDS with multilineage dysplasia; MDS-MLD-RS, MDS-MLD with ring sideroblasts; MDS-SLD, MDS with single-lineage dysplasia; MDS-SLD-RS, MDS-SLD with ring sideroblasts; MDS/MPN, myelodysplastic/myeloproliferative neoplasm; MDS/MPN-RS-T; MDS/MPN with ring sideroblasts and thrombocytosis; NTD, non-transfusion dependent; VAF, variant allele frequency; WHO, World Health Organization.

Supplementary Table 5. Concomitant medication in the CANFIRE and LUCAS trials. All n = 1 unless otherwise stated.

MedDRA-PT	CANFIRE (N = 11)	LUCAS Cohort A ESA-exposed (N = 27)	LUCAS Cohort B ESA-naïve (N = 9)
Anticoagulant therapy		Aspirin	
Autoimmune thyroiditis			L-Thyroxin
Benign prostatic hyperplasia		Tamsulosin (n = 2)	
Blood bilirubin increased	Ursodeoxycholic acid		
Blood cholesterol increased		Ezetimib	
Cardiac failure		Indapamid	
Constipation		Movicol	
Coronary artery disease		Acetylsalicylic acid	
Diabetes mellitus			Metformin, empagliflozin
Folate deficiency		Folic acid	
Gastritis		Pantoprazol	
Generalised oedema		Torasemid	
Glaucoma		Xalocom	
Headache	Acetylsalicylic acid		
Hypercholesterolaemia		Fluvastatin, Simvastatin	Atorvastatin
Hypertension	Dapagliflozin	Candesartan (n = 2), Metoprolol (n = 3), Captopril, Bisoprolol	Bisoprolol, Losartan

Supplementary Table 5. Concomitant medication in the CANFIRE and LUCAS trials. All n = 1 unless otherwise stated.

MedDRA-PT	CANFIRE (N = 11)	LUCAS	
		Cohort A ESA-exposed (N = 27)	Cohort B ESA-naïve (N = 9)
Hyperthyroidism			L-Thyroxin Jod 100µg/150µg
Hyperuricaemia	Allopurinol (n = 2)	Allopurinol (n = 2)	
Hypokalaemia			Kalinor retard P
Hypothyroidism		L-Thyroxin (n = 3), Euthyrox, Jodid	
Influenza			Azithromycin, Acetylcysteine, Xylomelazolin, Paracetamol
Intraocular pressure increased		Dorzo comp 20+5	
Iron overload	Desferral, Exjade (n = 2)	Ferriprox, Deferasirox (n = 2), Deferipron, Exjade (n =2)	Exjade
Muscle spasms		Magnesium (n = 2)	
Myocardial infarction		ASS	
Nasopharyngitis	Cefuroxim, acc		
Neutropenia		Neulasta, Ratiograstim, levofloxacin (n =2), granulocyte colony stimulation factor (n = 2)	
Osteoporosis	Alendronat	Aledron	

Supplementary Table 5. Concomitant medication in the CANFIRE and LUCAS trials. All n = 1 unless otherwise stated.

MedDRA-PT	CANFIRE (N = 11)	LUCAS	
		**Cohort A ESA-exposed (N = 27)**	**Cohort B ESA-naïve (N = 9)**
Osteoporosis prophylaxis		Vitamin D	
Overchelation		Exjade	
Pain		Novalgin, Ibuprofen	
Platelet count increased			Eliquis
Prophylaxis		Eliquis, Clopidogrel, Torasemid, Exjade, atorvastatin, Vitamin D	
Prophylaxis against gastrointestinal ulcer	Pantoprazol	Pantoprazole (n = 2)	
Pyrexia	Sultamicillin		
Rheumatic disorder		Prednisolon	
Rheumatoid arthritis		Rituximab	
Skin infection	Amoxicillin, Clavulansäure		
Supplementation therapy		Magnesium, Kupferglukonat, Vitamin ADEK Kps	
Thyroid cystectomy	Thyronajod		

Supplementary Table 5. Concomitant medication in the CANFIRE and LUCAS trials. All n = 1 unless otherwise stated.

MedDRA-PT	CANFIRE (N = 11)	LUCAS	
		Cohort A ESA-exposed (N = 27)	Cohort B ESA-naïve (N = 9)
Thyroid operation		Levothyroxine Sodium, Euthyrox	
Tumour pain		Novalgin	
Type 2 diabetes mellitus		Metformin	Metformin
Vitamin C deficiency		Dekristol	

CONSORT 2025 checklist

CONSORT 2025 checklist of information to include when reporting a randomised trial*

Section / Topic	No	CONSORT 2025 checklist item description	Reported on page no.
**Title and abstract**			
Title and structured abstract	1a	Identification as a randomised trial	N/A (open label trial)
	1b	Structured summary of the trial design, methods, results, and conclusions	3
**Open science**			
Trial registration	2	Name of trial registry, identifying number (with URL) and date of registration	3, 6–7
Protocol and statistical analysis plan	3	Where the trial protocol and statistical analysis plan can be accessed	4
Data sharing	4	Where and how the individual de-identified participant data (including data dictionary), statistical code and any other materials can be accessed	23
Funding and conflicts of interest	5a	Sources of funding and other support (e.g., supply of drugs), and role of funders in the design, conduct, analysis and reporting of the trial	24
	5b	Financial and other conflicts of interest of the manuscript authors	23–24
**Introduction**			
Background and rationale	6	Scientific background and rationale	4–5
Objectives	7	Specific objectives related to benefits and harms	6
**Methods**			
Patient and public involvement	8	Details of patient or public involvement in the design, conduct and reporting of the trial	N/A
Trial design	9	Description of trial design including type of trial (e.g., parallel group, crossover), allocation ratio, and framework (e.g., superiority, equivalence, non-inferiority, exploratory)	6–7
Changes to trial protocol	10	Important changes to the trial after it commenced including any outcomes or analyses that were not prespecified, with reason	8
Trial setting	11	Settings (e.g., community, hospital) and locations (e.g., countries, sites) where the trial was conducted	Suppl. Information
Eligibility criteria	12a	Eligibility criteria for participants	7–8
	12b	If applicable, eligibility criteria for sites and for individuals delivering the interventions (e.g., surgeons, physiotherapists)	N/A

Intervention and comparator	13	Intervention and comparator with sufficient details to allow replication. If relevant, where additional materials describing the intervention and comparator (e.g., intervention manual) can be accessed	8–9
Outcomes	14	Pre-specified primary and secondary outcomes, including the specific measurement variable (e.g., systolic blood pressure), analysis metric (e.g., change from baseline, final value, time to event), method of aggregation (e.g., median, proportion), and time point for each outcome	9–10
Harms	15	How harms were defined and assessed (e.g., systematically, non-systematically)	9
Sample size	16a	How sample size was determined, including all assumptions supporting the sample size calculation	10–11
	16b	Explanation of any interim analyses and stopping guidelines	11
Randomisation:			
Sequence generation	17a	Who generated the random allocation sequence and the method used	N/A
	17b	Type of randomisation and details of any restriction (e.g., stratification, blocking and block size)	N/A
Allocation concealment mechanism	18	Mechanism used to implement the random allocation sequence (e.g., central computer/telephone; sequentially numbered, opaque, sealed containers), describing any steps to conceal the sequence until interventions were assigned	N/A
Implementation	19	Whether the personnel who enrolled and those who assigned participants to the interventions had access to the random allocation sequence	N/A
Blinding	20a	Who was blinded after assignment to interventions (e.g., participants, care providers, outcome assessors, data analysts)	N/A
	20b	If blinded, how blinding was achieved and description of the similarity of interventions	N/A
Statistical methods	21a	Statistical methods used to compare groups for primary and secondary outcomes, including harms	10–11
	21b	Definition of who is included in each analysis (e.g., all randomised participants), and in which group	10–11
	21c	How missing data were handled in the analysis	11
	21d	Methods for any additional analyses (e.g., subgroup and sensitivity analyses), distinguishing prespecified from post-hoc	9–10
**Results**			
Participant flow, including flow diagram	22a	For each group, the numbers of participants who were randomly assigned, received intended intervention, and were analysed for the primary outcome	11, 15, 28 (Fig.2)
	22b	For each group, losses and exclusions after randomisation, together with reasons	28, Fig.2
Recruitment	23a	Dates defining the periods of recruitment and follow-up for outcomes of benefits and harms	11, 15
	23b	If relevant, why the trial ended or was stopped	11, 15

Intervention and comparator delivery	24a	Intervention and comparator as they were actually administered (e.g., where appropriate, who delivered the intervention/comparator, how participants adhered, whether they were delivered as intended [fidelity])	8–9, 11–12, 15, Fig. 2
	24b	Concomitant care received during the trial for each group	Supp. Table 5
Baseline data	25	A table showing baseline demographic and clinical characteristics for each group	Table 1, Supp. Table 1
Numbers analysed, outcomes and estimation	26	For each primary and secondary outcome, by group:<br>● the number of participants included in the analysis<br>● the number of participants with available data at the outcome time point<br>● result for each group, and the estimated effect size and its precision (such as 95% confidence interval)<br>● for binary outcomes, presentation of both absolute and relative effect size	14–15, Figs. 3–4
Harms	27	All harms or unintended events in each group	14, 16–17
Ancillary analyses	28	Any other analyses performed, including subgroup and sensitivity analyses, distinguishing pre-specified from post-hoc	14, 16, Figs.5–6
**Discussion**			
Interpretation	29	Interpretation consistent with results, balancing benefits and harms, and considering other relevant evidence	17–23
Limitations	30	Trial limitations, addressing sources of potential bias, imprecision, generalisability, and, if relevant, multiplicity of analyses	21–23

*We strongly recommend reading this statement in conjunction with the CONSORT 2025 Explanation and Elaboration and/or the CONSORT 2025 Expanded Checklist for important clarifications on all the items. We also recommend reading relevant CONSORT extensions. See www.consort-spirit.org.

Citation: Hopewell S, Chan AW, Collins GS, Hróbjartsson A, Moher D, Schulz KF, et al. CONSORT 2025 Statement: updated guideline for reporting randomised trials. BMJ. 2025; 388:e081123. https://dx.doi.org/10.1136/bmj-2024-081123.

© 2025 Hopewell et al. This is an Open Access article distributed under the terms of the Creative Commons Attribution License (https://creativecommons.org/licenses/by/4.0/), which permits unrestricted use, distribution, and reproduction in any medium, provided the original work is properly cited.

Study locations

LUCAS study locations

Investigator	Study Centre(s)
Prof. Dr. Uwe Platzbecker	Universitätsklinikum Leipzig<br>Klinik und Poliklinik für Hämatologie, Zelltherapie, Hämostaseologie und Infektiologie<br>Liebigstraße 22, Haus 7, 04103 Leipzig
Prof. Dr. Katharina Götze	Klinikum rechts der Isar der TU München<br>III. Medizinische Klinik - Hämatologie und Onkologie<br>Ismaninger Straße 22<br>81675 München
Dr. Stefan Mahlmann	Friedrich-Ebert-Krankenhaus GmbH<br>Klinik für Hämatologie, Onkologie und Nephrologie<br>Friesenstrasse 44<br>24534 Neumünster
Dr. Kathrin Rieger	Charité Berlin - Campus Benjamin Franklin<br>Med. Klinik m. S. Hämatologie, Onkologie, Tumorimmunologie<br>Hindenburgdamm 30<br>12200 Berlin<br><br>Charité Berlin Campus Virchow Klinikum Med. Klinik m. S. Hämatologie, Onkologie und Tumorimmunologie

Investigator	Study Centre(s)
	Augustenburger Platz 1<br>13353 Berlin
PD Dr. Martin Schmidt-Hieber	Carl-Thiem-Klinikum Cottbus gGmbH<br>2. Med. Klinik<br>Thiemstraße 111<br>03048 Cottbus
PD Dr. Thomas Illmer	Gemeinschaftspraxis Dr. Jacobasch<br>Dresden<br>Arnoldstraße 18<br>01307 Dresden
Prof. Dr. Aristoteles Giagounidis	Marienhospital Düsseldorf<br>Klinik für Onkologie und Hämatologie,<br>Palliativmedizin<br>Rochusstraße 2<br>40479 Düsseldorf
Dr. Friederike Wortmann	Universitätsklinikum Schleswig-<br>Holstein<br>Klinik für Hämatologie und Onkologie<br>Campus Lübeck<br>Ratzeburger Allee 160<br>23538 Lübeck
Prof. Dr. Rudolf Weide	InVO-Institut für Versorgungsforschung in<br>der Onkologie

Investigator	Study Centre(s)
	Neversstraße 5<br>56068 Koblenz
Dr. Daniel Sasca	Universitätsmedizin der Johannes<br>Gutenberg-Universität Mainz<br>III. Medizinische Klinik und Poliklinik -<br>Hämatologie, Internistische Onkologie und<br>Pneumologie<br>Langenbeckstraße 1<br>55131 Mainz
PD Dr. Mohamad Jawhar	Universitätsklinikum Mannheim<br>III. Medizinische Klinik - Hämatologie und<br>Onkologie<br>Theodor-Kutzer-Ufer 1-3<br>68167 Mannheim
Dr. Martina Haibach	ONCOSEARCH<br>Institut für Klin. Studien GbR<br>Nägselbachstr. 49 c<br>91052 Erlangen
Dr. Ursula Vehling-Kaiser	VK & K Studien GbR<br>Studienzentrum<br>Achdorfer Weg 5<br>84036 Landshut

Investigator	Study Centre(s)
Dr. Mohammad Amen Wattad	Klinikum Hochsauerland GmbH<br>Klinik f. Hämatologie, Onkologie,<br>Palliativmedizin, Stammzelltransplantation<br>Schederweg 12<br>59872 Meschede
Dr. Julia Glück-Wolf	Rems-Murr-Kliniken gGmbH<br>Hämatologie, Onkologie und Palliativmedizin<br>Am Jakobsweg 1<br>71364 Winnenden

CANFIRE study locations

Investigator	Study Centre(s)
Active (date: 07/2026)	
Dr. Dominic Brauer	Universitätsklinikum Leipzig<br>Klinik und Poliklinik für Hämatologie, Zelltherapie, Hämostaseologie und Infektiologie<br>Liebigstraße 22, Haus 7, 04103 Leipzig
Dr. Philipp Kiewe	Medizinisches Versorgungszentrum<br>"Onkologischer Schwerpunkt am Oskar-Helene-Heim"<br>Clayallee 225 a<br>14195 Berlin
closed	
Prof. Dr. Katharina Götze	Klinikum rechts der Isar der TU München<br>III. Medizinische Klinik - Hämatologie und Onkologie<br>Ismaninger Straße 22<br>81675 München
PD Dr. Alexander Höllein	Rotkreuzklinikum München<br>III. Medizinische Abteilung<br>Nymphenburger Str. 163<br>80634 München
Dr. Katja Sockel	Universitätsklinikum Carl Gustav Carus an der Technischen Universität Dresden

Investigator	Study Centre(s)
	Medizinischen Klinik und Poliklinik I / Hämatologie Fetscherstraße 74 01307 Dresden
Dr. Karin Meyer	Universitätsklinikum Bonn Med. Klinik III / ZIM, Hämatologie/Onkologie Venusberg - Campus 1 53127 Bonn

Trial protocols and statistical analysis plans

Trial Protocol
A Phase II, Single-Arm, Open-Label Study to Assess the Efficacy and Safety of Canakinumab for the Treatment of Anemia in Patients with IPSS-R Very Low, Low, or Intermediate Risk Myelodysplastic Syndromes or MDS/MPN

CANFIRE Trial

Coordinating investigator
Dr. Anne Sophie Kubasch
Klinik und Poliklinik für Hämatologie,
Zelltherapie, Hämostaseologie und Infektiologie
Universität Leipzig, Medizinische Fakultät
Liebigstraße 22, 04103 Leipzig, Germany

Biometry
Dr. Jens Przybilla
Universität Leipzig, Medizinische Fakultät
Härtelstraße 16-18, 04107 Leipzig, Germany

Sponsor
Universität Leipzig
Ritterstr. 26, 04109 Leipzig, Germany

Date: 26.02.2024
Version: Final 4.0
EudraCT-No.: 2020-002246-16

The information provided in this document is confidential and is not to be circulated to third parties.

Table of Contents

PAGE

GENERAL INFORMATION 4
Responsible Parties 4
Synopsis 6
Synopse (German version) 9
Schedule of Assessments and Procedures 12
Flow Chart 14

1 RATIONALE 15
1.1 Medical Background 15
1.2 Rationale 16
1.3 Risk-Benefit Consideration 17

2 OBJECTIVES 18
2.1 Primary Objective 18
2.2 Secondary Objectives 18

3 TRIAL DESIGN AND DESCRIPTION 19
3.1 Trial Design 19
3.2 Requirements at the Trial Sites regarding Personnel and Equipment 19
3.3 Trial Sites and Number of Trial Subjects 20
3.4 Expected Duration of Trial 20
3.5 Premature Termination of the Trial 21

4 TRIAL SUBJECTS 22
4.2 Justification for the Inclusion of vulnerable populations 26
4.3 Participation in more than one Clinical Trial 26
4.4 Statement on the Inclusion of Dependent Individuals 26
4.5 Rationale for Gender Distribution 26

5 INVESTIGATIONAL PRODUCT 27
5.1 Trial Drugs 27
5.2 Packaging and Labelling of the Trial Drug 27
5.3 Storage and Handling Requirements 28
5.4 Drug Accountability 28
5.5 Administration of Trial Drug 29

6 INDIVIDUAL TRIAL PROCEDURES 34
6.1 Patient Information and Informed Consent 34
6.2 Enrolment in the Trial 34
6.3 Description of Assessments and Procedures 36
6.4 Assessments during follow-up (FU) 39
6.5 Premature Termination of the Therapy or Follow-up 39
6.6 Plan for Further Treatment 41

7 ADVERSE EVENTS (AE/SAE) 42
7.1 Adverse Events (AE) 42
7.2 Safety Analysis 44
7.3 Concomitant Diseases 44
7.4 Serious Adverse Events/Serious Adverse Reactions (SAE/SAR) 44
7.5 Periodic Reports 45
7.6 Suspected Unexpected Serious Adverse Reactions (SUSAR) 46
7.7 Other Safety Relevant Issues 46
7.8 Therapeutic Procedures 47
7.9 Dealing with Pregnancy 47

8 BIOMETRY ... 48
8.1 Biometrical Aspects of the Trial Design ... 48
8.2 End Points ... 48
8.3 Statistical Description of the trial hypothesis ... 49
8.4 Sample Size Discussion ... 49
8.5 Statistical Methods ... 49
8.6 Statistical Monitoring ... 50
8.7 Interim Analysis ... 50
8.8 Final Analysis ... 50
8.9 Modification of the revised IWG 2018 hematological response criteria ... 51

9 CONCOMITANT SCIENTIFIC PROJECTS ... 54

10 ETHICAL, LEGAL AND ADMINISTRATIVE ASPECTS ... 55
10.1 GCP-Statement ... 55
10.2 Initial Submission ... 55
10.3 Protocol Amendments ... 55

11 DOCUMENTATION ... 56
11.1 General information and Access Rights ... 56
11.2 Patient File and Source Data ... 56
11.3 Data Management ... 57
11.4 Archiving ... 57

12 REFERENCE EVALUATIONS ... 58
12.1 Central morphology evaluation in Düsseldorf ... 58
12.2 Instructions for bone marrow / peripheral blood samples ... 58

13 SUPERVISION OF THE CLINICAL TRIAL ... 59
13.1 Access to Source Data ... 59
13.2 Monitoring ... 59
13.3 Audits / inspections ... 59
13.4 Data protection and Confidentiality ... 60
13.5 Declaration regarding Data Protection ... 60
13.6 Declaration regarding the Pseudonymized Transfer of Personal Data ... 61
13.7 Anonymisation of Data after the end of Archiving ... 61

14 ADMINISTRATIVE AGREEMENTS ... 61
14.1 Adherence to the Protocol ... 61
14.2 Funding and Insurance ... 61
14.3 Notification of Local Authorities ... 62
14.4 Publication Policy and Registration ... 62
14.5 Data Sharing Statement ... 62

15 PROTOCOL SIGNATURES ... 64

16 PROTOCOL AGREEMENT ... 65

17 APPENDIX ... 66
17.1 Classification of Adverse Events ... 66
17.2 Abbreviations ... 68
17.3 Labelling ... 70
17.4 Template trial protocol ... 70
17.5 Supplemental Materials ... 71

18 REFERENCES ... 74

GENERAL INFORMATION[1]

Responsible Parties

Sponsor	Universität Leipzig<br>Ritterstr. 26, 04109 Leipzig, Germany<br><br>Authorised representative of the sponsor:<br>Dr. Anne Sophie Kubasch
Coordinating investigator	**Dr. Anne Sophie Kubasch**<br>Klinik und Poliklinik für Hämatologie, Zelltherapie, Hämostaseologie und Infektiologie<br>Universität Leipzig - Medizinische Fakultät<br>Liebigstraße 22, 04103 Leipzig<br>Phone: +49 341 97-20257<br>Fax: +49 341 97-13059<br>E-Mail: annesophie.kubasch@medizin.uni-leipzig.de
Deputy of Coordinating investigator	**Prof. Dr. Uwe Platzbecker**<br>Klinik und Poliklinik für Hämatologie, Zelltherapie, Hämostaseologie und Infektiologie<br>Universität Leipzig - Medizinische Fakultät<br>Liebigstraße 22, 04103 Leipzig<br>Phone: +49 341 97-13050<br>Fax: +49 341 97-13059<br>E-Mail: uwe.platzbecker@medizin.uni-leipzig.de
Project Management	**Dr. Susanne Melzer**<br>Zentrum für Klinische Studien Leipzig<br>Universität Leipzig - Medizinische Fakultät<br>Härtelstr.16-18, 04107 Leipzig<br>Phone: +49 341 97 16317<br>Fax: +49 341 97 16259<br>E-Mail: susanne.melzer@zks.uni-leipzig.de
Pharmacovigilance	**Madlen Dörschmann**<br>Zentrum für Klinische Studien Leipzig<br>Universität Leipzig - Medizinische Fakultät<br>Härtelstr.16-18, 04107 Leipzig<br>Phone: +49 341 97 16129<br>Fax: +49 341 97 16278<br>E-Mail: pharmacovigilance@zks.uni-leipzig.de
Database	**Berit Kalina**<br>Zentrum für Klinische Studien Leipzig<br>Universität Leipzig - Medizinische Fakultät<br>Härtelstr.16-18, 04107 Leipzig<br>Phone: +49 341 97 16275<br>Fax: +49 341 97 16259<br>E-Mail: berit.kalina@zks.uni-leipzig.de

[1] The contents of this trial protocol refer to women and men equally. However, for reasons of better readability, the male form is chosen for all personal designations. The female form is always taken into account. An exception are those contents solely referring to women.

Data Management	**Anke Stolle**<br>Zentrum für Klinische Studien Leipzig<br>Universität Leipzig - Medizinische Fakultät<br>Härtelstr.16-18, 04107 Leipzig<br>Phone: +49 341 97 16268<br>Fax: +49 341 97 16259<br>E-Mail: anke.stolle@zks.uni-leipzig.de
Biometry	**Dr. Jens Przybilla**<br>Zentrum für Klinische Studien Leipzig<br>Universität Leipzig - Medizinische Fakultät<br>Härtelstr.16-18, 04107 Leipzig<br>Phone: +49 341 97 15770<br>Fax: +49 341 97 16259<br>E-Mail: jens.pryzbilla@zks.uni-leipzig.de
Monitoring	**Angelika Siegmund**<br>Zentrum für Klinische Studien Leipzig<br>Universität Leipzig - Medizinische Fakultät<br>Härtelstr.16-18, 04107 Leipzig<br>Phone +49 341 97 16294<br>Fax: +49 341 97 16219<br>E-Mail: angelika.siegmund@zks.uni-leipzig.de
Central Morphology	**Central Morphology Lab,**<br>**Sabine Haase**<br>Marien Hospital Düsseldorf<br>Klinik für Onkologie und Hämatologie, Palliativmedizin<br>Hämatologisches Speziallabor<br>Rochusstraße 2, 40479 Düsseldorf<br>Phone: +49 2 11 44 0068 79<br>Fax: +49 (02 11) 44 0024 27<br>E-Mail: sabine.haase@vkkd-kliniken.de
Funding	**Novartis Pharma AG**<br>Emma Chen Sasse, MD. MSc<br>HeadWorldwide Senior Medical Director Hematology<br>Phone: +41 79 438 7990<br>E-Mail: emma.sasse@novartis.com
Concomitant scientific project/biobank	**PD Dr. Michael Cross**<br>**Dr. Marie Schneider**<br>José Carreras Forschungslabor<br>Universitätsklinikum Leipzig AöR<br>Klinik und Poliklinik für Hämatologie, Zelltherapie und Hämostaseologie<br>Johannisallee 32A, 04103 Leipzig, Germany<br>Phone: +49 341 97 13137<br>Fax: +49 341 97 13089<br>E-Mail: michael.cross@medizin.uni-leipzig.de<br>E-Mail: marie.schneider@medizin.uni-leipzig.de

Synopsis

Title of the trial	A Phase II, Single-Arm, Open-Label Study to Assess the Efficacy and Safety of Canakinumab for the Treatment of Anemia in Patients with IPSS-R very low, low or Intermediate risk myelodysplastic syndromes (MDS) or MDS/MPN
Acronym	CANFIRE Trial
Indication	Lower-risk MDS or MDS/MPN patients (IPSS-R up to 3.5: very low/low/intermediate) with anemia.
Primary objective of the trial/ primary endpoint	**Primary objective:**<br>To study the erythroid response rate (HI-E) of canakinumab in patients with IPSS-R very low, low, or intermediate risk MDS or MDS/MPN.<br><br>**Primary endpoint:**<br>Erythroid response (HI-E) rate according to IWG 2018 criteria[1] after 6 months of treatment.
Secondary objectives of the trial/secondary endpoints	**Secondary objectives:**<br>- To study the HI-E response duration<br>- To study the safety and toxicity of single agent canakinumab<br>- To study the proportion of disease progression<br>- To assess the impact of canakinumab on quality of life (QoL) over 6 months post screening
Trial design	Phase II<br>Single-Arm<br>Open-Label
Trial population	Lower-risk MDS or MDS/MPN patients (IPSS-R up to 3.5: very low/low/intermediate) with symptomatic anemia, relapsed/refractory/ intolerant/ineligible to standard therapy with ESA<br><br>**Key Inclusion criteria:**<br>• Cytomorphology based confirmed diagnosis of lower-risk MDS or MDS/MPN including MDS/MPN-RS-T, MDS/MPNu, aCML or CMML (as per the World Health Organization [WHO] 2016 classification)<br>• IPSS-R up to 3.5: very low/low/intermediate risk disease (MDS); MDS/MPN < 10% bone marrow blasts; CPSS-Score low/intermediate for CMML<br>• Symptomatic anemia (based on complete Hb time course and transfusion history):<br>- NTD (non-transfusion dependent): < 3 RBC transfusions and mean hemoglobin level <10 g/dl within the last 16 weeks<br>- LTB (low transfusion burden): 3-7 RBC transfusions within the last 16 weeks<br>- HTB (high transfusion burden): ≥ 8 RBC transfusions within the last 16 weeks<br><br>• Relapsed / refractory / intolerant / ineligible (sEPO ≥ 200 U/L) to ESA treatment<br>• Age ≥ 18 years<br>• Written informed consent

	**Key Exclusion criteria:**<br>• Known clinically significant anemia due to iron, vitamin B12, or folate deficiencies, or autoimmune or hereditary hemolytic anemia, or gastrointestinal bleeding<br>• Prior allogeneic or autologous stem cell transplant<br>• Known history of diagnosis of AML<br>• Severe neutropenia (ANC ≤ 0.5 Gpt/L)<br>• Severe thrombocytopenia (PLT <30 Gpt/L)<br>• Serum creatinine > 1.5x ULN OR measured or calculated creatinine clearance < 40 ml/min<br>• AST or ALT ≥ 3.0 x ULN<br>• ECOG > 2<br>• Total bilirubin ≥ 2.0 x ULN<br>• Active second malignancy<br>• Prior or concomitant treatment with anticancer medications or disease modifying agents<br>• Previously treated with canakinumab
Sample size	n= 50<br>(n = 41 to be analysed – allowing for up to n = 9 included patients who fail to start treatment)<br><br>**Will not be achieved due to premature termination of recruitment.**
Therapy	**200 mg canakinumab administered subcutaneously every three weeks for a duration of 6 months**<br>Dose can be reduced to 150 mg and/or 100 mg if 200 mg are not tolerated.<br>**Responder will be further treated with 200 mg canakinumab for up to a maximum of three years treatment.**<br><u>The treatment will terminated prematurely in case of:</u><br>- grade 3-4 toxicity unresponsive to dose reduction or treatment interruption<br>- disease progression<br>- withdrawal of consent<br>- loss of contact<br>- major protocol violation such as omitting study procedure(s)<br><u>Treatment phase scheme:</u><br>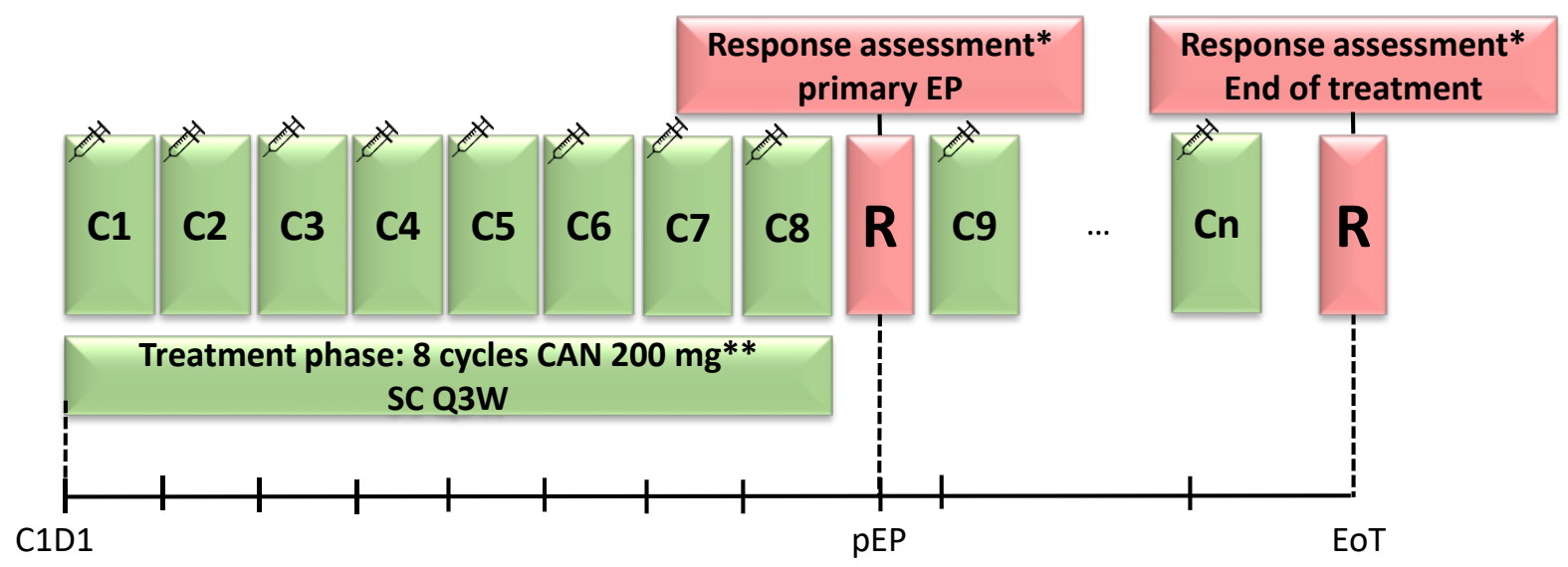<br><br>*according to IWG 2018<br>**intended CAN dose: 200 mg; can be reduced down to 150 mg and, if required, to 100 mg
Biometry	**The trial aimed to perform the following statistical analyses:**<br><br><u>Efficacy</u>: Concerning clinical efficacy, the trial is designed to test the null hypothesis that the HI-E response probability is ≤ 5% against the planning alternative hypothesis that the HI-E response probability is ≥20%. Using a one-sided significance level of 2.5% and

	requiring a power of 85% leads to a sample size of N=41 with the exact binomial test.<br><br>Description of the primary efficacy analysis and population: The analysis follows the intent to treat principle. Only documented HI-E responses count as success; the denominator is the number of included patients. The exact binomial test will be used. The HI-E response rate will be estimated with a two-sided 95% confidence interval.<br><br>Safety: Safety data will be evaluated descriptively.
Trial Duration	**Patient related:**<br>Treatment: 6 months up to three years<br>Follow-up: 2 months after end of treatment (one follow-up visit)<br>Total duration for individual patient: 12 months (non-responder), up to loss of response<br><br>**Trial related:**<br>Recruitment period: 23 months<br>First patient first visit to last patient last visit: + 2 months after last patient has lost response<br>Duration of the entire trial: cannot be estimated, as last patient will be treated as long as loss of response occurs

Synopse (German version)

Titel der Studie	Eine einarmige, offene Phase-II-Studie zur Bewertung der Wirksamkeit und Sicherheit von Canakinumab für die Behandlung von Anämie bei Patienten mit IPSS-R sehr niedrigem, niedrigem oder intermediärem Risiko myelodysplastischem Syndrom (MDS) oder MDS/MPN
Kurzbezeichnung der Studie	CANFIRE Studie
Indikation	MDS oder MDS/MPN-Patienten mit niedrigem Risiko (IPSS-R bis zu 3,5: sehr niedrig/niedrig/intermediär) mit symptomatischer Anämie
Primäres Ziel der Studie/ primärer Endpunkt	**Primäres Ziel:**<br>Untersuchung der erythroiden hämatologischen Ansprechrate (HI-E) von Canakinumab bei Patienten mit IPSS-R sehr niedrigem, niedrigem oder mittlerem MDS-Risiko<br><br>**Primärer Endpunkt:**<br>Erythroide hämatologische Ansprechrate (HI-E) nach den Kriterien der IWG 2018[1] nach 6 Monaten Behandlung.
Sekundäre Ziele der Studie/ sekundäre Endpunkte	**Sekundäre Ziele:**<br>- Untersuchung der HI-E Responsedauer<br>- Untersuchung der Sicherheit und Toxizität von Canakinumab als Einzelwirkstoff<br>- Untersuchung des Anteils der Krankheitsprogression<br>- Beurteilung des Einflusses von Canakinumab auf die Lebensqualität (QoL) über 6 Monate nach dem Screening
Studiendesign	Phase II<br>1-Arm<br>Offenes Design
Studienpopulation	MDS- oder MDS/MPN-Patienten mit niedrigem Risiko (IPSS-R bis zu 3,5: sehr niedrig/niedrig/intermediär) mit symptomatischer Anämie, rezidiviert/refraktär/unverträglich/nicht geeignet für die Standardtherapie mit ESA<br><br>**Haupteinschlusskriterien:**<br>- Zytomorphologie-basierte, bestätigte Diagnose von MDS oder MDS/MPN mit geringerem Risiko, einschließlich MDS/MPN-RS-T, MDS/MPNu, aCML oder CMML (gemäß Klassifikation der Weltgesundheitsorganisation (WHO) 2016)<br>- IPSS-R bis zu 3,5: sehr niedriges/niedriges/mittleres Risiko (MDS); MDS/MPN < 10% Knochenmarkblasten; CPSS-Score niedrig/mittleres Risiko für CMML<br>- Symptomatische Anämie (basierend auf vollständigem Hb-Zeitverlauf und Transfusionsanamnese):<br>- NTD (nicht transfusionsabhängig): < 3 RBC-Transfusionen und mittlerer Hämoglobinwert <10 g/dl innerhalb der letzten 16 Wochen<br>- LTB: 3-7 RBC-Transfusionen innerhalb der letzten 16 Wochen<br>- HTB: ≥ 8 RBC-Transfusionen innerhalb der letzten 16 Wochen<br>- Rezidiviert/refraktär/intolerant/nicht geeignet (sEPO ≥ 200 U/L) für eine Therapie mit ESA

	- Alter ≥ 18 Jahre<br>- Schriftliche Einwilligung<br><br>**Hauptausschlusskriterien:**<br>- Bekannte klinisch signifikante Anämie aufgrund von Eisen-, Vitamin B12- oder Folatmangel, autoimmune oder hereditäre hämolytische Anämie oder gastrointestinaler Blutungen<br>- Vorherige allogene oder autologe Stammzellentransplantation<br>- Bereits diagnostizierte AML<br>- Schwere Neutropenie (ANC ≤ 0.5 Gpt/L)<br>- Schwere Thrombozytopenie (PLT <30 Gpt/L)<br>- Serumkreatinin > 1,5x ULN ODER gemessene oder berechnete Kreatinin-Clearance < 40 ml/min<br>- AST oder ALT ≥ 3.0 x ULN<br>- ECOG > 2<br>- Gesamtbilirubin ≥ 2.0 x ULN<br>- Aktives zweites Malignom<br>- Vorhergehende oder begleitende Behandlung mit Krebsmedikamenten oder krankheitsmodifizierenden Substanzen<br>- Vorhergehende Behandlung mit Canakinumab
Patientenzahl	n= 50<br><br>(n = 41 zu analysieren - unter Berücksichtigung von n = 9, die im Verlauf verloren gehen)<br><br>**Wird durch die vorzeitige Beendigung der Rekrutierung nicht erreicht**
Therapie	**200 mg Canakinumab wird einmal alle drei Wochen über einen Zeitraum von 6 Monaten subkutan verabreicht**<br>- Dosis kann auf 150 mg und/oder 100 mg reduziert werden, wenn 200 mg nicht vertragen werden<br>**Responder werden mit 200 mg Canakinumab weiterbehandelt, bis zu maximal 3 Jahren.**<br>Die Behandlung wird vorzeitig beendet:<br>- Grad 3-4 Toxizität, die auf Dosisreduktion nicht anspricht oder Behandlungsunterbrechung<br>- Fortschreiten der Erkrankung<br>- Rücknahme der Einwilligung<br>- Kontaktverlust<br>- Schwerwiegende Protokollverletzungen wie das Unterlassen von Studienverfahren<br>Schema der Behandlungsphase:<br>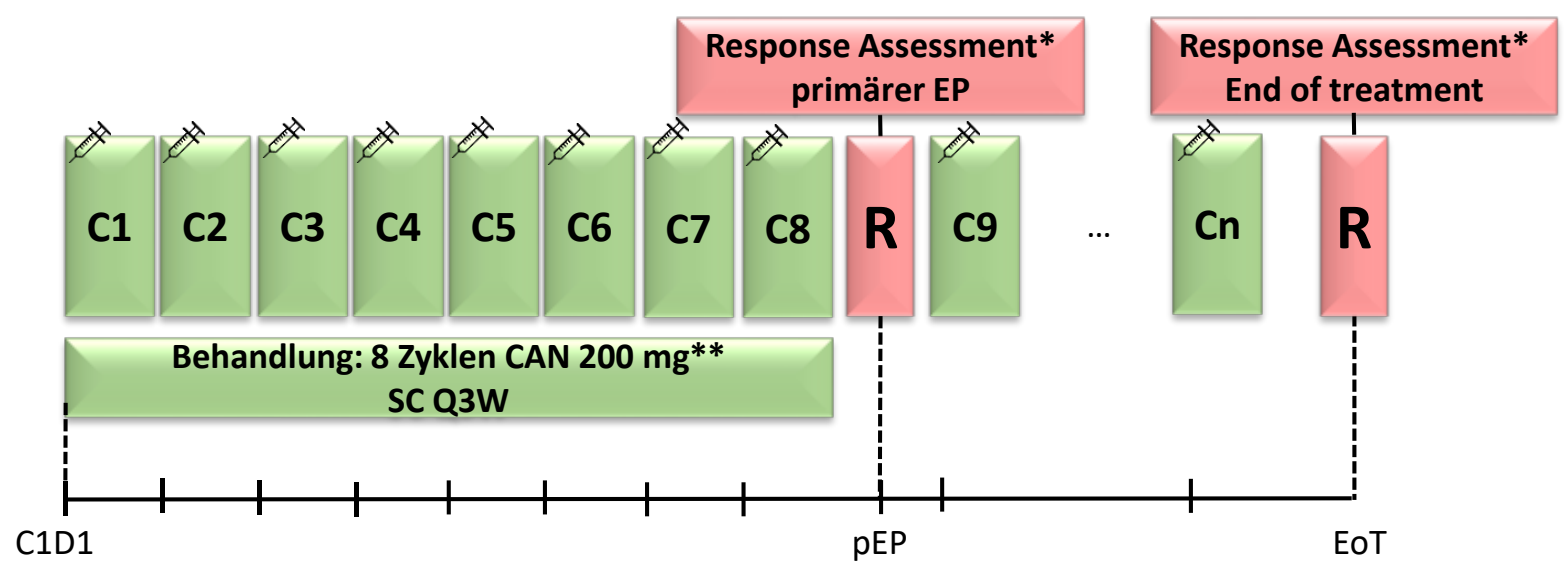<br><br>*gemäß IWG 2018<br>**vorgesehene CAN-Dosis: 200 mg; kann auf 150 mg und, falls erforderlich, auf 100 mg reduziert werden
Biometrie	**Ziel der Studie war die Durchführung der folgenden statistischen Analysen:**

	Wirksamkeit: Hinsichtlich der klinischen Wirksamkeit ist die Studie so angelegt, die Nullhypothese, dass das HI-E-Ansprechen ≤ 5% beträgt, gegen die alternative Planungshypothese, dass die HI-E-Reaktionswahrscheinlichkeit ≥20% beträgt, zu testen. Die Verwendung eines einseitigen Signifikanzniveaus von 2,5% und die Annahme einer Power von 85% führt zu einer Stichprobengröße von N=41 mit dem exakten Binomialtest.<br><br>Beschreibung der primären Wirksamkeitsanalyse und der Population: Die Analyse folgt dem Prinzip der Behandlungsabsicht. Nur dokumentiertes HI-E-Ansprechen zählen als Erfolg; der Nenner ist die Anzahl der eingeschlossenen Patienten. Es wird der exakte binomiale Test verwendet. Die HI-E-Ansprechrate wird mit einem zweiseitigen 95%-Konfidenzintervall geschätzt.<br><br>Sicherheit: Die Sicherheitsdaten werden deskriptiv ausgewertet.
Zeitplan	**Patientenbezogen:**<br>Behandlung: 6 Monate bis zu drei Jahre<br>Nachbeobachtung: 2 Monate nach Behandlungsende (eine Follow-up Visite)<br>Gesamtdauer für individuellen Patienten: 12 Monate (Non-Responder)<br><br>**Studienbezogen:**<br>Rekrutierungszeitraum: 23 Monate<br>Erster Patient erste Visite bis letzter Patient letzte Visite: + 2 Monate nach Verlust des Ansprechens des letzten Patienten<br>Dauer des gesamten Studie: Kann nicht geschätzt werden, da die Studie bis zum Verlust des Ansprechens des letzten Patienten fortgesetzt wird

Schedule of Assessments and Procedures

Trial procedures	Screening	Treatment				Primary endpoint	End of treatment[2]	Follow-up 1/ End of study
		Cycle 1		Cycle 2-n				
	**≤ 28 days** prior to start of treatment	**Day 1 ± 3** of each cycle[3]	**Day 8**	**Day 1 ± 3** of each cycle	**Day 11 ± 4**[4]	**21 ± 3 days** after the eighth IMP dose	**28 ± 3 days** after last IMP dose	**56 ± 14 days** after EoT
**Informed consent**	x							
**In- / exclusion criteria**	x							
**Demographics and MDS-/MDS/MPN-related medical history**	x							
**Concomitant MDS-/MDS/MPN-specific medication**	x	x		x		x	x	x
**Initial disease status including tuberculosis test**	x							
**Transfusion and Hb history (16 weeks preceding first CAN administration)**	x							
**Physical examination (incl. height, weight), vital signs**	x	x		x		x	x	
**ECOG performance status**	x	x		x		x	x	
**Quality of life (QoL)**		x		x		x	x	
**Pregnancy test**	x	x		x			x	
**Canakinumab administration**		x		x				
**Toxicity/AE assessment**		x	x	x	x	x	x	x
**Response assessment**						x	x	
**Disease status (telephone visit)**								x
**Serum chemistry (local)**	x	x	x	x	x	x	x	

[2] For non-responder pEP is equivalent to EoT visit.
[3] One cycle = 21 days ± 3
[4] If treatment is continued after the primary endpoint visit, the day 11 visit can be omitted from the 9th cycle onwards

Trial procedures	Screening	Treatment				Primary endpoint	End of treatment[5]	Follow-up 1/ End of Study
		Cycle 1		Cycle 2-n				
	**≤ 28 days** prior to start of treatment	**Day 1 ± 3** of each cycle[6]	**Day 8**	**Day 1 ± 3** of each cycle	**Day 11 ± 4**[4]	**21 ± 3 days** after the eighth IMP dose	**28 ± 3 days** after last IMP dose	**56 ± 14 days** after EoT
**Automated CBC (local)**	x[7]	x	x	x	x	x	x	
**Cytogenetics (from BM, local)**[8]	x					x	x	
**Molecular genetics (from PB or BM, local)**	x					x	x	
**Central morphology (central, Düsseldorf)**[9]								
**CBC with differential (slides from PB)**	x					x	x	
**Bone marrow cytology assessment (slides from BM)**	x					x	x	
Concomitant research (optional)								
**Bone marrow sampling**	x					x	x	
**Peripheral blood sampling**	x	x		x		x	x	
**Buccal swab**	x							

5 For non-responder pEP is equivalent to EoT visit.

6 One cycle = 21 days ± 3

7 Hb measurement needs to be repeated two weeks after screening measurement, if result from central morphology is still pending

8 BM sampling during treatment period (cyles 1-8) according to local standards. For responding patients, BM sampling is recommended once a year, on the first day of a given cycle when CAN is administered (17 ± 2 cycles after pEP and thereafter 17 ± 2 cycles after the previous yearly visit).

9 Only for responding patients, central morphology (CBC with differential and BM cytology assessment) will be carried out once a year, on the first day of a given cycle when CAN is administered (17 ± 2 cycles after pEP and thereafter 17 ± 2 cycles after the previous yearly visit).

Flow Chart

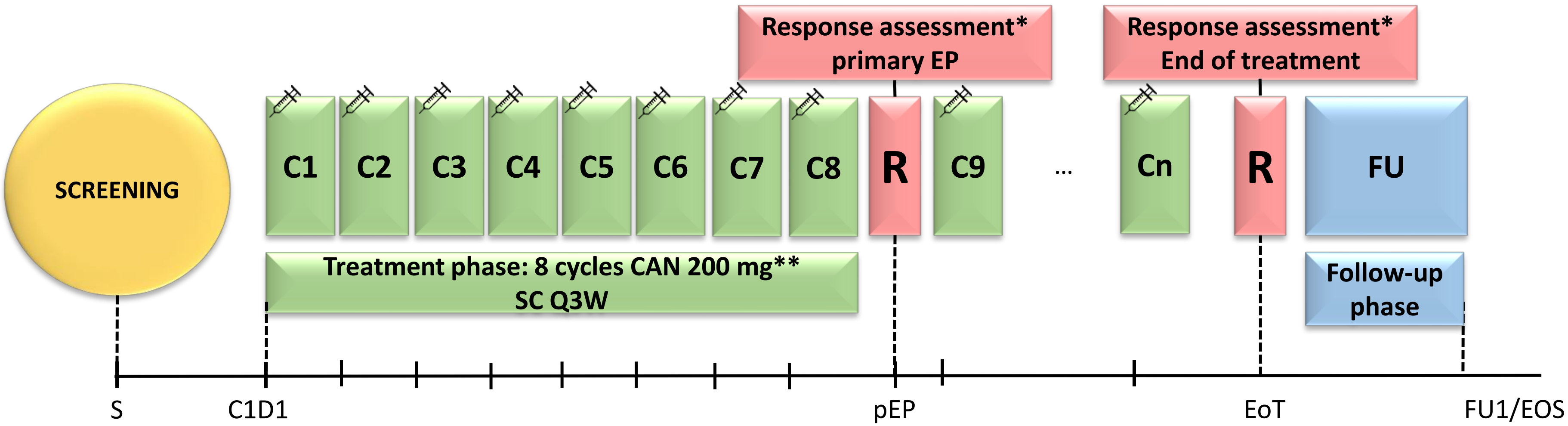

*according to IWG 2018

**intended CAN dose: 200 mg; can be reduced down to 150 mg and, if required, to 100 mg

1 RATIONALE

1.1 Medical Background

Medical need

MDS are clonal hematopoietic stem/progenitor cell (HSPC) disorders characterized by ineffective hematopoiesis, peripheral cytopenias and a risk for transformation to acute myeloid leukemia (AML). Overall, therapeutic options are limited and allogeneic stem cell transplantation (allo-HSCT) remains the only potential curative treatment option. Allo-HSCT, however, is predominately used in patients with higher-risk (HR) MDS and is a burdensome approach associated with numerous complications. Patients with lower-risk (LR) disease, who make up two-thirds of the MDS population, suffer predominately from anemia and are commonly treated with supportive care (i.e. red blood cell transfusions), erythropoiesis-stimulating agents (ESAs) and other growth factors. However, response to ESAs is limited (below 50%) and transfusions are associated with fatigue, impaired quality of life, increased cardiovascular risks and toxic iron overload[2], calling for new treatment approaches in this patient population.

MDS and inflammation

In MDS, hematopoietic stem and progenitor cells are key in terms of disease development. These cells acquire somatic mutations with aging, often resulting in the emergence of (sub)clonal hematopoiesis, which is believed to play a major role in MDS development. In this context, clonal hematopoiesis of indeterminate potential (CHIP) represents a possible precursor to MDS and myeloid cancer. In CHIP, the genes most commonly mutated with increasing age are TET2 and DNMT3A[3]. TET2-mutated macrophages may contribute to a pro-inflammatory environment and patients with a CHIP mutation at a VAF>0.1 have elevated levels of the pro-inflammatory driver cytokine TNFα, suggesting a more aggressive inflammatory milieu[4]. It has recently been suggested that an inflammatory bone marrow can be instrumental in driving the selection of mutant clones. Thus, the establishment of a pro-inflammatory bone marrow microenvironment with aberrant innate immune and cytokine signaling is likely to be a pivotal event in the pathogenesis of hematological malignancies such as MDS[5].
The presence of abnormal levels of cytokines, chemokines and growth factors in the peripheral blood and bone marrow of MDS patients has been extensively documented. In general, levels of TNF-α, IFN-γ, TGF-β, IL-6 and the myeloid growth factors granulocyte colony-stimulating factor (G-CSF) and macrophage colony-stimulating factor (M-CSF) are increased in MDS patients compared to age-matched controls, reflecting a profound dysregulation of both inflammatory signaling and myeloid differentiation. Interestingly, the secretion of TNF-α and other related pro-inflammatory cytokines is elevated in low-risk (LR)-MDS, whereas regulatory T-cells (Tregs) and immunosuppressive cytokines such as IL-10 are increased in high-risk MDS patients as a consequence of the survival advantage gained by active suppression of a protective immune response[6].

IL-1β is a pleiotropic pro-inflammatory cytokine that plays multiple roles in both physiological and pathological conditions, including promotion of T cell maturation and B cell proliferation. The cytokine is synthesized in an inactive form (pro-IL-1β) by various cell types including monocytes, macrophages, dendritic cells and stromal components of the hematopoietic stem cell niche and is activated intracellularly by caspase 1. Secreted IL-1β binds to its cognate receptor (IL-1R1) and triggers release of growth factors or cytokines by a broad range of cells including lymphocytes, macrophages, endothelial cells and fibroblasts, thus modulating the hematopoietic stem cell (HSC) environment. After myeloablation and HSC transplantation, short-term IL-1β exposure appears to support HSC regeneration, while chronic exposure to IL-1β may promote uncontrolled HSC division and subsequent exhaustion of the HSC pool[7].

Since chronic immune stimulation accelerates clonal expansion, which in turn is a driver of MDS and AML development[7], it is likely that dysregulation of IL-1β plays a key role in hematological diseases.
One important hallmark of MDS is the activation of the NLRP3 inflammasome. In MDS, damage-associated molecular patterns (DAMPs) including S100A8/S100A9 increase NLRP3 expression in the bone marrow through TLR4 signaling and formation of reactive oxygen species (ROS). The resulting activation of caspase-1, generation of IL-1β and increased pyroptotic cell death appears to contribute to the MDS phenotype. Moreover, MDS specimens display marked upregulation of inflammasome transcripts, including mRNAs encoding IL-1β and IL-18. These are increased 3.7-fold and 29.6-fold in LR MDS compared to normal donors, whereas expression was increased to a lesser extent (1.1-fold and 9.2-fold) in higher-risk MDS specimens. Interestingly, inhibition of NLRP3 or neutralization of S100A9 restores effective hematopoiesis, confirming the importance of this pathway as a promising therapeutic target in MDS[8,9].

1.2 Rationale

1.2.1. Hypothesis

On the basis of these data, we propose to investigate the efficacy and safety of single agent anti-IL-1β monoclonal antibody canakinumab in LR MDS (IPSS-R very low/low/ intermediate, up to 3.5) and MDS/MPN patients with symptomatic anemia refractory/intolerant/ineligible to standard therapy with ESA. The only currently approved treatment option for LR MDS patients with anemia are ESAs. However, response to ESAs is very limited (below 50%) and mostly only transient, suggesting an unmet medical need for novel therapeutic options for these patients with anemia lacking or losing response to ESAs[10].
The IL-1ß neutralizing antibody canakinumab has been FDA and EMA approved for the treatment of cryopyrin-associated periodic fever syndromes and active systemic juvenile idiopathic arthritis. Moreover, canakinumab is approved for gouty arthritis in the EU. Based on the rationale that chronic inflammatory state drives early disease progression in MDS[11], we aim at demonstrating that single agent canakinumab might be able to prevent or delay disease progression in LR MDS patients. Moreover, after IL-β blockade, inhibition of erythroid progenitor colony-forming capacity by pro-inflammatory cytokines should be interrupted, resulting in a reactivation of erythropoietin gene expression and improvement of anemia[12].
We will use the restoration of erythropoiesis to indicate response and focus on patients who failed standard ESA therapy in order to explore the potential of canakinumab as additional treatment option. The CANFIRE trial will be the first study investigating canakinumab in hematological malignancies. If successful, our targeted treatment approach can be the basis for further phase 2/3 studies investigating canakinumab in MDS and MDS/MPN patients with the potential to change the current treatment landscape.
The selected patient group actually represents the majority of the overall MDS and MDS/MPN population. Additionally, we decided to monitor the variant-allele frequency (VAF) as an indicator of clonal evolution during treatment (concurrent translational project). We exclude patients with severe neutropenia or thrombocytopenia, in order to limit a potential increased rate of infections or bleeding during canakinumab treatment.
The study will provide fresh insights into the potential of anti-inflammatory therapy to modulate the natural course of LR MDS[6,13,11,14,15] and will possibly pave the way for a new therapeutic approach that takes effective advantage of the important window of opportunity opened up by the increasingly early detection of MDS.
In order to investigate the biological aspects of the trial and its hypotheses we have built a translational program to analyse different aspects of the IL-1β blockade and NLRP3 activation in the enrolled patient population (section 9 “CONCOMITANT SCIENTIFIC PROJECTS”).

1.3 Risk-Benefit Consideration

Anemia is the predominant cytopenia in MDS. As detailed above, treatment with currently approved approaches remains limited. Epoetin alfa (EPO) has been approved by the EMA in 2017 and is the first and only prescribable product for MDS-related anemia since then (beyond RBC transfusions). However, EPO is only indicated if sEPO levels are between 0 and 200 mU/ml and of those who are eligible, only about 30-50% show a response. Consequently many patients with lower-risk MDS still require RBC transfusion as a result of lacking alternatives. RBC transfusions are associated with fatigue, impaired quality of life, increased cardiovascular risks and toxic iron overload[2], thus new therapeutic options are urgently sought.
As detailed above, canakinumab might positively influence erythropoiesis though blocking IL-1β and by this help to improve MDS-related anemia. Canakinumab is a monoclonal antibody and currently approved in Germany for a variety of inflammatory disorders. It is generally well tolerated and its use is approved in children. The drug can be administered intravenously or subcutaneously. The maximum blood concentration is found after 7 days of a single subcutaneous dose, and its half-life is 26 days. In previous studies, decreases in total WBC and mean platelet count were observed on canakinumab compared to placebo with no clear dose-related pattern. There was also a decrease in absolute neutrophil counts (ANC) with no clear association with higher infection rates. Interestingly, published data show a trend towards higher hemoglobin levels persisting for at least 5 months of treatment in canakinumab groups compared to placebo[16]. To reduce the risk of bleeding or infections after canakinumab treatment, we will exclude patients with initially severe thrombocytopenia (PLT <30 Gpt/L) or neutropenia (ANC<0.5 Gpt/l) (see “exclusion criteria” below).
Given the above, we consider the risk-benefit ratio of this trial as favourable.

2 OBJECTIVES

2.1 Primary Objective

To evaluate the proportion of patients who have an erythroid response (HI-E) according to the modified IWG 2018 criteria.

2.2 Secondary Objectives

- To evaluate the HI-E response duration
- To evaluate time to HI-E
- To evaluate hemoglobin changes from baseline
- To evaluate neutrophil and platelet (HI-N and HI-P) responses
- To evaluate disease progression according to IWG 2018 criteria
- To evaluate safety and toxicity of single agent canakinumab
- To assess the impact of canakinumab on quality of life (QoL)

3 TRIAL DESIGN AND DESCRIPTION

3.1 Trial Design

This is a Phase II, single-arm, open-label multicenter study, to evaluate the effects of canakinumab monotherapy on anemia in patients with very low, low or intermediate risk MDS or MDS/MPN who are not currently receiving treatment with ESAs.
After screening and assessment for eligibility, patients will be treated with canakinumab over 8 cycles of 21 days each with subcutaneous canakinumab administration at day 1 of each cycle. At the end of cycle 8 patients will be assessed for response (primary endpoint, HI-E according to IWG 2018 criteria[1]). Responders will be treated further for maximal up to three years total treatment time. Non-Responders will finish study treatment after the first six months. After end of treatment patients will be followed-up for another 6 months, resulting in a maximum trial participation phase of 12 months for non-responder and maximum 42 months for long-term responder (see Flow Chart above).

3.2 Requirements at the Trial Sites regarding Personnel and Equipment

Qualification of investigator/deputy and medical staff in the trial team
This chapter defines general requirements for trial staff in the CANFIRE trial as required by GCP. Trial specific and/or national requirements regarding the qualification of the trial sites and trial staff are specified in the qualification documents used for application to the institutional review board in more detail.

The **coordinating investigator** and his deputy are licensed to practice medicine and have at least two years of working experience in the study specific indication. They have theoretical and practical experience in conducting clinical trials.

Investigators and their deputies (according to German Medicinal Products Act §40 (1a) are licensed to practice medicine, and have at least two years of work experience in treating patients with MDS and MDS/MPN. They have theoretical and practical experience in conducting clinical trials.
The Investigator is responsible for selecting and assembling the trial team members (especially the medical staff) according to the requirements of this trial protocol and the specification in the qualifications documents the sponsor will provide to each trial site. Furthermore, the investigator is responsible for training and supervision of the trial team and providing all necessary information during the course of the trial. This has to be documented accordingly.
Prior to the start of the trial all staff involved in the study will receive specific training on the study conduct in general, the diagnostic measures, the response criteria for endpoint evaluation, the handling of eCRF, safety measures, canakinumab handling and administration, etc.
The **investigators** agree, when signing the protocol, to adhere to the instructions and procedures described in it and thereby to adhere to the principles of Good Clinical Practice that it conforms to.
The responsibilities of the **investigators** include:

- Understanding of the properties of the study medication as described in the Summary of product characteristics (SmPC)
- Execution of the treatment plan
- Sufficient time and capacity to perform the clinical study
- Correct collection and documentation of study-related data and reporting,
- Provision of data for the sponsor, for the monitor, and for audits/inspections
- Ensuring strict confidentiality and requesting similar confidentiality from her/his staff concerning information about participants and information provided by the sponsor.

Study documents provided by the sponsor (protocols, CRFs and other material) will be stored appropriately to ensure their confidentiality. The information provided by the sponsor to the investigator may not be disclosed to others without direct written authorisation from the sponsor, except to the extent necessary to obtain informed consent from patients who wish to participate in the trial,

- Providing financial disclosure
- The clinical investigator has full responsibility for the conduct of the clinical study in the study center.

3.2.1 **Essential technical equipment at the trial sites and involvement of other facilities in the trial**

Study sites must be equipped with the following:

- Refrigerator with temperature control to store canakinumab (2°C-8°C)
- Internet access for communication purposes and in order to use the provided eCRF system for documentation
- Storage space for trial-specific documents (e.g. ISF, manuals)
- Refrigerator (4°C), Freezer (-20°C), centrifuge (for processing BM / PB samples)
- Local cytogenetics facility (for karyotype / cytogenetic profiling)
- Local facility for molecular genetics (for NGS / MDS panel / molecular profiling)
- Facility for preparation of PB and BM slides, which will be sent to the central lab
- Hematology lab for routine analyses (e.g. CBC)

3.3 Trial Sites and Number of Trial Subjects

About six study sites in Germany will participate in the CANFIRE trial and recruit n=50 patients in order to achieve a minimum of n=41 patients to be analysed.
The participating sites are renowned MDS specialist centers and members of the German MDS Study Group (D-MDS). They have a large MDS patient pool, a large catchment area in the respective regions and excellent contact with the office-based hematologists in their areas who are regularly referring patients for inclusion in clinical trials.

Due to premature termination of enrolment total recruitment period is reduced to 23 months. Patients on treatment will be treated up to primary end-point visit or end of treatments visit, whatever comes first.

Four clinical trial sites have screened patients, up to now. Sites without patients will be closed upon authorities' approval of premature termination of recruitment.

3.4 Expected Duration of Trial

Within a recruitment period of 23 months 10 patients were included.
Start of the study is defined as date of the first written informed consent. End of study is defined as the last visit of the last patient (LPLV).

<u>Patient related duration</u>:
Treatment: up to three years
Follow-up: 2 months
Total duration for individual patients: 12 months (non-responder)

<u>Trial related duration</u>:
Recruitment period: 23 months
First patient in (FPI) to last patient out (LPO): + 2 months after loss of response of last patient

The duration of the entire trial cannot be estimated, as responders are treated up to loss of response.

3.5 Premature Termination of the Trial

Premature termination of the trial for a single patient is described in chapter 6.5.1.

3.5.1 Termination of the Trial at a Single Site

The trial can be aborted at a single site if:

- the protocol is not adhered to,
- the quality of data is deficient,
- there is inadequate recruitment.

The coordinating investigator decides whether or not to exclude the site, together with the sponsor and biometrician.
Investigators and sites no longer participating in the trial must inform the coordinating investigator immediately and should provide justification for the decision. Further treatment of patients still involved in the trial has to be arranged together with the coordinating investigator.
A trial site may be temporarily placed on hold or closed by the sponsor if no or insufficiently qualified personnel (investigator/deputy) is available.

3.5.2 Termination of the entire Trial

Both the sponsor and the investigator reserve the right to terminate the study according to the study contract. The sponsor has the right to terminate the trial prematurely if there are any relevant medical or ethical concerns, or if completing the trial is no longer practicable.
All closures should occur only after consultation between the sponsor, the coordinating investigator and the study sites. All affected institutions (e.g. IEC(s), competent authorities study center) must be informed as applicable according to legal requirements. All study materials (except documentation that has to remain stored at site) must be returned to the sponsor. The investigator will retain all other documents until notification given by the sponsor for destruction.

Premature termination of the trial will be considered if:

- The risk-benefit ratio becomes unacceptable based on safety findings or any interim analysis from this study or upcoming information from other clinical or animal studies
- It is no longer ethical to continue treatment with the IMP
- The sponsor considers that the trial must be discontinued for safety reasons
- The study conduct does not suggest a proper completion of the study within a reasonable time frame

The final decision regarding the premature termination of the trial will be made by the coordinating investigator/the sponsor or the authorised representative.
Since the trial is subject to German Medicinal Products Act (Arzneimittelgesetz – AMG), the approval can be rescinded or the trial can be terminated by the responsible federal authority (competent authority - CA) or the responsible ethics committee.

4 TRIAL SUBJECTS

For this protocol we will recruit lower-risk MDS (IPSS-R up to 3.5: very low/low/intermediate) or MDS/MPN patients with less than 10% bone marrow blasts and symptomatic anemia.

Patients will be included in the trial in a <u>two-step procedure</u>:

1. <u>Registration</u> for the study with informed consent, preliminary check of inclusion/exclusion criteria, and initiation of study-specific screening investigations (see 4.1.1). Patients should only be registered if valid baseline data are expected to be available at the end of the baseline evaluation period.

2. <u>Formal inclusion</u> with a final check of inclusion and exclusion criteria (see 4.1.2) after completion of all study-specific screening investigations at the end of the baseline evaluation period and shortly before start of treatment.
The **baseline evaluation period** overlaps with the screening period and consists of the **last 16 complete weeks** (Monday to Sunday, 112 days) before the day of formal inclusion (see Figure 1).
Further details on enrollment process can be found in chapter 6.2.

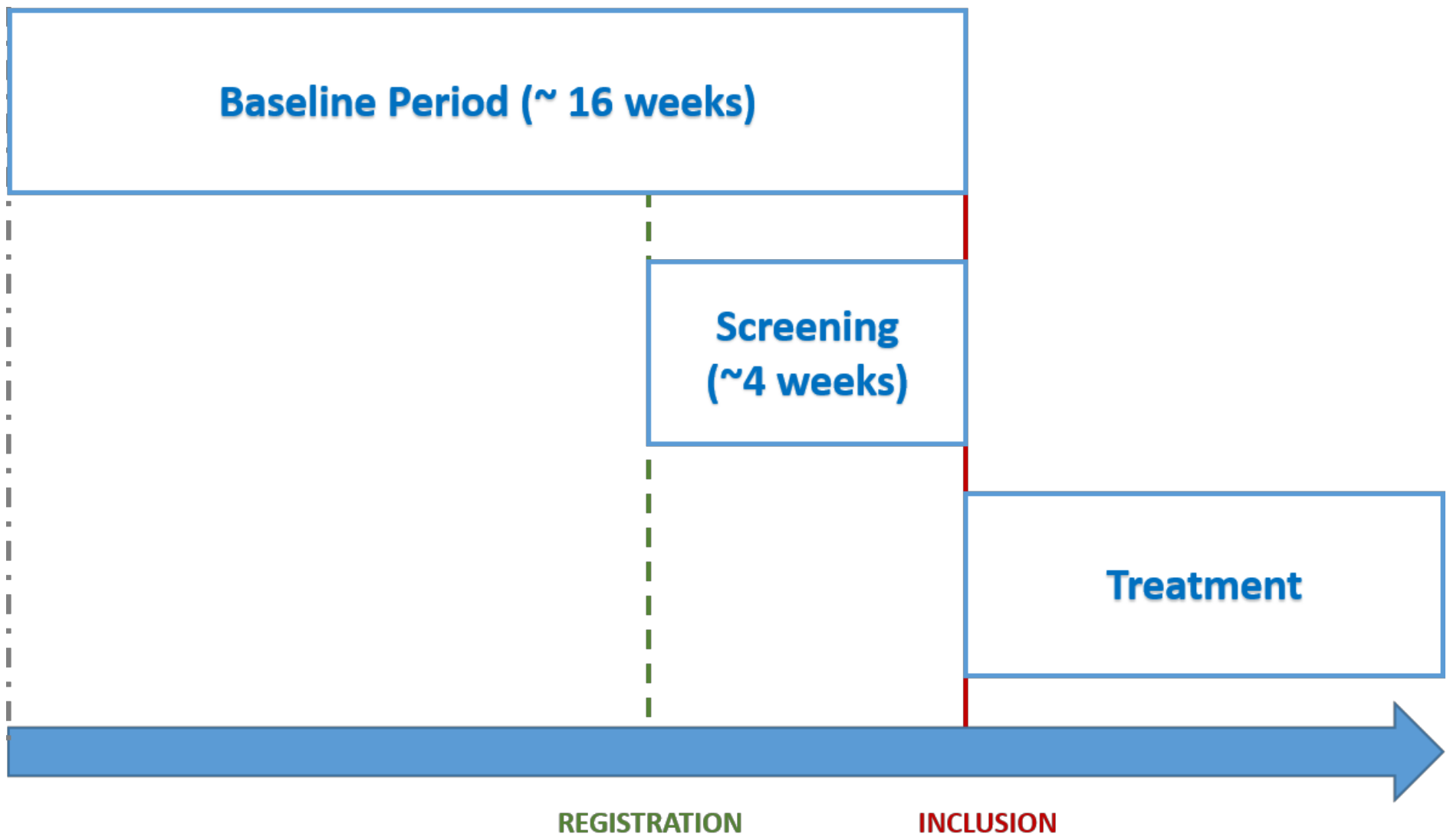

Figure 1: Enrollment process

4.1.1 I/E criteria for patient registration for screening investigations

4.1.1.1 Inclusion criteria

Each patient must meet all of the following inclusion criteria to be enrolled in the trial.

Target population

1) **Diagnosis of**
   a) Lower-risk **myelodysplastic syndrome (MDS)**[17] OR
   b) **Myelodysplastic/myeloproliferative neoplasm (MDS/MPN)** including MDS/MPN-RS-T, MDS/MPNu, aCML, or CMML (as per the World Health Organization [WHO] 2016 classification)
   Note: Diagnoses will be confirmed by <u>central morphological review during screening assessment</u>
2) **Very low, low or intermediate risk disease MDS with up to 3.5 points** according to the revised International Prognostic Scoring System (IPSS-R) classification (to be

confirmed during screening assessment). For MDS/MPN < 10% bone marrow blasts at screening. For CMML low or intermediate risk according to CMML-Specific Prognostic Scoring System (CPSS Score).

3) **Symptomatic anemia (all NTD, LTB, or HTB):** has to be documented in the 16 weeks baseline period ending on the day of inclusion. Patients should be registered only if it is expected at time of registration that
   a) **a valid and complete hemoglobin and transfusion history** will be available at inclusion AND
   b) the **hemoglobin mean** over the baseline period will be **less than 10 g/dL OR three or more RBC-transfusions** will have been given during the baseline period documenting **transfusion dependence.**

4) **Documented transfusion strategy**: A transfusion trigger threshold is needed which characterizes the transfusion strategy - ideally for the whole baseline period, but at least for the time from registration to the end of the study.
5) Relapsed / refractory / intolerant / ineligible (endogenous serum erythropoietin levels ≥ 200 U/L) to ESA treatment

Regulatory requirements
- Age ≥ 18 years
- Written informed consent

Special considerations for clinical trials during Covid-19 pandemic

Participants who have had a known infection of SARS CoV-2 or COVID-19 are permitted, if PCR test is negative prior to the screening visit and if patients are free of COVID-19 symptoms. Patients with a complete COVID-19 vaccination history do not need to be tested, unless they are symptomatic, according to institutional policy and/or local regulatory guidelines.

Because canakinumab is used as an immunosuppressant, it cannot be excluded that asymptomatic SARS-CoV-2 infections may be exacerbated by study treatment. Therefore, regular testing for SARS-CoV-2 during therapy is recommended for all patients.

4.1.1.2 Exclusion Criteria

Patients meeting any of the following exclusion criteria are not to be enrolled in the trial.

Compliance with major study procedures
1) Patient does not accept bone marrow sampling during screening and treatment period.
2) Patient does not accept peripheral blood sampling for screening and during treatment.
3) Patient does not accept subcutaneous application of canakinumab every three weeks

Contraindications
4) Known clinically significant anemia due to iron, vitamin B12, or folate deficiencies, or autoimmune or hereditary hemolytic anemia, or gastrointestinal bleeding
   a. iron deficiency must be determined by calculated transferrin saturation (iron/total iron binding capacity) ≤ 20%, or serum ferritin ≤ 15 µg/L
5) Prior allogeneic or autologous stem cell transplant
6) Known history of diagnosis of AML

Safety
7) Severe neutropenia defined by ANC ≤ 0.5 Gpt/l
8) Severe thrombocytopenia with platelets (PLT) < 30 Gpt/L
9) Serum creatinine > 1.5x ULN OR measured or calculated creatinine clearance < 40 ml/min (*NOTE: creatinine clearance should be calculated per institutional standard. GFR can also be used*)

10) Serum aspartate aminotransferase/serum glutamic oxaloacetic transaminase (AST/SGOT) or alanine aminotransferase/serum glutamic pyruvic transaminase (ALT/SGPT) ≥ 3.0 x upper limit of normal (ULN) → both have to be measured
11) Eastern Cooperative Oncology Group (ECOG) > 2
12) Total bilirubin ≥ 2.0 x ULN
    a. higher levels are acceptable if these can be attributed to active red blood cell precursor destruction within the bone marrow (ie, ineffective erythropoiesis).
    b. subjects are excluded if there is evidence of autoimmune hemolytic anemia manifested as a corrected reticulocyte count of > 2% with either a positive Coombs' test or over 50% indirect bilirubin
13) Active second malignancy at time of study entry
14) Prior history of malignancies, other than MDS, unless the subject has been free of the disease for ≥ 5 years. However, subjects with the following history/concurrent conditions are allowed:
    a. Basal or squamous cell carcinoma of the skin
    b. Carcinoma in situ of the cervix
    c. Carcinoma in situ of the breast
    d. Incidental histologic finding of prostate cancer (T1a or T1b using the tumor, nodes, metastasis [TNM] clinical staging system)
15) Major surgery within 8 weeks prior to screening (*NOTE: Subjects must have completely recovered from any previous surgery prior to inclusion*)
16) Myocardial infarction, uncontrolled angina, uncontrolled heart failure, or uncontrolled cardiac arrhythmia within 6 months prior to screening
17) Positive test result as an indicator for active or latent tuberculosis (evaluation performed per local treatment guidelines or clinical practice)
18) Subjects with suspected or proven immunocompromised state or infections, including:
    a. Known history of testing positive for Human Immunodeficiency Virus (HIV) infections.
    b. Known active or recurrent, hepatitis B and C (positive or indeterminate laboratory results).
    c. Those with any other medical condition such as active infection, treated or untreated, which in the opinion of the investigator places the subject at an unacceptable risk for participation in immunomodulatory therapy.
    d. Those requiring systemic or local treatment with any immune modulating agent in doses with systemic effects e.g.: Prednisone >20 mg (or equivalent) oral or intravenous daily for >14 days; Prednisone > 5 mg and ≤ 20 mg (or equivalent) daily for > 30 days; Equivalent dose of methotrexate >15 mg weekly.
    e. History of severe allergic or anaphylactic reactions or hypersensitivity to recombinant proteins or excipients in the investigational product (see SmPC).

Excluded treatments before and during study treatment

19) Anticancer cytotoxic chemotherapeutic agent or treatment for at least 14 days prior to registration and during study treatment
20) Corticosteroids or other immunosuppressive therapies (TNF-Blocker, other IL-1 Blocking Agent, Disease-modifying anti-rheumatic drugs (DMARDs) including ciclosporin, cyclophosphamide, hydroxychloroquine, leflunomide, methotrexate, mycophenolate, sulfasalazine) for at least 14 days prior to registration and during study treatment
21) Treatment with ESAs, luspatercept, granulocyte colony-stimulating factor (G-CSF), granulocyte-macrophage colony-stimulating factor (GM-CSF) for at least 14 days prior to registration and during study treatment
22) Treatment with disease modifying agents (eg, immune-modulatory drugs [IMiDs such as lenalidomide, hypomethylating agents] or experimental agents for underlying MDS disease for at least 14 days prior to registration and during study treatment

23) Prior treatment with canakinumab
24) Live vaccination during study treatment

Special considerations for females

25) Pregnant or breastfeeding females
26) Positive pregnancy test in women of childbearing potential
NOTE: Urine or serum pregnancy test should be repeated within 3 days prior to receiving study treatment. If the urine test is positive or cannot be confirmed as negative, a serum pregnancy test is required additionally
27) Female subjects of childbearing potential unwilling to use an adequate method of contraception for the course of the study through 90 days after the last dose of study medication
NOTE: Abstinence is acceptable if this is the usual lifestyle and preferred contraception for the subject

Special considerations for males

28) Male subjects with procreative capacity not willing to use an adequate method of contraception, starting with the first dose of study therapy through 90 days after the last dose of study therapy
NOTE: Abstinence is acceptable if this is the usual lifestyle and preferred contraception for the subject

Regulatory requirements

29) Participation in other interventional trials
30) Patients under legal supervision or guardianship

4.1.2 Inclusion/Exclusion criteria for definite patient inclusion

4.1.2.1 Inclusion criteria to be checked after screening assessments

Each patient must meet all of the inclusion criteria mentioned in 4.1.1.1 and additionally the following to be enrolled in the study:

a) Diagnosis confirmed by central morphology review
   - MDS or
   - MDS/MPN including MDS/MPN-RS-T, MDS/MPNu, aCML, and CMML
b) Confirmed disease risk
   - IPSS-R up to 3.5 (MDS); confirmed by central morphology review
   - < 10% bone marrow blasts (MDS/MPN), confirmed by central morphology review
   - CPSS Score low or intermediate (CMML)
c) Symptomatic Anemia confirmed by formal algorithm (based on complete hemoglobin time course and transfusion history for the baseline period documented in the study database):
   - **NTD (non transfusion dependent):** < 3 RBC transfusions and mean hemoglobin[10] level <10 g/dl within the last 16 weeks
   - **LTB (low transfusion burden):** 3-7 RBC transfusions within the last 16 weeks in at least two transfusion episodes
   - **HTB (high transfusion burden)**: ≥ 8 RBC transfusions within the last 16 weeks

[10] Hb measurements ideally available every two weeks. At least five valid (> 7 days after RBC event) Hb measurements that span at least 100 days within the baseline evaluation period.

4.2 Justification for the Inclusion of vulnerable populations

This clinical trial will not include particularly vulnerable individuals as defined by § 40 chapter 4 and § 41 chapters 2 and 3 AMG.

4.3 Participation in more than one Clinical Trial

During the verification of the inclusion and exclusion criteria the investigator/his deputy or authorised medical staff of the trial team checks if the patient is currently participating in any other interventional clinical trial(s). Should this be the case, the patient will not be included.
Moreover, by signing the informed consent form, the patient confirms that he is not participating in any other interventional clinical trial simultaneously.

4.4 Statement on the Inclusion of Dependent Individuals

During the screening procedure, all patients will be interviewed concerning any potential relationship to the investigator/ his deputy or to medical staff of the trial team or the coordinating investigator or the sponsor.
In case of a potential benefit of the trial for the patient he should not be excluded from the trial-However, if any dependency exists, the patient cannot be included by the person he is dependent on.

4.5 Rationale for Gender Distribution

Canakinumab is investigated for the treatment of male and female patients with MDS, all patients fulfilling the inclusion and exclusion criteria - of both genders - will be included in the present study.

5 INVESTIGATIONAL PRODUCT

5.1 Trial Drugs

Generic Name:	Canakinumab, ACZ885 (Ilaris®)
Address Manufacturer:	Novartis Pharma AG, Lichtstrasse 35, CH-4056 Basel, Switzerland
Marketing authorisation holder:	Novartis Pharmaceuticals UK Limited<br>2nd Floor, The WestWorks, White City Place<br>195 Wood Lane<br>London<br>W12 7FQ<br>United Kingdom
Drug allocation:	Provided by Novartis
Batch number:	Not yet available
Formulation used:	150 mg/ml solution for injection (1 ml solution in a single dose glass vial)<br>Human monoclonal antibody produced in mouse myeloma Sp2/0 cells by recombinant DNA. Patients will be administered 200 mg canakinumab per dose.<br>Excipients: mannitol, histidine, histidine hydrochloride monohydrate, polysorbate 80, water for injections.<br>The solution is clear to opalescent and colourless to slightly brownish yellow.<br>For subcutaneous use.
Packaging:	One vial contains 150 mg of canakinumab<br>Each ml of solution contains 150 mg canakinumab.
Storage conditions:	Store in a refrigerator (2° to 8°C). Do not freeze. Store in the original package in order to protect from light.
Stability:	Shelf life: 3 years.<br>From a microbiological point of view, the product should be used immediately after first opening.<br>The solution should be free of visible particles and clear to opalescent. The solution should be colourless or may have a slight brownish-yellow tint. If the solution has a distinctly brown discolouration it should not be used.
Preventive measures/Incompatibility:	Traceability:<br>In order to improve the traceability of biological medicinal products, the name and the batch number of the administered product should be clearly recorded.<br>Infections:<br>Canakinumab is associated with an increased incidence of serious infections. Therefore, patients should be monitored carefully for signs and symptoms of infections during and after treatment with canakinumab. Physicians should exercise caution when administering canakinumab to patients with infections, a history of recurring infections, or underlying conditions which may predispose them to infections.

5.2 Packaging and Labelling of the Trial Drug

The investigational product will be manufactured, packaged and labelled by Novartis according to the standards of Good Manufacturing Practice (GMP).

The trial drug will be labelled as required by the ICH Guideline E6 and the German Medicinal Products Act (AMG § 10). The labelling will be in German. The sponsor is responsible for label design and approval.
The master label is part of the documents that will be submitted to the responsible federal authority.
The investigational product will not be shipped before all requirements for the start of the clinical trial have been fulfilled according to the sponsor's requirements (regulatory green light). The trial medication will be shipped by Novartis to the participating trial sites after the sponsor´s release for shipment. A detailed documentation of every shipment, number of vials and packages, which will be used in this clinical trial will be made and kept on file.

5.3 Storage and Handling Requirements

The investigator shall take responsibility for and shall take all steps to maintain appropriate records and ensure appropriate supply, storage, handling, distribution and usage of investigational product in accordance with the protocol and any applicable laws and regulations.
The investigational product must be stored in a secure, limited-access location under the storage conditions specified above.
Receipt and dispensing of trial medication must be recorded by an authorised person at the trial site.
Clinical supplies may not be used for any purpose other than that stated in the protocol.

5.4 Drug Accountability

All shipments will be coordinated by Novartis. After initiation of the trial site by the sponsor the delivery of trial medication is triggered. Novartis will send trial medication to the site for first patients after the sponsor´s release.
Storage, application and destruction of (e.g. unused) IMP will be carried out by the responsible personnel at the trial site.
The investigator is responsible for the appropriate storage at the trial site and the detailed explanation of application of trial drugs to patients. The investigator or designated personnel will inventory and acknowledge receipt of all trial drug shipments. All trial drugs must be kept in a locked area with access restricted to designated trial personnel.

The compliance should be evaluated at on-site monitoring visits defined in the monitoring plan. The medical staff at the trial site and the monitor from the ZKS appraise the drug accountability as part of the on-site checks.

For each trial subject an individualized drug accountability log will be recorded at trial site.
Unused trial medication and empty canakinumab vials/packages are collected and destroyed at the trial site. The site monitor will assure that a final report of drug accountability is prepared and placed in both the Investigator Site File (ISF) and the Trial Master File (TMF).
The application of canakinumab is documented in the patient's medical record AND the respective parts of the Case Report Form (CRF). Orderly disposal of canakinumab will be accomplished and documented by the investigator/designated personnel. Disposal will be performed according to local standards after release by the monitor. Storage at the trial sites and administration to patients will be documented by the investigator/designated personnel.
Upon completion or termination of the trial, all unused and/or partially used investigational product will be destroyed at the site per institutional policy provided that procedures for proper disposal have been established according to applicable federal, state, local and institutional guidelines. The investigator is responsible to arrange for disposal of all empty canakinumab vials/packages and to keep appropriate records of disposal.

5.5 Administration of Trial Drug

5.5.1 **Procedures**
200 mg canakinumab will be administered subcutaneously every three weeks (21 days) for 8 cycles/a duration of 6 months (non-responder) or up to three years (responder).
Canakinumab will be provided as 150 mg/ml solution for injection.

Instructions for use:

Allow the vial to warm to room temperature before injection. The solution should be practically free of visible particles and clear to opalescent. The solution should be colorless or may have a slight brownish-yellow tint.
Using an 18 G or 21 G x 2 inch needle (or similar as locally available) and a >1.5 ml syringe, carefully withdraw the required volume depending on the dose to be administered. Once the required volume is withdrawn, recap and remove the withdrawal needle from the syringe and attach a 27 G x 0.5 inch needle (or similar as locally available) to immediately inject the solution subcutaneously.

Subcutaneously inject canakinumab in the following injection sites: upper thigh, abdomen, upper arm or buttocks. It is recommended to select a different injection site each time the product is injected to avoid soreness. Broken skin and areas which are bruised or covered by a rash should be avoided. Injection into scar tissue should be avoided as this may result in insufficient exposure to canakinumab.

Disposal:
Any unused medicinal product or waste material should be disposed of in accordance with requirements stated in this protocol (see section 5.4).

Dose reduction:
Standard dose for canakinumab in this trial is 200 mg, applied every three weeks. In case of tolerability problems, the dose can be reduced in the following cycle to 150 mg and/or, if needed, to 100 mg.

Stopping criteria:
The treatment will be terminated prematurely in case of:
- grade 3-4 toxicity unresponsive to dose reduction
- disease progression
- withdrawal of consent
- loss of contact
- on physicians decision

5.5.2 **Compliance**
Canakinumab will be administered as subcutaneous injection by the qualified study personnel on site. The monitor appraises the drug accountability and thus compliance to the treatment scheme as part of the on-site checks.

Patients will only be included, if no option of an approved MDS therapy (according to decision of the treating physician) is available:
- Erythropoiesis-stimulating agents (ESA) is indicated for the treatment of symptomatic anaemia (hemoglobin concentration of ≤ 10 g/dL) in adults with lower-risk MDS who have low serum erythropoietin (< 200 mU/mL).
- Luspatercept: is approved e.g. for patients with lower-risk MDS with ring sideroblasts and transfusion dependency who failed erythropoiesis-stimulating agents. But,

luspatercept is not currently a licensed therapeutic option for patients without ring sideroblasts.
- Lenalidomide: is approved for lower-risk MDS patients with isolated deletion in chromosome 5 (del 5q) who are RBC transfusion-dependent due to anemia.
- alloHSCT: should be the preferred treatment strategy for young patients with high-risk cytogenetics, provided a donor is available and the patient's general condition is good.

5.5.3 **Dealing with Side-effects**

Summary of the safety profile (SmPC, May 2021)

The most frequent adverse drug reactions were infections predominantly of the upper respiratory tract. No impact on the type or frequency of adverse drug reactions was seen with longer-term treatment.

Hypersensitivity reactions to the active substance or to any of the excipients have been reported in patients treated with canakinumab: The majority of these events were mild in severity. During clinical development of canakinumab in over 2,600 patients, no anaphylactoid or anaphylactic reactions attributable to treatment with canakinumab were reported. However, the risk of severe hypersensitivity reactions, which is not uncommon for injectable proteins, cannot be excluded.

Opportunistic infections have been reported in patients treated with canakinumab: Canakinumab is associated with an increased incidence of serious infections. Therefore patients should be monitored carefully for signs and symptoms of infections during and after treatment with canakinumab.

Known adverse reactions are (please always refer to the current SmPC)[11]:

Very common: Respiratory tract infections (including pneumonia, bronchitis, influenza, viral infection, sinusitis, rhinitis, pharyngitis, tonsillitis, nasopharyngitis, and upper respiratory tract infection), ear infection, cellulitis, gastroenteritis, urinary tract infection, upper abdominal pain, injection site reaction, arthralgia, decreased creatinine renal clearance, proteinuria and leukopenia

Common: Vulvovaginal candidiasis, dizziness/vertigo, musculoskeletal pain, back pain and neutropenia

Uncommon: Gastro-oesophageal reflux disease and decreased platelet count

Long-term data and laboratory abnormalities in CAPS (Cryopyrin-associated periodic syndrome) patients

During clinical trials with canakinumab in CAPS patients mean values for hemoglobin increased and those for white blood cell, neutrophils and platelets decreased.

Elevations of transaminases have been observed rarely in CAPS patients.

Asymptomatic and mild elevations of serum bilirubin have been observed in CAPS patients treated with canakinumab without concomitant elevations of transaminases.

In the long-term, open-label studies with dose escalation, events of infections (gastroenteritis, respiratory tract infection, upper respiratory tract infection), vomiting and dizziness were more frequently reported in the 600 mg or 8 mg/kg dose group than in other dose groups.

Laboratory abnormalities in abnormalities in TRAPS, HIDS/MKD and FMF patients

Neutrophils

Although ≥ Grade 2 reductions in neutrophil count occurred in 6.5% of patients (common) and Grade 1 reductions occurred in 9.5% of patients, the reductions are generally transient and neutropenia-associated infection has not been identified as an adverse reaction.

Platelets

[11] Frequency categories: very common (≥ 1/10); common (≥ 1/100 to < 1/10); uncommon (≥ 1/1,000 to < 1/100); rare (≥ 1/10,000 to < 1/1,000); very rare (< 1/10,000); not known (cannot be estimated from the available data).

Although reductions in platelet count (≥ Grade 2) occurred in 0.6% of patients, bleeding has not been identified as an adverse reaction. Mild and transient Grade 1 reduction in platelets occurred in 15.9% of patients without any associated bleeding adverse events.

<u>Laboratory abnormalities in SJIA patients</u>

Haematology

In the overall SJIA programme, transient decreased white blood cell (WBC) counts ≤ 0.8 x LLN were reported in 33 patients (16.5%). In the overall SJIA programme, transient decreases in absolute neutrophil count (ANC) to less than 1 x 109/l were reported in 12 patients (6.0%). In the overall SJIA programme, transient decreases in platelet counts (< LLN) were observed in 19 patients (9.5%).

ALT/AST

In the overall SJIA programme, high ALT and/or AST > 3 x upper limit of normal (ULN) were reported in 19 patients (9.5%).

<u>Laboratory abnormalities in gouty arthritis patients</u>

Haematology

Decreased white blood cell counts (WBC) ≤ 0.8 x lower limit of normal (LLN) were reported in 6.7% of patients treated with canakinumab compared to 1.4% treated with triamcinolone acetonide. Decreases in absolute neutrophil counts (ANC) to less than 1 x 10^9/l were reported in 2% of patients in the comparative trials. Isolated cases of ANC counts < 0.5 x 10^9/l were also observed. Mild (< LLN and > 75 x 10^9/l) and transient decreases in platelet counts were observed at a higher incidence (12.7%) with canakinumab in the active-controlled clinical studies versus the comparator (7.7%) in gouty arthritis patients.

Uric acid

Increases in uric acid level (0.7 mg/dl at 12 weeks and 0.5 mg/dl at 24 weeks) were observed after canakinumab treatment in comparative trials in gouty arthritis. In another study, among patients who were starting on ULT, increases in uric acid were not observed. Uric acid increases were not observed in clinical trials in non-gouty arthritis populations.

ALT/AST

Mean and median increases in alanine transaminase (ALT) of 3.0 U/l and 2.0 U/l, respectively, and in aspartate transaminase (AST) of 2.7 U/l and 2.0 U/l, respectively, from baseline to end of study were seen in the canakinumab treated groups versus the triamcinolone acetonide-treated group(s), however the incidence of clinically significant changes (≥ 3 x the upper limit of normal) was greater for patients treated with triamcinolone acetonide (2.5% for both AST and ALT) compared with canakinumab-treated patients (1.6% for ALT and 0.8% for AST).

Triglycerides

In active-controlled gouty arthritis trials, there was a mean increase in triglycerides of 33.5 mg/dl in canakinumab treated patients compared with a modest decrease of -3.1 mg/dl with triamcinolone acetonide. The incidence of patients with triglyceride elevations > 5 x upper limit of normal (ULN) was 2.4% with canakinumab and 0.7% with triamcinolone acetonide. The clinical significance of this observation is unknown.

<u>Long term data from observational study</u>

A total of 243 CAPS patients (85 paediatric patients aged ≥ 2 to ≤ 17 years and 158 adult patients aged ≥ 18 years) were treated with canakinumab in routine clinical practice in a long-term registry study (mean of 3.8 years of canakinumab exposure). The safety profile of canakinumab observed following long-term treatment in this setting was consistent with what has been observed in interventional studies in CAPS patients.

<u>Elderly population</u>

There is no significant difference in safety profile observed in patients ≥ 65 years of age.

Tuberculosis screening

In approximately 12% of CAPS patients tested with a purified protein derivative skin test in clinical trials, followup testing yielded a positive test result while treated with canakinumab without clinical evidence of a latent or active tuberculosis infection.

It is unknown whether the use of interleukin-1 (IL-1) inhibitors such as canakinumab increases the risk of reactivation of tuberculosis. Before initiation of therapy, all patients must be evaluated for both active and latent tuberculosis infection. Particularly in adult patients, this evaluation should include a detailed medical history.

Appropriate screening tests (e.g. tuberculin skin test, interferon gamma release assay or chest X-ray) should be performed according to local recommendations. Patients must be monitored closely for signs and symptoms of tuberculosis during and after treatment with canakinumab. All patients should be instructed to seek medical advice if signs or symptoms suggestive of tuberculosis (e.g. persistent cough, weight loss, subfebrile temperature) appear during canakinumab therapy. In the event of conversion from a negative to a positive test, especially in high-risk patients, alternative means of screening for a tuberculosis infection should be considered.

5.5.4 **Interactions/Counterindicated/Forbidden Concomitant Medication**

Interactions between canakinumab and other medicinal products have not been investigated in formal studies.

An increased incidence of serious infections has been associated with administration of another IL-1 blocker in combination with TNF inhibitors. **Use of canakinumab with TNF inhibitors is not recommended** because this may increase the risk of serious infections.

The expression of hepatic CYP450 enzymes may be suppressed by the cytokines that stimulate chronic inflammation, such as interleukin-1 beta (IL-1 beta). Thus, CYP450 expression may be reversed when potent cytokine inhibitory therapy, such as canakinumab, is introduced. This is clinically relevant for CYP450 substrates with a narrow therapeutic index where the dose is individually adjusted. On initiation of canakinumab in patients being treated with this type of medicinal product, therapeutic monitoring of the effect or of the active substance concentration should be performed and the individual dose of the medicinal product adjusted as necessary.

No data are available on either the effects of live vaccination or the secondary transmission of infection by live vaccines in patients receiving canakinumab. Therefore, live vaccines should not be given concurrently with canakinumab unless the benefits clearly outweigh the risks. Should vaccination with live vaccines be indicated after initiation of canakinumab treatment, the recommendation is to wait for at least 3 months after the last canakinumab injection and before the next one.

The results of a study in healthy adult subjects demonstrated that a single dose of canakinumab 300 mg did not affect the induction and persistence of antibody responses after vaccination with influenza or glycosylated protein-based meningococcus vaccines.

5.5.5 Overdose and Abuse

Reported experience with overdose is limited. In early clinical trials, patients and healthy volunteers received doses as high as 10 mg/kg, administered intravenously or subcutaneously, without evidence of acute toxicity.

In case of overdose, it is recommended for the patient to be monitored for any signs or symptoms of adverse reactions, and appropriate symptomatic treatment instituted immediately.

All cases of misuse, application errors etc. should be documented, even without the occurrence of an AE or SAE. Overdose must be reported as an SAE whether or not there is an accompanying AE.

Overdose and abuse is quite unlikely within the CANFIRE trial because the canakinumab administration is done by the authorised study staff only.

6 INDIVIDUAL TRIAL PROCEDURES

6.1 Patient Information and Informed Consent

An authorised trial physician will seek the patient's consent before performing any trial specific medical procedures with the patient. Patients who are considered not competent to consent to participate, e.g. due to an insufficient level of understanding, cannot participate in the trial.
In accordance with international guidelines, the informed consent of trial participants will be in writing (written, dated and signed by the person performing the interview referred to below, and by the subject).
The patient's consent must refer explicitly to the collection and processing of health-related data. Therefore, the patient should be informed explicitly about the purpose of collecting the data and scope of what is to be collected and that personal data, including health related data, will be stored and used for analyses in a pseudonymized form.
Before obtaining informed consent, the potential trial participant will receive information regarding the clinical trial in an interview. It will be performed by a qualified medical member of the trial group authorised by the investigator for this task.
The interview has to cover the following items:

- the nature, objectives, benefits, implications, risks and potential inconveniences of the clinical trial
- the expected duration of the subject's participation in the clinical trial
- the information that the patient may withdraw his/her consent to participate at any time without giving reasons. The patient is to be informed that in case of revocation of his/her consent, the stored data may be used further, as may be necessary to
    - assess effects of the medicinal product being tested,
    - guarantee that the patient's personal interests are not adversely affected,
    - comply with the requirement to provide complete authorisation documentation.
- potential treatment alternatives
- follow-up measures in case of early termination of the trial for the patient or overall
- the applicable damage compensation system in case of damage to a patient
- the right on data access, rectification and withdrawal of personal data

The subject will have the opportunity to ask questions at any moment.
Adequate time will be provided for the subject to consider his or her decision.

6.1.1 Withdrawal of Informed Consent

Patients may withdraw their consent to participate at any time without giving reasons. Nevertheless, the patient should be asked for the reason of the premature termination after being informed that he does not need to do so. Information as to when and why a patient was registered and when he withdrew consent must be retained in the documentation.
The patient is to be informed that in case of revocation of his/her consent, the stored data may be used further (according to § 40 lit 2a sublit 3 to 5 German Medicinal Products Act), as may be necessary to

- assess effects of the drug being tested,
- guarantee that the patient's personal interests are not affected adversely,
- comply with the requirement to provide complete authorisation documentation.

Data no longer required for the aforementioned purposes shall be deleted immediately.

6.2 Enrolment in the Trial

Patients will be included in the trial in a <u>two-step procedure</u>:
1. <u>Registration</u> for the study with informed consent, preliminary check of inclusion/exclusion criteria, and initiation of study-specific screening investigations (see 4.1.1).

2. Formal inclusion with a final check of inclusion and exclusion criteria (see 4.1.1.1) after completion of all study-specific screening investigations at the end of the baseline evaluation period and shortly before start of treatment.

Registration:
Patients will be identified by the participating centres, either directly at the respective clinics or via referring physicians. Patients will exclusively be recruited at the participating trial sites, which are all specialised in MDS and also have the necessary infrastructure allowing to perform the required trial specific procedures. Once potential patients are identified, they will be asked for trial participation and informed consent by investigators or authorised medical personnel of the CANFIRE trial. If the patients are eligible for the trial according to the eligibility criteria and willing to participate, they can be registered. Patients should only be registered if valid baseline data are expected to be available at the end of the baseline evaluation period.
A web-based registration will be performed. The trial staff enters the data relevant for registration (e.g. date of registration and date of informed consent) into the database and will receive a registration confirmation afterwards (by e-mail/fax for filing in the ISF).
In cases, when the internet-based tool is not available (e.g. server shut down, internet connection blocked, etc.), the registration form can also be filled in by hand and sent to the ZKS Leipzig by e-mail (scan) or fax.

Formal inclusion:
The patient can be included if the following conditions are met:
a) final check of inclusion and exclusion criteria (see 4.1.2.1) after completion of all study-specific screening investigations and shortly before start of treatment.
b) central laboratory: confirmation of the inclusion criteria (IC 1: diagnosis, IC 2: IPSS-R; see chapter 4.1.2.1) and
c) mandatory documentation of the following minimum data in the study database for the whole baseline evaluation period (16 complete weeks (MO to SU) before day of inclusion):
- complete transfusion history
- complete Hb-measurement time course with at least five valid (i.e., not within 7 days after a transfusion) Hb measurements that span at least 100 days.

During the inclusion, a formal algorithm will check validity of this data in order to confirm symptomatic anemia and make sure the baseline data suffice to assess response therapy later.
Once conditions a) – c) are met, the patient can be formally included. The recruiting site receives a confirmation that treatment can start and are informed about the baseline Hb level and RBC-transfusion count as a reference for response assessment.
All assessments and procedures essential for trial inclusion are described below (see chapter 6.3).

6.2.1 Discovery of a Violation of the Eligibility Criteria after the Fact

In general, the violation of eligibility criteria is not a reason for premature withdrawal of the patient from the trial therapy or from the whole trial.
If a violation of a selection criterion is discovered after registration of a patient, this has to be documented in the eCRF, which will result in an automatic report to the responsible trial team members at the ZKS Leipzig.
After consultation with the coordinating investigator, the CANFIRE project manager of the ZKS Leipzig informs the investigator/deputy or authorised medical staff immediately regarding the further treatment of the patient. The patient's data will continue to be recorded unless the patient revokes his/her informed consent. For procedures after premature trial termination of a single patient see chapter6.5.2.
If it is decided that the patient discontinues his/her trial participation due to the violation of a selection criterion, data will be collected until the date of the decision of termination of his/her

participation in the trial. Patients being withdrawn based on the violation of a selection criterion might be replaced by recruitment of an additional candidate (for replacement rules see chapter 6.5). If the discontinuing patient had not yet received any trial medication, he/she is regarded as “screening failure” (also see chapter 6.5) and no EOT visit and no follow-up as per protocol is necessary. If the discontinuing patient had received at least one dose of trial medication, he/she is defined as “dropout” (also see chapter 6.5) and will undergo the EOT visit follow-up visits as per protocol after his/her discontinuation if consent is not withdrawn.

6.3 Description of Assessments and Procedures

6.3.1 Laboratory assessments

6.3.1.1 Bone marrow (BM) analyses and CBC with differential (central morphology, Düsseldorf)

Bone marrow analyses and CBC with differential will be performed by a central laboratory in Düsseldorf / Germany. For this purpose, 5 unstained bone marrow slides and 2 peripheral blood slides should be sent to the central lab in Düsseldorf. Address and shipment details are provided in the laboratory manual as part of the ISF. If the marrow cannot be aspirated, a biopsy should be performed. Besides the pre-defined BM analysis schedules (see Schedule of Assessments) a BM biopsy is taken whenever it seems necessary to the investigator.

6.3.1.2 Cytogenetic analyses (local lab)

The cytogenetic analysis with banding analysis (optional FISH) should be performed at local labs. Therefore 2 - 5 mL of bone marrow will be collected and analyzed. If the cytogenetic analysis was unsuccessful (this means that cytogenetic examination has been performed on BM or on blood material, but the result was considered as a failure; e.g. NN with < 10 mitoses), analysis must be repeated.

6.3.1.3 Molecular analyses (local lab)

Sequencing analyses with an MDS-specific panel (e.g. Illumina Trusight Myeloid Panel) will be carried out at screening, primary endpoint and EOT.

6.3.1.4 Serum chemistry (local lab)

For serum chemistry adequate volumes of peripheral blood should be collected. The following parameters should be analyzed:

- Electrolytes (at least Sodium, Potassium; Calcium, Magnesium)
- Lactate dehydrogenase (LDH)
- Aspartate aminotransferase (AST)
- Alanine aminotransferase (ALT)
- Gamma-glutamyl transferase (GGT)
- Alkaline phosphatase (AP)
- Creatine phosphokinase (CK)
- Serum albumin
- Total protein
- Bilirubin direct or total
- Creatinine
- Uric acid
- C-reactive protein (CRP)
- Glucose
- Quick
- Partial thromboplastin time (PTT)

For further instructions relating to laboratory assessment see laboratory manual.

6.3.1.5 <u>**Automated CBC (local lab)**</u>

For CBC an adequate volume of peripheral blood should be collected. The following parameters should be analysed:

- Red blood cells (RBC) [Erythrocytes]
- Red blood indices [MCH, MCV, MCHC]
- Hemoglobin (Hb)
- Hematocrit (HCT)
- Leukocytes (WBC) [Neutrophils, Lymphocytes, Monocytes, Eosinophils and Basophils]
- Thrombocytes (PLT)
- Absolute neutrophil counts (ANC)

6.3.1.6 <u>**Pregnancy test (local lab)**</u>

Serum or urine pregnancy testing ß-HCG is to be done during screening, at day 1 of cycle 1 (not more than 3 days prior to initiation of canakinumab), at day one of each following cycle and at end of treatment visit in female patients with childbearing potential.
Childbearing potential ends with sterilization or for women who are older than 50 years and who have been in the menopause for more than one year as well as for women who are post menopausal. In each case of delayed menstrual period (over one month between menstruations) confirmation of absence of pregnancy is required. This requirement also applies to women of childbearing potential with infrequent or irregular menstrual cycles. In such a case pregnancy testing is recommended once a week.

6.3.2 Assessments at screening

The following baseline assessments/procedures have to be conducted or obtained within 28 days prior to start of study treatment:

- Signed written informed consent
- Check of in- and exclusion criteria
- Demographics (age, sex, date of informed consent) and MDS- and MDS/MPN related medical history
- Concomitant and previous MDS-/MDS/MPN-specific medication
- Transfusion and Hb history (for 16 weeks preceding first canakinumab administration)
- Physical examination and vital signs (weight, height, blood pressure, heart rate)
- ECOG performance status
- Pregnancy test for female patients with child bearing potential
- Initial disease status
- Serum chemistry
- Automated CBC (*NOTE: Hb measurement needs to be repeated two weeks after screening visit, if central morphology result is still pending*)
- Cytogenetics from bone marrow
- Molecular genetics from peripheral blood or bone marrow
- **Central morphology (Düsseldorf)**
    - 2 peripheral blood smears for CBC differential (Düsseldorf, Germany)
    - 5 bone marrow smears for morphology (Düsseldorf, Germany)
- Sampling for concomitant research (**optional**)
    - Bone marrow: 15 ml Sodium Heparin
    - Peripheral blood: 7.5 ml Serum + 22.5 ml Sodium Heparin

- Buccal swab[12]

6.3.3 Assessments during and after start of study treatment

Please note: allowed timeframes for visits: ± 3d during treatment phase (cycle length 21 days), ± 14d for follow-up phase

Day 1 of each cycle

- Final check of in- and exclusion criteria before first canakinumab administration (only at cycle 1)
- Concomitant medication
- Physical examination and vital signs (weight, blood pressure, heart rate)
- ECOG performance status
- QoL assessment using the QUALMS and EORTC-C30 questionnaire
- Pregnancy test for female patients with child bearing potential
- Canakinumab administration
- Toxicity / AE assessment
- Serum chemistry
- Automated CBC
- Sampling for concomitant research (**optional**)
  - Peripheral blood: 7.5 ml Serum + 22.5 ml Sodium Heparin

Day 8 of cycle 1 (AND day 11±4 of cycle 2-n[13])

- Toxicity / AE assessment
- Serum chemistry
- Automated CBC

Primary end point visit

Primary endpoint visit should be performed 21±3 days after the eighth canakinumab dose and corresponds for non-responders to the end of treatment-visit. The primary endpoint visit includes the following assessments:

- Concomitant medication
- Physical examination and vital signs (weight, blood pressure, heart rate)
- ECOG performance status
- QoL assessment using the QUALMS and EORTC-C30 questionnaire
- Pregnancy test for female patients with child bearing potential
- Toxicity / AE assessment
- Response assessment
- Serum chemistry
- Automated CBC
- Cytogenetics
- Molecular genetics
- **Central morphology (Düsseldorf)**
  - 2 peripheral blood smears for CBC differential (Düsseldorf, Germany)

---

[12] If available locally, the following materials are recommended: Swabs with regular tip (coated with short Nylon), for example 4N6FLOQSwabs (ThermoFisher Scientific)
[13] If treatment is continued after the primary end point visit, the day-1 visit is sufficient. A further Hb value sampling on day 11 is obsolete for clinical responders in the case of early termination of enrolment.

  - 5 bone marrow smears for morphology (Düsseldorf, Germany)
- Sampling for concomitant research (**optional**)
  - Bone marrow: 15 ml Sodium Heparin
  - Peripheral blood: 7.5 ml Serum + 22.5 ml Sodium Heparin

<u>End of treatment visit (EOT)</u>
End of treatment visit should be performed 28 days (±3 days) after the last canakinumab dose. The visit is equivalent to primary endpoint for non-responders. The end of treatment visit includes the following assessments:

- Concomitant medication
- Physical examination and vital signs (weight, blood pressure, heart rate)
- ECOG performance status
- QoL assessment using the QUALMS and EORTC-C30 questionnaire
- Pregnancy test for female patients with child bearing potential
- Toxicity / AE assessment
- Response assessment
- Serum chemistry
- Automated CBC
- Cytogenetics
- Molecular genetics
- **Central morphology (Düsseldorf)**
  - Peripheral blood smears for CBC differential (Düsseldorf, Germany)
  - Bone marrow smears for morphology (Düsseldorf, Germany)
- Sampling for concomitant research (**optional**)
  - Bone marrow: 15 ml Sodium Heparin
  - Peripheral blood: 7.5 ml Serum + 22.5 ml Sodium Heparin

6.4 Assessments during follow-up (FU)

Follow-up will be performed 56 ± 14 days after EOT visit (via telephone).
During both FU visit toxicity/AEs, concomitant medication and patients' disease status will be assessed.

6.5 Premature Termination of the Therapy or Follow-up

The primary statistical analysis follows the intention to treat principle as closely as possible. For a valid analysis, it is of great importance to minimise the rate of drop-outs. Therefore, in patients who do not withdraw their consent, all trial visits shall be performed as scheduled. In order to account for possible risks due to trial assessments, see section 6.5.1, 6.5.2 and 6.5.3. A subject who terminates the study for any reason (e.g. failure to satisfy the selection criteria) before first treatment with study medication is regarded a "screening failure".
In case of premature termination of therapy, it is necessary to document the date (as exactly as possible), the reason of termination and the current condition of the patient. Therefore, the eCRF section for "End of Study (ES)" has to be completed for each patient. Data entry to this eCRF page will trigger an automatic report to the responsible trial team members at the ZKS Leipzig.

The ES - eCRF section routinely contains the following data:
- Date of individual end of trial
- Reason for trial termination
- Latest patient contact
- Disease status at ES

- Date and reason of death, if applicable

6.5.1 Premature Termination of the Therapy for Individual Patients

All patients are treated for 8 cycles. Continuation of therapy after 8 cycles in case of HI-E response is at the discretion of the physician. After the primary endpoint/response assessment the therapy will be stopped if loss of HI-E (disease progression) is determined. The end of treatment visit is then performed and the patient goes into follow-up.
Regardless, the patient's treatment will be discontinued before the end of the treatment if any of the following occurs:

- Adverse events that, in the judgment of the investigator, may cause severe or permanent harm or which rule out continuation of study drug - judgement after consolidation with the coordinating investigator
- Major violation of the study protocol, which after consolidation of the coordinating investigator and the sponsor affect the right and safety of patients and the integrity of collected data
- (Suspected) pregnancy (report on form “Report on the arising of a pregnancy during exposition to a trial medication” to the ZKS)
- Administrative decision by the investigator or the sponsor
- The subjects initiates treatment with prohibited medication (see section 4.1.1.2)
- If a patient is significantly non-compliant with the requirements of the protocol, thereby endangering patient’s safety
- Patient’s decision/withdrawal of consent (e.g., patient declines further treatment)
- Physician decision if continuation is not in the patient’s best interest
- Termination of the study by the Sponsor

The investigator will discuss with the subject the most appropriate way to ensure the subject’s health. Any subject who has received at least one dose of the study medication should undergo an EOT visit for the evaluation of treatment response.
With exception of the rules described above, premature termination of therapy should be avoided. In case the patient misses the scheduled visits, the investigator may contact the patient directly, in order to motivate him/her to continue.
All further trial visits will take place as planned and described in section 6. **Termination of trial therapy does not mean that the patient is off-trial.**

6.5.2 Premature trial termination for individual patients

All enrolled patients are planned to receive eight cycles of canakinumab (i.e. about 6 months of therapy) followed by about 6 months of follow-up after EOT. If a subject receives less than 8 cycles of canakinumab and undergoes EOT earlier than planned, the 6 months follow-up starts immediately after the premature EOT visit. Premature termination of trial therapy does not necessarily lead to individual trial termination (see chapter 0 for explanation).
The only circumstances in which a premature trial termination (i.e. no further trial visits) in an enrolled patient is unavoidable are:

- A patient is worsening (and not responding to treatment) that contraindicates permanently participation in any trial visits
- Withdrawal of informed consent
- Complete loss of contact to the patient
- Death of the patient.

6.5.3 Premature Termination of the Follow-up for Individual Patients

Follow up will be prematurely terminated in case of loss of contact or death of the patient.

6.6 Plan for Further Treatment

With the premature enrollment stop, two transfusion-free patients with stable Hb are currently undergoing therapy as clinical responders. Novartis will continue to provide the medication free of charge to these patients within the CANFIRE trial as long as they benefit from it. Patients will be treated up to loss of response.

Patients who are still on treatment but have not yet reached the primary endpoint visit will continue to be treated in accordance with the protocol until primary endpoint visit, unless there are reasons for an early end of treatment. In the event of a clinical response, Novartis will also provide medication for these patients accordingly.

7 ADVERSE EVENTS (AE/SAE)

7.1 Adverse Events (AE)

7.1.1 Definition

An Adverse Event (AE) is any untoward medical occurrence in a patient or clinical investigation subject administered a pharmaceutical product and which does not necessarily have to have a causal relationship with this treatment (ICH Guideline E2A).
Adverse Events encompass any unfavourable and unintended sign (including an abnormal laboratory finding, for example), symptom, or disease that arise newly or worsen after the inclusion of the patient into the trial.

7.1.1.1 Expected Adverse Events

Expected adverse events are for example (according to SmPC of May 2021 – please always also check against the current version and see also 5.5.3):

- Infections (respiratory tract infections, ear infection, cellulitis, gastroenteritis, urinary tract infection, vulvovaginal candidiasis)
- Nervous system disorders (dizziness, vertigo)
- Gastrointestinal disorders (upper abdominal pain, gastro-oesophageal reflux disease)
- Skin and subcutaneous tissue disorders (injection site reaction)
- Musculoskeletal and connective tissue disorders (arthralgia, musculoskeletal pain, back pain)
- General disorders and administration site conditions (fatigue/asthenia)
- Changes in blood analyses (decreased creatinine renal clearance, proteinuria, leukopenia, neutropenia, decreased platelet count)

All details are listed in the current SmPC, as part of the ISF.

7.1.1.2 Events not to be treated as Adverse Events

- Preplanned interventions are not considered AE's, if not defined otherwise (e.g., as a result of overdose)
- Medical or elective surgical procedure (scheduled in advance), e.g. surgery, endoscopy, tooth extraction, transfusion that are not associated with the indication under study. However, the event leading to the procedure is an AE. If this event is serious, the procedure must be described in the SAE narrative. In the case of a transfusion received that is not associated with the indication under study it should be documented in the eCRF (transfusion log) by indicating the specific reason for transfusion.
- Pre-existing disease or medical condition prior to the screening visit that does not worsen
- Situations in which an adverse event change did not occur, e.g. hospitalizations for cosmetic elective surgery or for social and/or convenience reasons.
  In addition, the following reasons for hospitalizations are not considered AEs, and therefore not SAEs:
    - A standard procedure for protocol therapy administration. However, hospitalization or prolonged hospitalization for a complication of therapy administration will be reported as an SAE.
    - Routine treatment or monitoring of the studied indication not associated with any deterioration in condition.
    - The administration of blood or platelet transfusion as routine treatment of studied indication.

- o A procedure for protocol/disease-related investigations (eg, surgery, scans, endoscopy, sampling for laboratory tests, bone marrow sampling). However, hospitalization or prolonged hospitalization for a complication of such procedures remains a reportable SAE.
- o Hospitalization or prolongation of hospitalization for technical, practical, or social reasons, in absence of an AE.
- o A procedure that is planned (ie, planned prior to start of treatment on study); must be documented in the source document and the eCRF. Hospitalization or prolonged hospitalization for a complication remains a reportable SAE.
- o An elective treatment of or an elective procedure for a pre-existing condition, unrelated to the studied indication that has not worsened from baseline.
- o Emergency outpatient treatment or observation that does not result in admission, unless fulfilling other seriousness criteria above.

7.1.2 **Adverse Drug Reaction (ADR)**

An adverse drug reaction of a medicinal product **carrying a marketing authorization** is defined in Article 2(n) of Directive 2001/20/EC as all “untoward and unintended responses to an investigational medicinal product which occurs at doses normally used”.

7.1.3 **Documentation and Reporting**

All adverse events will be documented on special AE-forms from start of treatment/first injection (day 1, cycle 1) until FU/ES (= 56 ± 14 days after EOT visit).

At each visit, the patient will be asked a non-leading question such as “Have you had any health problems since the last visit”. All AEs reported in response to questioning, as well as AEs reported spontaneously will be recorded on the ‘adverse event’ page(s) in the CRF, regardless of causality. In addition, the patient will be trained to inform the clinical trial site of any health problems arising between visits by phone or personal visit.

If an AE fulfils any of the criteria for an SAE (see chapter 7.4.1 for SAE-definition), both the AE pages of the CRF **and** the SAE form must be completed. This applies to all SAEs, **whether or not they are considered to be related to trial treatment.**

For both serious and non-serious AEs, documentation should be supported by an entry in the patient’s heath record. Required information in the patient’s health record should include:

- type of AE
- grade/severity/intensity
- seriousness (see also section 7.4)
- onset date
- end date
- actions required
- outcome
- assessment of its relationship to trial drug

All abnormal physical and/or laboratory results which are considered to be clinically relevant by the investigator should be recorded as AEs. If an abnormal laboratory result meets any of the criteria for a SAE, this must also be reported on the SAE Form.

Adverse Events are classified by their seriousness, intensity/severity and relationship to the IMP (see also 17.1)

7.2 Safety Analysis

All adverse event that occurred at or after first application of canakinumab will be used for the safety analyses. Safety analysis is carried for all patients having received at least one dose of IMP.
Following metrics will be calculated:

- Number and percentage of patients with at least one adverse event
- Absolute number of all adverse events
- Minimum, maximum and mean number of adverse events per patient
- Number and percentage of patients with at least one serious adverse event
- Absolute number of all serious adverse events
- Minimum, maximum and mean number of serious adverse events per patient
- Number and percentage of patients with at least one (serious) adverse event for which relatedness to the study drug cannot be excluded
- Minimum, maximum and mean number of (serious) adverse events for which relatedness to the study drug cannot be excluded
- Absolute numbers and percentages of patients with adverse events will be tabulated by CTCAE grade
- Absolute number and percentages of patients with adverse events of grades 3 to 5 will be tabulated by CTCAE grade

7.3 Concomitant Diseases

Concomitant diseases will be recorded at screening and confirmed at the first regular trial visit before first canakinumab administration (day 1 of cycle 1). Any worsening of concomitant diseases will be regarded as adverse event

7.4 Serious Adverse Events/Serious Adverse Reactions (SAE/SAR)

7.4.1 Definition

An Adverse Event/Reaction is defined to be serious according to ICH-Guideline E2A, if it

- **results in death**,
- **is life-threatening**,
  Note: The term "life-threatening" in the definition of "serious" refers to an event in which the patient was at risk of death at the time of the event; it does not refer to an event which hypothetically might have caused death had it been more severe.
- **requires in-patient hospitalisation or prolongation of existing hospitalisation[14]**,
- **results in persistent or significant disability/incapacity or**
- **is a congenital anormal/birth defect**.

Medical and scientific judgement should be exercised in deciding whether expedited reporting is appropriate in other situations, such as important medical events that may not be immediately life-threatening or result in death or hospitalisation but may jeopardise the patient or may require intervention to prevent one of the other outcomes listed in the definition above. These should also usually be considered serious.
If an AE is considered serious, both the AE page of the eCRF and the SAE Report Form must

[14] In general, hospitalisation means that the patient stays (usually at least an overnight stay) at the hospital or emergency ward for observation and/or treatment. Complications that occur during the stay are AEs. If a complication prolongs hospitalisation or fulfills any other serious criteria, the event is serious.
Hospitalisation for elective treatment of a pre-existing condition that did not worsen from baseline is not considered an AE.

be completed.

7.4.2 Documentation and Reporting Obligations: INVESTIGATOR

Serious Adverse Events have to be documented on the SAE-forms and the investigator must report them to the sponsor immediately. If more information about the SAE becomes available later, it must also be reported to the sponsor immediately.
All SAEs will be documented on special SAE-forms from start of treatment/first injection (day 1, cycle 1) until FU/ES (= 56 ± 14 days after EOT visit).
In the event of a patient's death, the investigator/deputy or the authorised medical staff provides the leading ethics committee(s), all involved ethics committees in multi-centred trials, the responsible federal authorities and the sponsor with all further information needed to fulfil their tasks **upon request**.
In all the reports, personal data are to be pseudonymized by using the patient's identification code. It must be possible to relate the initial and all follow-up reports to each other by means of the patient identification number.
The investigator/the deputy or the authorised medical staff must report every Serious Adverse Event (incl. death not causally related to using the trial drug) as soon as getting knowledge of it to the following address:

ZKS Leipzig/Pharmacovigilance
Universität Leipzig
Zentrum für Klinische Studien Leipzig
Haertelstr. 16-18, 04107 Leipzig
Phone: +49/341/97-16129
E-mail: pharmacovigilance@zks.uni-leipzig.de

**Fax: +49/341/97-16278**

The ZKS will inform Novartis as soon as becoming aware of any SAE, SUSAR, drug exposure during pregnancy and reports of IMP misuse or abuse including initial and follow up reports by e-Mail: ams.novartis@novartis.com.

7.4.3 Documentation and Reporting Obligations: SPONSOR

After the ZKS Leipzig receives the SAE, it is immediately passed on to the coordinating investigator/responsible person for medical assessment.
The coordinating investigator/responsible person forms a second medical opinion of the SAE with respect to causal relationships and the decision as to whether or not it was expected, as described in chapter 17.1.3 and 17.1.4 and forwards the second opinion to the ZKS Leipzig within two days of its arrival.
In the ZKS Leipzig the SAE data are entered into the SAE database immediately and the MedDRA coding takes place simultaneously.
Then forwarding as per law and as described in Chapter 7.6 only for Suspected Unexpected Serious Adverse Drug Reactions (SUSARs) takes place.
Details of the sponsor's documentation and reporting obligations will be specified in a special, trial-specific pharmacovigilance plan, which will be written and finalised alongside with this protocol, if possible.

7.5 Periodic Reports

7.5.1 Annual Safety Report

According to German Medicinal Products Act the sponsor writes a safety report annually

(Annual Safety Report, ASR) and sends this report to the leading ethics committee and the federal authority. A copy of the ASR is also provided to NOVARTIS.
The key date is the date of the first authorization of the clinical trial by a federal authority. All data obtained up to this date (each year) will be included in the ASR. Beginning with the key date, there is a time-limit of 60 days for the preparation and submission of the ASR.
The ASR will be prepared by the ZKS Leipzig (project manager, responsible biometrician and PV data manager) acc. to the national requirements. Deadlines regarding the ASR are based on the German Medicinal Products Act.
The final report will be released in co-operation with the coordinating investigator/sponsor's representative.

7.6 Suspected Unexpected Serious Adverse Reactions (SUSAR)

7.6.1 Definition

Suspected Unexpected Serious Adverse Drug Reactions (SUSARs) are side-effects (probably or definitely connected with the administration of the investigational product), the nature or severity of which are inconsistent with the information available about the product. Information about the trial product is contained in the IB.

7.6.2 Documentation und Reporting Obligations

Information for SPONSOR

The sponsor submits all information available about a SUSAR immediately to the leading ethics committee, the responsible federal authority, and to all participating primary investigators, at the latest within 15 calendar days after the event becomes known - according to national law.
For every SUSAR that results in death or a life-threatening condition, the leading ethics committee, the federal authority and all participating investigators must be informed by the sponsor within 7 calendar days after the event becomes known – according to national law. Additional information has to be given within 8 further calendar days.
Details of the sponsor's documentation and reporting obligations will be specified in a special, trial-specific pharmacovigilance plan, which will be written and finalised alongside with this protocol, if possible.

Information for INVESTIGATOR

The investigator passes down all relevant information concerning the SUSAR to all participating trial investigators at his/her trial centre. This has to be confirmed by the investigator in writing.

7.7 Other Safety Relevant Issues

Other safety issues also qualify for expedited reporting where they might substantially alter the current benefit-risk assessment of an investigational medicinal product and/or would be sufficient to consider changes in the investigational medicinal product administration and/or in the overall conduct of the trial, for instance:

New events related to the conduct of the trial or the development of the IMP likely to affect the safety of subjects, such as:

- a serious adverse event, which could be associated with the trial procedures and which could modify the conduct of the trial,
- a significant hazard to the subject population such as lack of efficacy of an IMP used for the treatment of a life-threatening disease,

- a major safety finding from a newly completed animal trial (such as carcinogenicity),
- a temporary halt of a trial for safety reasons if the trial is conducted with the same investigational medicinal products in another country by the same sponsor

The sponsor decides if the number of events or qualitative changes in the expected SARs comprise a safety issue and must be reported.

7.8 Therapeutic Procedures

If a patient requires treatment as a result of an Adverse Event, then it must meet the recognized standards of medical care in order to restore the patient’s health. Appropriate resuscitation devices and medication must be available in order to treat the patient as quickly as possible in the event of an emergency.
The action taken to treat the AE/SAE must be documented by the investigator either in the appropriate CRF and/or using additional documents.

7.9 Dealing with Pregnancy

Every pregnancy that occurs while taking part in the trial must be reported to the ZKS Leipzig by the investigator/deputy or the authorised medical staff within 24 hours of having learned of it by informal communication:

ZKS Leipzig / Pharmacovigilance
Universität Leipzig
Zentrum für Klinische Studien Leipzig
Härtelstr. 16-18, 04107 Leipzig
Telefon: +49/341/97-16129
Fax: +49/341/97-16278
E-mail: pharmacovigilance@zks.uni-leipzig.de

The ZKS Leipzig will then supply the essential forms for documentation to the investigator/deputy investigator without unduly delay. Pregnancies that occur in female trial subjects or partners of male trial subjects have to be reported by using the form: ”Report on the arising of a pregnancy during exposition to a trial medication”. Severe side effects and complications during a pregnancy as well as congenital birth defects are Serious Adverse Events by definition and therefore also have to be reported on the Serious Adverse Event form according to the reporting procedures described above.
The outcome of a pregnancy has to be reported on the form “Report on the outcome of a pregnancy during/after exposition to a trial medication”. This form will document the outcome of the pregnancy, including a spontaneous or voluntary abortion, details of the birth process, the presence or absence of congenital malformations and birth defects, maternal or foetal complications and the potential relationship to the trial drug.
Collecting data concerning the outcome of a pregnancy is only permitted if the trial subject and the partner of the male trial subject puts down her permission in writing beforehand. The collection of data on the child's health requires the prior information and consent of the guardian/parents (usually both).

8 BIOMETRY

8.1 Biometrical Aspects of the Trial Design

Within this single-arm, open label proof-of-concept trial design, we aim to show that canakinumab is safe and effective in LR MDS and MDS/MPN patients.

We focus on the short-term clinical response in hemoglobin levels and biological response in suppression of inflammation,

Responding patients will be treated for up to three years. The duration of response will be described.

8.2 End Points

8.2.1 Primary Efficacy End Point

The primary clinical endpoint "documented achievement of erythroid response (HI-E)" is based on internationally accepted and validated IWG 2018 response criteria[1] and defined as an increase in hemoglobin levels by at least 1.5 g/dL, persistent for at least 8 weeks over the baseline hemoglobin level (mean over 16 weeks prior to inclusion) for NTD patients or no transfusions within a 8 week-window for LTB and HTB (major response), and will be determined after 8 cycles of canakinumab treatment.

Implementing IWG 2018 Hb-response criteria in a clinical trial requires some modifications. Mostly, IWG 2018 rules are only specified further to make them technically operational, unambiguous, and to handle data problems expected in real world trial data.
The main deviation from IWG 2018 is the introduction of so-called sliding 8 week time windows over which Hb-measurements can be averaged not only for the determination of the baseline Hb-level, but also to determine Hb-response for NTD patients. This deviation makes the response definitions statistically robust and markedly increases the power to detect a true response (Hasenclever et al in preparation).

For an outline see Appendix 8.9 and for further details see the statistical analysis plan.
In determining the response rate, patients who do not complete the treatment or are not assessable for response after treatment count as failures.

Spontaneous persisting hemoglobin level increases without therapy in anemic LR MDS/CMML are extremely rare (<< 5%). We set out our study design to show that the response rate with canakinumab is above 20%.

8.2.2 Secondary Efficacy End Point

Duration of response is only defined for responding patients. The definition follows the IWG 2018 recommendation.
NTD patients: Date of response is the date of the first measurement 1.5 g/dL above baseline hemoglobin level that starts the 8 week period that confirms the response (see 8.2.1).
The response ends as soon as the Hb-mean in an 8 week sliding window is < 1.0 g/dL above the baseline Hb level or a transfusion is given after start of response. The response ends on the day of the last Hb measurement in the 8 week sliding window that documents the end of the response or on the day of the first past response transfusion (whichever comes first).

The end date of the response is the date of the Hb measurements that terminates the response.

LTB and HTB patients:
Major response is documented if there were no transfusions given in a sliding 8-week window provided that the Hb measurement to which the sliding window was assigned was above the stated Hb trigger and was not followed by a transfusion. The condition on the Hb values prevents postponing a transfusion on purpose to achieve a formal response. The transfusion response ends on the day of the first transfusion given after response is documented.

Minor response in LTB and HTB is documented if transfusion rate ratio is ≤ 0.5 in a sliding 8-week window (response window transfusion rate vs. baseline transfusion rate).

8.2.3 Secondary Biological End Point
No formal secondary endpoints specified.

8.2.4 Safety End Points
The Safety profile of canakinumab will be described by collecting AE and SAE information.

8.3 Statistical Description of the trial hypothesis

8.3.1 Statistical Hypotheses
The null hypothesis is that the HI-E response probability is ≤ 5%.

The (general) alternative hypothesis is that the HI-E response probability is >5%.

8.4 Sample Size Discussion

The study is designed assuming a specific alternative hypothesis that the HI-E response probability is ≥20%.
Using a one-sided significance level of 2.5% and requiring a power of 85% leads to a sample size of N=41 with the exact binomial test. We need to observe at least 6 out of 41 responses to reject the null hypothesis.
The denominator in estimating the HI-E response probability consists of all included patients that started treatment. If a patient is formally included and drops-out before start of treatment, the patient will be replaced.

With the early stop of enrolment, unfortunately only anecdotal evidence will result from the trial.

8.5 Statistical Methods

8.5.1 Analysis Population
The full analysis set consists of all included patients that started treatment.

8.5.2 Planned Methods for Analysis
For the primary clinical endpoint the exact binomial test will be used. The HI-E response rate will be estimated with an exact two-sided 95% confidence interval. Time courses of HB measurements and transfusions will be plotted for all patients. Duration of response will be

illustrated by a Kaplan-Meier plot. The median duration of response will be estimated with a two-sided 95% confidence interval.

Further biological parameters as well as safety data will be analysed descriptively.
No formal interim analysis is planned.

Due to the early stop of enrollment, analysis becomes descriptive, essentially listing relevant data for the patients.

8.6 Statistical Monitoring

The trial conduct will be closely supervised by means of central and statistical monitoring according to ICH E6(R2) [4]. The objectives are

- to detect safety relevant signals as soon as possible
- to detect non-compliance and relevant protocol violations and to prevent their future occurrence by prompt reaction
- to prevent missing visits or measurements by prompt reminders

Therefore, the following issues will be monitored:

- With regard to safety
    - Patients with treatment discontinuation due to adverse events
    - Serious adverse events
- With regard to protocol compliance
    - Patients with major or minor findings concerning the informed consent documents as detected by on site monitoring
    - Patients with treatment discontinuation
    - Patients with other protocol violations as detected e.g. by on site monitoring
- With regard to missing information
    - Missing visits or visits outside schedule

This is not an exclusive list. Statistical monitoring will be continuously adapted in response to new problems/risks which might arise during trial implementation.
Detailed information about the statistical and central monitoring incl. processes, content, endpoints, data to observed, time lines, reports and descriptive statistics will be given in a central monitoring plan.

8.6.1 Endpoints for Statistical Monitoring

- Rate of patients that are formally included, but fail to start treatment.
- Time to treatment discontinuation
- Patients with insufficient Hb measurement or transfusion history to evaluate HI-E response

8.7 Interim Analysis

No formal interim analysis is planned.

8.8 Final Analysis

The final analysis is split into two time points. The analysis of the primary efficacy endpoint and the further endpoints up to response assessment will be performed when all patients are documented up to the primary endpoint visit.

The analysis of the response duration and the time course of further endpoints in responding patients will be performed as soon as all responding patients either have lost their response or were treated for three years.

8.9 Modification of the revised IWG 2018 hematological response criteria

Implementing IWG 2018 Hb-response criteria in a clinical trial requires some modifications. Mostly, IWG 2018 rules are only specified further to make them technically operational, unambiguous, and to handle data problems expected in real world trial data.
The main deviation from IWG 2018 is the introduction of so-called sliding time windows over which Hb-measurements can be averaged not only for the determination of the baseline Hb-level, but also to determine Hb-response for NTD patients. This deviation makes the response definitions statistically robust and markedly increases the power to detect a true response (Hasenclever et al. in preparation).

8.9.1 **Baseline evaluation period**

Patients enter the CANFIRE trial in two steps:

- Registration for the study with informed consent, preliminary check of inclusion/exclusion criteria, and initiation of study-specific screening investigations during a screening period. (*CAVE: Patients should only be registered if valid baseline data is to be expected at the end of the screening period*)
- Formal inclusion with a final check of inclusion and exclusion criteria after completion of all study-specific screening investigations at the end of the baseline evaluation period and shortly before start of treatment.

The baseline evaluation period is defined relative to the day d0 of formal inclusion and overlaps with the screening period.
Baseline evaluation period consists of the days -112 to 0 (inclusive; last 16 weeks). In order to accommodate pragmatically for delays in formal inclusion after meeting the inclusion criteria for up to a week or in order to fulfill the validity criteria stated below an extension of the baseline evaluation period up to days -130 to 0 is tolerated.
Patients should be registered for the trial and start study-specific investigations only, if they fulfill the preliminary inclusion/exclusion criteria, which include that valid baseline data are expected to be available at the projected end of the baseline evaluation period.

Specification of minimum data required for the whole baseline evaluation period:

- A complete transfusion history during the whole baseline evaluation period must be documented before inclusion into the trial. For all transfusion episodes, this includes calendar date, the Hb-value preceding the decision to give a transfusion, and the number of RBCs given.
- *Ideally, there should be a uniform transfusion strategy applied during the whole baseline evaluation period.* The transfusion trigger threshold characterizing the transfusion strategy (e.g. < 8 g/dL) must be documented at registration and must be applied afterwards.
    - If transfusions were given during the baseline period, the Hb values that triggered the transfusions must be consistent with the stated transfusion trigger threshold. Only then is the transfusion burden before and after start of treatment are comparable.
    - A Hb-value just before a transfusion which lies outside the interval [transfusion trigger threshold ± 1.0 g/dl] generates a query and must be explained or justified clinically.

8.9.2 **Calculation of baseline Hb level**

Baseline Hb level will be calculated for NTD as well as transfusion dependent cases from all available and valid Hb measurements from the baseline evaluation. All Hb measurements must

be documented in the trial database before inclusion into the trial.
All measurements taken less than 7 days after a transfusion are discarded from the Hb history as invalid for NTD patients. Operationally, if a transfusion was given on day d, Hb-values taken after the transfusion from day d up to day d + 6 inclusive are discarded. For transfusion dependent cases, all Hb-values triggering a transfusion are retained. In case there was a transfusion on the same day as a blood sampling, it must be documented whether the Hb measurement was before or after the transfusion. The (remaining) Hb history is valid if the mean is based on at least 5 Hb measurements that spans at least 100 days. In addition, the baseline Hb level is defined as the weighted mean over all valid Hb measurements in the Hb history. To eliminate the bias potentially introduced when Hb-measurements are less than 7 days apart, weights to each Hb value are assigned in a given time window and weighted mean for averaging of time windows are used.

8.9.3 **Calculation of the baseline transfusion count**

The baseline RBC transfusion count (R) is the number of RBC-transfusions documented in the baseline evaluation period. Multiple RBCs can be given in a single transfusion episode.
Based on the RBC transfusion count, patients are classified as:

- non transfusion dependent (NTD): R ≤ 2 RBCs,
- low transfusion burden (LTB): 3 ≤ R ≤ 7 RBCs, and
- high transfusion burden (HTB): R ≥ 8 RBCs

8.9.4 **Sliding windows to calculate Hb means**

Repeated Hb measurements in a steady state fluctuate naturally with a certain standard deviation SD about their true underlying (unknown) mean. Based on available Hb time course data, we estimate that SD may be in the order of SD = 0.4 to 0.5 g/dL when using the same measurement method. If different methods to measure Hb are used, SD may be even larger.
Simulations show that the IWG 2018 response criteria are statistically instable with such chance fluctuations. Therefore, we use the mean over at least the four most recent Hb values in a sliding window of at least 8 weeks to define response.
We assign a **sliding window of at least eight weeks** to each Hb measurement taken more than 8 weeks after start of treatment. For a sliding window to be valid, we require that the sliding window contain at least 4 valid Hb measurements (in order to reduce the standard error of the mean to at least SD/2) and that the included Hb measurements span at least 50 days (in order represent an 8-week time period as required by IWG 2018). If an eight-week window contains less than 4 Hb measurements, it can be extended to maximally 12 weeks to fulfill this condition.

8.9.5 **Hb Response assessment in NTD patients**

HI-E response in NTD patients is defined algorithmically as follows:
Calculate Hb means for all valid Sliding Windows assigned to the time course of Hb measurements.

- A HI-E response in NTD patients is achieved as soon as the HbMean in a sliding window is ≥1.5 g/dL above the baseline Hb level without intermittent transfusions.
- The response starts on the day of the last Hb measurement to which the response defining sliding window was assigned.
- The response ends as soon as the HbMean in a sliding window is < 1.0 g/dL above the baseline Hb level or a transfusion is given after start of response.
- The response ends on the day of the last Hb measurement to which the sliding window was assigned that documents the end of the response or on the day of the first past response transfusion (whichever comes first).

8.9.6 Response in LTB and HTB patients (response in transfusions)

At registration, investigators have committed to a transfusion trigger threshold for the registered patient which should be applied from registration until the end of the study.
For LTB and HTB patients, the Hb time course must be documented too. We assume and require that there always be an Hb value before any transfusion episode.
Hb must be regularly measured such that compliance with the stated Hb trigger is documented. Response in transfusion RBC count is only meaningful if it is documented that the stated transfusion strategy was adhered to.

In order to determine response in LTB and HTB, we look at sliding 8-week windows assigned to Hb measurements as defined above for NTD.
An Hb response in LTB and HTB is documented if no transfusions were given in a sliding 8-week window provided that the Hb measurement to which the sliding window was assigned was consistent with the stated Hb trigger and was not followed by a transfusion in the next two days.
The condition on the Hb values prevents postponing a transfusion on purpose to achieve a formal response.
It is clinically implausible – in particular for HTB - to define the end of response (suggesting stop of treatment) by the first transfusion given after documented Hb-response.
In order to define the end of Hb-response, we adapt the concept of minor response mentioned in IWG 2018 for HTB only.
At baseline, we determine the baseline transfusion rate as number of RCBs divided by length of the baseline window. Similarly, there is a transfusion rate for each response window.
We define the transfusion rate ratio as the ratio of the transfusion rate in the response window divided by the baseline transfusion rate. We use transfusion rates instead of transfusion counts because the width of the time windows varies depending on availability of data.

The Hb-transfusion response ends on the last day of the first response window after a documented response that has response transfusion rate ratio > 0.5, i.e. inconsistent with at least a minor response.

9 CONCOMITANT SCIENTIFIC PROJECTS

The canakinumab trial will be accompanied by translational studies investigating the cellular and molecular consequences of IL-1β blockade in MDS patients, based on ongoing preclinical studies of the effects of antibody treatment *ex vivo* ("Efficacy of bi-specific antibody MAS825 to block inflammasome circuit in lower-risk MDS"). This will provide an ideal opportunity to validate and analyse candidate biomarkers in longitudinal *in vivo* studies using paired patient samples.
Detailed analyses of the baseline and treatment-dependent changes in inflammasome activity and erythropoiesis should allow the identification of biomarkers that both predict and describe the clinical response of LR-MDS patients to canakinumab.

Panels for gene expression analysis
The inflammasome profile in patient bone marrow will be assessed by qPCR (TaqMan® Assay) of a panel of inflammasome-related proteins (*NLRP3*, *ASC/PYCARD*, *CASP1*, *IL1β*, *IL18*). Where inflammasome expression is high, we will extend the expression studies to include other canonical inflammasome proteins capable of activating caspase-1 (*NLRP1*, *NLRC4*, *AIM2*) as well as non-canonical inflammasome signaling (*CASP4/5*) in order to identify the likely route of inflammasome activation in each patient.

Measuring circulating inflammatory markers
Patient-specific inflammatory profiles will also be assessed before and after treatment at the protein level by Luminex or ELISA immunoassay of secreted inflammatory inducers and mediators in bone marrow (including e.g. S100A9, IL-1β and IL-18).
The mRNA and protein expression data will be used to define patients with high and low inflammasome activity, and potentially to distinguish between subgroups with distinct features of inflammasome expression. Comparisons to clinical data will then be made to reveal inflammasome-dependent differential responses to therapy.
In addition, the levels of S100A9 and IL-1β in peripheral blood will be monitored by ELISA at multiple time points during therapy in order to describe the kinetics of inflammatory-suppression during treatment and to investigate correlations to response and outcome.

Influence of canakinumab on erythroid populations
Changes in erythroid response rate may result from changes in the proliferation and differentiation of erythroid progenitors or maturation and stability of erythrocytes. In order to identify the level at which erythropoiesis is affected by canakinumab-treatment, we will firstly use Flow Cytometry analysis (CD36/CD45/CD71/CD117/CD235ab) of bone marrow cells to scan the erythroid hierarchy before and after treatment, and secondly introduce bone marrow MNCs into colony assays to reveal the numbers of functional progenitors at the CFU-GEMM, BFU-E and CFU-E stages of differentiation.

Mutation analysis
Bone marrow MNCs/PBMCs of patients before and after treatment will be subjected to an established, comprehensive mutation analysis of a panel of mutations associated with myeloid malignancies. The identity and mutational burden will then be correlated with clinical features and response to canakinumab in order to identify associations between genetic subtypes, inflammatory features and susceptibility to inflammasome inhibition.

10 ETHICAL, LEGAL AND ADMINISTRATIVE ASPECTS

10.1 GCP-Statement

All persons participating in the conduct of the trial (sponsor, authorised representative of the sponsor, investigators, etc.) commit themselves to observe the Declaration of Helsinki of the WMA (in its current version), as well as all pertinent national laws and the ICH guideline for Good Clinical Practice (GCP) ICH E6(R2) (EMA/CHMP/ICH/135/1995) issued in June 2017.

10.2 Initial Submission

10.2.1 Submission to the Ethics Committee(s) and Competent Authority

Prior to submitting the trial related documents to the leading (and involved) ethics committee(s) and the responsible competent authority, the sponsor must enter the trial into the European database for clinical trials (EudraCT).
Afterwards, the protocol and all other associated documents according to GCP-V § 7 will be submitted to the leading ethics committee for approval. Parallel to the submission to the leading ethics committee (EC), each participating EC is informed of the submission and also receives a copy of the documents including those of the trial sites, which they have to approve. At the same time the study documents will be submitted to the responsible competent authority according to the requirements of GCP-V § 7[4]/the applicable national requirements.
The trial can start only after obtaining a positive review by the leading ethics committee and approval from the responsible federal authority. Both written approvals must be filed in the trial master file (TMF). Additionally, every participating centre must receive a copy of these documents to be filed in the investigator site file (ISF).

10.3 Protocol Amendments

Changes to an approved trial protocol have to be submitted to EC and CA and positively appraised before implementation of the changes, if the changes are such that they may affect the subjects' safety, e.g.

- fundamental changes to the therapeutic procedures
- Updates to the SmPC that may have an impact on the assessment of adverse events, including the definition of possible SUSARs.
- result in further data collection that requires changes to the patient information and/or informed consent form,
- affect the interpretation of the scientific documents upon which the trial is based or the significance of the results of the trial,
- significantly affect the leadership or conduct of the trial,
- concern the quality or the innocuousness of the investigational drug, or
- in clinical trials with drugs containing genetically modified organism affect the risk-benefit considerations for the environment at large.

Changes to the protocol may only be performed by the sponsor, the authorised representative or the coordinating investigator in co-operation with the biometrician.
After approval of the trial changes, all participating trial sites have to be informed about the changes in writing and supplied with potentially changed documents.
The whole process has to be documented in the Trial Master File (TMF).

11 DOCUMENTATION

11.1 General information and Access Rights

In the context of a database for electronic data capture only, the Case Report Form (CRF) will be designed by the ZKS Leipzig in cooperation with the sponsor/sponsor's authorised representative/Coordinating Investigator and provided as electronic form (eCRF). In order to facilitate the documentation as per protocol in case of malfunction of the electronic system or any of its components, a paper version of the CRF (interim CRF) will be provided in the ISF (investigator site file). The data on this paper version will be transferred to the eCRF as soon as the electronic system is available again.
Special CRF forms will be provided as paper CRF:

- **SAE-forms**, because these have to be sent to the ZKS Leipzig in a printable version for submission to the relevant authorities
- **Patient questionnaires** (QUALMS and EORTC-C30) since these will be filled in by the patient directly/ will be completed by authorized staff members together with the patient.

An eCRF will be provided for each patient. The patient will be identified as per patient-ID only. The eCRF documentation must be completed timely after each trial visit according to ICH Guideline E6 chapter 4.9.1, in order to enable central monitoring of the trial data.

Access to the data base will be limited to authorised staff only. Authorisation is granted by the site's investigator using the trial specific staff signature and delegation log. Based on the staff signature and delegation log access to the eCRF will be granted by the responsible staff at the ZKS Leipzig.
Authorised staff members on site will be able to enter and update data as well as finalise data by electronic signature during the conduct of the trial according to a trial specific concept for documentation. This concept is based on the internal Standard Operating Procedures implemented by the ZKS Leipzig and follows the ICH Guideline E6. All entries and data changes will be tracked automatically including date, time and person who entered/changed information (audit trail). Major correction(s) or major missing data have to be explained.

11.2 Patient File and Source Data

All information required by the protocol and therefor collected during the clinical trial must be recorded by the Investigator or an authorised member of the trial team as source data in the source documentation for the trial (e.g. patient file).
Source data according to ICH Guideline E6 are defined as any information in original records and/or certified copies of original records of clinical findings, observations, or other activities in a clinical trial necessary for the reconstruction and evaluation of the trial. Source data are contained in source documents.
The Source Data Agreement is defining source data and their location for respective CRF entries. It will be filled in at the initiation visit, signed by the investigator or deputy investigator and filed in the trial master file.

In order to confirm the completeness, accuracy and consistency of the trial data with the data in the source documents the investigator or deputy investigator has to electronically sign each patient's CRF after his/her individual end of trial participation.

11.3 Data Management

For creation of the trial database the EDC tool secuTrial®, developed and distributed by interActive Systems GmbH (iAS), will be used. The CDASH-compatible database will be validated according to the Standard Operating Procedures (SOPs) of the ZKS Leipzig prior to data capture.
The information entered into the eCRF by the investigator or an authorised member of the trial team is systematically checked for completeness, consistency and plausibility by routines implemented in CDMS such that discrepancies can be dealt with at data entry. Errors and warnings are listed in a validation report and can be resolved at any time during entry process. On completion of the data entry the site staff flags the eCRF-pages as 'data entry completed' (DEC).
During on-site monitoring or central/statistical monitoring, the monitor or the data manager at the ZKS Leipzig may create a manual query for discrepancies that are identified after DEC. All eCRF-pages with queries are marked in the system and a report with all queries listed is available. The site staff is responsible for data correction and can resolve queries directly in the eCRF-page.
The ZKS Leipzig will supervise and support the solution of queries and will close all correctly resolved queries. In case a query cannot be solved, the data management staff may close the query in agreement with the trial biometrician.
In case of self-evident corrections by data management staff, a list of self-evident corrections or data entry conventions (e.g. for data entry of paper-based life questionnaires) will be available and authorised by the coordinating investigator or the responsible biometrician of the clinical trial. Each trial site will receive a list of performed SECs at the end of the trial.
During the whole course of the trial, a backup of the data is made on a daily basis according to the backup policies of the ZKS Leipzig. Unauthorised access to patient data is prevented by the access concept of the trial database, which is based on a strict hierarchy and role concept. Any change of data (e.g. when data is changed in the database during query management) is recorded automatically via audit trail within the database.

At the end of the trial, once the database has been declared complete and accurate, the database will be locked. Thereafter, any changes to the database are possible only by joint written agreement between sponsor/sponsors authorised representative/coordinating investigator, biometrician and data manager.

11.4 Archiving

All relevant trial documentation (Trial Master File), the electronically stored data, the original CRFs and the final report will be stored for at least 10 years by the sponsor after the regular or premature end of the trial.
At the investigating sites, the investigators' files, patient identification lists, signed written consent forms, copies of all CRFs and the patients' files will be stored for at least 10 years after the regular or premature end of the trial. Local rules or other legal requirements (e.g. Strahlenschutzgesetz) are to be met if they require longer periods of archiving.
If the clinical trial is being conducted in order to gain approval for a trial drug, then the requirements of the Arzneimittelprüfrichtlinie will be adhered to, which state that the patient identification lists are to be stored for up to 15 years after the trial's completion/termination.

12 REFERENCE EVALUATIONS

12.1 Central morphology evaluation in Düsseldorf

Bone marrow analyses and CBC with differential will be performed by a central laboratory in Düsseldorf. For this purpose 5 unstained bone marrow slides and 2 peripheral blood slides should be sent to the central lab in Düsseldorf. Samples should be shipped at room temperature within a timeframe of max. 24 hours to the address provided in the lab manual (part of the ISF).

Time points for central morphology samples:

3 native unstained bone marrow slides	• Screening<br>• pEP<br>• for responders once yearly after pEP<br>• EOT
2 native peripheral blood slides:	• Screening<br>• pEP<br>• for responders once yearly after pEP<br>• EOT

12.2 Instructions for bone marrow / peripheral blood samples

Bone marrow collection as well as collection of peripheral blood samples will be performed according to the schedule of study assessments. Samples should be shipped at room temperature to the address provided in the lab manual (part of the ISF).

Time points for shipment of central biobanking samples:

bone marrow sample	• Screening<br>• pEP<br>• EOT
peripheral blood	• Screening<br>• At day 1 of each cycle during the treatment period (cycle 1-8)[15]<br>• pEP<br>• EOT
One buccal swab	• Screening

[15] Peripheral Blood sampling should be done for responding patients at least in three month interval from cycle 8 onward

13 SUPERVISION OF THE CLINICAL TRIAL

13.1 Access to Source Data

According to ICH Guideline E6 and the applicable German laws, the investigator must permit all authorised third parties access to the trial site and the medical records of the trial subjects (source data). These include the clinical trial monitors, auditors and other authorised employees of the sponsor, as well as members of the local or federal authorities. All these persons are sworn to secrecy.

13.2 Monitoring

The ZKS Leipzig will be responsible for trial monitoring. Initiation, regular and close-out visits will be performed in all trial sites. A risk-based monitoring strategy will be implemented, as required by ICH E6 R2 (Chapter 5.0).
During trial conduct, central and statistical monitoring procedures will be combined with on-site monitoring visits in order to achieve high protocol compliance and data quality, as well as to ensure patients' safety and rights. The chosen monitoring strategy depends on the results of the risk analysis done during the protocol development and will described in the trial specific monitoring plan.
In general, a first monitoring visit at a trial site will be scheduled after the inclusion of the site's first patient and documentation of the first treatment cycle, checking protocol compliance and preventing further systematic errors due to misunderstandings. All trial sites will then be visited regularly. The frequency of further on-site monitoring visits will depend on the trial site's recruitment rate and on whether problems have been detected with the site, either by prior on-site visits or by central monitoring.
Prior to every scheduled on-site visit, the monitor will be provided patient synopses summarising the data already available in the database, and indicating possible protocol deviations or inconsistencies. If deemed necessary (e. g. in case of a high number of data inconsistencies/queries), queries raised by pharmacovigilance and/or from the statistical monitoring will be communicated to the site in due time before the on-site visit, to enable a timely processing.

During the visits the monitor will:

- check informed consent forms of all patients enrolled
- perform source data verification of the key data (selected baseline parameters, therapy delivery, Hb measurements, serious adverse events, follow-up) in a random sample of the site's patients
- perform targeted source data verification for patients with possible deviations
- discuss open queries raised by data management or drug safety personnel
- check essential parts of the investigator site file (see monitoring plan)
- check source data for AEs or SAEs, which have not been properly reported in the eCRF
- check for major GCP-breaches and/or protocol violations according to the trial specific monitoring plan.

13.3 Audits / inspections

As part of quality assurance, the sponsor has the right to audit the trial site and any other institutions involved in the trial. The aim of an audit is to verify the validity, accuracy and completeness of data, to establish the credibility of the clinical trial, and to check whether the trial subject's rights and trial subject safety are being ensured. The sponsor may assign these activities to persons otherwise not involved in the trial (auditors). These persons are allowed

access to all trial documentation (especially the trial protocol, case report forms, trial subjects' medical records, drug accountability documentation, and trial-related correspondence).
In addition, inspections by regulatory health authority representatives and IEC are possible.

The investigator should notify the sponsor immediately of any such inspection. The investigator/institution agrees to allow the auditor or inspector direct access to all relevant documents and allocate his/her time and the time of his/her staff to the auditor/inspector to discuss findings and any issues. Audits and inspections may occur at any time during or after completion of the study.

All persons conducting audits will keep all trial subject data and other trial data confidential.

13.4 Data protection and Confidentiality

The sponsor, together with ZKS Leipzig and the trial sites, is responsible for the implementation and data processing in accordance with Article 4(7) of the EU General Data Protection Regulation 2016/679 in this trial. The ZKS Leipzig is responsible for implementation of procedures for data collection, storage, protection, retention and destruction. The ZKS Leipzig has implemented a data safety and security concept according to the requirements of the German Federal Office for Information Security (www.bsi.bund.de).
All data will be initially collected by investigators in the recruiting trial sites. Together with information on the trial, eligible patients will be informed about data capture, transmission, analysis processes and their rights according to the General Data Protection Regulation (GDPR). Once a patient is eligible and has given his/her informed consent to trial participation and data collection, the investigator will assign the patient a unique patient identification code. Patient identification code lists will be generated in advance by ZKS Leipzig and forwarded to the recruiting sites. These lists are part of the investigator site file and remain at the recruiting site. These lists are the only documents that allow for re-identification of the patients.
All clinical data entered by the investigators (or their designated staff) into eCRFs will be recorded in a pseudonymized form (i.e. without reference to the patient's name and date of birth) exclusively using the patient's identification code.
Clinical monitors appointed by ZKS Leipzig will regularly visit the recruiting sites and verify the informed consent forms. Data will only be used for analysis after it has been verified by monitors that the patient has unambiguously given his or her consent for trial participation as well as for data capture, transmission and analysis. The patients are informed of this fact and agree to the procedure with the patient information/informed consent.
In the event of withdrawal of consent, the necessity for storing data will be evaluated. While the General Data Protection Regulation (EU) 2016/679 strengthens personal data protection rights, encompassing the right to access, rectification and withdrawal of data, it also specifies the situations when restriction on those rights may be imposed. The withdrawal of informed consent should not affect the results of activities already carried out, such as the storage and use of data obtained on the basis of informed consent before.

13.5 Declaration regarding Data Protection

We hereby confirm that all clinical trial information will be recorded, processed, handled and stored by ZKS Leipzig, Härtelstr. 16-18, 04107 Leipzig, Germany on behalf of the sponsor.
Data captured by the investigators will be processed in such a way that it can be accurately reported, interpreted and verified while the confidentiality of records and the personal data of the subjects remain protected. Data capture and processing will be in accordance with the applicable law on personal data protection and with the General Data Protection Regulation (EC) 2016/679 of the European parliament and of the council.
Access to the data is strictly limited to authorised persons. Data are protected against

unauthorised access.

13.6 Declaration regarding the Pseudonymized Transfer of Personal Data

The sponsor certifies herewith that the transfer of pseudonymized personal data will take place according to the documentation and communication regulations in §§ 12 und 13 of the GCP-guidelines (GCP-V)[4]. Moreover, the sponsor certifies that trial participants who do not permit the transfer of data will not be admitted to the trial. This will be ascertained by including the relevant information in the patient information/informed consent.

13.7 Anonymisation of Data after the end of Archiving

After the end of the archiving period, all clinical data present at the ZKS Leipzig will be stored in an anonymous form.
All data will be subject to an anonymization process removing personalised data as far as possible, i.e. without endangering the possibility to answer scientific questions related to the trial. Anonymised data will be relocated to a separate, access restricted, file location and secondary data sources will be deleted.
Non clinical data (like contact information) will be deleted after the end of the archiving period.

14 ADMINISTRATIVE AGREEMENTS

14.1 Adherence to the Protocol

The study will be conducted in accordance with the protocol, GCP and applicable regulatory requirements.
The clinical trial described here will be conducted and analysed in accordance with local laws (AMG/GCP-Verordnung) and ICH Guideline E6 (Good Clinical Practice - GCP).
Protocol violations are all deviations from the procedures outlined in this document, e.g.

- examinations that are missed or that take place at the wrong time
- non-compliance
- intake of prohibited medication

After a patient has been enrolled, it is the investigator's responsibility to avoid protocol violations in order to obtain unbiased data for the trial.
Those protocol violations deemed to be major are defined by the risk analysis performed before and during trial implementation and will be further detailed in separate documents belonging to the risk assessment/monitoring plan. This list can be augmented in the course of the trial.
Major protocol violations will be reported to the ZKS Leipzig, which will inform the sponsor.
All protocol violations will be documented and discussed with the responsible biometrician before closing the data base and carrying out the statistical analysis.
The investigator must ensure that the recorded data are documented as per protocol. Minor variations are inevitable in clinical routine, but must be documented together with a justification.

14.2 Funding and Insurance

This study is funded by Novartis Oncology. Novartis Oncology provides funds for the conduct of the trial and provides the IMP (canakinumab).
The collection, administration, analysis and interpretation of data is the responsibility of the sponsor, as well as the preparation of the final report and the decision to publication.
The sponsor is responsible for the provision of insurance or indemnity to cover potential harm done to the patients due to the trial participation according to the national requirements and the corresponding national laws. All trial participants will be insured during trial participation by a volunteers' trial insurance to the medical faculty of the Leipzig University:
Policy number: 28 - 138971 03302

Policy holder: HDI Global SE, Eisenbahnstraße 1-3, 04315 Leipzig, Germany
Maximum sum insured: 500,000.00 €/patient
At each trial site, a copy of the policy and the general policy conditions will be filed in the ISF. The patient will receive a copy of the general policy conditions.

14.3 Notification of Local Authorities

Prior to enrolment of the first patient in the trial, the sponsor, his/her legal representatives/ contractors and all investigators and their deputies are responsible according to German Medicinal Products Act AMG § 67 (1) and the requirements of the GCP-V §§ 12 and 13 for notifying the local regulatory authority of their participation in the trial.
According to § 67 (3) AMG and §§ 12,13 GCP-V the sponsor, his/her legal representatives/ contractors and all investigators and their deputies are also responsible for notifying the local regulatory authority of amendments, premature termination of trial arms or of the whole trial and the regular trial termination.

14.4 Publication Policy and Registration

The results of this trial will be submitted for publication in a peer-reviewed, international English-language journal of appropriate aim and scope. Accordingly, the clinical trial will be registered at clinicaltrials.gov register before recruitment starts. According to the results of main and concomitant studies, the results may be submitted in separate or combined manuscripts; decisions about the form and scope of individual manuscripts will be discussed among all persons participating in the design, conduct and analysis of the trial who qualify for authorship. The coordinating investigator together with the biometrician(s) is responsible for drafting and circulating manuscripts and for discussing and handling requests by co-authors or/and sponsors to edit the text.
The authorship will follow the criteria for authorship developed by the International Committee of Medical Journal Editors (ICMJE), including those that distinguish authors from other contributors.
The ICMJE recommends that authorship be based on the following 4 criteria:

- Substantial contributions to the conception or design of the work; or the acquisition, analysis, or interpretation of data for the work; AND
- Drafting the work or revising it critically for important intellectual content; AND
- Final approval of the version to be published; AND
- Agreement to be accountable for all aspects of the work in ensuring that questions related to the accuracy or integrity of any part of the work are appropriately investigated and resolved.

All those designated as authors should meet all four criteria for authorship, and all who meet the four criteria should be identified as authors. Those who do not meet all four criteria will be acknowledged in the manuscript.
The scientific use of data resulting from this trial by local trial sites is ruled by the site contracts between the sponsor and the local trial sites. Generally, sites might use data for own scientific questions (independent from the questions discussed in this trial protocol) and publication after consultation with the sponsor.

14.5 Data Sharing Statement

According to the recommendations on data sharing by the International Committee of Medical Journal Editors (ICMJE) data resulting from the CANFIRE-trial will be made available to the scientific community as follows:
After publication of the major results and upon reasonable request from researchers performing an individual patient data meta-analysis, individual patient data that underlie published results will be shared after de-identification. This requires approval by the local

Ethics Committee/Institutional Review Board (IRB) of the researcher requesting the data along with public registration of the meta-analysis.
Summary statistics that go beyond the scope of published material will be made available to researchers for meta-analysis upon reasonable request and if the necessary data analysis is not unduly time-consuming. Together with publication of the main results, the trial protocol in full will be made publically available as well as the statistical analysis plan.

15 PROTOCOL SIGNATURES

Confirmation of the Final Protocol
We hereby certify that this is the final version of the protocol:

Coordinating investigator: Dr. Anne Sophie Kubasch	26.02.2024<br>Date	Signature
Biometrician: Dr. Jens Przybilla	27.2.24<br>Date	Signature

16 PROTOCOL AGREEMENT

Herewith I declare that I have read and understood the present protocol and agree to take into account and follow each part of it. I will ensure that all trial patients enrolled in this trial site will be treated, observed and documented in accordance with this protocol. I will ensure that all persons assisting with the trial under my supervision are adequately informed about the protocol, the investigational product and their duties.
I further declare that I do not have any financial and other competing interests in this trial.

Centre-ID	_____ - __/__/__/
Address trial site (stamp)	

Date

Signature Investigator

Date

Signature Deputy Investigator

17 APPENDIX

17.1 Classification of Adverse Events

17.1.1 Degree of Seriousness

The seriousness of an Adverse Event will be determined in accordance with the definitions in 7.1 and 7.4.

17.1.2 Assessment of Intensity/Severity

The assessment of the intensity accords with CTCAE V5.0

Mild Adverse Event	• asymptomatic or mild symptoms;<br>• clinical or diagnostic observations only;<br>• intervention not indicated.
Moderate Adverse Event	• minimal, local or noninvasive intervention indicated;<br>• limiting age-appropriate instrumental ADL*[16].
Severe Adverse Event	• medically significant but not immediately life-threatening;<br>• hospitalisation or prolongation of hospitalisation indicated;<br>• disabling;<br>• limiting self care ADL**
Life-threatening Adverse Event	• Life-threatening consequences;<br>• urgent intervention indicated
Death related to Adverse Event	

17.1.3 Determining the Causal Relationship

The investigator/ the deputy or the authorised medical staff must assess whether or not the Adverse Event is causally related to the administration of the trial medication. The following classification is to be used.

- Reasonable possibility
- No reasonable possibility

A reasonable possibility exists, if one of the following WHO-UMC criteria is met:

- occurring in a plausible time relationship to drug administration, and which cannot be explained by concurrent disease or other drugs or chemicals. The response to withdrawal of the drug (dechallenge) should be clinically plausible. The event must be definitive pharmacologically or phenomenologically, using a satisfactory rechallenge procedure if necessary.
- with a reasonable time sequence to administration of the drug, unlikely to be attributed to concurrent disease or other drugs or chemicals, and which follows a clinically reasonable response on withdrawal (dechallenge). Rechallenge information is not required to fulfil this definition.
- with a reasonable time sequence to administration of the drug, but which could also be explained by concurrent disease or other drugs or chemicals. Information on drug withdrawal may be lacking or unclear.

---

[16] **Activities of Daily Living (ADL):**
*Instrumental ADL refer to preparing meals, shopping for groceries or clothes, using the telephone, managing money, etc.
**Self care ADL refer to bathing, dressing and undressing, feeding self, using the toilet, taking medications, and not bedridden.

- more data is essential for a proper assessment or the additional data are under examination
- cannot be judged because information is insufficient or contradictory, and which cannot be supplemented or verified

No reasonable possibility exists, if the following WHO-UMC criterion is met:

- with a temporal relationship to drug administration which makes a causal relationship improbable, and in which other drugs, chemicals or underlying disease provide plausible explanations.

17.1.4 **Expected/Unexpected**

Adverse Events are unexpected if they do not occur in the manner or with the intensity described in the SmPC (see investigator's files).

17.1.5 **Outcome of an Adverse Event**

The outcome of an Adverse Event is classified as follows:

- recovered/resolved
- recovering/resolving
- not recovered/not resolved
- recovered/resolved with sequel
- fatal*
- unknown

*Note: A patient's death is not in itself an event, but the consequence of one. The event that led to the patient's death must be documented completely and reported even if death occurs four weeks after stopping medication and independent of whether or not there is a relation to the therapy or not.

17.2 Abbreviations

aCML	Atypical chronic myeloid leukaemia
AE	Adverse Event
ALT	Alanine aminotransferase
AMG	Arzneimittelgesetz (= German Medicinal Products Act)
AML	Acute myeloid leukemia
ANC	Absolute neutrophil counts
AOSD	Adult-onset Still's disease
AP	Alkaline phosphatase
ASR	Annual Safety Report
AST	Aspartate aminotransferase
BM	Bone marrow
BMMNC	Bone marrow mononuclear cell
CA	Competent Authority
CAN	Canakinumab
CAPS	Cryopyrin-associated periodic syndromes
CBC	Complete blood count
CHIP	Clonal hematopoiesis of indeterminate potential
CK	Creatine phosphokinase
CMML	Chronic myelomonocytic leukemia
CPSS	CMML-Specific Prognostic Scoring System
CRP	C-reactive protein
DAMP	Damage-associated molecular pattern
ECOG	Eastern Cooperative Oncology Group
eCRF	electronic Case Report Form
EMA	European Medicines Agency
ES	End of study
EOT	End of therapy
EORTC	European Organization for Research and Treatment of Cancer
EORTC-C30	Quality of Life of cancer patients questionnaire
EPO	Epoetin alfa
ESA	Erythropoiesis-stimulating agent
FDA	Food and Drug Administration
FMF	Familial Mediterranean fever
FPI	First patient in
FU	Follow-up
GCP	Good Clinical Practice
GCP-V	GCP-Verordnung
G-CSF	Granulocyte colony-stimulating factor
GGT	Gamma-glutamyl transferase
Hb	Hemoglobin
HCT	Hematocrit
HIDS	Hyperimmunoglobulin D syndrome
HI-E	Erythroid response
HI-N	Hematologic improvement of neutrophils
HI-P	Hematologic improvement of platlets
HR	High risk
HS(P)C	Hematopoietic stem (progenitor) cell
HTB	High transfusion burden
ICH	International Council for Harmonisation
IPSS	International Prognostic Scoring System
ISF	Investigator Site File
IWG	International Working Group
LDH	Lactate dehydrogenase

LLN	Lower limit of normal
LPO	Last patient out
LR	Low risk
LTB	Low transfusion burden
M-CSF	Macrophage colony-stimulating factor
MCH	Mean corpuscular haemoglobin
MCHC	Mean corpuscular haemoglobin concentration
MCV	Mean corpuscular volume
MDS	Myelodysplastic syndrome
MKD	Mevalonate kinase deficiency
MPN	Myeloproliferative neoplasms
MPN-RS-T	Myeloproliferative neoplasm with ring sideroblasts and thrombocytosis
MPNu	MPN-unclassifiable
NGS	Next generation sequencing
NTD	Non-transfusion dependent
PB	Peripheral blood
PBMC	Peripheral blood mononuclear cell
PEI	Paul-Ehrlich-Institut (Agency of the German Federal Ministry of Health. Its research and control activities promote the quality, efficacy and safety of biological medicinal products.)
pEP	Primary endpoint visit
PLT	Platlets
PTT	Partial thromboplastin time
QoL	Quality of life
QUALMS	Quality of Life in Myelodysplasia Scale
RBC	Red blood cell
ROS	Reactive oxygen species
SAE	Serious Adverse Event
SAR	Serious Adverse Reaction
sEPO	Serum erythropoietin
SJIA	Systemic juvenile idiopathic arthritis
SUSAR	Suspected Unexpected Serious Adverse Reaction
TRAPS	Tumour necrosis factor receptor associated periodic syndrome
ULN	Upper limit of normal
VAF	Variant-allele frequency
WBC	White blood cells
WHO-UMC	World Health Organization – Uppsala Monitoring Centre
ZKS Leipzig	Zentrum für klinische Studien Leipzig

17.3 Labelling

17.3.1 Labelling of the vial (primary packaging) and labelling of the pack (secondary packaging)

As the commercially available formulation (150mg Canakinumab (ILARIS®) in 1mL solution) is used, the label will be supplemented by an additional sticker stating „for use in clinical trial only“ (German Version: „Nur zur klinischen Prüfung bestimmt“).

17.4 Template trial protocol

This trial protocol was written based on a template by the ZKS Leipzig based on the SOPs of the ZKS Leipzig.
The used template version is: Final 9.0 from 20.01.2020

17.5 Supplemental Materials

These documents are relevant to the protocol, but they are not considered part of the protocol. They are stored and modified separately. As such, modifications to these documents do not require protocol amendments.

17.5.1 ECOG PERFORMANCE STATUS SCALE

ECOG Performance Status	
**Grade**	**Description**
0	Fully active, able to carry on all pre-disease performance without restriction
1	Restricted in physically strenuous activity but ambulatory and able to carry out work of a light or sedentary nature, e.g., light house work, office work
2	Ambulatory and capable of all self-care but unable to carry out any work activities. Up and about more than 50% of waking hours
3	Capable of only limited self-care, confined to bed or chair more than 50% of waking hours
4	Completely disabled. Cannot carry on any self-care. Totally confined to bed or chair
5	Dead

Source: Oken_1982

17.5.2 Definition of IPSS-R (International Scoring System-Revised)

Score points							
	**0**	**0.5**	**1**	**1.5**	**2**	**3**	**4**
**Karyotype**[1]	A	-	B	-	C	D	E
**Blasts in bone marrow (%)**	≤ 2	-	>2 < 5	-	5-10	>10	-
**Hb value (g/dl)**	≥ 10	-	8 ≤ 10	< 8	-	-	-
**Platelets (/nl)**	≥ 100	50 < 100	< 50	-	-	-	-
**Neutrophiles (/nl)**	≥ 0.8	< 0.8	-	-	-	-	-

Risk score	Points
**Very low risk**	≤ 1.5
**Low risk**	2-3
**Intermediate risk**	3.5-4.5
**High risk**	5-6
**Very high risk**	>6

[1] A: very good (-Y, del(11q))
B: good (normal, del(5q), del(12p), del(20q), double including del(5q) but not chr7)
C: intermediate (del(7q), +8, +19, i(17q), any other single or double independent clones)
D: poor (-7, inv(3)/t(3q)/del(3q), double including -7/del(7q), complex (3 abnormalities))
E: very poor (complex > 3 abnormalities)

17.5.3 MDS groups according to WHO classification (2016)

Subtype	Dysplastic lines	Cyto-penias	Ring sideroblasts (% of erythroid cells)	Blasts in BM and PB	Karyotype (conventional banding)
**MDS-SLD**	1	1 or 2	<15% / <5 %[2]	BM <5%, PB <1% no Auer rods	All, except del(5q) ± 1 other non-chr 7 abnormality
**MDS-MLD**	2 or 3	1-3	<15% / <5 %[2]	BM <5%, PB <1% no Auer rods	All, except del(5q) ± 1 other non-chr 7 abnormality
**MDS-RS**					
MDS-RS-SLD	1	1 or 2	≥15% / ≥5 %[2]	BM <5%, PB <1% no Auer rods	All, except del(5q) ± 1 other non-chr 7 abnormality
MDS-RS-MLD	2 or 3	1-3	≥15% / ≥5 %[2]	BM <5%, PB <1% no Auer rods	All, except del(5q) ± 1 other non-chr 7 abnormality
**MDS with del(5q)**	1-3	1 or 2	not relevant	BM <5%, PB <1% no Auer rods	Isolated del(5q) or with 1 other non-chr 7 abnormality
**MDS-EB**					
MDS-EB1	0-3	1-3	not relevant	BM 5-9% or PB 2-4% no Auer rods	not relevant
MDS-EB2	0-3	1-3	not relevant	BM 10-19% or PB 5-19% or Auer rods	not relevant
**MDS-U (unclassifiable)**					
with 1% blood blasts	1-3	1-3	not relevant	BM <5%, PB =1% no Auer rods	not relevant
with single lineage dysplasia and pancytopenia	1	3	not relevant	BM <5%, PB <1% no Auer rods	not relevant
based on defining cytogenetic abnormality	0	1-3	<15%[4]	BM <5%, PB <1% no Auer rods	MDS-defining abnormality

1 cytopenias defined as hemoglobin <100 g/L, Platelets <100 x 10^9/L, ANC <1.8 x 10^9/L
2 if SF3B1 has a mutation
3 1% peripheral blasts have to be judged at 2 different times
4 cases with ≥15% of ring sideroblasts have (per definition) significant dyserythropoiesis and are therefore MDS-RS-SLD

17.5.4 CMML-specific prognostic scoring system (CPSS)

Variable	Score		
	**0**	**1**	**2**
WHO Type	CMML1	CMML2	
FAB Type (Leukocyte count)	<13 GPT/L	≥ 13 GPT/L	
Cytogenetics	low	intermediate	high
Transfusion need	no	yes	
**CPSS risk group**			
**Low**	**0**		
**Intermediate 1**	**1**		
**Intermediate 2**	**2-3**		
**High**	**4-5**		

Cytogenetics: low: normal, isolated -Y, intermediate: other abnormalities, high: +8, aberrations of chromosome 7, complex karyotype; Transfusion need defined as ≥ 2 RBC units every 8 weeks for 4 months.

18 REFERENCES

1. Platzbecker U, Fenaux P, Adès L, et al. Proposals for revised IWG 2018 hematological response criteria in patients with MDS included in clinical trials. Blood 2019;133(10):1020–1030.
2. Platzbecker U, Hofbauer LC, Ehninger G, Hölig K. The clinical, quality of life, and economic consequences of chronic anemia and transfusion support in patients with myelodysplastic syndromes. Leuk Res 2012;36(5):525–36.
3. Buscarlet M, Provost S, Zada YF, et al. DNMT3A and TET2 dominate clonal hematopoiesis and demonstrate benign phenotypes and different genetic predispositions. Blood 2017;130(6):753–762.
4. Cook EK, Izukawa T, Young S, et al. Feeding the Fire: The Comorbid and Inflammatory Backdrop of Clonal Hematopoiesis of Indeterminate Potential (CHIP) By Mutation Subtype. Blood 2017;130426.
5. Neves-Costa A, Moita LF. TET1 is a negative transcriptional regulator of IL-1β in the THP-1 cell line. Mol Immunol 2013;54(3–4):264–70.
6. Gañán-Gómez I, Wei Y, Starczynowski DT, et al. Deregulation of innate immune and inflammatory signaling in myelodysplastic syndromes. Leukemia 2015;29(7):1458–69.
7. Arranz L, Arriero M del M, Villatoro A. Interleukin-1β as emerging therapeutic target in hematological malignancies and potentially in their complications. Blood Rev 2017;31(5):306–317.
8. Basiorka AA, McGraw KL, Eksioglu EA, et al. The NLRP3 inflammasome functions as a driver of the myelodysplastic syndrome phenotype. Blood 2016;128(25):2960–2975.
9. Zambetti NA, Ping Z, Chen S, et al. Mesenchymal Inflammation Drives Genotoxic Stress in Hematopoietic Stem Cells and Predicts Disease Evolution in Human Pre-leukemia. Cell Stem Cell 2016;19(5):613–627.
10. Platzbecker U. Treatment of MDS. Blood 2019;1331096–1107.
11. Kordasti SY, Afzali B, Lim Z, et al. IL-17-producing CD4+ T cells, pro-inflammatory cytokines and apoptosis are increased in low risk myelodysplastic syndrome. Br J Haematol 2009;145(1):64–72.
12. Cluzeau T, McGraw KL, Irvine B, et al. Pro-inflammatory proteins S100A9 and tumor necrosis factor-α suppress erythropoietin elaboration in myelodysplastic syndromes. Haematologica 2017;102(12):2015–2020.
13. Chen S, Zambetti NA, Bindels EMJ, et al. Massive parallel RNA sequencing of highly purified mesenchymal elements in low-risk MDS reveals tissue-context-dependent activation of inflammatory programs. Leukemia 2016;30(9):1938–42.
14. Senovilla L, Aranda F, Galluzzi L, Kroemer G. Impact of myeloid cells on the efficacy of anticancer chemotherapy. Curr Opin Immunol 2014;3024–31.
15. De Mooij CEM, Netea MG, Van Der Velden WJFM, Blijlevens NMA. Targeting the interleukin-1 pathway in patients with hematological disorders. Blood 2017;129(24):3155–3164.
16. Howard C, Noe A, Skerjanec A, et al. Safety and tolerability of canakinumab, an IL-1β inhibitor, in type 2 diabetes mellitus patients: A pooled analysis of three randomised double-blind studies. Cardiovasc Diabetol 2014;1394.
17. Greenberg PL, Tuechler H, Schanz J, et al. Revised international prognostic scoring system for myelodysplastic syndromes. Blood 2012;120(12):2454–65.
18. Petti MC, Latagliata R, Breccia M, et al. Spontaneous remission in adult patients with de novo myelodysplastic syndrome: A possible event. Haematologica 2001;861277–1280.

Trial Protocol

A Phase II, Open-Label, Multicenter Study of Orally Administered CA-4948 for the Treatment of Anemia in Patients with Very Low, Low or Intermediate Risk Myelodysplastic Syndromes (MDS)

LUCAS Trial

Coordinating investigator

Prof. Dr. Uwe Platzbecker,

Klinik und Poliklinik für Hämatologie, Zelltherapie und Hämostaseologie

Universität Leipzig - Medizinische Fakultät
Liebigstraße 22, 04103 Leipzig, Germany

Biometry

Dr. Dirk Hasenclever

Institut für Medizinische Informatik, Statistik und Epidemiologie,

Universität Leipzig
Härtelstraße 16-18, 04107 Leipzig, Germany

Sponsor

Universität Leipzig

Ritterstr. 26, 04109 Leipzig, Germany

Date: 31.05.2022

Version: Final 4.0

EudraCT-No.: 2020-003986-20

Table of Contents

		PAGE
**GENERAL INFORMATION**		**5**
Responsible Parties		5
Synopsis		8
Deutsche Synopse		13
**1**	**SCHEDULE OF ASSESSMENTS AND PROCEDURES**	**18**
Flow Chart		24
**2**	**RATIONALE**	**25**
2.1	Medical Background	25
2.2	Rationale	30
2.3	Risk-Benefit Consideration	33
**3**	**OBJECTIVES**	**35**
3.1	Primary Objective	35
3.2	Secondary Objectives	35
**4**	**TRIAL DESIGN AND DESCRIPTION**	**36**
4.1	Trial Design	36
4.2	Requirements at the Trial Sites regarding Personnel and Equipment	36
4.3	Trial Sites and Number of Trial Subjects	37
4.4	Expected Duration of the Trial	37
4.5	Premature Termination of the Trial	37
**5**	**TRIAL SUBJECTS**	**39**
5.1	I/E-criteria for registration for screening investigations	39
5.2	I/E-criteria for definite inclusion	43
5.3	Participation in more than one Clinical Trial	44
5.4	Statement on the Inclusion of Dependent Individuals	44
5.5	Rationale for Gender Distribution	44
**6**	**INVESTIGATIONAL PRODUCT**	**45**
6.1	Trial Drugs	45
6.2	Packaging and Labelling of the Trial Drug	45
6.3	Storage and Handling Requirements	46
6.4	Drug Accountability	46
6.5	Administration of Trial Drug	47
**7**	**INDIVIDUAL TRIAL PROCEDURES**	**53**
7.1	Patient Information and Informed Consent	53
7.2	Enrolment in the Trial	54
7.3	Description of the Treatment Procedures	55
7.4	Description of examinations	58

7.5 Premature Termination of the Therapy or Follow-up 61
7.6 Plan for Further Treatment 62

8 ADVERSE EVENTS (AE/SAE) 63

8.1 Adverse Events (AE) 63
8.2 Safety Analysis 64
8.3 Concomitant Diseases 64
8.4 Serious Adverse Events/Serious Adverse Reactions (SAE/SAR) 65
8.5 Periodic Reports 67
8.6 Suspected Unexpected Serious Adverse Reactions (SUSAR) 67
8.7 Other Safety Relevant Issues 68
8.8 Therapeutic Procedures 68
8.9 Dealing with Pregnancy 69

9 BIOMETRY 70

9.1 Biometrical Aspects of the Trial Design 70
9.2 End Points 70
9.3 Statistical Methods 71
9.4 Statistical Monitoring 71
9.5 Interim Analysis 72
9.6 Final Analysis 72

10 CONCOMITANT SCIENTIFIC PROJECTS 73

11 ETHICAL, LEGAL AND ADMINISTRATIVE ASPECTS 76

11.1 GCP-Statement 76
11.2 Initial Submission 76
11.3 Protocol Amendments 76

12 DOCUMENTATION 78

12.1 General information and Access Rights 78
12.2 Patient File and Source Data 78
12.3 Data Management 79
12.4 Archiving 79

13 REFERENCE EVALUATION 80

14 SUPERVISION OF THE CLINICAL TRIAL 81

14.1 Access to Source Data 81
14.2 Monitoring 81
14.3 Audits 82
14.4 Inspections 82
14.5 Data protection and Confidentiality 82
14.6 Declaration regarding Data Protection 83
14.7 Declaration regarding the Pseudonymized Transfer of Personal Data 83
14.8 Anonymisation of Data after the end of Archiving 83

15 ADMINISTRATIVE AGREEMENTS 83

15.1 Adherence to the Protocol 83

15.2 Funding and Insurance 84
15.3 Notification of Local Authorities 84
15.4 Publication Policy and Registration 84
15.5 Data Sharing Statement 85

16 PROTOCOL SIGNATURES 86

17 PROTOCOL AGREEMENT 87

18 APPENDIX 88

18.1 Modification of the revised IWG 2018 hematological response criteria 88
18.2 Classification of Adverse Events 91
18.3 Acronyms 94
18.4 Labelling 99
18.5 Template trial protocol 99
18.6 Supplemental Materials 99

19 REFERENCES 115

GENERAL INFORMATION[1]

Responsible Parties

Sponsor<br>(according to German Medicinal Products Act)	**Universität Leipzig** (Leipzig University)<br>Ritterstr. 26, 04109 Leipzig, Germany<br><br>Authorized representative of the sponsor:<br>Prof. Dr. Uwe Platzbecker
Coordinating investigator(s)	**Prof. Dr. Uwe Platzbecker**<br>Klinik und Poliklinik für Hämatologie, Zelltherapie und Hämostaseologie<br>Universität Leipzig - Medizinische Fakultät<br>Liebigstraße 22, 04103 Leipzig, Germany<br><br>Phone: +49 341 97 13050<br>Fax: +49 341 97 13059<br>E-Mail: uwe.platzbecker@medizin.uni-leipzig.de
Deputy of Coordinating investigator	**Dr. Anne Sophie Kubasch**<br>Klinik und Poliklinik für Hämatologie, Zelltherapie und Hämostaseologie<br>Universität Leipzig - Medizinische Fakultät<br>Liebigstraße 22, 04103 Leipzig, Germany<br><br>Phone: +49 341 97 20257<br>Fax: +49 341 97 13059<br>E-Mail: annesophie.kubasch@medizin.uni-leipzig.de
Project Management	**Dr. Susanne Melzer**<br>Zentrum für Klinische Studien Leipzig<br>Universität Leipzig - Medizinische Fakultät<br>Härtelstr.16-18, 04107 Leipzig, Germany<br>Phone: +49 341 97 16317<br>Fax: +49 341 97 16259<br>E-Mail: susanne.melzer@zks.uni-leipzig.de
Pharmacovigilance	**Madlen Dörschmann**<br>Zentrum für Klinische Studien Leipzig<br>Universität Leipzig - Medizinische Fakultät<br>Härtelstr.16-18, 04107 Leipzig, Germany<br>Phone: +49 341 97 16129

[1] The contents of this trial protocol refer to women and men equally. However, for reasons of better readability, the male form is chosen for all personal designations. The female form is always taken into account. An exception are those contents solely referring to women.

	Fax: +49 341 97 16278<br>E-Mail: pharmacovigilance@zks.uni-leipzig.de
Data Management (Database)	**Berit Kalina**<br>Zentrum für Klinische Studien Leipzig<br>Universität Leipzig - Medizinische Fakultät<br>Härtelstr.16-18, 04107 Leipzig, Germany<br>Phone: +49 341 97 16275<br>Fax: +49 341 97 16259<br>E-Mail: berit.kalina@zks.uni-leipzig.de
Data Management (Project assistance)	**Anke Stolle**<br>Zentrum für Klinische Studien Leipzig<br>Universität Leipzig - Medizinische Fakultät<br>Härtelstr.16-18, 04107 Leipzig, Germany<br>Phone: +49 341 97 16268<br>Fax: +49 341 97 16259<br>E-Mail: anke.stolle@zks.uni-leipzig.de
Biometry	**Dr. Dirk Hasenclever**<br>Institut für Medizinische Informatik, Statistik und Epidemiologie<br>Universität Leipzig - Medizinische Fakultät<br>Härtelstr.16-18, 04107 Leipzig, Germany<br>Phone: +49 341 97 16121<br>Fax +49 341 97 16109<br>E-Mail: dirk.hasenclever@imise.uni-leipzig.de
Monitoring	**Angelika Siegmund**<br>Zentrum für Klinische Studien Leipzig<br>Universität Leipzig - Medizinische Fakultät<br>Härtelstr.16-18, 04107 Leipzig, Germany<br>Phone +49 341 97 16294<br>Fax: +49 341 97 16259<br>E-Mail: angelika.siegmund@zks.uni-leipzig.de
Reference Boards	**Central Morphology Lab,**<br>**Sabine Haase**<br>Marien Hospital Düsseldorf<br>Klinik für Onkologie und Hämatologie, Palliativmedizin<br>Hämatologisches Speziallabor<br>Rochusstraße 2, 40479 Düsseldorf, Germany<br>Phone: +49 2 11 44 00-68 79<br>Fax: +49 (02 11) 44 00-24 27<br>E-Mail: sabine.haase@vkkd-kliniken.de
Funding	**Curis, Inc.**<br>128 Spring St,<br>Lexington MA, 02421-3112<br>Phone: 617-503-6500

	Fax: 617-503-6501<br>E-Mail: rvonroemeling@curis.com;<br>emartinez@curis.com
Concomitant scientific projects	**Leipzig:**<br>PD Dr. Michael Cross<br>Dr. Marie Schneider<br>José Carreras Forschungslabor<br>Universitätsklinikum Leipzig AöR<br>MK1 – Hämatologie und Zelltherapie<br>Johannisallee 32A, 04103 Leipzig, Germany<br>Phone: +49 341 97 13137<br>Fax: +49 341 97 13089<br>E-Mail: michael.Cross@medizin.uni-leipzig.de<br>E-Mail: marie.schneider@medizin.uni-leipzig.de<br><br>**Dresden:**<br>Prof. Dr. med. Marc Schmitz<br>Institute of Immunology, Faculty of Medicine, Technische Universität Dresden<br>Fetscherstr. 74<br>01307 Dresden<br>Phone: +49 (0) 351 4586501<br>E-Mail: marc.schmitz@tu-dresden.de<br><br>**London:**<br>Dr. Shahram Kordasti<br>King's College London<br>Applied Cancer Immunopathology<br>Guy’s Hospital, St Thomas Street<br>London, SE1 9RT<br>Phone: +44 (0) 20 7848 8028<br>E-Mail: shahram.kordasti@kcl.ac.uk

Synopsis

Title of the trial	A phase II, open-label, multicenter study of orally administered CA-4948 for the treatment of anemia in patients with very low, low or intermediate risk myelodysplastic syndromes (MDS).
Acronym	LUCAS
Indication	Anemia in patients with very low, low or intermediate risk myelodysplastic syndromes (MDS)
Primary objective of the trial/ primary endpoint	**Primary Objective**<br>To evaluate the proportion of patients who have an erythroid response (HI-E) according to IWG 2018 criteria [1] separately for both independent substudies.
Secondary objectives of the trial/secondary endpoints	**Secondary Objective**<br>• HI-E response duration<br>• Time to HI-E<br>• Hemoglobin changes from baseline<br>• Red blood cell transfusion (RBC) independence ≥ 8 and ≥ 12 weeks<br>• Frequency of RBC transfusions in transfusion dependent patients<br>• Neutrophil and platelet (HI-N and HI-P) responses<br>• Disease progression according to IWG 2018 criteria<br>• Safety and toxcicity profile of CA-4948<br>• Quality of life (QoL)<br>• Mutational pattern and burden of selected genes and their influence on response
Trial design	This is a phase II, open-label, multicenter study to evaluate the effects of CA-4948 monotherapy on anemia in patients with very low, low or intermediate risk MDS who are either naïve or refractory to erythropoiesis-stimulating agents (ESA)
Methodology	Patients who meet the study eligibility criteria will be enrolled within 28 days of screening.<br>Patients will receive CA-4948 administered orally twice a day (on days 1-21 of 4 repeated 28-days cycles).<br>**Cohort A** (n= **approximately 42**): ESA exposed (and refractory or intolerant)<br>**Cohort B** (n= **approximately 42**): ESA naive AND serum erythropoietin level >200 U/L<br><br>All patients will be treated with **200 mg CA-4948 (BID)** as starting dose.<br>**Criteria for Evaluation:**<br>***Safety:*** We aim to determine the safety profile of CA-4948. Assessments will include drug exposure; characterization of toxicities; characterization of the AEs including type, incidence, severity (graded by CTCAE v5.0), seriousness, and relationship to treatment; effects on vital signs and laboratory parameters; and

	changes from baseline in ECGs, physical examinations, and ECOG performance status.
Trial population	**Key Inclusion Criteria**<br>• De novo myelodysplastic syndrome (MDS) OR *de novo* myelodysplastic/myeloproliferative neoplasias (MDS/MPN) including MDS/MPN-RS-T, MDS/MPNu, aCML or CMML<br>• IPSS-R up to 3.5: very low/low/intermediate risk disease (MDS); MDS/MPN < 10% bone marrow blasts; CPSS-Score low/intermediate for CMML<br>• Symptomatic anemia (based on complete Hb time course and transfusion history):<br>o NTD (non transfusion dependent): < 3 RBC transfusions and mean hemoglobin level <10 g/dl within the last 16 weeks<br>o LTB (low transfusion burden): 3-7 RBC transfusions within the last 16 weeks in at least two transfusion episodes<br>o HTB (high transfusion burden): ≥ 8 RBC transfusions within the last 16 weeks<br>• Defined transfusion strategy<br>• No available option of an approved MDS therapy and classification of prior erythropoiesis-stimulating agent (ESA) treatment as follows:<br>o Cohort A: ESA exposed (and refractory or intolerant)<br>o Cohort B: ESA naive AND serum erythropoietin level >200 U/L<br><br>**Key Exclusion Criteria**<br>• Inability to swallow and retain oral medications (> 10 pills)<br>• ECOG ≥ 3<br>• Inacceptable organ function (Serum creatinine > 2 × ULN or calculated creatinine clearance < 30 ml/min; AST OR ALT > 2 × ULN; Total bilirubin > 2 × ULN)<br>• Prior treatment with azacitidine or decitabine.<br>• Treatment with ESA, G-CSF, GM-CSF, lenalidomide, luspatercept and/or another investigational drug or device up to 14 days before registration<br>• Treatment with iron chelation therapy 56 days before registration, except for subjects on a stable or decreasing dose for at least eight weeks prior to inclusion and during study treatment<br>• Major surgery within 28 days prior to registration.<br>• Known human immunodeficiency virus infection<br>• Active infectious hepatitis<br>• Presence of an acute or chronic toxicity resulting from prior anti-cancer therapy that has not resolved to Grade ≤ 1 (except anemia and alopecia)<br>• Severe cardiovascular disease<br>• Positive serum pregnancy test in women of childbearing potential

	• Women of childbearing potential and men who partner with a woman of childbearing potential unwilling to use highly effective contraceptive methods for the duration of the study and for 90 days after the last dose of CA-4948<br>• Age under 18 years at registration<br>• Inability to provide written informed consent
Sample size	**Number of Patients:**<br>Approximately 84 patients (42 patients in each of the two cohorts).
Therapy	**Investigational Product, Dose, and Mode of Administration:**<br>CA-4948 will be formulated as 50 mg immediate release film coated tablets.<br>CA-4948 will be given in 28 day cycles with CA-4948 administered orally for BID dosing for 21 consecutive days (d1-d21), followed by 7 days off drug. Patients will take the dose of CA-4948 orally with water (no food two hours prior and one hour after) at approximately the same time each day, approximately 12 hours apart.<br><br>**Duration of Treatment:**<br>Each treatment cycle will be 28 days in length. Patients will be treated for 16 weeks (**4 cycles**) until the primary endpoint (response assessment). Patients with erythroid response (HI-E) after four cycles who tolerate CA-4948 may continue to receive CA-4948 until PD or loss of HI-E response.<br><br>Treatment will be stopped, in case of: PD (determined by the investigator based on the 2018 IWG Revised Response Criteria for MDS), intolerable toxicity, withdrawal from the trial, or study termination.<br><br>**Dose Modifications:**<br>A dose reduction or discontinuation from CA-4948 treatment should be considered in the following situations:<br>• Non-hematologic toxicity* of Grade ≥3 with the following exceptions:<br>▪ Grade ≥3 nausea or vomiting, that resolves to < Grade 2 within three days with appropriate treatment<br>▪ Grade ≥3 fatigue lasting ≤ 7 days<br>▪ Grade ≥3 electrolyte disturbance resolving to ≤ Grade 1 or baseline within three days (supplementation allowed)<br>In case of non-hematological toxicities, hold CA-4948 dosing until recovery to ≤ Grade 2 (exception: rhabdomyolysis, treatment resumed after recovery to grade 1). When resuming treatment, the dose must be reduced by one level.<br><br>*Note: Infections will not be considered a toxicity, given the propensity for these events within the study population, unless their severity or duration is longer than expected. Grade ≥ 3 asymptomatic laboratory abnormalities

	suspected to be treatment-related should be considered to qualify as a toxicity.<br>• Drug-induced liver injury [2]<br>▪ Permanently discontinue CA-4948 dosing<br>• Grade 4 neutropenia or thrombocytopenia in the absence of preexisting neutropenia or thrombocytopenia or disease progression (blast count <5%)**<br>▪ Hold CA-4948 dosing until recovery to ≤ Grade 2. Transfusion is allowed. When resuming treatment, CA-4948 dose may be reduced by one level.<br>**Note: SAEs due to progression (i.e. pancytopenia, anemia, leukopenia/neutropenia, thrombocytopenia, neutropenic fever, neutropenic infections, thrombocytopenic bleedings) are excluded from toxicities.<br>• Any toxicity considered in the Investigator´s decision clinically unacceptable<br>▪ Hold CA-4948 dosing until resolves to ≤ Grade 1 or baseline, or permanently discontinue<br>• In case of AEs, CA-4948 may be held for up to 28 days, after which it will be discontinued if the AE has not resolved. However, if there is evidence of clinical benefit, and the Investigator believes it is in the best interest of the patient to resume CA-4948 after a hold of more than 28 days, this may be allowed after resolution of the AE. If treatment is resumed, dose of CA-4948 should be reduced by one dose level.<br>• Reasons unrelated to toxicity (e.g., intercurrent illness or elective surgery) at the discretion of the Investigator<br>▪ Hold CA-4948 dosing for up to 28 days. If there is evidence of clinical benefit and the Investigator believes it is in the best interest of the patient to resume CA 4948 after a hold of more than 28 days.<br>If a patient experiences other grade 3/4 non-hematologic toxicity considered at least possibly related to CA-4948, the CA-4948 treatment will be interrupted until toxicity resolves to ≤ Grade 1. Missed doses of CA-4948 will not be made up.<br><br>**Study Medication Dose Adjustments**<br><table><tr><th></th><th>Dose of CA-4948 (BID)</th><th>Total Daily Dose</th></tr><tr><td>Starting Dose (mg)</td><td>200</td><td>400</td></tr><tr><td>Dose level -1 (mg)</td><td>150</td><td>300</td></tr><tr><td>Dose level -2 (mg)</td><td>100</td><td>200</td></tr></table>
Biometry	**Sample size**: Each of the two cohort constitutes an independent substudy. For each substudy, we use the optimal Simon 2-stage Phase II design, separately with up to 42 patients in each of the two cohorts.<br>For each of the two cohorts, we plan to enroll patients in two stages, with the option to stop early for futility after the first 21 patients, if less than two responses are documented in the first 21 patients included.

	**Analysis set:** The analysis set consists of all formally included patients that have taken any treatment medication. Patients who withdraw before any drug exposure may be replaced.<br><br>**Response rate:** The response rate in each cohort will be estimated with an exact two-sided 95% confidence interval. The response rate will be described by initial transfusion dependency. Furthermore, the difference of the response rates in the two cohorts will be estimated with a two-sided 95% confidence interval as well as the overall response rate in both cohorts combined.<br><br>**Interim Analysis:** The Simon design implies interim analyses in each cohort after the first 21 patients have their response documented.<br><br>**Final Analysis:** The Simon design implies final analyses in each cohort are performed after all 42 patients have their response documented.
Trial Duration	Recruitment period: 24 months<br>Treatment period per patient: 4 months non responder, responder > 4 months until disease progression or loss of HI-E response.<br>FPI-LPO: approximately 30 months<br>Trial Duration: approximately 42 months

Deutsche Synopse

Title der Studie	Eine offene Phase-II-Studie mit oral verabreichtem CA-4948 zur Behandlung von Anämie bei Patienten mit myelodysplastischen Syndromen (MDS) mit sehr niedrigem, niedrigem oder mittlerem Risiko.
Akronym	LUCAS
Indikation	Anämie bei Patienten mit myelodysplastischen Syndromen (MDS) mit sehr niedrigem, niedrigem oder mittlerem Risiko
Primäres Ziel der Studie/ primärer Endpunkt	**Primärer Endpunkt**<br>Erhebung des Anteils der Patienten, die eine erythroide Reaktion (HI-E) haben, gemäss den Kriterien der IWG 2018 [1], getrennt für beide unabhängigen Kohorten.
Sekundäre Ziele der Studie/ sekundäre Endpunkte	**Sekundäre Endpunkte**<br>• Dauer des HI-E-Ansprechens<br>• Zeit bis HI-E<br>• Unabhängigkeit der Erythrozytentransfusion (RBC) ≥ 8 und ≥ 12 Wochen<br>• Häufigkeit von RBC-Transfusionen bei transfusions-abhängigen Patienten<br>• Neutrophilen- und Thrombozytenanprechen (HI-N und HI-P)<br>• Krankheitsprogression nach den Kriterien der IWG 2018<br>• Sicherheits- und Toxizitätsprofil von CA-4948<br>• Lebensqualität (QoL)<br>• Mutationsmuster und Belastung ausgewählter Gene und deren Einfluss auf das Ansprechen
Studiedesign	Hierbei handelt es sich um eine offene Phase-II-Studie zur Bewertung der Auswirkungen der Monotherapie mit CA-4948 auf die Anämie bei Patienten mit MDS mit sehr niedrigem, niedrigem oder mittlerem Risiko, die bisher nicht oder vorab mit einem Erythropoese-stimulierenden Mittel (ESA) behandelt wurden.
Methodik	Patienten, die die Kriterien für die Studienberechtigung erfüllen, werden innerhalb von 28 Tagen nach dem Screening in die Studie eingeschlossen.<br>Die Patienten erhalten CA-4948 zweimal täglich oral verabreicht (an den Tagen 1-21 in vier 28-Tage-Zyklen).<br>**Die Kohorte A (n= ca. 42)**: ESA-exponiert (und refraktär oder unverträglich)<br>**Kohorte B (n= ca. 42)**: ESA-naiv UND Serum-Erythropoetin-Spiegel >200 U/L<br>Alle Patienten werden mit **200 mg CA-4948 (BID)** als Anfangsdosis behandelt.
	**Kriterien für die Bewertung:**<br>***Sicherheit:*** Unser Ziel ist es, das Sicherheitsprofil von CA-4948 zu bestimmen. Die Bewertungen umfassen die Arzneimittelexposition,

	die Charakterisierung von Toxizitäten, die Charakterisierung der AEs einschließlich Ausprägung, Inzidenz, Schweregrad (abgestuft nach CTCAE v5.0), Schwere und Beziehung zur Behandlung, Auswirkungen auf Vitalparameter und Laborparameter sowie Veränderungen gegenüber dem Baselinewert in EKGs, körperlichen Untersuchungen und dem ECOG-Leistungsstatus.
Studienpopulation	**Haupteinschlusskriterien**<br>• De novo myelodysplastisches Syndrome (MDS) ODER *de novo* myelodysplastische/myeloproliferative Neoplasien (MDS/MPN) einschließlich MDS/MPN-RS-T, MDS/MPNu, aCML oder CMML<br>• IPSS-R bis zu 3,5: sehr niedriges, niedriges oder mittleres Risiko (MDS); MDS/MPN < 10% Knochmarksblasten; CPSS-Score bei CMML: niedrig, intermediär<br>• Symptomatische Anämie (basierend auf vollständigem Hb-Zeitverlauf und Transfusionsanamnese):<br>o NTD (nicht transfusionsabhängig): < 3 RBC-Transfusionen und mittlerer Hämoglobinwert <10 g/dl innerhalb der letzten 16 Wochen<br>o LTB: 3-7 RBC-Transfusionen innerhalb der letzten 16 Wochen in mindestens zwei Transfusions-episoden<br>o HTB: ≥ 8 RBC-Transfusionen innerhalb der letzten 16 Wochen<br>• Definierte Transfusionsstrategie<br>• Keine verfügbare Option einer zugelassenen MDS-Therapie und Einstufung der vorherigen Behandlung mit Erythropoiese-stimulierenden Agenzien (ESA) wie folgt:<br>o Kohorte A: ESA-exponiert (und refraktär oder intolerant)<br>o Kohort B: ESA naïve UND Serum Erythropoietinlevel >200 U/L<br><br>**Hauptausschlusskriterien**<br>• Unfähigkeit orale Medikamente zu schlucken (> 10 Tabletten)<br>• ECOG ≥3<br>• Inadequate Organfunktion (Serumkreatinin > 2 × ULN der berechnete Kreatinin-Clearance < 30 ml/min; AST ODER ALT > 2 × ULN; Gesamt Bilirubin > 2 × ULN)<br>• Vorherige Behandlung mit Azacitidin oder Decitabin<br>• Behandlung mit ESA, G-CSF, GM-CSF, Lenalidomid, Luspatercept und/oder anderen Prüfpräparaten oder -produkten bis zu 14 Tage vor der Registrierung<br>• Behandlung mit Eisenchelationstherapie 56 Tage vor der Registrierung, mit Ausnahme von Patienten, die vor dem Einschluss und während der Studienbehandlung mindestens 8 Wochen lang eine stabile oder abnehmende Dosis erhielten<br>• Größere Operation innerhalb von 28 Tagen vor der Registrierung

	• Bekannte Infektion mit dem Human Immunodeficiency Virus<br>• Aktive infektiöse Hepatitis<br>• Vorliegen einer akuten oder chronischen Toxizität, die aus einer früheren Krebstherapie resultiert und nicht auf Grad ≤1 (mit Ausnahme von Anämie und Alopezie) erholt ist<br>• Schwere Herz-Kreislauf-Erkrankung<br>• Positiver Serum-Schwangerschaftstest bei Frauen im gebärfähigen Alter<br>• Frauen im gebärfähigen Alter und Männer, die mit einer Frau im gebärfähigen Alter zusammenleben, die nicht bereit sind, hochwirksame Verhütungsmethoden zu verwenden, für die Dauer der Studie und für 90 Tage nach der letzten CA-4948-Gabe<br>• Alter unter 18 Jahren bei der Registrierung<br>• Unfähigkeit ein schriftliches Einverständnis zu erteilen
Patientenzahl	**Anzahl der Patienten:**<br>Ca. 84 Patienten (42 Patienten in zwei Kohorten).
Therapie	**Prüfprodukt, Dosis und Art der Verabreichung:**<br>CA-4948 wird als 50 mg Filmtablette mit sofortiger Wirkstofffreisetzung hergestellt.<br>CA-4948 wird in 28-tägigen Zyklen verabreicht, wobei CA-4948 oral als BID-Dosierung für 21 aufeinanderfolgende Tage (d1-d21) verabreicht wird, gefolgt von 7 Tagen ohne Medikament. Die Patienten nehmen die Dosis von CA-4948 oral mit Wasser ein (zwei Stunden vor und eine Stunde nach der Einnahme keine Nahrung), und zwar jeden Tag etwa zur gleichen Zeit, im Abstand von etwa 12 Stunden.<br><br>**Dauer der Behandlung:**<br>Jeder Behandlungszyklus wird 28 Tage lang sein. Die Patienten werden 16 Wochen **(4 Zyklen)** lang behandelt, bis der primäre Endpunkt (Beurteilung des Ansprechens) erreicht ist. Patienten mit einem erythroiden Ansprechen (HI-E) nach vier Zyklen, die CA-4948 vertragen, können CA-4948 bis zum PD oder Verlust des HI-E-Ansprechens weiter erhalten.<br>Die Behandlung wird beendet, im Falle des Fortschreitens der Erkrankung (bestimmt durch den Prüfarzt auf der Grundlage der 2018 IWG Revised Response Criteria for MDS), unverträglicher Toxizität, Rücknahme der Einwilligung oder Studienabbruch.

	**Dosis-Änderungen:**<br>Eine Dosisreduktion oder ein Abbruch der Behandlung mit CA-4948 sollte in den folgenden Situationen in Betracht gezogen werden:<br>• Nicht-hämatologische Toxizität* des Grades ≥3 mit folgenden Ausnahmen:<br>▪ Grad ≥3 Übelkeit oder Erbrechen, das bei entsprechender Behandlung innerhalb von drei Tagen auf < Grad 2 abklingt<br>▪ Grad ≥3 Ermüdung mit einer Dauer von ≤ 7 Tagen<br>▪ Grad ≥3 Elektrolytstörung, die sich innerhalb von drei Tagen auf ≤ Grad 1 oder Baselinewert regeneriert (Supplementation erlaubt)<br>▪ Bei nicht-hämatologischen Toxizitäten ist die CA-4948 Dosis bis zur Erholung auf Grad ≤2 zu pausieren (Ausnahme: Rhabdomyolyse, Fortsetzen der Behandlung nach Erholung auf Grad 1). Bei Wiederaufnahme der Behandlung muss die Dosis um eine Stufe reduziert werden.<br>**Hinweis: Infektionen werden angesichts der Erwartungshaltung dieser Ereignisse innerhalb der Studienpopulation nicht als Toxizität betrachtet, es sei denn, die Schwere oder Dauer ist länger als erwartet. Grad ≥3 asymptomatische Laboranomalien, bei denen der Verdacht besteht, dass sie im Zusammenhang mit der Behandlung stehen, sollten als Toxizität betrachtet werden.*<br>• Arzneimittelbedingte Leberschädigung [2]<br>▪ Dauerhaft CA-4948 Gabe absetzen<br>• Grad 4 Neutropenie oder Thrombozytopenie, bei fehlender vorheriger Neutropenie oder Thrombozytopenie oder Progress (Blastenzahl <5%)**<br>▪ CA-4948 Gabe bis zur Genesung auf Grad ≤ 2 pausieren. Transfusion ist erlaubt. Bei Wiederaufnahme der Behandlung kann die CA-4948 Dosis um eine Stufe reduziert werden.<br>***Hinweis: SAEs aufgrund des Fortschreitens der Leukämie (d.h. Panzytopenie, Anämie, Leukopenie/Neutropenie, Thrombozytopenie, neutropenisches Fieber, neutropenische Infektionen, thrombozytopenische Blutungen) sind von Toxizitäten ausgeschlossen*.<br>• Jede Toxizität, die in der Entscheidung des Prüfarztes als klinisch inakzeptabel betrachtet wird<br>▪ CA-4948 bis zur Erholung auf Grad ≤ 1 oder auf den Baseline-Wert pausieren oder Gabe dauerhaft einstellen<br>• Im Falle unerwünschter Ereignisse (AE) kann die CA-4948 Gabe bis zu 28 Tage lang pausiert werden; falls das AE bis dahin nicht abgeklungen ist, erfolgt ein permanentes Absetzen. Wenn es jedoch Beweise für einen klinischen Nutzen gibt und der Pürfarzt der Meinung ist, dass es im besten Interesse des Patienten ist, CA-4948 nach einer Wartezeit von mehr als 28 Tagen wiederaufzunehmen, kann dies nach dem Abklingen des AE erlaubt werden. Wenn die Behandlung wiederaufgenommen wird, sollte die Dosis von CA-4948 um eine Dosisstufe reduziert werden.<br>• Gründe, die nicht mit der Toxizität zusammenhängen (z.B. hinzukommende Erkrankung oder elektive Operation), liegen im Ermessen des Prüfarztes

<table>
<tr>
<td></td>
<td>
CA-4948 Gabe bis zu 28 Tage lang pausieren. Wenn es Beweise für einen klinischen Nutzen gibt und der Prüfarzt der Meinung ist, dass es im besten Interesse des Patienten ist, die Gabe von CA 4948 nach einer Pause von mehr als 28 Tagen wiederaufzunehmen.
Erleidet ein Patient eine andere nicht-hämatologische Toxizität Grad 3/4, die zumindest möglicherweise mit CA-4948 zusammenhängt, wird die Behandlung mit CA-4948 unterbrochen, bis die Toxizität auf ≤ Grad 1 abgeklungen ist. Verpasste Dosen von CA-4948 werden nicht nachgeholt.
Dosismodifikationen der Studienmedikation
<table>
<tr><th></th><th>CA-4948 Dosis (BID)</th><th>Tägliche Gesamtdosis</th></tr>
<tr><td>Startdosis (mg)</td><td>200</td><td>400</td></tr>
<tr><td>Level -1 Dosis (mg)</td><td>150</td><td>300</td></tr>
<tr><td>Level -2 Dosis (mg)</td><td>100</td><td>200</td></tr>
</table>
</td>
</tr>
<tr>
<td>Biometrie</td>
<td>
Patientenanzahl: Jede der beiden Kohorten stellt eine unabhängige Teilstudie dar. Für jede Teilstudie verwenden wir das optimale 2-stufige Phase-II-Design von Simon, separat mit bis zu 42 Patienten in jeder der beiden Kohorten.
Für jede der beiden Kohorten planen wir die Rekrutierung von Patienten in zwei Phasen, mit der Option, nach den ersten 21 Patienten vorzeitig aus Futility-Gründen aufzuhören, falls weniger als zwei Responses bei den ersten 21 Patienten dokumentiert sind.
Analyse-Set: Der Analysesatz besteht aus allen formell eingeschlossenen Patienten, sobald diese das Prüfprodukt eingenommen haben. Patienten, die kein Prüfprodukt erhalten, können ersetzt werden.
Ansprechrate: Die Ansprechrate in jeder Kohorte wird mit einem genauen zweiseitigen 95%-Konfidenzintervall geschätzt. Die Ansprechrate wird durch die anfängliche Transfusionsabhängigkeit beschrieben. Darüber hinaus wird die Differenz der Ansprechraten in den beiden Kohorten mit einem zweiseitigen 95%-Konfidenzintervall sowie die Gesamtansprechrate in beiden Kohorten zusammen geschätzt.
Zwischenauswertung: Das Simon-Design beinhaltet eine Zwischenauswertung in jeder Kohorte, nachdem das Ansprechen der ersten 21 Patienten dokumentiert wurde.
Endauswertung: Das Simon-Design sieht vor, dass die Endauswertung in jeder Kohorte durchgeführt wird, nachdem das Ansprechen aller 42 Patienten dokumentiert ist.
</td>
</tr>
<tr>
<td>Studiendauer</td>
<td>Rekrutierungsdauer: 24 Monate
Dauer der Behandlung pro Patient: 4 Monate (kein Ansprechen), > 4 Monate bei Ansprechen bis zum Fortschreiten der Erkrankung oder Verlust des HI-E-Ansprechens
FPI-LPO: ca. 30 Monate
Studiendauer: ca. 42 Monate</td>
</tr>
</table>

1 SCHEDULE OF ASSESSMENTS AND PROCEDURES

Trial Procedures	Screen-ing	Treatment[2]										End of Treatment Visit	Safety Follow-up Visit	Survival Follow-up
	(-28 days)	Cycle 1[3]			Cycle 2[3]			Cycles 3-4[3]		Primary endpoint visit[4]	Cycles 5-n (only responders)[3]	(+ 7 days post-last dose)	(28 ± 3 days post-last dose)	every 3 to 6 months up to 2 years post-last dose
		Day 1	Day 8	Day 15	Day 1	Day 8	Day 15	Day 1	Day 15		Day 1			
**Informed consent**	X													
**Inclusion/exclusion criteria**	X													
**Demographics and Medical history**	X													
**Physical Examination[5]**	X	X	X	X	X		X	X			X	X	X	

[2] All assessments will be performed prior to daily treatment with CA-4948, unless otherwise specified in the protocol. Pre- and post-dose assessments are for the morning dose of CA-4948.

[3] Each treatment cycle will be 28 days in length +3 days tolerance possible

[4] After 4-treatment cycles. **Corresponds for responder to cycle 5 day 1 visit and for non-responder to end of treatment-visit.**

[5] Complete physical examination only at screening and safety follow-up visit. Symptom-directed examination is sufficient for all other visits.

Trial Procedures	Screen-ing	Treatment[2]										End of Treatment Visit	Safety Follow-up Visit	Survival Follow-up
	(-28 days)	Cycle 1[3]			Cycle 2[3]			Cycles 3-4[3]		Primary endpoint visit[4]	Cycles 5-n (only responders)[3]	(+ 7 days post-last dose)	(28 ± 3 days post-last dose)	every 3 to 6 months up to 2 years post-last dose
		Day 1	Day 8	Day 15	Day 1	Day 8	Day 15	Day 1	Day 15		Day 1			
Vital signs[6] (including weight and heigt)	X	X	X	X	X		X	X			X	X	X	
ECOG Performance Status	X	X			X			X			X	X	X	
Hematology[7]	X	X [8]	X	X	X	X	X	X	X[9]	X	X	X	X	
Serum chemistry[10]	X	X[8]	X	X	X	X	X	X			X	X	X	
Serology (HBV/HCV testing, only in	X													

[6] During treatment, vital signs (including temperature (oral), heart rate, blood pressure (seated 5 minutes)) will be collected pre-dose at each visit-day and also 15, 30 min, 1, 2, and 4 hours (± 5 minutes) post-dose on Cycle 1 Day 1; and 1 hour (± 5 minutes) post-dose on Cycle 2 Day 1. Height needs only to be collected at screening visit, weight on screening visit, first day of each cycle, end of treatment visit and safety follow up.

[7] Complete blood count with differential, platelet count, fibrinogen. **Hb measurement needs to be performed for all cycles on day 15 additionally.**

[8] If Screening assessment was performed within 3 days prior to Cycle 1 Day 1, it does not need to be repeated prior to initiation of dosing.

[9] Only Hb measurement

[10] Sodium, potassium, chloride, calcium, inorganic phosphate, magnesium, blood urea nitrogen, creatinine, glucose, bilirubin (direct and total), AST, ALT, ALP, albumin, globulin, total protein, triglycerides, cholesterol, lipase, amylase, troponin, aldolase (only if CK is not measured), lactate dehydrogenase and creatine kinase (CK), uric acid. Isoenzymes CK-MB and CK-MM will be required if total CK is elevated and/or when clinically indicated.

Trial Procedures	Screen-ing	Treatment[2]										End of Treatment Visit	Safety Follow-up Visit	Survival Follow-up
	(-28 days)	Cycle 1[3]			Cycle 2[3]			Cycles 3-4[3]		Primary endpoint visit[4]	Cycles 5-n (only responders)[3]	(+ 7 days post-last dose)	(28 ± 3 days post-last dose)	every 3 to 6 months up to 2 years post-last dose
		Day 1	Day 8	Day 15	Day 1	Day 8	Day 15	Day 1	Day 15		Day 1			
patients with history)[11]														
Thyroid function tests[12]	X				X			X			X		X	
Pregnancy test[13]	X	X[8]			X			X			X	X		
Coagulation parameters[14]	X	X[8]	X	X	X		X	X			X	X	X	
Urinalysis[15]	X	X[8]	X	X	X			X			X	X	X	

[11] In patients with a history of HBV, hepatitis B core antibody (HBcAb) testing is required and if positive, then HB DNA testing will be performed. In patients with a history of HCV <6 months prior to start of CA-4948, viral load testing is required. HIV test needs to be performed as part of screening assessments for all patients.

[12] Thyroid function tests – TSH and FT4, will be monitored at Screening, on Day 1 of every other cycle starting at Cycle 2 and at the Safety Follow-Up Visit.

[13] In women of childbearing potential, serum β-HCG at Screening; either serum β-HCG or urine pregnancy test at subsequent visits.

[14] Partial thromboplastin time (PTT), and prothrombin time (PT) or international normalized ratio (INR).

[15] Protein, glucose, ketones, urobilinogen, occult blood, and, when indicated by dipstick abnormality, microscopic sediment evaluation.

Trial Procedures	Screen-ing	Treatment[2]										End of Treatment Visit	Safety Follow-up Visit	Survival Follow-up
	(-28 days)	Cycle 1[3]			Cycle 2[3]			Cycles 3-4[3]		Primary endpoint visit[4]	Cycles 5-n (only responders)[3]	(+ 7 days post-last dose)	(28 ± 3 days post-last dose)	every 3 to 6 months up to 2 years post-last dose
		Day 1	Day 8	Day 15	Day 1	Day 8	Day 15	Day 1	Day 15		Day 1			
**Electrocardiogram[16]**	X	X			X									
**Bone marrow assessment**														
Cytogenetics, **local**	X									X		X[17]		
Cytomorphology, **central** (Bone marrow smear)	X									X		X[17]		

[16] Triplicate ECG (after supine for 5 minutes) will be recorded at Screening, at pre-dose and 3 hours (± 10 minutes) post-dose on Cycle 1 Day 1 and Cycle 2 Day 1.
[17] Bone marrow assessment (aspiration) should be performed at EoT only for Responders. For Non-responders the bone marrow assessment is performed at primary endpoint visit that coincides with the EoT.

Trial Procedures	Screen-ing	Treatment[2]										End of Treatment Visit	Safety Follow-up Visit	Survival Follow-up
	(-28 days)	Cycle 1[3]			Cycle 2[3]			Cycles 3-4[3]		Primary endpoint visit[4]	Cycles 5-n (only responders)[3]	(+ 7 days post-last dose)	(28 ± 3 days post-last dose)	every 3 to 6 months up to 2 years post-last dose
		Day 1	Day 8	Day 15	Day 1	Day 8	Day 15	Day 1	Day 15		Day 1			
**Evaluate extent of disease/response to treatment[18]**	X									X				
**AEs assessment**		X	X	X	X	X	X	X		X	X	X	X[19]	X[19]
**Concomitant medications**	X	X	X	X	X	X	X	X			X	X		
**Dispense CA-4948**		X			X			X			X			
**Treatment compliance (patient diary)**					X			X			X	X		
**Quality of life (EORTC QLQ-C30 and EORTC QLQ-FA12)**	X	X			X			X			X	X	X	

[18] complete blood count with differential and bone marrow aspirate is required at Screening and will be repeated to confirm a CR per investigator's discretion and based on 2018 IWG criteria for MDS.
[19] All AEs will be reported following the first dose of CA-4948 through the Safety Follow-up visit. Only treatment-related AEs/SAEs will be captured from Safety Follow-up visit until the End of study Visit.

Trial Procedures	Screen-ing	Treatment[2]										End of Treatment Visit	Safety Follow-up Visit	Survival Follow-up
	(-28 days)	Cycle 1[3]			Cycle 2[3]			Cycles 3-4[3]		Primary endpoint visit[4]	Cycles 5-n (only responders)[3]	(+ 7 days post-last dose)	(28 ± 3 days post-last dose)	every 3 to 6 months up to 2 years post-last dose
		Day 1	Day 8	Day 15	Day 1	Day 8	Day 15	Day 1	Day 15		Day 1			
**Activity tests**	X	X			X			X			X	X	X	
**Survival status[20]**														X
**Concomitant research**														
**Whole blood samples (incl. plasma)**	X	X		X	X			X		X		X[21]		
**Bone marrow aspirat**	X									X		X[21]		
**Bone marrow punch**	X									X		X[21]		
**Saliva sample**	X													

[20] Survival status confirmed every 3-6 months for up to two years or until death, whichever occurs first, from the start of CA-4948 treatment. Telephone survey acceptable.
[21] Samples are taken at EoT only for Responders. For Non-responders the samples are taken at primary endpoint visit that coincides with the EoT.

Flow Chart

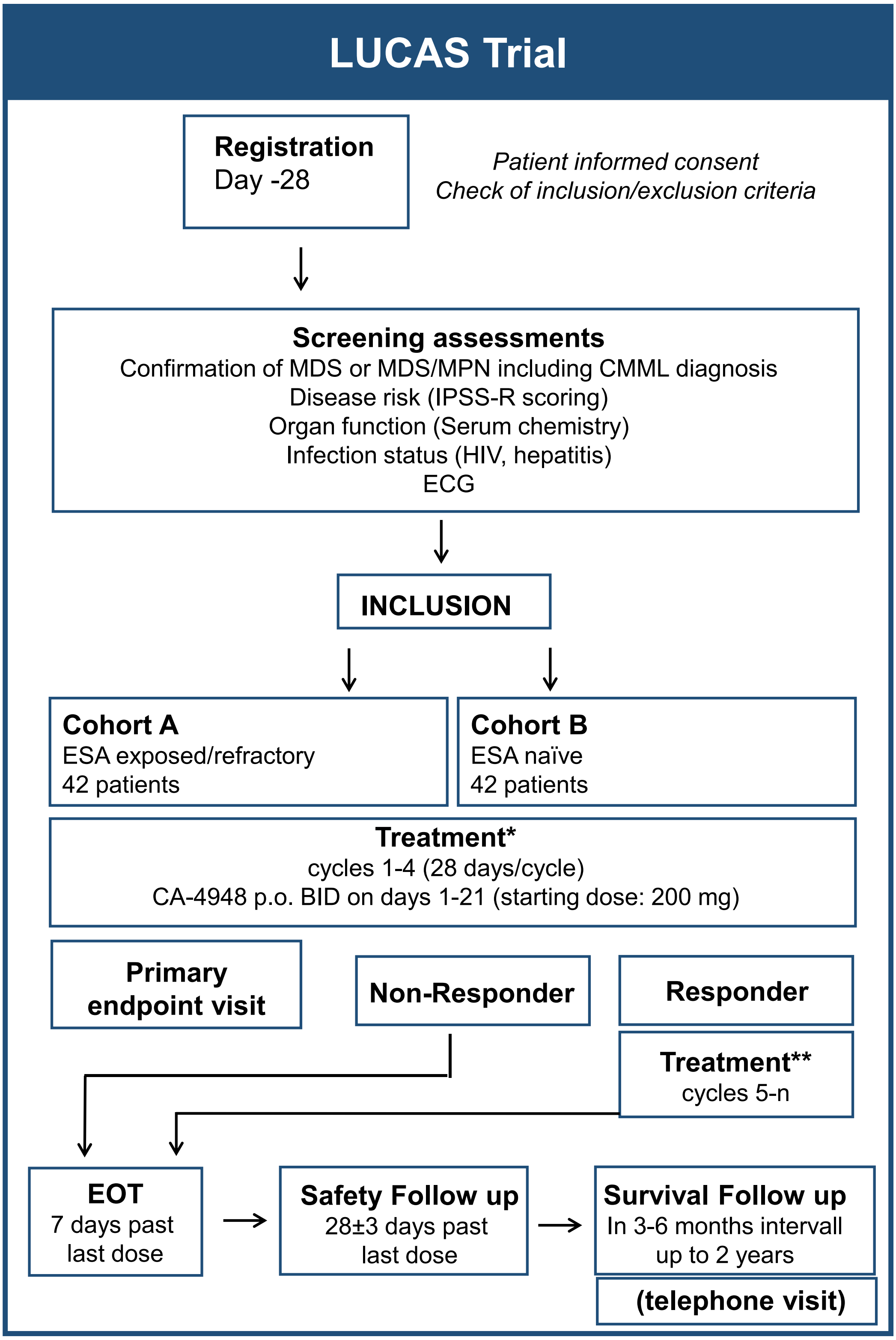

Figure 1: In the LUCAS trial two cohorts will be treated with CA-4948. Treatment over 4 cycles (in total: 16 weeks)*, based on investigators decision the treatment can be continued until disease progression**.

2 RATIONALE

2.1 Medical Background

2.1.1 Introduction

CA-4948 is a novel small molecule inhibitor of interleukin-1 receptor-associated kinase 4 (IRAK4), with a half maximal inhibitory concentration (IC50) value of 57 nanomolar (nM) against IRAK4, and with >350-fold higher binding affinity for IRAK4 compared with IRAKs 1, 2 and 3. IRAK4 kinase plays an essential role in toll-like receptor (TLR) and interleukin-1 receptor (IL-1R) signaling pathways and these pathways are frequently dysregulated in non-Hodgkin's lymphoma (NHL) and AML/MDS malignancies [3–6]. CA 4948 also has inhibitory activity against FLT3 and FLT3 mutants based on preclinical data.

A Phase 1, dose escalation and expansion study (CA-4948-101) assessing the safety, pharmacokinetics (PK), pharmacodynamics, and clinical activity of CA-4948 in patients with relapsed or refractory hematologic malignancies is currently ongoing. The CA-4948-102 trial will assess the safety, PK, pharmacodynamics, and clinical activity of CA-4948 in patients with lower-risk myelodysplastic syndrome (MDS).

2.1.2 Hematologic Malignancies

2.1.2.1 Myelodysplastic Syndrome

Myelodysplastic syndromes are conditions that can occur when blood-forming cells in the bone marrow become abnormal. The major clinical problems in these disorders are morbidities caused by cytopenias and the potential for MDS to evolve into AML. In general population, the incidence rate of MDS is approximately 4.9 per 100,000 people per year [7,8]. Myelodysplastic syndromes are rare among the children/adolescent and young adults, with an incidence rate of 0.1 per 100,000 people per year in those younger than 40 years of age. However, among the individuals between the ages of 70 and 79 years, the incidence rate increases to 30.2 per 100,000 people, and further to 59.8 per 100,000 people among those 80 years of age or older [7,8].

According to the Revised International Prognostic Scoring System (IPSS-R, see further information in 18.6.5), untreated MDS are classified into five risk groups: very low, low, intermediate, high, and very high with median survival time (in the absence of therapy) of 8.8, 5.3, 3, 1.6, and 0.8 years, respectively. The classification is based on the risk scores for bone marrow blast percentage, cytogenetic subgroup, and number of cytopenias; but, this may also include age, performance status, transfusion needs, and other clinical (and increasingly molecular) factors.

The management of MDS is complicated by the generally advanced age of the patients (median age at diagnosis, 70 to 75 years) [9], the attendant non-hematological comorbidities, and the relative inability of older patients to tolerate certain intensive forms of therapy. In addition, when the illness progresses into AML, these patients experience lower response rates to standard therapy than patients with de-novo AML [10].

An intensified surveillance strategy for asymptomatic lower risk MDS (LR-MDS) with a higher genetic risk prognosis, worsening cytopenia and an increasing number of circulating or bone-

marrow blasts, and signs of cytogenetic evolution increases the need for safe treatments to modify the natural history of early MDS. Targeting anemia in genetically defined del(5q) MDS with ESA is effective, but transient. For patients with increasing transfusion requirements, lenalidomide is effective, but progression will eventually occur. Patients with a TP53 mutation are particularly at risk. In addition, the presence of mutated TET2 and RUNX1 also carries a higher risk of progression. Complications include transfusion-related iron overload, immune dysregulation, and development of thrombocytopenia [1]. The oral compound CA-4948, which targets IRAK4, may possess disease-modifying potential in LR-MDS.

Higher risk MDS patients have IPSS score of ≥2.5 and immature blast cells may make up more than 5% of the cells in the marrow. The low blood counts can lead to anemia, neutropenia, or thrombocytopenia. Higher risk MDS carry a greater risk of progression to AML and short survival. At present, allogeneic hematopoietic stem cell transplantation (allo-HSCT) is considered the only curative therapy for the patients with higher risk MDS in fit eligible patients. It has been speculated that epigenetic deregulation plays an important role in the pathogenesis of MDS and standard therapies include the hypomethylating agents azacitidine and decitabine, with hematopoietic stem cell transplantation being the only potentially curative strategy.

2.1.2.2 Role for IRAK4 in MDS

Innate immune signaling plays an important role in MDS and AML [3,11]. Deletion of miR-146a located on chromosome 5q33.3 occurs in 80% of del(5q) MDS and AML [11,12]. TRAF6 and IRAK1 are two targets of miR-146a [13] consistent with increased TRAF6 and IRAK1 protein levels observed in miR-146a knock-out mice or miR-146a decoy inhibition in human cells [14,15]. IRAK1 is overexpressed and hyperactivated in MDS and treatment of MDS/AML cell lines and patient cells with a dual IRAK1/4 inhibitor blocked NF-κB activation and cell proliferation *in-vitro* and delayed MDS-like disease in mouse engraftment models [16]. Other mechanisms of upregulated TLR/IL-1R signaling have been reported for MDS/AML, including overexpression of MYD88, overexpression of TLR1/2/6, and identification of MDS patients harboring mutations within TLR2 that exhibit increased pIRAK1 levels [17,18]. Furthermore, levels of IL-1β and IL-1 receptors are increased in AML patients, and silencing of the IL-1 receptor led to significant suppression of clonogenicity and *in-vivo* disease progression [19]. Additionally, genetic knockdown of IRAK1 or treatment with an IRAK1 inhibitor in primary AML samples and xenograft model showed a significant reduction in leukemia burden [20].

A significantly higher IRAK4 expression in high risk MDS and AML with complex cytogenetics stem and progenitor samples have also been shown [5,21]. Differential expression of alternative IRAK4 mRNA splice variants in MDS and AML patient samples that were associated with U2AF1 mutations have been described [22]. In particular, MDS/AML cases harboring U2AF1 mutations had substantially higher levels of the IRAK4 "long-form" transcript which produces full-length active IRAK4 protein containing the MYD88 interacting domain. In contrast, U2AF1-wt cells expressed predominately an IRAK4 "short-form" transcript variant lacking exon 4, MYD88 interacting coding sequence, thus producing an inactive IRAK4 protein. Importantly, expression of the long IRAK4 mRNA variant is associated with poor AML patient outcome. U2AF1 mutations are present in ~11% of MDS and 6% of AML patient samples [23].

2.1.2.3 Targeting IRAK4

The IRAK family consists of four members: IRAK1, IRAK2, IRAK3/M and IRAK4. IRAK4 is a key downstream mediator of TLR and IL-1R pathways [3,24,25]. TLRs and IL-1R are essential

for the innate immune response [26]. Furthermore, TLR/IL-1R-mediated signaling plays a role in the adaptive immune response and controls cellular processes, such as inflammation, apoptosis and cellular differentiation. Therefore, dysregulation of TLR/IL-1R-mediated signaling is recognized as an important factor in both cancer initiation and progression [3].

MYD88 is an adaptor protein essential for innate immune signaling. Under normal conditions, MYD88 forms a multi-unit complex with IRAK4 upon activation of TLRs or IL-1R, resulting in activation of IRAK4, followed by phosphorylation and activation of IRAK1 by IRAK4. Activated IRAK1 and IRAK4 proteins bind to TNF [21] receptor associated factor 6 (TRAF6), mediating nuclear factor kappa light chain enhancer of activated B-cells (NF-κB signaling; [16,24]). Mutations in MYD88 can lead to aberrant signaling, contributing to cancer cell survival and proliferation [21]. Although development of inhibitors of MYD88 and other adaptor proteins has been challenging, IRAK4 may be a more viable and promising target for inhibition since its function is necessary for signaling through MYD88 [4,15]. The central role of TLR/IL-1R signaling in inflammatory, autoimmune and oncology disorders, further solidifies IRAK4 as an attractive therapeutic target [6,21].

In summary, IRAK4 kinase plays an essential role in TLR and IL-1R signaling pathways and these pathways are frequently dysregulated in non-Hodgkin's lymphoma and AML/MDS malignancies.

2.1.2.4 Mechanism of Action of CA-4948

CA-4948 Inhibits Activities of the Toll-Like Receptor and IL-1 Receptor Pathway

CA-4948's ability to inhibit the TLR signaling pathway was evaluated in a monocytic cell line. Inhibition of the TLR pathway in the presence of CA-4948 was demonstrated via reduced phosphorylation of the downstream proteins I-KappaB kinase (IKKB), NF-κB p65 and extracellular-signal regulated kinase (ERK). In separate experiments using the same cell line, CA-4948 reduced levels of an NF-κB reporter gene, whose expression was driven by TLR-agonist stimulation of NF-κB family members p65 and c-Rel. Furthermore, CA-4948 blocked IL-1R stimulated cytokine production in these cells. Thus, CA-4948 acts via inhibition of IRAK4, resulting in blockade of the MYD88 signaling pathway. By inhibiting this pathway, proinflammatory and other growth-related pathways are repressed.

2.1.2.5 Nonclinical Safety

Preclinical CA-4948 studies conducted to date include neurobehavioral safety evaluation in mice, cardiovascular evaluation in rats and dogs, 28-day repeat-dose toxicity studies in mice and dogs with toxicokinetics, genotoxicity assessment in bacteria and mice, as well as phototoxicity assessment *in-vitro*.

Neurobehavioral Evaluation

Safety pharmacology studies in mice revealed no neurobehavioral effects at doses up to 100 mg/kg/day. Please refer to the CA-4948 Investigator's Brochure (IB), current version is part of the investigator site file, for further information on observed changes at a dose of 300 mg/kg/day.

Cardiac Evaluation

Cardiac safety studies did not indicate a prominent electrophysiological risk either *in-vitro* or *in-vivo*. *In-vitro*, CA-4948 did not produce any significant inhibition of human ether-a-go-go related gene (hERG) potassium current up to the highest feasible concentration of 30 µM. *In-vivo*, a cardiovascular radiotelemetry assessment was conducted in dogs after single oral doses of 5, 15 and 30 mg/kg CA-4948 or vehicle. A transient increase in heart rate was observed for the first 4 hours post CA-4948, but this was not thought to be biologically significant. There were no effects on hemodynamic or electrocardiogram (ECG) parameters or body temperature, compared with control.

Mice 28-day Toxicology Study

In the Good Laboratory Practices (GLP) study in CD-1 mice, repeated oral daily doses of CA-4948 for 28 consecutive days was well tolerated up to 125 mg/kg/day, with no mortalities. At this dose, reddish pink urine, mild hypoactivity and piloerection were observed transiently in a few mice. In mice treated at higher doses (≥ 200 mg/kg/day), mortalities were observed. The majority of mice found dead or sacrificed in moribund conditions exhibited clinical signs prior to death including reddish pink urine, mild to severe hypoactivity, piloerection, body cold to touch, reduced skin turgor, partial ptosis and/or immobility, along with pathological changes in the bone marrow, lymphoid tissues, pancreas and skeletal and cardiac muscle. At the highest dose evaluated in mice (300 mg/kg/day), mild-to-marked mineralization of the base of the aorta, atrium, and ventricle were also noted.

Other findings at doses ≥ 200 mg/kg/day included reduced levels of red and white blood cell counts, lymphocytes, neutrophils and hemoglobin, and increases in reticulocyte and platelet counts. These findings were associated with mild to moderate reductions in bone marrow cellularity. Increased levels of aspartate aminotransferase (AST), creatine kinase (CK)-MB and aldolase were also reported and correlated with macroscopic and microscopic changes observed in heart and skeletal muscle. Increases in alkaline phosphatase (ALP) at ≥ 125 mg/kg/day and alanine aminotransferase (ALT) as well as total and indirect bilirubin at doses > 200 mg/kg/day were observed but not associated with any microscopic evidence of liver injury.

Based upon the results of this study, the severely toxic dose is considered to be 200 mg/kg/day and the no observed adverse effect level (NOAEL) is considered to be 125 mg/kg/day.

Of note, in the 14-day toxicology study in dogs (see IB for additional details) where an increase in CK levels was also noted in the high dose (50 mg/kg/day) group, no microscopic correlate (i.e., changes in skeletal or myocardial muscle) was noted for these changes.

Dog 28-day Toxicology Study

In dogs, adverse clinical and post-dose observations were generally limited to the 50 mg/kg/day group, while the 5 and 15 mg/kg/day doses were generally well tolerated. Changes at the 50 mg/kg/day dose did not include any clear effects on hematology parameters, but did include red urine, loose/liquid and sometimes discolored/red feces, vomit, salivation, subdued/hunched behavior, reduced appetite and consumption of food, loss of body weight, increases in AST/ alanine aminotransferase (ALT), CK, triglycerides, creatinine, and

urea levels, reductions in tri-iodothyronine and thyroxine levels, body tremors, cold to touch/warm to touch, and prolonged QT interval, corrected (QTc) interval.

Males administered 50 mg/kg/day for eight days showed apparently prolonged group mean QT and QTc(V) intervals on Day 14, despite having been off dose for the prior six days. These parameters were 28 or 20 msec longer, respectively, than in concurrent controls, and were increased from baseline by 34 or 22 msec, respectively. No other correlating findings were noted, and no microscopic changes occurred in the heart. A relationship between prolongation of the QTc interval with CA-4948 dosed at 50 mg/kg/day could not be excluded, though no QT/QTc interval effects were noted in the cardiovascular safety pharmacology study in telemetered dogs that received up to 30 mg/kg/day (see above). Additional findings included thymic atrophy and decreased cellularity in the lymphoid system (lymph nodes, gut-associated lymphoid tissue, Peyer's patch and thymus).

Cardiac troponin I (cTnI) levels were increased at 4 to 6 hours after the first dose of 15 or 50 mg/kg/day but generally not thereafter. In 15 mg/kg/day animals, increased cTnI levels were only notable in 2 animals (2.4 or 8 fold increase over baseline). Similar to the high-dose group, thymic atrophy was seen. Male sexual organs and associated tissues were found to be targets of toxicity in dogs at 15 or 50 mg/kg/day, and included changes in germ cell depletion, tubule degeneration, luminal cellular debris in the epididymis, and reduced luminal sperm.

Dosing at the 50 mg/kg/day dose level in dogs continued only up until day eight due to early death in one animal, and the premature sacrifice of three additional animals two to five days after their last dose. After a washout period of 12 to 15 days, a reduced high dose of 30 mg/kg/day was administered to surviving animals for up to 12 days. This was tolerated but with increasing evidence of clinical observations, declining food consumption and minor weight losses for some individuals. Fewer signs were observed for those administered 30 mg/kg/day, although subdued/lethargic behavior was noted for most of these animals within the first week after the restart of dosing.

The cause of death in the animal at 50 mg/kg/day was attributed to gastrointestinal tract lesions; specifically, acute inflammation in the ileum and/or cecum, and was considered due to CA-4948. Based on this, the prolongation of QTc interval and increasing evidence of clinical observations when the surviving animals were restarted at 30 mg/kg/day, dose levels >30 mg/kg/day were considered to have exceeded tolerable levels and 15 mg/kg/day is the highest non-severely toxic dose (HNSTD) in dogs.

<u>Genotoxic Studies</u>

There is no genotoxic potential of CA-4948 based on studies in bacteria and mice. Although marginal increases in the frequency of micronucleated polychromatic erythrocytes at ≥ 150 mg/kg/day were reported in the *in-vivo* bone marrow micronucleus test in CD-1 mice, these increases could be due to secondary effects of hypothermia induced by the test item in CD-1 mice.

<u>Phototoxicity Study</u>

CA-4948 was found to be negative for causing phototoxicity.

Table 1: Results from *in-vivo* Toxicology Studies (for further details, see IB version 2.0 16 Oct 2018)

	NOAEL (mg/kg)	HNSTD (mg/kg)	STD (mg/kg)
**CD1 Mouse Studies**			
28-day GLP Study	125	125	200
14-day non-GLP Study	150	150	450/300*
**Beagle Dog Studies**			
28-day GLP Study	15	15	50/30**
14-day non-GLP Study	20	20	40/75***

*Dose reduced on Day 4

**Dose level lowered due to early mortality of an animal and removal of several animals from study due to toxicity

***Dose increased on Day 11

2.2 Rationale

2.2.1 Hypothesis and Experimental Aspects of the Clinical Trial

CA-4948 is an inhibitor of IRAK4, a key molecule involved in the function of signaling pathways that controls inflammation, apoptosis, cellular differentiation, and other important cellular processes in certain hematological malignancies. CA-4948's IRAK4 inhibitory activity is unique and may contribute additive therapeutic benefit by targeting key inflammatory pathways in MDS.

Increasing clinical and biological data indicate that innate immune signaling is an important determinant of the pathophysiology of myelodysplastic syndromes (MDS) [18,27–29] MDS represent a group of acquired clonal disorders of hematopoietic stem and progenitor cells (HSPCs), characterized by ineffective hematopoiesis, peripheral cytopenias, genetic instability, and an increased risk of progression to acute myeloid leukemia (AML) [30]. Recently, cell-extrinsic factors promoting inflammation in the bone marrow have been proposed as important mechanisms that contribute to selective competitive advantage of mutant cells to expand and survive. Thus, a pro-inflammatory bone marrow microenvironment and aberrant innate immune and cytokine signaling might be pivotal in the pathogenesis of hematological malignancies like MDS [28]. The presence of abnormal levels of cytokines, chemokines and growth factors in the peripheral blood and bone marrow of MDS patients has been extensively documented. In general, levels of TNF-α, IFN-γ, TGF-β, IL-6, the myeloid growth factors granulocyte colony-stimulating factor (G-CSF) and macrophage colony-stimulating factor (M-CSF) are increased in MDS patients compared to age-matched controls, reflecting a profound dysregulation of both inflammatory signaling and myeloid differentiation. Interestingly, the secretion of TNF-α and other related pro-inflammatory cytokines are elevated in lower-risk MDS, whereas immunosuppressive cytokines like IL-10 and suppressive regulatore T-cells are more intensely secreted in higher-risk MDS patients, leading to the survival of the malignant clone and progression of disease because of active suppression of a protective immune response [18].

One important hallmark of MDS is the activation of the NLRP3 inflammasome (*Figure 2*). Through TLR4 signaling and formation of reactive oxygen species (ROS) in the bone marrow, damage-associated molecular patterns (DAMPs) like S100A8 and S100A9 increase NLRP3 expression in MDS hematopoietic stem cellsHSC. The resulting activation of IRAK1 and IRAK4 as well as caspase-1 followed by generation of IL-1β and pyroptotic cell death seems to contribute to the MDS phenotype. Inhibition of IRAK4 kinase activity could therefore alter the key inflammatory pathways in MDS (IL-1β and TLR-driven signaling) with the aim to improve anemia and prevent disease progression.

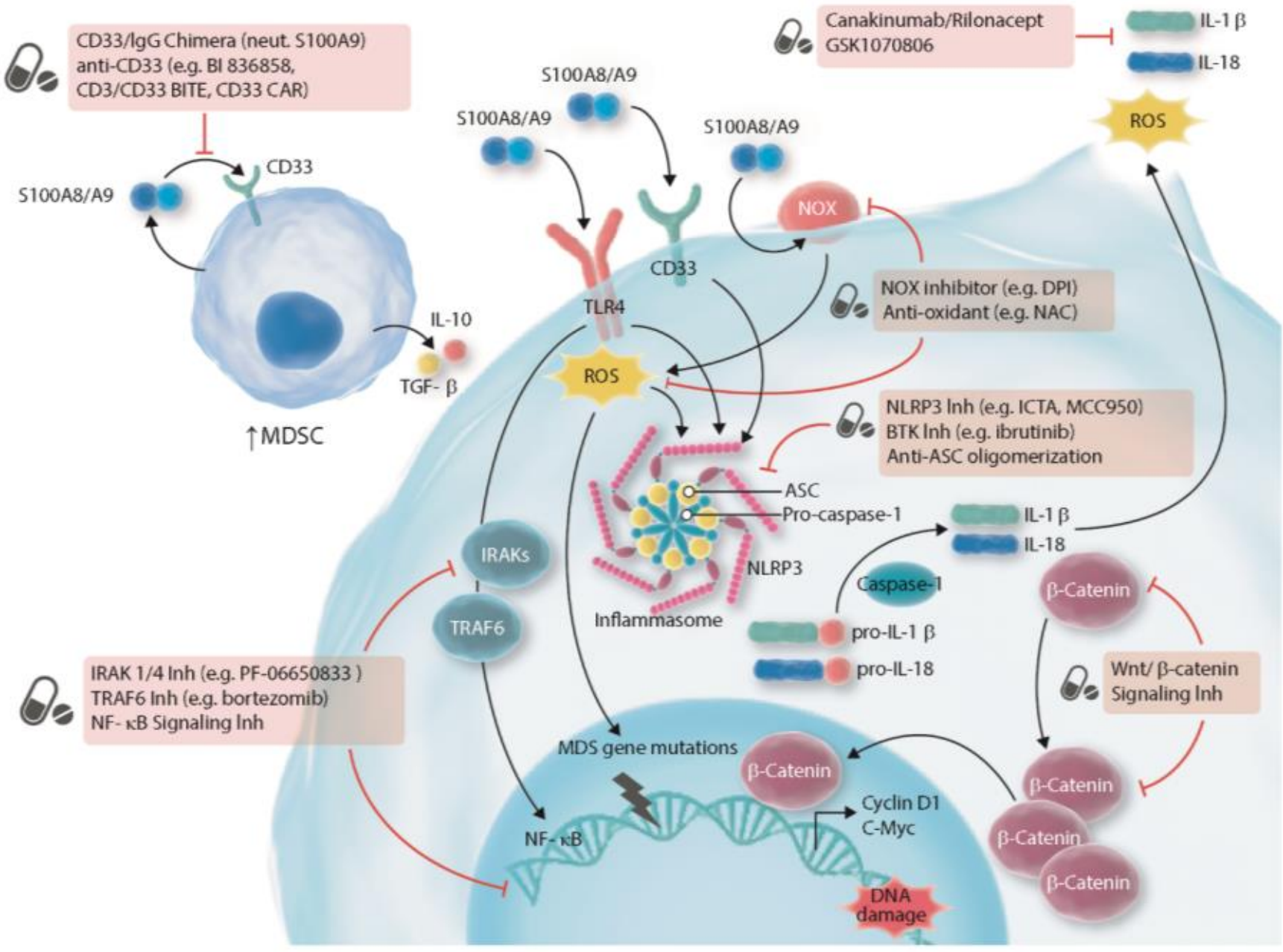

Figure 2. *The central role of inflammatory signaling in the pathogenesis of myelodysplastic syndromes [31].*

Moreover, MDS specimens displayed marked upregulation of inflammasome transcripts, including mRNAs encoding IL-1β and IL-18 (increased 3.7-fold and 29.6-fold) in lower-risk MDS compared to normal donors, whereas expression was increased to a lesser extent (1.1-fold and 9.2-fold) in higher-risk MDS specimens. Interestingly, inhibition of NLRP3 or neutralization of DAMPs restores effective hematopoiesis, underscoring the importance of this pathway as a valuable therapeutic target in MDS [16,32].

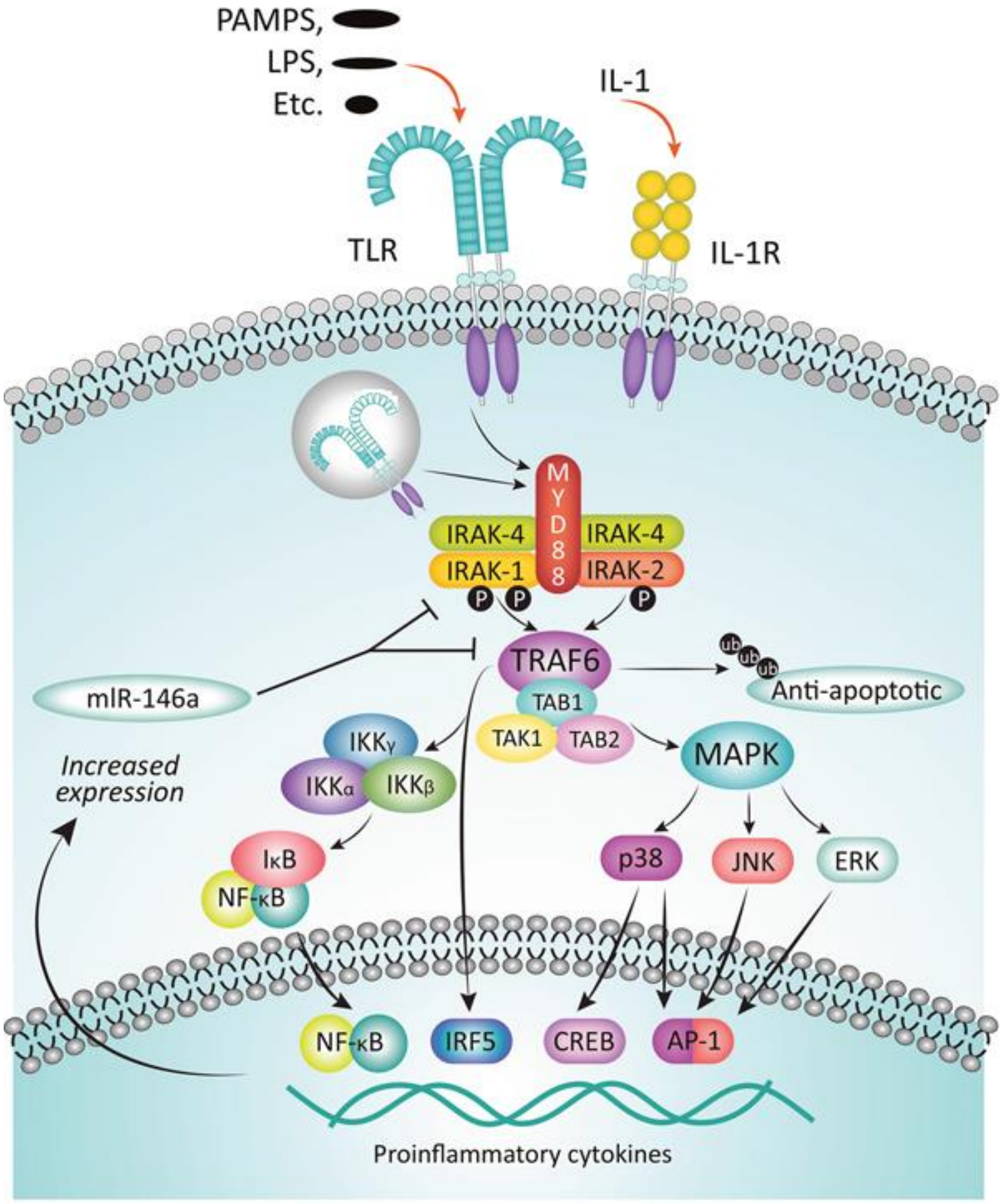

Figure 3. *Function of IRAKs in the myddosome complex [33].*

Thus, this multicenter, open-label trial of orally administered CA-4948 monotherapy in adult patients with lower-risk MDS aims to gather more safety data and obtain an early signal of efficacy. CA-4948 will be administered BID on days 1-21 of repeated 28-day cycles. Safety parameters for frequent evaluation (see the Schedule of Events) were selected based on the results of the toxicology and safety pharmacology studies. During the first two cycles, monitoring will be frequent to ensure safety. Based on the preclinical studies, potential hematologic toxicities may include neutropenia, thrombocytopenia, lymphopenia and anemia. Other potential toxicities include elevations in transaminases and bilirubin, as well as changes in other serum chemistries. Specifically, elevations in markers of muscle damage or inflammation (i.e., CK, the isoenzymes CK-MB and CK-MM and aldolase), elevations in amylase and lipase, and a reduction in thyroid function tests have been seen. In addition, cardiac adverse events (AEs) are possible given prolongation of QTc interval and elevations in cTnI.

The clinical activity of CA-4948 will be assessed according to the 2018 IWG Revised Response Criteria for MDS [1]. For all patients, exploratory biomarkers will be assessed in blood and/or tumor samples.

2.3 Risk-Benefit Consideration

First results for in-human studies were recently gained in the CA-4948-101 trial. Nearly 30 resistant/refractory NHL patients were exposed to up to 400 mg CA-4948 (twice daily) of continuous oral dosing. In general CA-4948 had shown an acceptable safety profile in this trial (CA-4948-101). Three patients were dosed for more than 6-12 months and were able to dose escalate to 300 mg BID with no dose limiting toxicity (DLT). Nevertheless, reversible rhabdomyolysis was found at 400 mg BID in 2 patients:

Case 1: The patient experienced signs and symptoms at the end of the first week, including muscle soreness and urine discoloration. High elevation of creatine phosphokinase levels. Co-medication included rosuvastatin, known to induce rhabdomyolysis. Study treatment was interrupted. Rosuvastatin treatment was discontinued upon investigators decision. The symptoms subsided without complications (no acute renal failure), and creatine phosphokinase levels normalized. CA-4948 was re-introduced at a lower dose of 200 mg BID after less than two weeks of interruption. The event of Grade 3 rhabdomyolysis did not re-occur, and the reduced CA-4948 dose was well tolerated. The causality with study drug was suspected, a definitive relationship to CA-4948 was not been established due to the statin co-medication.

Case 2: Grade 3 rhabdomyolysis with similar signs and symptoms as above occurred in a second patient receiving a continuous daily dose of 400 mg BID over seven days. This patient had vigorous physical exercises as risk factors for rhabdomyolysis. Treatment was interrupted for two weeks, and the patient recovered without complications. Study drug was re-introduced at a dose level of 300 mg BID, which was subsequently well tolerated.

Based on these results the 400 mg BID dose level was considered above the Maximum Tolerated Dose (MTD) for the CA-4948-101 trial. As a consequence starting and ongoing dose was reduced for all patients to 300 mg BID. According to the different biology, patients with NHL or MDS are different patient populations, which might show different tolerability for CA-4948. For patients with high risk MDS treatment at higher CA-4948 doses might be beneficial, if tolerated.

For patients with lower-risk MDS, who have a low risk of progression to AML or death, the main treatment goals are to improve cytopenias (most commonly anemia, which can cause fatigue) and health-related quality of life (HRQoL). For patients with higher-risk MDS, treatments aim to modify the disease course, reduce the risk of progression to AML, and extend survival. Treatment options for patients with lower-risk MDS include erythropoiesis-stimulating agents (ESAs) (e.g. epoetin alfa), biologic response modifiers (e.g. lenalidomide), erythroid maturation agents (luspatercept) and investigational drugs. Nevertheless, luspatercept and lenalidomide is only approved for a small sub cohort of patients with lower-risk MDS. Thus, after ESA failure, the majority of MDS patients have no other available treatment options and the inclusion in a clinical trial is the only chance.

Based on the preclinical studies, potential hematologic toxicities of CA-4948 may include neutropenia, thrombocytopenia, lymphopenia and anemia. Other potential toxicities include elevations in transaminases, bilirubin, alkaline phosphatase, blood urea nitrogen, triglycerides, sodium, creatinine, markers of muscle damage or inflammation, amylase, and lipase, a reduction in thyroid function, and prolongation of QTc interval and elevations in cTnI.

In clinical studies the most commonly reported TEAEs include decreased neutrophil count, increased creatinine and alanine aminotransferase (ALT). Preliminary data exist using CA-4948 in lymphoma patients, that a small sub cohort of patients developed transient anemia (CTCAE grad 1 and 2). Therefore, we will closely monitor the hemoglobin level of patients, especially at the beginning of therapy, similar to other already known drugs used in MDS (e.g. lenalidomide).

Patients presenting with an impaired liver or kidney function or a severe cardiovascular disease will not be included into the LUCAS trial and there will be a close observation of the patients during their trial participation.

To ensure patient safety, close monitoring is carried out during the first two treatment cycles and includes the measurements/analysis of: transaminases, bilirubin, amylase, lipase, troponin, lactate dehydrogenase, creatine kinase (isoenzymes CK-MB and CK-MM if clinically indicated), uric acid, TSH, FT4, urine analysis, Furthermore, patients stay in the clinic for observation on the day of treatment start (day 1, cycle 1) and will be monitored closely for vital signs, temperature, heart rate, blood pressure and by ECG during their stay.

If CA-4948 showed the potential to improve non-transfusion-dependent and transfusion-depend anemia and by this to keep the patients off RBC transfusions on a longer term, this would represent a great advantage in terms of safety, quality of life and convenience for these patients. It also would significantly reduce the burden of transfusion management in the MDS centers and prevent long-term damage, such as iron overload and its consequences, resulting from RBC transfusions.

To minimize the risk of already reported rhabdomyloysis during CA-4948 therapy, patients are treated with an initial dose of 200 mg BID in the LUCAS trial. Although other CA-4948 studies use 300 mg BID as the initial dose, the investigators consider that 200 mg BID is sufficient for patients with anemia and LR-MDS.

The CA-4948-101 trial enrolls patients with high-risk MDS or AML. The patients have already received several prior therapies, which have not been successful. In this patient population, a significantly increased IRAK4 expression was observed and therefore constitutes an interesting therapeutic target. For acute treatment, "high doses" of IRAK4 inhibitors are suitable. Within this trial we enroll de novo low risk (LR)-MDS patients. This patient population does not have a comparable therapeutic history and, due to the lower severity of the disease.

Both trials follow different therapeutic approaches: "high-dose" CA4948 therapy in combination with hypomethylating agents in the CA-4948-101 trial and "low-dose" CA-4948 monotherapy for Hb stabilization and improvement of quality of life in the LUCAS trial.

With the minimal dose reduction from 300mg to 200mg BID in this lower-risk MDS setting, we aim to show fewer side effects (including hematological toxicities and possible rhabdomylosis), leading to fewer cases of dose reduction or treatment stops and thus enables patients to receive continuous treatment at a stable dose (21 days treatment, 7 days therapy pause).

Given the above, we consider the risk-benefit ratio of this trial as favorable.

3 OBJECTIVES

3.1 Primary Objective

To evaluate the proportion of patients who have an erythroid response (HI-E) according to the modified IWG 2018 criteria [1] separately for both independent substudies.

3.2 Secondary Objectives

- To evaluate HI-E response duration
- To evaluate time to HI-E
- To evaluate hemoglobin changes from baseline
- To evaluate red blood cell transfusion (RBC) independence ≥ 8 and ≥ 12 weeks
- To evaluate frequency of RBC transfusions in transfusion dependent patients
- To evaluate neutrophil and platelet (HI-N and HI-P) responses
- To evaluate disease progression according to IWG 2018 criteria
- To evaluate safety and tolerability of CA-4948
- Quality of life (QoL)
- Mutational pattern and burden of selected genes and their influence on response

Concomitant scientific projects are described in chapter 10.

4 TRIAL DESIGN AND DESCRIPTION

4.1 Trial Design

This is a phase 2, open-label, multicenter study, to evaluate the effects of CA-4948 monotherapy on anemia in patients with very low, low or intermediate risk MDS who are not currently receiving treatment with an erythropoiesis-stimulating agent (ESA). Within this study-population we study two cohorts independently: Cohort A: ESA exposed (and refractory or intolerant) and Cohort B: ESA naive AND serum erythropoietin level >200 U/L. This trial will be conducted to characterize the safety, tolerability and efficacy of CA-4948.

4.2 Requirements at the Trial Sites regarding Personnel and Equipment

Qualification of investigator/deputy and medical staff in the trial team

This chapter defines general requirements for trial staff in the LUCAS trial as required by GCP. Trial specific and/or national requirements regarding the qualification of the trial sites and trial staff are specified in the qualification documents used for application to the institutional review board and/or responsible authorities in more detail.

Coordinating Investigator/Principal Investigator

The **coordinating investigator in multicentre trials** and his/her deputy are licenced to practice medicine, are medical specialists and have at least two years of working experience in the study specific indication (MDS/MPN/CMML patients). They have theoretical and practical experience in conducting clinical trials.

Principle Investigator/ Investigator

Principal Investigator and Investigators are licenced to practice medicine, are medical specialists[22]) and have at least two years of work experience in study specific indication (MDS/MPN/CMML patients*)*.

The Investigator is responsible for selecting and assembling the trial team members (especially the medical staff) according to the requirements of this trial protocol and the specifications in the qualifications documents the sponsor will provide to each trial site. Furthermore, the investigator is responsible for training and supervision of the trial team and providing all necessary information during the course of the trial. This has to be documented accordingly.

4.2.1 Essential technical equipment at the trial sites and involvement of other facilities in the trial

Trial site should fulfill at least the following technical requirements:

- Personal computer with internet access

[22] The necessary completion of the medical specialist training as a criterion for the qualification of the investigator must be determined trial specificly after consultation with the sponsor and defined in the qualification documents.

- Storage space for trial medication, trial specific documents (investigator site file)
- In case of use of electronic patient files, only validated systems, with change tracking and access only to trial patients by third parties or assisted control,

For the concomitant research programm trial sites should be equiped at least with refrigerator and centrifuge.

4.3 Trial Sites and Number of Trial Subjects

It is expected that approximately 84 patients will be included (each 42 patients per cohort), paragraph on sample size discussion in chapter 9.1.

4.4 Expected Duration of the Trial

Recruitment will start with the inclusion of the first patient at the first trial site and stop after the planned recruitment period (24 months) and/or number of 84 included patients is reached.

Start of the study is defined as date of the first written informed consent. End of study correlates to last patient last visit (LPLV).

The study design provides for two separate substudies in cohorts A and B. Sample sizes for each substudy will be reached at different time points. Thus, stop of accrual and time of final analysis will differ by cohort.

4.5 Premature Termination of the Trial

Premature termination of the trial for a single patient is described in chapter 7.5.

4.5.1 Termination of the Trial at a Single Site

The trial can be aborted at a single site if:

- the protocol is not adhered to,
- the quality of data is deficient,
- there is inadequate recruitment.

The coordinating investigator decides whether or not to exclude the site, together with the sponsor and biometrician if appropriate.

Investigators and sites no longer participating in the trial must inform the coordinating investigator immediately and should provide justification for the decision. Further treatment of patients still involved in the trial has to be arranged together with the coordinating investigator.

CAUTION: Trial site may be temporarily placed on hold or closed by the sponsor if no or insufficiently qualified personnel (investigator/deputy) is available.

4.5.2 Termination of the whole Trial or of individual Arms of the Trial

The trial can be terminated prematurely by the sponsor´s representative in the event of

- serious adverse events/unacceptable toxicity

- changes in the risk-benefit considerations, e.g. as a result of unexpected adverse events
- new insights from other trials
- an insufficient recruitment rate.

The final decision regarding the premature termination of the trial will be made by the coordinating investigator/the sponsor or the authorised representative.

Since the trial is subject to German Medicinal Products Act (Arzneimittelgesetz – AMG), the approval can be rescinded or the trial can be terminated by the responsible federal authority (competent authority - CA) or the responsible ethics committee.

5 TRIAL SUBJECTS

Patients will be included in the trial in a two-step procedure:

1. Registration for the study with informed consent, preliminary check of inclusion/exclusion criteria, and initiation of study-specific screening investigations (see 5.1). Patients should only be registered if valid baseline data are expected to be available at the end of the baseline evaluation period.

2. Formal inclusion with a final check of inclusion and exclusion criteria (see 5.2) after completion of all study-specific screening investigations at the end of the baseline evaluation period and shortly before start of treatment.

The **baseline evaluation period** overlaps with the screening period and consists of the **last 16 complete weeks** (MO to SO, 112 days) before the day of formal inclusion.

Further details on enrolment process can be found in chapter 7.2.

5.1 I/E-criteria for registration for screening investigations

5.1.1 Inclusion Criteria

Target Population

1. **Diagnosis of**
   a. de novo **myelodysplastic syndrome (MDS)** OR
   b. de novo **myelodysplastic/myeloproliferative neoplasias (MDS/MPN)** including MDS/MPN-RS-T, MDS/MPNu, aCML, or CMML according to WHO criteria

   NOTE: Diagnosis will be confirmed during screening assessment
2. **Very low, low or intermediate risk disease MDS with up to 3.5 points** according to the revised International Prognostic Scoring System (IPSS-R) classification (to be confirmed during screening assessment). For MDS/MPN < 10% bone marrow blasts at screening. For CMML low or intermediate risk according to CMML-Specific Prognostic Scoring System (CPSS Score).
3. **Symptomatic anemia:** Symptomatic anemia (all NTD, LTB, or HTB) has to be documented in the 16 weeks baseline period ending on the day of inclusion.

   Patients should be registered only if it is expected at time of registration that
   a. **a valid and complete Hb and transfusion history** will be available at inclusion AND
   b. the **Hb Mean** over the baseline period will be **less than 10 g/dL** OR **three or more RBC-transfusions** will have been given during the baseline period documenting transfusion dependence.
4. **Defined transfusion strategy**: A transfusion trigger threshold is documented which characterizes the transfusion strategy - ideally for the whole baseline period, but at least for the time from registration to the end of the study.
5. No available option of an approved MDS therapy (according to decision of the treating physician) and classification of prior **erythropoiesis-stimulating agent (ESA) treatment** as follows:

a. *Cohort A*: **ESA exposed (and refractory or intolerant)**
b. *Cohort B:* **ESA naïve** AND serum erythropoietin level **>200 U/L**

5.1.2 Exclusion Criteria

Compliance with major study procedures

1. Inability to swallow and retain oral medications (> 10 pills)[23]
2. Patient does not accept bone marrow sampling during screening and after the treatment.
3. Patient does not accept up to weekly peripheral blood sampling during screening and treatment.

Safety

4. Eastern Cooperative Oncology Group (ECOG) Performance Status of ≥3 at registration
5. Inacceptable organ function at registration (serum chemistry is part of screening assessments):
    a. Serum creatinine >2 × ULN (Kidney function) or calculated creatinine clearance < 30 ml/min (NOTE: creatinine clearance should be calculated per institutional standard. GFR can also be used)" (both measurements required)
    b. Aspartate aminotransferase (AST) > 2 × ULN OR alanine aminotransferase (ALT) > 2 × ULN (Liver function) (both measurements required)
    c. Total bilirubin >2 × ULN
        i. exception >3 × ULN in patients with documented Gilbert's syndrome

Interfering treatments

6. Prior treatment with azacitidine (injectable or oral) or decitabine.
7. The patient medically requires treatment with the following drugs that are forbidden during the trial or was exposed to one of these 14 days before registration:
    a) Erythropoiesis stimulating agent (ESA) or luspatercept,
    b) Granulocyte colony-stimulating factor (G-CSF) and granulocyte-macrophage colony stimulating factor (GM-CSF)
    c) Lenalidomide
    d) Another investigational drug or device, or approved therapy for investigational use
8. Iron chelation therapy

    NOTE: if therapy was initiated 56 days prior to registration patient can be included. Recently initiated iron chelation [< 56 days prior to registration] might influence

[23] During the informed consent process, the investigator must actively ask the patient: "Do you have problems swallowing tablets?"

interpretation of hematological response after start of trial medication

9. Major surgery within 28 days prior to registration.

Concomitant diseases

10. Known human immunodeficiency virus (HIV) infection (HIV test needs to be performed as part of screening assessments)
11. Active infectious hepatitis (Type B (HBV) or Type C (HCV) need to be measured for screening assessments)
12. Hepatitis virus detectable within 6 months before registration in patients with a history of hepatitis
13. History of other invasive malignancy, unless definitively treated with curative intent, provided it is deemed to be at low risk for recurrence by the treating physician

    NOTE: examples of such conditions include but are not limited to non-melanoma skin cancer, carcinoma in situ [including superficial bladder cancer and cervical intraepithelial neoplasia] and organ-confined prostate cancer

14. Presence of an acute or chronic toxicity resulting from prior anti-cancer therapy that has not resolved to Grade ≤1 (except anemia and alopecia), as determined by NCI CTCAE v 5.0 at registration.
15. Known allergy or hypersensitivity to any component of the formulation of CA-4948[24] used in this study
16. Severe cardiovascular disease (as assessed by the investigator)

    NOTE: examples of such conditions include but are not limited to

    - myocardial infarction within 6 months registration,
    - unstable angina within 6 months registration,
    - New York Heart Association (NYHA) Class III or greater congestive heart failure,
    - serious arrhythmias uncontrolled on treatment,
    - clinically significant pericardial disease,
    - Known QTc (according to Friderica, [34]) abnormality > 450 msec on electrocardiogram (ECG) (will be re-measured during screening assessments)

Formal requirements

17. Positive serum pregnancy test in women of childbearing potential
18. Women of childbearing potential and men who partner with a woman of childbearing potential unwilling to use highly effective contraceptive methods for the duration of the

[24] The 50 mg CA-4948 tablet is formulated to contain 50 mg CA-4948 drug substance, 190.0 mg lactose monohydrate, 208.2 mg microcrystalline cellulose, 20.8 mg croscarmellose sodium, 20.8 mg hypromellose, 5.0 mg sodium lauryl sulphate, and 5.2 mg magnesium stearate. The ingredients are wet-granulated, dried and pressed into 500 mg core tablets. The resulting core tablets are then film-coated with Opadry AMB II. The total weight of the coated tablet is 520 mg. The drug product is packaged with silica gel desiccant in canister in HDPE bottle with an induction sealed foil seal and screw cap.

study and for 90 days after the last dose of CA-4948

19. Age under 18 years at registration
20. Inability to provide written informed consent (Subject without legal capacity who is unable to understand the nature, scope, significance and consequences of this clinical trial)
21. Simultaneous participation in another interventional clinical trial or participation in any clinical trial involving administration of an investigational medicinal product within 28 days prior registration

5.2 I/E-criteria for definite inclusion

5.2.1 Inclusion Criteria to be checked after screening assessments

Target population

1. **Diagnosis confirmed by central morphology review**
    a. MDS or
    b. MDS/MPN including MDS/MPN-RS-T, MDS/MPNu aCML, or CMML
2. **Revised IPSS (IPSS-R) risk classification (for MDS only) confirmed by central morphology review**
    a. Very low risk or
    b. Low risk or
    c. Intermediate risk disease (up to 3.5 points)
    d. For MDS/MPN <10% marrow blast
    e. For CMML low or intermediate risk according to CPSS Score.
3. **Symptomatic Anaemia confirmed by formal algorithm** (based on complete Hb time course and transfusion history for the baseline period documented in the study database)**:**
    c. **NTD (non transfusion dependent):** < 3 RBC transfusions and mean hemoglobin[25] level <10 g/dl within the last 16 weeks
    d. **LTB (low transfusion burden):** 3-7 RBC transfusions within the last 16 weeks in at least two transfusion episodes
    e. **HTB (high transfusion burden):** ≥ 8 RBC transfusions within the last 16 weeks
4. ESA treatment during registration and inclusion
    a. *Cohort A*: **no ESA exposure** in the past 28 days.
    b. *Cohort B*: **ESA naive** AND serum erythropoietin level **>200 U/L**

[25] Hb measurements ideally available every two weeks. At least five valid (> 7 days after RBC event) Hb measurements that span at least 100 days within the baseline evaluation period.

5.2.2 Exclusion Criteria

Safety

5. ECOG ≥3
6. Inacceptable organ function (see definitions in 5.1.2)

   NOTE: Local lab needs to be repeated if period of registration and inclusion is > 7 days

Interfering treatments (between registration and inclusion)

7. Treatment with azacitidine (injectable or oral) or decitabine
8. Treatment with G-CSF, GM-CSF, Luspatercept, Lenalidomide, Iron chelating agents, another investigational drug or device
9. Major surgery

Concomitant diseases (between registration and inclusion)

10. Severe CVD
11. HIV infection, active hepatitis infection, detectable hepatitis virus
12. Presence of an acute or chronic toxicity from prior anti-cancer therapy with Grade >1

Formal requirements

13. Positive pregnancy test in women of childbearing potential

    NOTE: Either a serum or a urine pregnancy test needs to be repeated, if period of registration and inclusion is > 7 days

5.3 Participation in more than one Clinical Trial

During the verification of the inclusion and exclusion criteria the investigator/his deputy or authorised medical staff of the trial team checks if the patient is currently participating in any other interventional clinical trial(s). Should this be the case, the patient will not be included.

Moreover, by signing the informed consent form, the patient confirms that he is not participating in any other interventional clinical trial simultaneously.

5.4 Statement on the Inclusion of Dependent Individuals

During the screening procedure, all patients will be interviewed concerning any potential relationship to the investigator/ his deputy or to medical staff of the trial team or the coordinating investigator or the sponsor.

In case of a potential benefit of the trial for the patient he should not be excluded from the trial, if any dependency exist, the patient cannot be included by the person he is dependent on.

5.5 Rationale for Gender Distribution

All patients with very low, low or intermediate risk MDS that fulfil the inclusion and exclusion criteria will be informed about the clinical trial and asked to participate.

6 INVESTIGATIONAL PRODUCT

Curis Inc. will supply CA-4948 at no charge to study patients participating in this clinical trial.

6.1 Trial Drugs

Generic Name:	CA-4948
Potential Marketing Authorization Holder:	Curis Inc., 128 Spring St | Building C, Suite 500 Lexington, MA 02421
Manufacturer:	Almac Clinical Services Limited, Finnabair Industrial Estate, Dundalk, Co. Louth, A91P9KD, Ireland<br>And<br>Almac Clinical Services Limited,<br>9 Charlestown Road, Seagoe Industrial Estate<br>Craigavon, BT63 5PW, United Kingdom
Drug allocation:	Provided by sponsor
Batch number:	Will be provided later
Formulation used:	CA-4948 is formulated as 50 mg immediate release film coated tablets. The 50 mg tablets are yellow, round biconvex and plain on both sides.
Packaging:	25 tablets per bottle
Storage conditions:	Store at ambient temperature (15-25 °C)
Stability:	Two years
Preventive measures/Incompatibility:	Store at proper storage conditions

6.2 Packaging and Labelling of the Trial Drug

The investigational product will be manufactured, packed and labelled by Almac (two manufacturing sites involved as mentioned above) according to the standards of Good Manufacturing Practice (GMP).

The trial drug will be labelled as required by the ICH-GCP Guideline E6 and the German Medicinal Products Act (AMG § 10). The labelling will be in the official language(s) of the countries in which the trial takes place.

The master label for the investigational medicinal product (IMP) is filed in attachement 18.4.

The trial preparations will not be shipped before all requirements for the start of the clinical trial have been fulfilled according to the internal processes. The trial medication will be shipped

by Curis, Inc. to the sponsor (executed by Almac). A detailed documentation of every shipment, number of tablets and packages, which will be used in this clinical trial will be made and kept on file.

6.3 Storage and Handling Requirements

The investigator shall take responsibility for and shall take all steps to maintain appropriate records and ensure appropriate supply, storage, handling, distribution and usage of investigational product in accordance with the protocol and any applicable laws and regulations.

Clinical supplies must be stored in a secure, limited-access location under the storage conditions specified above.

Receipt and dispensing of trial medication must be recorded by an authorised person at the trial site.

Clinical supplies may not be used for any purpose other than that stated in the protocol.

The study drug should be stored at controlled room temperature (20-25°C or 68-77°F; excursions are permitted between 15° and 30°C [59° and 86°F]) in a tightly closed container, in a secure area, with access limited to the investigator and authorized site staff.

Only patients enrolled in the trial may receive the investigational product. The investigational materials are to be handed out only by authorized trial personell. For self-administration, patients may be provided with a sufficient number of tablets at the first day of each cycle.

6.4 Drug Accountability

The trial drug (CA-4948) will be shipped by the sponsor (executed by the pharmacy of the University Hospital Leipzig) directly to the trial site. The trial drug may only be used within the framework of this clinical trial and in accordance with this trial protocol.

Each trial site will receive enough trial medication to treat the first two patients up to primary endpoint visit at the beginning of the trial. Depending on the number of patients being included in the trial, each trial site will receive additional trial product after a written request to the ZKS Leipzig, which will inform the pharmacy of the University Hospital Leipzig on the need of medication at the site. All trial drugs must be kept in a locked area with access restricted to designated trial personnel.

Expired bottles will be replaced.

The trial team will confirm receipt of the trial drug in writing and will keep a record of the trial drug dispensed to and returned from each patient.

Receipt, distribution, return and disposal of the trial drug must be properly documented on the forms provided by the sponsor, giving the following information: trial protocol number, sender, receiver, date, mode of transport, quantity, batch number and expiration date.

The sponsor or its designee will monitor the drug accountability records at regular points during the trial and will perform a drug reconciliation at the end of trial.

Upon completion or termination of the trial, all unused and/or partially used investigational product will be destroyed at the site per institutional policy provided that procedures for proper disposal have been established according to applicable federal, state, local and institutional guidelines. The principal investigator is responsible to arrange for disposal and to keep appropriate records of disposal.

6.5 Administration of Trial Drug

6.5.1 Procedures

CA-4948 will be given in 28 day cycles with CA-4948 administered orally for BID dosing for 21 consecutive days (d1-d21), followed by 7 days off drug. Four cycles are scheduled before primary response assessment.

All doses of CA-4948 (in clinic and self-administered) will be administered with water, at approximately the same times each day, approximately 12 hours apart, if possible. No food or meals should be consumed 2 hours prior to and 1 hour after CA 4948 dosing. The morning dose of CA-4948 will be administered in clinic on the first day of each cycle.

For self-administration, patients will be provided with and trained to complete a patient diary, which must be returned to the clinic for review at the next cycle. Missed or vomited doses should not be made up and must be noted on the patient diary.

Patients may continue to receive CA-4948 cycles until progressive disease (PD) has been documented or other discontinuation criteria have been met (Section 6.5.1.26.5.1.3).

6.5.1.1 Planned Duration of Treatment

Each treatment cycle will be 28 days in length. Patients will be treated for 16 weeks until the primary endpoint assessment.

Treatment will be stopped, in case of: PD (determined by the investigator based on the 2018 IWG Revised Response Criteria for MDS [1]), intolerable toxicity, withdrawal from the trial, or study termination.

Patients with erythroid response (HI-E) after four cycles who tolerate CA-4948 may continue to receive CA-4948 until PD or loss of HI-E response.

6.5.1.2 Starting dose and dose modifications (dose reductions and holds)

The starting dose will be 200 mg of CA-4948 twice daily.

Table 2: Study Medication Dose Adjustments

	**Dose of CA-4948 (BID)**	**Total Daily Dose**
Starting Dose (mg)	200	400
Dose level -1 (mg)	150	300
Dose level -2 (mg)	100	200

A dose **reduction or discontinuation** from CA-4948 treatment should be considered in the following situations:

- Non-hematologic toxicity of Grade ≥3
    - Hold CA-4948 dosing until recovery to ≤ Grade 2. When resuming treatment, the dose must be reduced by one level.
      Exceptions for this rule:
        - Grade ≥3 nausea or vomiting, that resolves to ≤ Grade 1 within 3 days with appropriate treatment
        - Grade ≥ 3 fatigue lasting ≤ 7 days
        - Grade ≥ 3 electrolyte disturbance resolving to ≤ Grade 1 or baseline within three days (supplementation allowed)
        - Rhabdomyolysis, see 6.5.3
- Drug-induced liver injury [35]
    - Permanently discontinue CA-4948 dosing
- Grade 4 neutropenia or thrombocytopenia in the absence of a preexisting condition or disease progression (blast count <5%)[26]
    - Hold CA-4948 dosing until recovery to ≤ Grade 2. Transfusion is allowed. When resuming treatment, CA-4948 dose may be reduced by one level according to the discretion of the PI.
- Any toxicity considered in the Investigator´s decision clinically unacceptable
    - Hold CA-4948 dosing until resolves to ≤ Grade 1 or baseline, or permanently discontinue
- In case of AEs, CA-4948 may be held for up to 28 days, after which it will be discontinued if the AE has not resolved. However, if there is evidence of clinical benefit, and the Investigator believes it is in the best interest of the patient to resume CA-4948 after a hold of more than 28 days, this may be allowed after resolution of the AE. If treatment is resumed, dose of CA-4948 should be reduced by one dose level.
- Reasons unrelated to toxicity (e.g., intercurrent illness or elective surgery) at the discretion of the Investigator
    - Hold CA-4948 dosing for up to 28 days. If there is evidence of clinical benefit and the Investigator believes it is in the best interest of the patient to resume CA 4948 after a hold of more than 28 days.

If a patient experiences other grade 3/4 non-hematologic toxicity considered at least possibly related to CA-4948, the CA-4948 treatment will be interrupted until toxicity resolves to ≤ Grade 1. Missed doses of CA-4948 will not be made up.

Dose adjustments for hematologic toxicities can be find in 18.6.6.

[26] SAEs due to leukemia progression (i.e. pancytopenia, anemia, leukopenia/neutropenia, thrombocytopenia, neutropenic fever, neutropenic infections, thrombocytopenic bleedings) are excluded from DLTs.

Included patients will undergo regular hemoglobin measurements. If hemoglobin levels drops below a predefined threshold (per institutional guidelines), the patient will receive RBC transfusions and the dosage will be adjusted if necessary.

6.5.1.3 Early Stopping Rules

The design allows stopping a cohort for futility after documentation of response in 21 patients (see chapter 9).

6.5.1.4 Schedule of Visits and Assessments

See Schedule of Assessments and Procedures for overview (page 18).

6.5.1.4.1 Screening

All Screening assessments will occur within 28 days prior to administration of the first dose of CA-4948. Re-screening of patients will be allowed.

6.5.1.4.2 Treatment

CA-4948 will be administered BID on days 1-21 of repeated 28-day cycles. Clinic visits will occur on Days 1, 8 and 15 during Cycle 1 and Cycle 2, on Day 1 and 15 for Cycles 3 and 4, and on Day 1 every other cycle starting at Cycle 5. An End of Treatment Visit will occur within 7 days after the last dose of CA-4948.

6.5.1.4.3 Follow-up

All patients will undergo a Safety Follow-up Visit 28±3 days following the last dose of CA-4948. Patients who discontinue treatment for reasons other than PD will continue to undergo tumor imaging/response assessments as outlined in the Schedule of Assessments and Procedures (page 18). Survival status will be confirmed every three to six months until death or for up to two years from the start of CA-4948, whichever occurs first

6.5.2 Compliance

At each visit (first day of the cycle), the patients receive the amount of the trial medication required until the next appointment (including a reserve). The patient notes the intake in the patient diary (to be provided by ZKS Leipzig). The investigator examines the record at each visit and takes back empty packages or unused medication. The monitor appraises the drug accountability and thus compliance of the patients as part of the on-site checks.

6.5.3 Dealing with Side-effects

The only specific toxicity to CA-4948 is rhabdomyolysis.

Prevention: avoid or reduce contributing risk factors including vigorous and exhausting physical work-outs. Avoid dehydration. If rhabdomyolysis grade ≥ 2 occurs, interrupt treatment until recovery, provide hydration, prevention or treatment according to institutional guidelines. Resume treatment after recovery to Grade 1 at lower dose level (reduction by one dose level, see table 2 in section 6.5.1.2).

Additional assessment of potential risks:

If probability of Tumor lysis syndrome (TLS) is high - follow institutional guidelines. If concomitant treatment with very strong CYP 3A4 inhibitor is indicated (example anti-fungal

azoles), CA-4948 accumulation is possible. Consult with the sponsor. Recommendations for identification and management of TLS can be find in chapter 18.6.3 and 18.6.4.

6.5.4 Alternative Medication

Only the study medication may be administered.

6.5.5 Concomitant Medication

Concomitant medications and treatments will be collected from Screening through the Safety Follow-up Visit.

All prior and concomitant anti-cancer therapy for patients' advanced disease will be collected, including chemotherapies, immunotherapies, radiation, surgery, stem cell transplant, biologics, and experimental treatments.

6.5.6 Drug Interactions/Precautions

CA-4948 was found to be a substrate of P-glycoprotein (P-gp). Therefore, medications known to be inhibitors of P-gp should be used with caution. See the following website for a list of drugs known to be P-gp inhibitors. *NOTE: The list on this site is not meant to be exhaustive, check site frequently for updates:*

https://www.fda.gov/drugs/developmentapprovalprocess/developmentresources/druginteractionslabeling/ucm093664.htm.

Initial studies demonstrate that CA-4948 is not an inhibitor of CYP450 enzymes in human liver microsomes suggesting that CA-4948 should have minimal metabolic drug-drug interactions. Strong CYP 3A4 inhibitors (e.g., anti-fungal azoles) should be used with caution as they may lead to CA-4948 accumulation.

6.5.7 Counterindicated/Forbidden Concomitant Medication

- Other anti-cancer agents and investigational agents, including chemotherapy, immunomodulatory therapy are prohibited for 14 days prior to the first dose of CA-4948 and throughout the duration of the study treatment.

- Current or planned glucocorticoid therapy, with the following exceptions:

    a) Doses ≤10 mg/day prednisolone or equivalent is allowed, provided that the steroid dose has been stable or tapering for at least 14 days prior to the first dose of CA-4948
    b) Inhaled, intranasal, intraarticular and topical steroids are permitted

- Non-oncologic live-attenuated vaccines are prohibited for 28 days prior to the first dose of CA-4948, during treatment, and for three months after the last dose of study treatment.

- The following medications are prohibited for 14 days prior to the first dose of CA-4948 during the study, and up to the Safety Follow-up Visit:

a) Erythropoiesis-Stimulating Agents (ESAs), Thrombopoietin receptor agonists (TPO-RAs)
b) Lenalidomide
c) Luspatercept
d) Medications which, in the opinion of the investigator, have a high risk of causing prolonged QTc and/or Torsades de Pointes (see crediblemeds.org for a list of drugs that may prolong QT by risk category)
e) Pegfilgrastim or equivalent

6.5.8 Permitted Supportive Therapy

Unless otherwise prohibited (Section 6.5.7), supportive therapy for optimal medical care may be administered per institutional standard of care at the study centers. Efforts should be made to keep all other concurrent medications at stable doses during the study and to refrain from starting any new medications, unless clinically indicated. Specifically, the following should be considered:

- Antiemetic therapy may be considered to prevent or control nausea and vomiting prior to or after each dose of CA-4948.

- Drugs for the treatment of diarrhoea (any grade) are used according to common routine. .

- Red blood cell transfusion or platelet transfusion support.

6.5.9 Contraception Methods

Women of childbearing potential[27] and men who partner with a woman of childbearing potential must agree to use at least one of the below mentioned contraceptive methods which, in the Investigator's opinion, are highly effective and adequate for the patient's circumstances while on CA-4948 treatment and up to 90 days after last treatment.

Highly effective methods of contraception for this study include:

- Hormonal implanted contraceptive (initiated at least 1 month prior registration)
- Birth control pills (initiated at least 1 month prior registration)
- Intrauterine device
- Birth control patch
- Hormonal injected contraceptive (initiated at least 1 month prior registration)
- Sterilization
- Abstinence (if in line with the patient's usual and preferred lifestyle)
- Barrier method (condom or diaphragm/cap with spermicide) plus hormonal contraception/an intrauterine device

---

[27] Women are considered of child bearing potential unless they are surgically sterile (have undergone a hysterectomy, bilateral tubal ligation, or bilateral oophorectomy) or are postmenopausal (at least 12 consecutive months with no menses without an alternative medical cause).

6.5.10 Overdose and Abuse

No specific information is available on the treatment of CA-4948 overdose.

In the event of an overdose, the coordinating investigator and sponsor should be immediately notified. The patient should be carefully monitored for potential AEs and symptomatic treatment instituted as per institutional standards of care. Continued study treatment may be decided at the discretion of the investigator in consultation with the Principal Investigator and Sponsor.

7 INDIVIDUAL TRIAL PROCEDURES

7.1 Patient Information and Informed Consent

An authorised trial physician will seek the patient's consent before performing any trial specific medical procedures with the patient. Patients who are considered not competent to consent to participate, e.g. due to an insufficient level of understanding, cannot participate in the trial.

In accordance with international guidelines, the informed consent of trial participants will be in writing (written, dated and signed by the person performing the interview referred to below, and by the subject).

The patient's consent must refer explicitly to the collection and processing of health-related data. Therefore the patient should be informed explicitly about the purpose of collecting the data and scope of what is to be collected and that personal data, including health related data, will be stored and used for analyses in a pseudonymized form.

Before obtaining informed consent, the potential trial participant will receive information regarding the clinical trial in an interview. It will be performed by a qualified medical member of the trial group authorised by the investigator for this task.

The interview has to cover the following items:

- the nature, objectives, benefits, implications, risks and potential inconveniences of the clinical trial
- the expected duration of the subject's participation in the clinical trial
- the information that the patient may withdraw his/her consent to participate at any time without giving reasons. The patient is to be informed that in case of revocation of his/her consent, the stored data may be used further, as may be necessary to
    - assess effects of the medicinal product being tested,
    - guarantee that the patient's personal interests are not adversely affected,
    - comply with the requirement to provide complete authorisation documentation.
- potential treatment alternatives
- follow-up measures in case of early termination of the trial for the patient or overall
- the applicable damage compensation system in case of damage to a patient
- the right on data access, rectification and withdrawal of personal data

The subject will have the opportunity to ask questions at any moment.

Adequate time will be provided for the subject to consider his or her decision.

7.1.1 Withdrawal of Informed Consent

Patients may withdraw their consent to participate at any time without giving reasons. Nevertheless, the patient should be asked for the reason of the premature termination after being informed that he does not need to do so. Information as to when and why a patient was registered and when he withdrew consent must be retained in the documentation.

The patient is to be informed that in case of revocation of his/her consent, the stored data may be used further (according to § 40 lit 2a sublit 3 to 5 German Medicinal Products Act), as may be necessary to

- assess effects of the drug being tested,
- guarantee that the patient's personal interests are not affected adversely,
- comply with the requirement to provide complete authorisation documentation.

Data no longer required for the aforementioned purposes shall be deleted immediately.

7.2 Enrolment in the Trial

Patients will be included in the trial in a two-step procedure:

1. Registration for the study with informed consent, preliminary check of inclusion/exclusion criteria, and initiation of study-specific screening investigations (see 5.1).

2. Formal inclusion with a final check of inclusion and exclusion criteria (see 5.2) after completion of all study-specific screening investigations at the end of the baseline evaluation period and shortly before start of treatment.

Registration:

Patients will be identified by participating trial sites. Once potential patients are identified, they will be asked for trial participation and informed consent by investigators of the LUCAS trial. If the patients are eligible for the trial according to the eligibility criteria and willing to participate, they can be registered. Patients should only be registered if valid baseline data are expected to be available at the end of the baseline evaluation period.

A web-based registration will be performed. The trial staff enters the data relevant for registration (e.g. date of registration and date of informed consent) into the database and will receive a registration confirmation afterwards. The registration confirmation will be sent to the trial site as an automatic e-mail and/or fax for filing in the investigator site file (ISF).

In cases, when the internet-based tool is not available (e.g. server shut down, internet connection blocked, etc.), the registration form of the eCRF can also be filled in by hand and sent to the ZKS Leipzig by e-mail (scan) or fax.

Formal inclusion:

The patient can be included if the following conditions are met:

a) final check of inclusion and exclusion criteria (see 5.2) after completion of all study-specific screening investigations and shortly before start of treatment.

b) confirmation of the inclusion criteria (IC 1: diagnosis, IC 2: IPSS-R) received from the central laboratory (see chapter 13) and

c) mandatory documentation of the following minimum data in the study database for the whole baseline evaluation period (16 complete weeks (MO to SU) before day of inclusion):

- complete transfusion history
- complete Hb-measurement time course with at least five valid (i.e., not within 7 days after a transfusion) Hb measurements that span at least 100 days.

During the inclusion, a formal algorithm will check validity of this data in order to confirm symptomatic anaemia and make sure the baseline data suffice to assess response therapy later.

Once conditions a-c are met, the patient can be formally included. The recruiting site receives a confirmation that treatment can start and are informed about the baseline Hb level and RBC-transfusion count as a reference for response assessment.

7.2.1 Discovery of a Violation of the Eligibility Criteria after the Fact

In general, the violation of eligibility criteria is not a reason for premature withdrawal of the patient from the trial therapy or from the whole trial.

If a violation of a selection criterion is discovered after registration of a patient, this has to be documented in the eCRF, which will result in an automatic report to the responsible trial team members at the ZKS Leipzig.

After consultation with the coordinating investigator and biometrics the project management of the ZKS Leipzig informs the investigator/deputy or authorised medical staff immediately regarding the further treatment of the patient. The patient's data will continue to be recorded unless the patient revokes his/her informed consent. For procedures after premature trial termination of a single patient see chapter 7.5.

If, after inclusion of the patient in the study, the patient is found to violate any criteria related to safety, we will proceed as follows:

If the violation of an inclusion/exclusion criterion is discovered after inclusion in the trial but BEFORE start of CA-4948 treatment, then the patient will be excluded from the study and will be replaced. If a patient who is already being treated with CA-4948 is found to have violated an inclusion/exclusion criterion, then this aspect will be discussed between the coordinating investigator (LKP) and the treating investigator immediately after it becomes known. Patient safety has the highest priority in the decision on the further treatment of the patient. If this is endangered, the therapy is discontinued (end of treatment). If it was only determined after completion of the therapy that a criterion for inclusion was not fulfilled, the patient will be followed up until end of study.

7.3 Description of the Treatment Procedures

This study is divided into three periods: Screening, Treatment, and Follow-up. A description of individual study procedures is presented in the following sections.

7.3.1 Screening and Baseline Procedures and Assessments

During the 28 days prior to start of dosing, the following Screening procedures will be performed:

- Review inclusion/exclusion criteria for patient eligibility and sign the informed consent form (ICF)
- Obtain patient demographics and medical history (including genetic and molecular tumor testing results, when available, family history, bleeding history, and prior/concomitant medication data)

- Conduct complete physical examination including weight and height and vital sign assessment
- Perform clinical laboratory tests (hematology, serum chemistry, coagulation parameters, urinalysis and thyroid function test)
- Perform Electrocardiogram, ECG (performed in triplicate, after supine for 5 minutes)
- Perform serum pregnancy test (for women of childbearing potential)
- Perform HBV/HCV testing (only in patients with a history)
- Assess ECOG performance status
- Aspirate for morphologic evaluation (bone marrow biopsy if possible for concomitant research)
- Cytogenetics
- Central lab: cytomorphology
- Evaluate extent of disease
- Document Concomitant medications
- Evaluate Quality of life (EORTC QLQ-C30 and EORTC QLQ-FA12)
- Perform activity tests
- *(optional)* samples for concomitant reasearch

7.3.2 Treatment Period

If screening assessments were performed within 3 days of Cycle 1 Day 1, they do not need to be repeated, with the exception of vital signs.

During the treatment period, all assessments will be performed **prior to daily** administration of CA-4948, unless otherwise specified. Pre- and post-dose assessments are for the morning dose of CA-4948. The following procedures will be conducted according to the Schedule of of Assessments and Procedures):

- Check of inclusion/exclusion criteria (only bevor start of first treatment)
- Conduct physical examination, only symptom-directed examination (excluded day 8 of cycle 2)
- Measure weight on first day of each cycle
- Documentation of vital signs, including temperature (oral), heart rate, blood pressure (excluded day 8 of cycle 2)
- Perform clinical laboratory tests:
    - Hematology (C1 and 2: day 1, 8, 15; complete set for C3-Cn only on D1 but Hb measurement should be performed for C3-Cn on D1 and D15)
    - serum chemistry (C1 and 2: day 1, 8, 15, and on day 1 for C3-Cn)
    - coagulation parameters (C1: day 1, 8, 15; C2: day 1 and 15; and on day 1 for C3-Cn)
    - urinalysis (on first day of each cycle, and for cycle 1 additionally on days 8 and 15)
    - thyroid function test (on first day of C2-Cn)
- Perform ECG (on C1D1 and C2D1)
- Perform pregnancy test (for women of childbearing potential, on first day of each cycle)
- Assess ECOG performance status (on first day of each cycle)
- Document concomitant medications

- AE assessment
- Dispense CA-4948 at first day of each cycle
- Check of patient diary to check for compliant CA-4948 dosing (on first day of each cycle)
- Evaluate Quality of life (EORTC QLQ-C30 and EORTC QLQ-FA12): on first day of each cycle
- Perform activity tests (on first day of each cycle)
- *(optional)* samples for concomitant reasearch

7.3.3 Primary endpoint visit

Primary endpoint visit should be performed after four treatment cycles and corresponds for responders to day 1 of cycle 5 and for non-responders to the end of treatment-visit. The primary endpoint visit includs the following assessments:

- Perform clinical laboratory tests: hematology
- Cytogenetics local
- Central lab: cytomorphology
- Evaluate extent of disease/response to treatment
- AE assessment
- *(optional)* samples for concomitant reasearch

7.3.4 End of Treatment

End of treatment visit should be performed seven days after last CA-4948 dose. The visit is equivalent to primary endpoint for non-responders. The end of treatment visit includes the following assessments:

- Conduct physical examination
- Measure weight
- Documentation of vital signs (temperature (oral), heart rate, blood pressure)
- Perform clinical laboratory tests: hematology, serum chemistry, coagulation parameters and urinalysis
- Perform pregnancy test (for women of childbearing potential)
- Assess ECOG performance status
- Cytogenetics local: only for reponders; For Non-responders the bone marrow assessment is performed at primary endpoint visit that coincides with the EoT.
- Central lab: cytomorphology: only for responders; For Non-responders the bone marrow assessment is performed at primary endpoint visit that coincides with the EoT.
- Document Concomitant medications
- AE assessment
- Check of patient diary to check for compliant CA-4948 dosing
- Evaluate Quality of life (EORTC QLQ-C30 and EORTC QLQ-FA12)
- Perform activity tests
- *(optional)* samples for concomitant reasearch

7.3.5 Safety Follow-up visit

Safety follow-up visit should be performed 28±3 days after last CA-4948 dose, including the following assessments:

- Conduct complete physical examination
- Measure weight
- Documentation of vital signs (temperature (oral), heart rate, blood pressure)
- Perform clinical laboratory tests: hematology, serum chemistry, coagulation parameters, urinalysis and thyroid function tests
- Assess ECOG performance status
- Document Concomitant medications
- AE assessment
- Evaluate Quality of life (EORTC QLQ-C30 and EORTC QLQ-FA12)
- Perform activity tests

7.3.6 Survival follow up

Survival follow-up will be performed by telephone in three to six months intervals for up to two years to request survival status and treatment-related AEs/SAEs (including follow-up of AEs/SAEs until resolution, return to baseline, or until they are ≤ Grade 1).

7.4 Description of examinations

Time points for all of the assessments are given in table chapter 1.

Demographics and Medical History

Demographics and medical history will be obtained by the investigator or designee during the screening period. Demographics will include date of birth, sex, ethnicity, and race.

Relevant medical history should be recorded and should include prior/current medical conditions, including specific diagnosis, abnormal physical exam findings or clinically significant laboratory abnormalities (excluding study disease-related abnormalities) from the baseline assessment. Furthermore concomitanat medication should be documented.

Physical Examination

A complete physical examination will be performed only at screening and safety follow-up visit. Symptom-directed examination is sufficient for all other visits.

Weight and Height

Weight will be measured at screening visit, first day of each cycle, end of treatment visit and at safety follow up. Height will be captured at screening visit only.

Vital Signs

Vital signs including temperature (oral), heart rate, and blood pressure (seated 5 minutes) will be assessed during screening, during treatment period, at the end-of-treatment visit, and at the safety follow-up visit.

During treatment, vital signs, including temperature (oral), heart rate, blood pressure (seated 5 minutes) will be collected pre-dose at each visit marked in the schedule of events; AND post-dose also on Day 1 of Cycle 1 (15 min, 30 min, 1 hour, 2 hours, and 4 hours; ± 5 minutes) and Day 1 of Cycle 2 (1 hour ± 5 minutes).

Clinical Laboratory Tests

Local laboratories will perform all clinical laboratory tests and results will be provided to the investigator. Samples will be collected at scheduled study visits **before** the administration of CA-4948 unless otherwise noted, and more frequently if clinically indicated.

Hematology laboratory include complete blood count (CBC) with differential, platelet count and fibrinogen.

Complete blood count includes the following:

- Red blood cells (RBC)
- red blood indices [MCH, MCV, MCHC]
- hemoglobin (HGB): Hb measurement needs to be performed for all cycles on day 15 additionally.
- hematocrit (HCT)
- leukocytes (WBC) [neutrophils, lymphocytes, monocytes, eosinophils and basophils],
- thrombocytes (PLT)
- absolute neutrophil counts (ANC)

Serum chemistry tests include sodium, potassium, chloride, calcium, inorganic phosphate, magnesium, blood urea nitrogen, creatinine, glucose, bilirubin (direct and total), AST, ALT, ALP, albumin, globulin, total protein, triglycerides, cholesterol, lipase, amylase, troponin, aldolase (only, if CK is not measured), lactate dehydrogenase and creatine kinase (CK) and uric acid. Isoenzymes CK-MB and CK-MM will be required if total CK or Myoglobin is elevated and/or when clinically indicated. Serum EPO levels are measured during screening procedure.

Coagulation parameters include partial thromboplastin time (PTT), and prothrombin time (PT) or international normalized ratio (INR).

Urinalysis will evaluate protein, glucose, ketones, urobilinogen, occult blood, and when indicated by dipstick abnormality, microscopic sediment evaluation.

Thyroid function tests will evaluate thyroid stimulating hormone (TSH) and free T4 (FT4). Laboratory tests will be performed at the time points specified in the Schedule of Assessments and Procedures.

Electrocardiogram

Triplicate ECG (after supine for 5 minutes) will be recorded at Screening, at pre-dose and 3 hours (± 10 minutes) post-dose on Cycle 1 Day 1 and Cycle 2 Day 1.

Cytogenetic analyses (local lab)

The cytogenetic analysis with banding analysis (optional FISH) should be performed at local labs. Therefore 2 - 5 mL of bone marrow will be collected and analyzed. If the cytogenetic

analysis was unsuccessful (this means that cytogenetic examination has been performed on BM or on blood material, but the result was considered as a failure; e.g. NN with < 10 mitoses), analysis must be repeated.

Bone marrow (BM) analyses and CBC with differential (central morphology, Düsseldorf)

Bone marrow analyses and CBC with differential will be performed by a central laboratory in Düsseldorf / Germany. For this purpose 5 unstained bone marrow slides and 2 peripheral blood slides will be sent to the central lab at screening, primary endpoint assessment and at EoT.

Pregnancy Test

Pregnancy tests will be performed for women of childbearing potential. A serum pregnancy test must be used at screening and must be negative for enrollment. Serum or urine pregnancy tests may be used on Day 1 of each cycle and at the End of Treatment Visit. A positive result observed following enrollment should be confirmed with a repeated serum pregnancy test. Pregnancy tests may be performed more frequently as clinically indicated.

HBV/HCV Testing

In patients with a history of HBV, HBcAb testing is required and if positive, then HB DNA testing will be performed. In patients with a history of HCV <6 months prior to start of CA-4948, viral load testing is required.

ECOG Performance Status

ECOG performance status (scale provided in 18.6.1) will be assessed on Screening, Day 1 of each treatment cycle, End of treatment visit and Safety Follow-up visit.

Prior and Concomitant Medications and Treatments

All prior anti-cancer therapies (chemotherapy, immunotherapy, radiation, surgery, stem cell transplant, biologics, and experimental treatments) will be recorded under section medical history. All concomitant medications used from start of treatment with CA-4948 up to the Safety Follow-up Visit, including treatments for AEs, will be recorded.

Adverse Events

Monitoring and recording of AEs will be conducted throughout the study. AEs will be captured on the electronic case report forms (eCRFs) from the first day of CA-4948 dosing until the Safety Follow-up visit. Only treatment-related AEs will be captured from Safety Follow-up visit (28±3 days following the last day of study treatment) until the End of Study. Treatment-related AEs/SAEs that are ongoing at the time of Safety Follow-up Visit should continue to be followed until resolution, return to baseline, or until they are Grade ≤1. Definitions, documentation, and reporting of AEs are described in detail in Section 8.

Activity assessment

Various scales are used in the assessment of activity. For example Timed „Up & Go“ [36] and IADL (Instrumental Activities of Daily Living). In addition, the MDS specific frailty index will be perfomed including a grip strength test, a 4-m walk test and a timed ten-times chair stands test [37].

7.4.1 Efficacy Evaluations

7.4.1.1 Baseline Disease Assessment

Disease evaluation for MDS will be performed during Screening including evaluation of blood chemistry and complete blood count, including platelets and differential, bone marrow aspirate and cytogenetics. The patient’s most recent bone marrow aspirate samples, either archived or fresh, and pathology report must be obtained for verification of diagnosis. If an aspirate is unobtainable (i.e., a “dry tap”) at baseline/screening, the diagnosis may be made from the core biopsy. Complete blood count with differential and bone marrow aspirate to evaluate the extent of disease.

7.4.1.2 Disease Response Assessments for Lower Risk MDS

The activity of CA-4948 will be assessed in accordance with 2018 Revised IWG criteria [38] for MDS. For MDS/MPN the same will be adopted with regards to HI. Patients with a response after 16 weeks of treatment will continue taking CA-4948 until disease progression, treatment failure, or dose-limiting adverse events occurred. Patients without a response who will be following the 21-day treatment schedule are offered to continue dosing.

7.5 Premature Termination of the Therapy or Follow-up

The primary statistical analysis follows the intention to treat principle as closely as possible. For a valid analysis, it is of great importance to minimise the rate of drop-outs. Therefore, in patients who do not withdraw their consent, all trial visits shall be performed as scheduled. In order to account for possible risks due to trial assessments, see Schedule of Assessments and Procedures.

In case of premature termination of therapy and/or trial, it is necessary to document the date (as exactly as possible), the reason of termination and the current condition of the patient. Therefore, the eCRF “End of study (ES)” has to be completed for each patient. Data entry to this eCRF page will trigger an automatic report to the responsible trial team members at the ZKS Leipzig.

The “End of study”- eCRF routinely contains the following data:

- Date of individual end of trial
- Reason for trial termination
- Date of death, if applicable
- Latest patient contact

7.5.1 Premature Termination of the Therapy for Individual Patients

Patients will be discontinued from further study treatment in the event of any of the following:

- Unmanageable toxicity
- Patient’s decision/withdrawal of consent (e.g., patient declines further treatment)
- Physician decision if continuation is not in the patient’s best interest
- Termination of the study by the Sponsor

- Significant protocol deviation
- Pregnancy
- Lost to follow-up
- Confirmed PD

Termination of trial therapy does not mean that the patient is off-trial.

7.5.2 Replacement of Patients

There will be no replacement of patients.

7.5.3 Premature trial termination for individual patients

All included patients will be followed up until two years after EOT. Premature termination of trial therapy does not necessarily lead to individual trial termination (see chapter 7.5.1 for explanation).

The only circumstances in which a premature trial termination (i.e. no further trial visits) of an included patient is unavoidable are:

- a patient is worsening (and not responding to treatment) that contraindicates permanently participation in any trial visits
- withdrawal of informed consent,
- complete loss of contact to the patient or
- death of the patient.

7.5.4 Premature Termination of the Follow-up for Individual Patients

Follow up will be prematurely terminanted in case of loss of contact or death of the patient.

7.6 Plan for Further Treatment

Patients will be treated as usual after the individual’s end of therapy.

8 ADVERSE EVENTS (AE/SAE)

8.1 Adverse Events (AE)

8.1.1 Definition

An Adverse Event (AE) is any untoward medical occurrence in a patient or or clinical investigation subject administered a pharmaceutical product and which does not necessarily have to have a causal relationship with this treatment (ICH Guideline E2A).

Adverse Events encompass any unfavourable and unintended sign (including an abnormal laboratory finding, for example), symptom, or disease that arise newly or worsen after the inclusion of the patient into the trial.

8.1.2 Adverse Event Grade

AE severity grading will be assessed using the NCI CTCAE (see also 18.2). For those events without assigned CTCAE grades, the recommendation on page 1 of the CTCAE that converts mild, moderate and severe into CTCAE grades should be used. A copy of the NCI CTC is available online at: http://evs.nci.nih.gov/ftp1/CTCAE/About.html.

8.1.3 Adverse Drug Reaction (ADR)

In the **pre-approval clinical experience** with a new medicinal product or its new usages, particularly as the therapeutic dose(s) may not be established: all noxious and unintended responses to a medicinal product related to any dose should be considered adverse drug reactions. The phrase "responses to a medicinal products" means that a causal relationship between a medicinal product and an adverse event is at least a reasonable possibility, i.e., the relationship cannot be ruled out (ICH Guideline E2A).

8.1.4 Documentation and Reporting

All AEs will be documented on special AE-forms from the first dose of CA-4948 until the Safety Follow-up visit (= 28 ± 3 days after last dose). Only treatment-related AEs will be captured from Safety Follow-up until the End of study visit. All treatment-related AEs/SAEs will be followed until resolution, return to baseline, or until they are Grade ≤1 until End of study.

At each visit, the patient will be asked a non-leading question such as "Have you had any health problems since the last visit". All AEs reported in response to questioning, as well as AEs reported spontaneously will be recorded on the 'adverse event' page(s) in the CRF, regardless of causality. In addition, the patient will be trained to inform the clinical trial site of any health problems arising between visits by phone or personal visit.

If an AE fulfils any of the criteria for an SAE (see chapter 8.4.1 for SAE-definition), both the AE pages of the CRF **and** the SAE form must be completed. This applies to all SAEs, **whether or not they are considered to be related to trial treatment.**

For both serious and non-serious AEs, documentation should be supported by an entry in the patient's heath record. Required information in the patient's health record should include:

- type of AE
- grade/severity/intensity acc. to CTCAE v.5.0
- seriousness (see also section 8.4)
- onset date
- end date
- actions required
- outcome
- assessment of its relationship to trial drug

All abnormal physical and/or laboratory results which are considered to be clinically relevant by the investigator should be recorded as AEs. If an abnormal laboratory result meets any of the criteria for a SAE, this must also be reported on the SAE Form.

Adverse Events are classified by their seriousness, intensity/severity and relationship to the IMP (see also 18.2)

8.2 Safety Analysis

During the course of the trial, every patient will be monitored closely. On every visit day this encompasses documenting Adverse Events.

8.3 Concomitant Diseases

Concomitant diseases will be recorded at Screening and confirmed at the first regular trial visit at day 1 of cycle 1. Any worsening of concomitant diseases will be regarded as adverse event.

8.4 Serious Adverse Events/Serious Adverse Reactions (SAE/SAR)

8.4.1 Definition

An Adverse Event/Reaction is defined to be serious according to ICH-Guideline E2A, paragraph IIB [39] §3 (8) GCP-V [40], if it

- **results in death**,
- **is life-threatening**,

  Note: The term "life-threatening" in the definition of "serious" refers to an event in which the patient was at risk of death at the time of the event; it does not refer to an event which hypothetically might have caused death had it been more severe.
- **requires in-patient hospitalisation or prolongation of existing hospitalisation[28]**,
- **results in persistent or significant disability/incapacity or**
- **is a congenital anormal/birth defect** .

Medical and scientific judgement should be exercised in deciding whether expedited reporting is appropriate in other situations, such as important medical events that may not be immediately life-threatening or result in death or hospitalisation but may jeopardise the patient or may require intervention to prevent one of the other outcomes listed in the definition above. These should also usually be considered serious.

In the present trial, the following adverse events will also be considered as serious:

- Rhabdomyolysis

  CAVE: Please report any Rhabdomyolysis event immediately regardless of severity using the paper-based SAE form (see 8.4.2)

8.4.2 Documentation and Reporting Obligations: INVESTIGATOR

Serious Adverse Events have to be documented on the SAE-forms and the investigator must report them to the sponsor immediately. If more information about the SAE becomes available later, it must also be reported to the sponsor immediately.

All SAEs will be documented on special SAE-forms from the first dose of CA-4948 until the Safety Follow-up visit (= 28 ± 3 days after last dose). Only treatment-related SAEs will be captured from Safety Follow-up visit until the End of study visit. All treatment-related SAEs will be followed until resolution, return to baseline, or until they are Grade ≤1 until End of study.

[28] In general, hospitalisation means that the patient stays (usually at least an overnight stay) at the hospital or emergency ward for observation and/or treatment. Complications that occur during the stay are AEs. If a complication prolongs hospitalisation or fulfills any other serious criteria, the event is serious.
Hospitalisation for elective treatment of a pre-existing condition that did not worsen from baseline is not considered an AE.

In the event of a patient’s death, associated event causing the death should be determined. The investigator/deputy or the authorised medical staff provides the leading ethics committee(s), all involved ethics committees in multi-centred trials, the responsible federal authorities and the sponsor with all further information needed to fulfil their tasks **upon request**.

In all the reports, personal data are to be pseudonymized by using the patient’s identification code. It must be possible to relate the initial and all follow-up reports to each other by means of the patient identification number, name and address or the like.

The investigator the deputy or the authorised medical staff must report every Serious Adverse Event (incl. death not causally related to using the trial drug*)* as soon as getting knowledge of it to the following address:

ZKS Leipzig/Pharmacovigilance
Universität Leipzig
Zentrum für Klinische Studien Leipzig
Haertelstr. 16-18, 04107 Leipzig
Phone: +49/341/97-16129
E-mail: pharmacovigilance@zks.uni-leipzig.de

Fax: +49/341/97-16278

The ZKS Leipzig will inform Curis immediately after awareness of the SAE, SUSAR (8.6) or pregnancy (8.9) by e-Mail. The pseudonymized data are additionally pseudonymized in the ZKS before being passed to Curis in the USA.

Any SAE that occurs at any time after completion of trial specific treatment or after the designated follow-up period that the sponsor and/or investigator considers to be related to any study drug must be reported to Curis: Email address: drugsafety@curis.com

8.4.3 Documentation and Reporting Obligations: SPONSOR

After the ZKS Leipzig receives the SAE, it is immediately passed on to the coordinating investigator/responsible person for medical assessment.

The coordinating investigator/responsible person forms a second medical opinion of the SAE with respect to causal relationships and the decision as to whether or not it was expected, as described in chapter 18.2.3 and 18.2.4 and forwards the second opinion to the ZKS Leipzig within two days of its arrival.

In the ZKS Leipzig the SAE data are entered into the SAE database immediately and the MedDRA coding takes place simultaneously.

Then forwarding as per law and as described in Chapter 8.6 only for Suspected Unexpected Serious Adverse Drug Reactions (SUSARs) takes place.

Details of the sponsor’s documentation and reporting obligations will be specified in a special, trial-specific pharmacovigilance plan, which will be written and finalised alongside with this protocol, if possible.

8.5 Periodic Reports

8.5.1 Annual Safety Report

According to the regulatory authorities in Germany the sponsor writes a safety report annually (Annual Safety Report, ASR[29]) and sends this report to the leading ethics committee(s) and the federal authorities.

The key date is the date of the first authorization of the clinical trial by a federal authority. All data obtained up to this date (each year) will be included in the ASR. Beginning with the key date, there is a time-limit of 60 days for the preparation and submission of the ASR.

The ASR will be prepared by the ZKS Leipzig (project manager, responsible biometrician and PV data manager) acc. to the national requirements Deadlines regarding the ASR are based on the German Medicinal Products Act.

The final report will be released in cooperation with the principal investigator/sponsor’s representative.

8.5.2 Periodic SUSAR Reports for Ethic Committees (and/or investigators)

Because of the procedures concerning SUSAR-reporting (see 8.6.2) it can be necessary that all SUSARs from other Member States have to be reported periodically at least every six months as a line listing accompanied by a brief report by the sponsor highlighting the main points of concern. These periodic reports should only include SUSARs reported within the period covered by the report.

If this is required by any of the involved member states, the SUSAR-reports will be prepared by the ZKS Leipzig (project manager, responsible biometrician and PV data manager). The final report will be released in cooperation with the sponsor´s representative.

It also can be necessary to inform the trial sites every six months about the SUSARs as a line listing instead of single reports. If this is required by any of the involved member states, the SUSAR-listing will be prepared by the ZKS Leipzig (PV data manager).

Detailed procedures will be specified in the trial specific pharmacovigilance plan.

8.6 Suspected Unexpected Serious Adverse Reactions (SUSAR)

8.6.1 Definition

Suspected Unexpected Serious Adverse Drug Reactions (SUSARs) are side-effects, the nature or severity of which are inconsistent with the information available about the product. Information about the trial product is contained in the Investigator’s Brochure.

---

[29] See “Detailed guidance on the collection, verification and presentation of adverse reaction reports arising from clinical trials on medicinal products for human use”.

8.6.2 Documentation und Reporting Obligations

Information *for SPONSOR*

The sponsor submits all information available about a SUSAR immediately to the leading ethics committee, the responsible federal authorities, and to all participating primary investigators, at the latest within 15 calendar days after the event becomes known – according to national law.

For every SUSAR that results in death or a life-threatening condition, the leading ethics committee, the federal authority and all participating investigators must be informed by the sponsor within seven calendar days after the event becomes known – according to national law. Additional information has to be given within eight further calendar days

Details of the sponsor's documentation and reporting obligations will be specified in a special, trial-specific pharmacovigilance plan, which will be written and finalised alongside with this protocol, if possible.

Information for INVESTIGATOR

The investigator passes down all relevant information concerning the SUSAR to all participating trial investigators at his/her trial centre. This has to be confirmed by the investigator in writing.

8.7 Other Safety Relevant Issues

Other safety issues also qualify for expedited reporting where they might substantially alter the current benefit-risk assessment of an investigational medicinal product and/or would be sufficient to consider changes in the investigational medicinal products administration and/or in the overall conduct of the trial, for instance:

New events related to the conduct of the trial or the development of the IMP likely to affect the safety of subjects, such as:

- a serious adverse event, which could be associated with the trial procedures and which could modify the conduct of the trial,
- a significant hazard to the subject population such as lack of efficacy of an IMP used for the treatment of a life-threatening disease,
- a major safety finding from a newly completed animal trial (such as carcinogenicity),
- a temporary halt of a trial for safety reasons if the trial is conducted with the same investigational medicinal products in another country by the same sponsor

The sponsor decides if the number of events or qualitative changes in the expected SARs comprise a safety issue and must be reported.

8.8 Therapeutic Procedures.

If a patient requires treatment as a result of an Adverse Event, then it must meet the recognized standards of medical care in order to restore the patient's health. Appropriate resuscitation

devices and medication must be available in order to treat the patient as quickly as possible in the event of an emergency.

The action taken to treat the AE/SAE must be documented by the investigator either in the appropriate CRF and/or using additional documents.

8.9 Dealing with Pregnancy

Every pregnancy that occurs while taking part in the trial must be reported to the ZKS Leipzig by the investigator/deputy or the authorised medical staff within 24 hours of having learned of it by informal communication:

ZKS Leipzig / Pharmacovigilance
Universität Leipzig
Zentrum für Klinische Studien Leipzig
Härtelstr. 16-18, 04107 Leipzig
Telefon: +49/341/97-16129
Fax: +49/341/97-16278
E-mail: pharmacovigilance@zks.uni-leipzig.de

The ZKS Leipzig will then supply the essential forms for documentation to the investigator/deputy investigator without unduly delay. Pregnancies that occur in female trial subjects or partners of male trial subjects have to be reported by using the form ”Report on the arising of a pregnancy during exposition to a trial medication”. Severe side effects and complications during a pregnancy as well as congenital birth defects are Serious Adverse Events by definition and therefore also have to be reported on the Serious Adverse Event form according to the reporting procedures described above.

The outcome of a pregnancy has to be reported on the form “Report on the outcome of a pregnancy during/after exposition to a trial medication”. This form will document the outcome of the pregnancy, including a spontaneous or voluntary abortion, details of the birth process, the presence or absence of congenital malformations and birth defects, maternal or foetal complications and the potential relationship to the trial drug.

Collecting data concerning the outcome of a pregnancy is only permitted if the trial subject and the partner of the male trial subject puts down her permission in writing beforehand. The collection of data on the child's health requires the prior information and consent of the guardian/parents (usually both).

9 BIOMETRY

9.1 Biometrical Aspects of the Trial Design

Each of the two cohorts constitute an independent substudy.

For each substudy, we use the optimal Simon 2-stage Phase II design: [41] separately with up to 42 patients in each of the two cohorts, thus up to 84 patients will be included.

The design for both cohorts is based on the following specifications:

Response rate to be rejected: $\pi 0 = 0.05$ (Nullhypothesis H0: $\pi \le \pi 0$)

Response rate not to be missed: $\pi A = 0.20$

Error rates: $\alpha = 0.05$ probability to declare an inactive drug (response probability $\pi 0$) promising

$\beta = 0.1$ probability to dismiss an active drug that has response probability πA

For each of the two cohorts, we plan to enroll N=42 patients in two stages, with the option to stop early for futility after the first 21 patients have their response documented:

Stage 1: dismiss CA-4948 in the respective substudy if less than two responses are documented in the first 21 patients included.

Stage 2: (only if 2 or more responses from stage 1): dismiss CA-4948 in the respective cohort if response less than five responses are documented in all 42 patients. Otherwise reject the null-hypothesis

This design has the following properties:

Expected sample size EN ($\pi 0 = 0.05$) = 26.7

Probability of early stopping if Nullhypothesis $\pi 0 \le 0.05$ is true: $\ge 72\%$

With 5 or more responses out of 42, the Nullhypothesis H0: $\pi \le \pi 0$ is formally rejected (one-sided test) and CA-4948 declared promising.

Note: Treatment duration is 16 weeks. Acrual will be about 1-2 patients per month. When 21 patients have been included in a substudy and it is not yet clear that the study goes to the second stage, we will not stop accrual in order not to prolong the study duration.

9.2 End Points

9.2.1 Primary Efficacy End Point

The primary efficacy endpoint is erythroid response (HI-E) according to IWG 2018 criteria [1].

Implemening IWG 2018 Hb-response criteria in a clinical trial requires some modifications. Mostly, IWG 2018 rules are only specified further to make them technically operational, unambiguous, and to handle data problems expected in real world trial data.

The main deviation from IWG 2018 is the introduction of so-called sliding time windows over which Hb-measurements can be averaged not only for the determination of the baseline Hb-level, but also to determine Hb-response for NTD patients. This deviation makes the response definitions statistically robust and markedly increases the power to detect a true response (Hasenclever et al in preparation).

For an outline see Appendix 18.1 and for further details see the statistical analysis plan.

In determining the response rate, patients who do not complete the treatment or are not assessable for response after treatment count as failures.

9.2.2 Secondary efficacy End Points

- HI-E response duration
- Time to HI-E
- Frequency of RBC transfusions in transfusion dependent patients
- Neutrophil and platelet (HI-N and HI-P) responses according to IWG 2018 criteria [1]

9.2.3 Safety End Points

The Safety profile of CA-4948 will be described by collecting AE and SAE information.

9.3 Statistical Methods

9.3.1 Analysis Population

The analysis set consists of all formally included patients that have taken any treatment medication. Patients who withdraw before any drug exposure may be replaced.

9.3.2 Planned Methods for Analysis

The test of the nullhypotheses – separately by cohort - is determined by the Simon design.

The response rate in each cohort will be estimated with an exact two-sided 95% confidence interval.

The response rate will be described by initial transfusion dependency status. Time course of Hb and transfusions will plotted for all patients.

9.4 Statistical Monitoring

The trial conduct will be closely supervised by means of central and statistical monitoring according to ICH E6(R2) [42]. The objectives are

- to detect safety relevant signals as soon as possible
- to detect non-compliance and relevant protocol violations and to prevent their future occurrence by prompt reaction
- to prevent missing visits or measurements by prompt reminders

Therefore, the following issues will be monitored:

- With regard to safety
  - Patients with treatment discontinuation due to adverse events
  - Serious adverse events
- With regard to protocol compliance
  - Patients with major or minor findings concerning the informed consent documents as detected by on site monitoring
  - Patients with treatment discontinuation
  - Patients with other protocol violations as detected e.g. by on site monitoring
- With regard to missing information
  - Missing visits or visits outside schedule
  - Missing values with respect to central laboratory measurements

This is not an exclusive list. Statistical monitoring will be continuously adapted in response to new problems/risks which might arise during trial implementation.

Detailed information about the statistical and central monitoring incl. processes, content, endpoints, data to observed, time lines, reports and descriptive statistics will be given in a central monitoring plan.

9.5 Interim Analysis

The Simon design implies interim analyses in each cohort after the first 21 patients have their response documented.

9.6 Final Analysis

The Simon design implies that final analyses in each cohort are performed after all 42 patients have their response documented.

10 CONCOMITANT SCIENTIFIC PROJECTS

Previous studies of the pathogenesis of MDS focused primarily on the genetically altered hematopoietic stem and precursor cells (HSPCs), rather than selection pressures conducive to the emergence and malignant propagation of this clone. Acquired somatic mutations and genetic aberrations may result in propagation of MDS clones by selectively activating pro-inflammatory cytokine responses in the bone marrow. Thus, targeting of critical innate immune pathways at an early stage may prevent or delay disease progression. In particular, strategies to inhibit NLRP3 (nucleotide-binding domain and leucine-rich repeat pattern recognition receptor 3) inflammasome activation may offer therapeutic potential in lower-risk MDS patients with myeloid-driven innate inflammatory signature. In these patients, excess alarmin signals (e.g. S100A9) and founder gene mutations in MDS HSPCs appear to license a common NLRP3 inflammasome platform that directs caspase-1 (CASP1)-dependent generation of mature interleukin-1β (IL-1β) and IL-18, and pyroptotic cell death. Consequently, NLRP3, CASP1, IL-1β, IL-18, IRAK1, and IRAK 4 are overexpressed and/or over-activated in MDS.

Binding of secreted IL-1β to IL-1 receptor 1 (IL-1R1) triggers a signaling cascade (including IRAK4 signaling) that controls gene expression of multiple transcription factors, chemokines, pro-inflammatory cytokines, interleukins and growth factors involved in hematological function and modulation of hematopoietic stem cell function. An IL-1-rich environment may augment the selective pressure in the stem cell niche and support the selection and expansion of leukemic over non-leukemic stem cells in lower-risk MDS. Thus, therapeutic targeting of overactive IL-1 signaling by IRAK4 inhibition may enhance normal hematopoiesis while inhibiting pre-/leukemic clones.

Hypothesis: We hypothesize that those lower-risk MDS patients with over-activation of IRAK4 signaling and inflammasome-driven myeloid inflammation may benefit from CA-4948 treatment.

Aim: We propose a translational research project comprising comprehensive immune, molecular and functional profiling in order to identify cellular and molecular biomarkers predictive of treatment response to CA-4948.

Biobanking: The biobanking of study samples in Leipzig will establish a centralized resource from which material will be distributed both for clinical analyses (morphology, pathology, NGS mutation analysis) and translational studies (see below). Sampling packs will be prepared and distributed and the subsequent shipping, processing and controlled storage of all samples centrally coordinated. The extensive analyses described below will focus primarily on samples from the pretreatment (screening), primary endpoint and end of treatment time points. Interim samples will be banked for validation and confirmational studies.

Aim 1) Analysis of IRAK4 pathway and immune profiling in bone marrow

A complexity of interactions between immune cell subsets that may act to both regulate and stimulate inflammation, depending on cellular and molecular context. In looking for markers predictive of response to IRAK4 inhibition, we will therefore explore both cellular composition and gene expression states in detail.

Aim 1.1) Analysis of changes in the composition of the immune cell repertoire (Leipzig, Dresden, London)
To identify predictors of response to CA-4948, we will monitor cellular immune responses within the bone marrow and peripheral blood before and during CA-4948 therapy. Frequency and phenotype of key myeloid and lymphoid immune cell populations as well as HSPCs will be analyzed using conventional multiparameter flow cytometry and/or CyTOF.

Aim 1.2) Transcriptome profiling of bone marrow samples (Leipzig, Dresden)
The immune landscape of bone marrow samples will also be explored using the nCounter Analysis System from NanoString Technologies. Generation and analysis of a large set of transcriptomic data from a defined cohort of MDS patients before and during CA-4948 therapy will provide further novel insights into the impact on CA-4948 on bone marrow-associated immune cells. For transcriptome profiling of bone marrow samples, the PanCancer Immune Profiling Panel of NanoString Technologies will be used to perform multiplex gene expression analysis of 770 genes including checkpoint inhibitors, chemokines, cytokines and signal transduction pathways (with Marc Schmitz, Dresden).

Aim 1.3) Analysis of IRAK4 pathway-related gene expression in sorted subpopulations (Leipzig)
Based on the results of Aim 1.1 and 1.2, subpopulations will be sorted by FACS from bulk bone marrow MNCs. The sorted subpopulations will be analyzed by validated TaqMan® Arrays for expression of genes encoding distinct inflammation- and IRAK4-related proteins (Leipzig).

Aim 1.4) Regional distribution of the immune landscape of bone marrow samples (Dresden)
In addition, the regional distribution of various immune cell subsets and progenitor cells in bone marrow tissues of selected patients can be evaluated before and during CA-4948 therapy by multiplex immunofluorescence staining utilizing the Vectra Imaging Platform. The different staining protocols will provide detailed information about frequency and phenotype of CD4+ and CD8+ T lymphocytes, regulatory T cells, macrophages and progenitor cells

Aim 2) Phosflow™ functional analysis of bone marrow MNCs (Leipzig)

The direct impact of CA-4948 is expected to be primarily on those cells that possess an intact and active TLR – IRAK – NFkB/MAPK signaling pathway. The determination of pathway activity at the single cell level by Phosflow™ analysis, combined with staining for surface markers, should therefore reveal which cell types in which patients are potential targets of CA-4948 inhibition. Combined Phosflow™ and surface marker analysis will be performed on unstimulated cells, those stimulated with recombinant S100 proteins and stimulated cells treated with CA-4948. The patient-specific *ex-vivo* data on pre-treatment, TLR-dependent signaling activity in specific cell subsets and on sensitivity to CA-4948 will be compared retrospectively to those on clinical response to investigate the potential of Phosflow™ analysis as a prognostic marker for future patient stratification.

Aim 3) Secretory signature as predictor of response to CA-4948 (Leipzig, Dresden)

To evaluate secretory predictors of response to CA-4948 in the inflammatory bone marrow milieu, we will quantify pro-inflammatory cytokines (e.g. IL-1β, IL-6, IL-18, IL-17), chemokines (e.g. CCL2, CCL3, CCL4, CCL5, CXCL1, CXCL4, CXCL8, CXCL12), and danger-associated molecular pattern (DAMP) molecules (S100A8/A9, HMGB1) in bone marrow and paired peripheral blood plasma samples using multiplex immunoassay Luminex^{TM} technology and conventional ELISA kits.

Aim 4) Molecular monitoring by Next Generation Sequencing (NGS, Leipzig)

Longitudinal monitoring of the clonal landscape in blood, marrow, and saliva samples of these patients will be performed by deep NGS using a panel of commonly mutated genes in MDS. We will trace clonal evaolution before, during and at the end of treatment by sensitive targeted NGS, and evaluate potential resistance mechanisms of treatement-emergent clones using whole-exome/genome and transcriptome sequencing.

11 ETHICAL, LEGAL AND ADMINISTRATIVE ASPECTS

11.1 GCP-Statement

All persons participating in the conduct of the trial (sponsor, authorised representative of the sponsor, investigators, etc.) commit themselves to observe the Declaration of Helsinki of the WMA (in its current version), as well as all pertinent national laws and the ICH guideline for Good Clinical Practice (GCP) ICH E6(R2) (EMA/CHMP/ICH/135/1995) issued in June 2017.

11.2 Initial Submission

11.2.1 Submission to the Ethics Committee(s) and Competent Authority

Prior to submitting the trial related documents to the leading (and involved) ethics committee(s) and the responsible competent authority, the sponsor must enter the trial into the European database for clinical trials (EudraCT).

Afterwards, the protocol and all other associated documents according to GCP-V § 7 will be submitted to the leading ethics committee (EC) for approval. In parallel, each participating EC is informed of the submission and also receives a copy of the documents including those of the trial sites, which they have to approve. At the same time the study documents will be submitted to the responsible competent authority (CA, BfArM) according to the requirements of GCP-V § 7[40]/the applicable national requirements.

The trial can start only after obtaining a positive review by the leading ethics committee and approval from the responsible federal authority. Both written approvals must be filed in the trial master file (TMF). Additionally, every participating centre must receive a copy of these documents to be filed in the investigator site file (ISF).

11.3 Protocol Amendments

Changes to an approved trial protocol have to be submitted to EC and CA and positively appraised before implementation of the changes, if the changes are such that they may affect the subjects' safety, e.g.

  - o fundamental changes to the therapeutic procedures
  - o Updates to the SmPC/investigator brochure that may have an impact on the assessment of adverse events, including the definition of possible SUSARs.

- result in further data collection that requires changes to the patient information and/or informed consent form,
- affect the interpretation of the scientific documents upon which the trial is based or the significance of the results of the trial,
- significantly affect the leadership or conduct of the trial,
- concern the quality or the innocuousness of the investigational drug

Changes to the protocol may only be performed by the sponsor, the authorised representative or the coordinating investigator in cooperation with the biometrician.

After approval of the trial changes, all participating trial sites have to be informed about the changes in writing and supplied with potentially changed documents.

The whole process has to be documented in the Trial Master File (TMF).

12 DOCUMENTATION

12.1 General information and Access Rights

In the context of a database for electronic data capture only, the Case Report Form (CRF) will be designed by the ZKS Leipzig in cooperation with the Sponsor/sponsor's authorised representative/Coordinating Investigator and provided as electronic form (eCRF). In order to facilitate the documentation as per protocol in case of malfunction of the electronic system or any of its components, a paper version of the CRF (interim CRF) will be provided in the ISF (investigator site file). The data on this paper version will be transferred to the eCRF as soon as the electronic system is available again.

Special CRF forms will be provided as paper CRF, for example:

- **SAE-forms**, because these have to be sent to the ZKS Leipzig in a printable version for submission to the relevant authorities

An eCRF will be provided for each patient. The patient will be identified as per patient-ID only. The eCRF must be completed shortly after each trial visit according to ICH Guideline E6 chapter 11.1 and to enable central monitoring of the trial data.

Access to the data base will be limited to authorised staff only. Authorisation is granted by the site's investigator using the trial specific staff signature and delegation log. Based on the staff signature and delegation log access to the eCRF will be granted by the responsible staff at the ZKS Leipzig.

Authorised staff members on site will be able to enter and update data as well as finalise data by electronic signature during the conduct of the trial according to a trial specific concept for documentation. This concept is based on the internal Standard Operating Procedures implemented by the ZKS Leipzig and follows the ICH Guideline E6. All entries and data changes will be tracked automatically including date, time and person who entered/changed information (audit trail). Major correction(s) or major missing data have to be explained.

12.2 Patient File and Source Data

All information required by the protocol and therefor collected during the clinical trial must be recorded by the Investigator or an authorised member of the trial team as source data in the source documentation for the trial (e.g. patient file).

Source data according to ICH Guideline E6 are defined as any information in original records and/or certified copies of original records of clinical findings, observations, or other activities in a clinical trial necessary for the reconstruction and evaluation of the trial. Source data are contained in source documents.

The Source Data Agreement is defining source data and their location for respective CRF entries. It will be filled in at the initiation visit, signed by the investigator or deputy investigator and filed in the trial master file.

In order to confirm the completeness, accuracy and consistency of the data with the data in the source documents the investigator or deputy investigator has to electronically sign each patient's CRF after his/her individual end of trial participation.

12.3 Data Management

For creation of the trial database the EDC tool secuTrial®, developed and distributed by interActive Systems GmbH (iAS), will be used. The database will be validated according to the Standard Operating Procedures (SOPs) of the ZKS Leipzig prior to data capture. CDASH format standards will be applied.

The information entered into the eCRF by the investigator or an authorised member of the trial team is systematically checked for completeness, consistency and plausibility by routines implemented in CDMS such that discrepancies can be dealt with at data entry. Errors and Warnings are listed in a validation report and can be resolved at any time during entry process. On completion of the data entry the site staff flags the eCRF-pages as 'data entry completed' (DEC).

During on-site monitoring or central/statistical monitoring, the monitor or the data manager at the ZKS Leipzig may create a manual query for discrepancies that are identified after DEC. All eCRF-pages with queries are marked in the system and a report with all queries listed is available. The site staff is responsible for data correction and can resolve queries directly in the eCRF-page.

The ZKS Leipzig will supervise and support the solution of queries and will close all correctly resolved queries. In case a query cannot be solved, the data management staff may close the query in agreement with the trial biometrician. In case of self evident corrections by datamanagement staff, a list of self evident corrections or data entry conventions (e.g. for data entry of paper-based life questionaires) will be available and authorised by the coordinating investigator or the responsible biometrician of the clinical trial. Each trial site will receive a list of performed SECs at the end of the trial.

During the whole course of the trial, a backup of the data is made on a daily basis according to the backup policies of the ZKS Leipzig. Unauthorised access to patient data is prevented by the access concept of the trial database, which is based on a strict hirarchy and role concept. Any change of data (e.g. when data is changed in the database during query management) is recorded automatically via audit trail within the database. At the end of the trial, once the database has been declared complete and accurate, the database will be locked. Thereafter, any changes to the database are possible only by joint written agreement between sponsor/sponsors authorised representative/coordinating investigator, biometrician and data manager.

12.4 Archiving

All relevant trial documentation (Trial Master File), the electronically stored data, the original CRFs and the final report will be stored for at least 10 years by the sponsor after the regular or premature end of the trial.

At the investigating sites, the investigators' files, patient identification lists, signed written consent forms, copies of all CRFs and the patients' files will be stored for at least 10 years after the regular or premature end of the trial. Local rules or other legal requirements (e.g. Strahlenschutzgesetz) are to be met if they require longer periods of archiving.

If the clinical trial is being conducted in order to gain approval for a trial drug, then the requirements of the Arzneimittelprüfrichtlinie will be adhered to, which state that the patient identification lists are to be stored for up to 10 years after the trial's completion/termination.

13 REFERENCE EVALUATION

At screening, bone marrow analyses and CBC with differential will be performed by a central laboratory in Düsseldorf (cytomorphology). For this purpose 5 unstained bone marrow slides and 2 peripheral blood slides are to be sent to the central lab in Düsseldorf. Central morphology results will be used to verify inclusion criteria 1 and 2.

Timepoints for central cytomorphology samples:

5 native unstained bone marrow slides	• Screening<br>• Primary endpoint assessment<br>• EoT
2 native peripheral blood slides	• Screening<br>• Primary endpoint assessment<br>• EoT

Further details are specified in separate working instructions.

14 SUPERVISION OF THE CLINICAL TRIAL

14.1 Access to Source Data

According to ICH Guideline E6 and the applicable German laws, the investigator must permit all authorised third parties access to the trial site and the medical records of the trial subjects (source data). These include the clinical trial monitors, auditors and other authorised employees of the sponsor, as well as members of the local or federal authorities. All these persons are sworn to secrecy.

14.2 Monitoring

The ZKS Leipzig will be responsible for trial monitoring. Initiation, regular and close-out visits will be performed in all trial sites. A risk-based monitoring strategy will be implemented, as required by ICH E6 R2 (Chapter 5.0).

During trial conduct, central and statistical monitoring procedures will be combined with on-site monitoring visits in order to achieve high protocol compliance and data quality, as well as to ensure patients' safety and rights. The choosen monitoring strategy depends on the results of the risk analysis done during the protocol development and will described in the trial specific monitoring plan.

In general, a first monitoring visit at a trial site will be scheduled after the inclusion of the site's first patient, checking protocol compliance and preventing further systematic errors due to misunderstandings. All trial sites will then be visited regularly. The frequency of further on-site monitoring visits will depend on the trial site's recruitment rate and on whether problems have been detected with the site, either by prior on-site visits or by central monitoring.

Prior to every scheduled on-site visit, the monitor will be provided patient synopses summarising the data already available in the database, and indicating possible protocol deviations or inconsistencies. If deemed necessary (e. g. in case of a high number of data inconsistencies/queries), queries rised by pharmacovigilance and/or from the statistical monitoring will be communicated to the site in due time before the on site visit, to enable a timely processing.

During the visits the monitor will (according to the trial specific monitoring plan):

- check informed consent forms of all patients enrolled
- perform source data verification of the key data (selected baseline parameters, therapy delivery, serious adverse events, follow-up) in a random sample of the site's patients
- perform targeted source data verification for patients with possible deviations
- discuss open queries raised by data management or drug safety personnel
- check essential parts of the investigator site file
- check source data for AEs or SAEs, which have not been properly reported in the eCRF
- check for major GCP-breaches and/or protocol violations

.

14.3 Audits

In order to guarantee that the conduct of the trial is in accordance with ICH-GCP and the national laws audits are planned of the trial sites to be carried out by an unbiased auditor.
The investigator agrees to provide access to all relevant documents for review by the auditor.

14.4 Inspections

According to German Medicinal Products Act (AMG) and the corresponding GCP-guidelines (GCP-V), inspections of the trial sites may be performed by the local or federal authorities at any time during or after completion of the trial.

The investigator agrees to provide access to all relevant documents for review by the inspectors.

14.5 Data protection and Confidentiality

The sponsor, together with ZKS Leipzig and the trial sites, is responsible for the implementation and data processing in accordance with Article 4(7) of the EU Data Protection Basic Regulation 2016/679 in this trial. The ZKS Leipzig is responsible for implementation of procedures for data collection, storage, protection, retention and destruction. The ZKS Leipzig has implemented a data safety and security concept according to the requirements of the German Federal Office for Information Security (www.bsi.bund.de).

All data will be initially collected by investigators in the recruiting trial sites. Together with information on the trial, eligible patients will be informed about data capture, transmission, analysis processes and their rights according to the General Data Protection Regulation (GDPR). Once a patient is eligible and has given his/her informed consent to trial participation and data collection, the investigator will assign the patient a unique patient identification code. Patient identification code lists will be generated in advance by ZKS Leipzig and forwarded to the recruiting sites. These lists are part of the investigator site file and remain at the recruiting site. These lists are the only documents that allow for re-identification of the patients.

All clinical data entered by the investigators (or their designated staff) into eCRFs will be recorded in a pseudonymized form (i.e. without reference to the patient’s name and date of birth) exclusively using the patient’s identification code.

Clinical monitors appointed by ZKS Leipzig will regularly visit the recruiting sites and verify the informed consent forms. Data will only be used for analysis after it has been verified by monitors that the patient has unambiguously given his or her consent for trial participation as well as for data capture, transmission and analysis. The patients are informed of this fact and agree to the procedure with the patient information/informed consent.

In the event of withdrawal of consent, the necessity for storing data will be evaluated. While the General Data Protection Regulation (EU) 2016/679 strengthens personal data protection rights, encompassing the right to access, rectification and withdrawal of data, it also specifies the situations when restriction on those rights may be imposed. The withdrawal of informed consent should not affect the results of activities already carried out, such as the storage and use of data obtained on the basis of informed consent before.

14.6 Declaration regarding Data Protection

We hereby confirm that all clinical trial information will be recorded, processed, handled and stored by ZKS Leipzig, Härtelstr. 16-18, 04107 Leipzig, Germany on behalf of the sponsor.

Data captured by the investigators will be processed in such a way that it can be accurately reported, interpreted and verified while the confidentiality of records and the personal data of the subjects remain protected. Data capture and processing will be in accordance with the applicable law on personal data protection and with the "General Data Protection Regulation" (EC) 2016/679 of the European parliament and of the council.

Access to the data is strictly limited to authorised persons. Data are protected against unauthorised access.

14.7 Declaration regarding the Pseudonymized Transfer of Personal Data

The sponsor certifies herewith that the transfer of pseudonymized personal data will take place according to the documentation and communication regulations in §§ 12 und 13 of the GCP-guidelines (GCP-V)[40]. Moreover, the sponsor certifies that trial participants who do not permit the transfer of data will not be admitted to the trial. This will be ascterained by including the relevant information in the patient information/informed consent.

14.8 Anonymisation of Data after the end of Archiving

After the end of the archiving period, all clinical data present at the ZKS Leipzig will be stored in an anonymous form.

All data will be subject to an anonymization process removing personalised data as far as possible, i.e. without endangering the possibility to answer scientific questions related to the trial. Anonymised data will be relocated to a separate, access restricted, file location and secondary data sources will be deleted.

Non clinical data (like contact information) will be deleted after the end of the archiving period.

15 ADMINISTRATIVE AGREEMENTS

15.1 Adherence to the Protocol

The study will be conducted in accordance with the protocol, GCP and applicable regulatory requirements.

The clinical trial described here will be conducted and analysed in accordance with local laws (AMG/GCP-Verordnung) and ICH Guideline E6 (Good Clinical Practice - GCP).

Protocol violations are all deviations from the procedures outlined in this document, e.g.

- examinations that are missed or that take place at the wrong time
- non-compliance
- intake of prohibited medication

After a patient has been enrolled, it is the investigator's responsibility to avoid protocol violations in order to obtain unbiased data for the trial.

Those protocol violations deemed to be major are defined by the risk analysis performed before and during trial implementation and will be further detailed in separate documents belonging to the risk assessment/monitoring plan. This list can be augmented in the course of the trial. Major protocol violations will be reported to the ZKS Leipzig, which will inform the sponsor.

All protocol violations will be documented and discussed with the responsible biometrician before closing the data base and carrying out the statistical analysis.

The investigator must ensure that the recorded data are documented as per protocol. Minor variations are inevitable in clinical routine, but must be documented together with a justification.

15.2 Funding and Insurance

The LUCAS trial is supported financially by Curis, Inc. Curis provides the CA-4948 free of charge for the participating trial sites.
The collection, administration, analysis and interpretation of data is the responsibility of the sponsor, as well as the preparation of the final report and the decision to publication.

The co-ordinating investigator is responsible for the provision of insurance or indemnity to cover potential harm done to the patients due to the trial participation according to the national requirements and the corresponding national laws. The insurance will be organized by HDI Global SE, Eisenbahnstrasse 1-3, 04315 Leipzig, Germany as requested by the co-ordinating investigator. The maximum benefit for the individual insured person (patient) is 500,000.00 € for all insured events of the trial the total sum is 50,000,000.00 €.

A copy of the insurance policy and general insurance conditions (AVB) can be obtained from the investigator site file. The patient is given a copy of the insurer's general terms and conditions.

15.3 Notification of Local Authorities

Prior to enrolment of the first patient in the trial, the sponsor, his/her legal representatives/ contractors and all investigators and their deputies are responsible according to German Medicinal Products Act AMG § 67 (1) and the requirements of the GCP-V §§ 12 and 13 for notifying the local regulatory authority of their participation in the trial.

According to § 67 (3) AMG and §§ 12,13 GCP-V the sponsor, his/her legal representatives/ contractors and all investigators and their deputies are also responsible for notifying the local regulatory authority of amendments, premature termination of trial arms or of the whole trial and the regular trial termination.

15.4 Publication Policy and Registration

The results of this trial will be submitted for publication in a peer-reviewed, international English-language journal of appropriate aim and scope. Accordingly, the clinical trial will be registered at clinicaltrials.gov before recruitment starts. According to the results of main and concomitant studies, the results may be submitted in separate or combined manuscripts; decisions about the form and scope of individual manuscripts will be discussed among all persons participating in the design, conduct and analysis of the trial who qualify for authorship. The coordinating investigator together with the biometrician(s) is responsible for drafting and

circulating manuscripts and for discussing and handling requests by co-authors or/and sponsors to edit the text.

The authorship will follow the criteria for authorship developed by the International Committee of Medical Journal Editors (ICMJE), including those that distinguish authors from other contributors.

The ICMJE recommends that authorship be based on the following 4 criteria:

- Substantial contributions to the conception or design of the work; or the acquisition, analysis, or interpretation of data for the work; AND
- Drafting the work or revising it critically for important intellectual content; AND
- Final approval of the version to be published; AND
- Agreement to be accountable for all aspects of the work in ensuring that questions related to the accuracy or integrity of any part of the work are appropriately investigated and resolved.

All those designated as authors should meet all four criteria for authorship, and all who meet the four criteria should be identified as authors. Those who do not meet all four criteria will be acknowledged in the manuscript.

The scientific use of data resulting from this trial by local trial sites is ruled by the site contracts between the sponsor and the local trial sites. Generally, sites might use data for own scientific questions (independent from the questions discussed in this trial protocol) and publication after consultation with the sponsor.

15.5 Data Sharing Statement

According to the recommendations on data sharing by the International Committee of Medical Journal Editors (ICMJE) data resulting from the LUCAS-trial will be made available to the scientific community as follows:

After publication of the major results and upon reasonable request from researchers performing an individual patient data meta-analysis, individual patient data that underlie published results will be shared after de-identification. This requires approval by the local Institutional Review Board (IRB) of the researcher requesting the data along with public registration of the meta-analysis.

Summary statistics that go beyond the scope of published material will be made available to researchers for meta-analysis upon reasonable request and if the necessary data analysis is not unduly time-consuming. Together with publication of the main results, the trial protocol in full will be made publically available as well as the statistical analysis plan.

16 PROTOCOL SIGNATURES

Confirmation of the Final Protocol

We hereby certify that this is the final version of the protocol:

Coordinating investigator:		
Prof. Dr. Uwe Platzbecker	Date	Signature
Biometrician:		
Dr. Dirk Hasenclever	Date	Signature

17 PROTOCOL AGREEMENT

Herewith I declare that I have read and understood the present protocol and agree to take into account and follow each part of it. I will ensure that all trial patients enrolled in this trial site will be treated, observed and documented in accordance with this protocol. I will ensure that all persons assisting with the trial under my supervision are adequately informed about the protocol, the investigational product and their duties.

I further declare that I do not have any financial and other competing interests in this trial.

Centre-ID	_____ - __/__/__/
Address trial site (stamp)	

Date

Signature *(Principal)* Investigator

Date

Signature Deputy Investigator *(**Germany only**, acc. to national law)*

18 APPENDIX

18.1 Modification of the revised IWG 2018 hematological response criteria

Implemening IWG 2018 Hb-response criteria in a clinical trial requires some modifications. Mostly, IWG 2018 rules are only specified further to make them technically operational, unambiguous, and to handle data problems expected in real world trial data.

The main deviation from IWG 2018 is the introduction of so-called sliding time windows over which Hb-measurements can be averaged not only for the determination of the baseline Hb-level, but also to determine Hb-response for NTD patients. This deviation makes the response definitions statistically robust and markedly increases the power to detect a true response (Hasenclever et al in preparation).

18.1.1 Baseline evaluation period

Patients enter the LUCAS trial in two steps:

- Registration for the study with informed consent, preliminary check of inclusion/exclusion criteria, and initiation of study-specific screening investigations during a screening period. (*CAVE: Patients should only be registered if valid baseline data is to be expected at the end of the screening period*)
- Formal inclusion with a final check of inclusion and exclusion criteria after completion of all study-specific screening investigations at the end of the baseline evaluation period and shortly before start of treatment.

The baseline evaluation period is defined relative to the day d0 of formal inclusion and overlaps with the screening period.

Baseline evaluation period consists of the days -112 to 0 (inclusive; last 16 weeks). In order to accommodate pragmatically for delays in formal inclusion after meeting the inclusion criteria for up to a week or in order to fulfill the validity criteria stated below an extension of the baseline evaluation period up to days -130 to 0 is tolerated.

Patients should be registered for the trial and start study-specific investigations only, if they fulfill the preliminary inclusion/exclusion criteria, which include that valid baseline data are expected to be available at the projected end of the baseline evaluation period.

Specification of minimum data required for the whole baseline evaluation period:

- A complete transfusion history during the whole baseline evaluation period must be documented before inclusion into the trial. For all transfusion episodes, this includes calendar date, the Hb-value preceding the decision to give a transfusion, and the number of RBCs given.
- *Ideally, there should be a uniform transfusion strategy applied during the whole baseline evaluation period.* The transfusion trigger threshold characterizing the transfusion strategy (e.g. < 8 g/dL) must be documented at registration and must be applied afterwards.
    - If transfusions were given during the baseline period, the Hb values that

triggered the transfusions must be consistent with the stated transfusion trigger threshold. Only then is the transfusion burden before and after start of treatment are comparable.

- A Hb-value just before a transfusion which lies outside the interval [transfusion trigger threshold ± 1.0 g/dl] generates a query and must be explained or justified clinically.

18.1.2 Calculation of baseline Hb level

Baseline Hb level will be calculated for NTD as well as transfusion dependent cases from all available and valid Hb measurements from the baseline evaluation. All Hb measurements must be documented in the trial database before inclusion into the trial.

All measurements taken less than 7 days after a transfusion are discarded from the Hb history as invalid for NTD patients. Operationally, if a transfusion was given on day d, Hb-values taken after the transfusion from day d up to day d + 6 inclusive are discarded. For transfusion dependent cases, all Hb-values triggering a transfusion are retained. In case there was a transfusion on the same day as a blood sampling, it must be documented whether the Hb measurement was before or after the transfusion. The (remaining) Hb history is valid if the mean is based on at least 5 Hb measurements that spans at least 100 days. In addition, the baseline Hb level is defined as the weighted mean over all valid Hb measurements in the Hb history. To eliminate the bias potentially introduced when Hb-measurements are less than 7 days apart, weights to each Hb value are assigned in a given time window and weighted mean for averaging of time windows are used (for details see the statistical analysis plan).

18.1.3 Calculation of the baseline transfusion count

The baseline RBC transfusion count (R) is the number of RBC-transfusions documented in the baseline evaluation period. Multiple RBCs can be given in a single transfusion episode.

Based on the RBC transfusion count, patients are classified as

- non transfusion dependent (NTD): $R \leq 2$ RBCs,
- low transfusion burden (LTB): $3 \leq R \leq 7$ RBCs, and
- high transfusion burden (HTB): $R \geq 8$ RBCs.

18.1.4 Sliding windows to calculate Hb means

Repeated Hb measurements in a steady state fluctuate naturally with a certain standard deviation (SD) about their true underlying (unknown) mean. Based on available Hb time course data, we estimate that SD may be in the order of SD = 0.4 to 0.5 g/dL when using the same measurement method. If different methods to measure Hb are used, SD may be even larger.

Simulations show that the IWG 2018 reponse criteria are statistically instable with such chance fluctuations. Therefore we use the mean over at least the four most recent Hb values in a sliding window of at least 8 weeks to define response.

We assign a **sliding window of at least eight weeks** to each Hb measurement taken more than 8 weeks after start of treatment. For a sliding window to be valid, we require that the sliding window contain at least 4 valid Hb measurements (in order to reduce the standard error of the mean to at least SD/2) and that the included Hb measurements span at least 50 days

(in order represent an 8 week time period as required by IWG 2018). If an eight week window contains less than 4 Hb measurements, it can be extended to maximally 12 weeks to fulfill this condition.

18.1.5 Response in NTD patients (Hb response)

HI-E response in NTD patients is defined algorithmically as follows:

Calculate Hb means for all valid Sliding Windows assigned to the time course of Hb measurements.

- A HI-E response in NTD patients is achieved as soon as the HbMean in a sliding window is ≥ 1.5 g/dL above the baseline Hb level without intermittent transfusions.
- The response starts on the day of the last Hb measurement to which the response defining sliding window was assigned.
- The response ends as soon as the HbMean in a sliding window is < 1.0 g/dL above the baseline Hb level or a transfusion is given after start of response.
- The response ends on the day of the last Hb measurement to which the sliding window was assigned that documents the end of the response or on the day of the first past response transfusion (whichever comes first).

18.1.6 Response in LTB and HTB patients (response in transfusions)

At registration, investigators have committed to a transfusion trigger threshold for the registered patient which should be applied from registration until the end of the study.

For LTB and HTB patients, the Hb time course must be documented too. We assume and require that there always be an Hb value before any transfusion episode.

Hb must be regularly measured such that compliance with the stated Hb trigger is documented. Response in transfusion RBC count is only meaningful if it is documented that the stated transfusion strategy was adhered to.

In order to determine response in LTB and HTB, we look at sliding 8-week windows assigned to Hb measurements as defined above for NTD.

An Hb response in LTB and HTB is documented if no transfusions were given in a sliding 8-week window provided that the Hb measurement to which the sliding window was assigned was consistent with the stated Hb trigger and was not followed by a transfusion in the next two days.

The condition on the Hb values prevents postponing a transfusion on purpose to achieve a formal response.

It is clinically implausible – in particular for HTB - to define the end of response (suggesting stop of treatment) by the first transfusion given after documented Hb-response.

In order to define the end of Hb-response, we adapt the concept of minor response mentioned in IWG 2018 for HTB only.

At baseline, we determine the baseline transfusion rate as number of RCBs divided by length of the baseline window. Similarly, there is a transfusion rate for each response window.

We define the transfusion rate ratio as the ratio of the transfusion rate in the response window divided by the baseline transfusion rate. We use transfusion rates instead of transfusion counts because the width of the time windows varies depending on availability of data.

The Hb-transfusion response ends on the last day of the first response window after a documented response that has response transfusion rate ratio > 0.5, i.e. inconsistent with at least a minor response.

18.2 Classification of Adverse Events

18.2.1 Degree of Seriousness

The seriousness of an Adverse Event will be determined in accordance with the definitions in 8.1 and 8.4.

18.2.2 Assessment of Intensity/Severity

The assessment of the intensity accords with CTCAE V5.0

Mild Adverse Event	• asymptomatic or mild symptoms;<br>• clinical or diagnostic observations only;<br>• intervention not indicated.
Moderate Adverse Event	• minimal, local or noninvasive intervention indicated;<br>• limiting age-appropriate instrumental ADL*[30].
Severe Adverse Event	• medically significant but not immediately life-threatening;<br>• hospitalisation or prolongation of hospitalisation indicated;<br>• disabling;<br>• limiting self care ADL**
Life-threatening Adverse Event	• Life-threatening consequences;<br>• urgent intervention indicated
Death related to Adverse Event	

[30] Activities of Daily Living (ADL)
*Instrumental ADL refer to preparing meals, shopping for groceries or clothes, using the telephone, managing money, etc.
**Self care ADL refer to bathing, dressing and undressing, feeding self, using the toilet, taking medications, and not bedridden.

18.2.3 Determining the Causal Relationship

The investigator/ the deputy or the authorised medical staff must assess whether or not the Adverse Event is causally related to the administration of the trial medication. The following classification is to be used.

- Reasonable possibility
- No reasonable possibility

A reasonable possibility exists, if one of the following WHO-UMC criteria is met:

- occurring in a plausible time relationship to drug administration, and which cannot be explained by concurrent disease or other drugs or chemicals. The response to withdrawal of the drug (dechallenge) should be clinically plausible. The event must be definitive pharmacologically or phenomenologically, using a satisfactory rechallenge procedure if necessary.
- with a reasonable time sequence to administration of the drug, unlikely to be attributed to concurrent disease or other drugs or chemicals, and which follows a clinically reasonable response on withdrawal (dechallenge). Rechallenge information is not required to fulfil this definition.
- with a reasonable time sequence to administration of the drug, but which could also be explained by concurrent disease or other drugs or chemicals. Information on drug withdrawal may be lacking or unclear.
- more data is essential for a proper assessment or the additional data are under examination
- cannot be judged because information is insufficient or contradictory, and which cannot be supplemented or verified

No reasonable possibility exists, if the following WHO-UMC criterion is met:

- with a temporal relationship to drug administration which makes a causal relationship improbable, and in which other drugs, chemicals or underlying disease provide plausible explanations.

18.2.4 Expected/Unexpected

Adverse Events are unexpected if they do not occur in the manner or with the intensity described in the Investigator's Brochure (see investigator's files).

18.2.5 Outcome of an Adverse Event

The outcome of an Adverse Event is classified as follows:

- recovered/resolved
- recovering/resolving
- not recovered/not resolved
- recovered/resolved with sequel
- fatal*
- unknown

*Note: A patient’s death is not in itself an event, but the consequence of one. The event that led to the patient’s death must be documented completely and reported even if death occurs four weeks after stopping medication and independent of whether or not there is a relation to the therapy or not.

18.3 Acronyms

ABC	Activated B-cell-like
aCML	Atypical Chronic Myeloid Leukemia
AE	Adverse event
ALC	Absolute lymphocyte count
ALP	Alkaline phosphatase
ALT	Alanine aminotransferase
AMG	Arzneimittelgesetz (= German Medicinal Products Act)
AML	Acute myeloid leukemia
ANC	Absolute neutrophil count
APL	Acute promyelocytic leukemia
ASR	Annual Safety Report
AST	Aspartate aminotransferase
AUC	Area under the plasma concentration versus time curve
AUC(0-24)	Area under the plasma concentration-time curve from 0 to 24 hours
AUC(INF)	Area under the plasma concentration-time curve from 0 to infinity
BCL6	B-cell lymphoma 6
BfArM	Bundesinstitut für Arzneimittel und Medizinprodukte (German Competent Authority)
BID	Twice daily (*Latin "bis in die"*)
BMMNC	Bone marrow mononuclear cells
CA	Competent Authority
CFR	Code of Federal Regulations
CK	Creatine kinase
Cmax	Maximum plasma concentration
Cmin	Trough plasma concentration
CML	Chronic myeloid leukemia
CMML	Chronic myelomonocytic leukemia
CNB	Core needle biopsy
CNS	Central nervous system
CR	Complete response
CRR	Complete remission rate
CSF	Cerebrospinal fluid
CT	Computed tomography
CTCAE v4.03	Common Terminology Criteria for Adverse Events Version 4.03

cTnI	Cardiac Troponin I
CYP450	Cytochrome P450
DCR	Disease control rate
DLBCL	Diffuse large B-cell lymphoma
DLT	Dose-limiting toxicity
DMSO	Dimethyl sulfoxide
DOR	Duration of response
ECG	Electrocardiogram
ECOG	Eastern Cooperative Oncology Group
eCRF	Electronic Case Report Form
EFS	Event free survival
ERK	Extracellular-signal regulated kinase
FDA	Food and Drug Administration
FDG-PET	Fluorodeoxyglucose-PET
FLT	Fluorothymidine
FLT3	FMS-like tyrosine kinase 3
FNA	Fine needle aspiration
FT4	Free T4
G6PD	Glucose-6-phosphpate dehydrogenase
GCB	Germinal center B-cell-like
GCP	Good Clinical Practice
GCP-V	GCP-Verordnung
GLP	Good Laboratory Practices
GVHD	Graft versus host disease
HbcAb	Hepatitis B core antibody
HBV	Hepatitis B virus
HCV	Hepatitis C virus
hERG	Human ether-a-go-go related gene
HI	Hematologic improvement
HI-E	Hematologic improvement–erythroid
HIPAA	Health Insurance Portability and Accountability Act
HIV	Human immunodeficiency virus
HNSTD	Highest non-severely toxic dose
HPLC	High performance liquid chromatography
HSCT	Hematopoietic stem cell transplant
HTB	High transfusion burden

IB	Investigator's Brochure
IC50	Half maximal inhibitory concentration
ICF	Informed consent form
ICH	International Council for Harmonisation
ICSH	International Council for Standardization in Hematology
IEC	Independent Ethics Committee
IFN-β	Interferon-beta
IgA	Immunoglobulin A
IgG	Immunoglobulin G
IgM	Immunoglobulin M
IKK	I KappaB kinase
IL-10	Interleukin-10
IL1R	Interleukin-1 receptor
IL-6	Interleukin-6
IL-8	Interleukin-8
IMP	Investigational medicinal product
INR	International normalized ratio
IPI	International Prognostic Index
IRAK4	Interleukin-1 receptor-associated kinase 4
IRB	Institutional Review Board
ISSWM	International Prognostic Scoring System for Waldenström macroglobulinemia
ITD	Internal tandem duplications
IV	Intravenous
IWG	International working group
JAK	Janus kinase
Kd	Binding constant
KD	Kinase domain
Kg	Kilogram
LDH	Lactate dehydrogenase
LKP	Leiter der klinischen Prüfung
LTB	Low transfusion burden
MABEL	Minimum anticipated biological effect level
MALT	Mucosa-associated lymphoid tissue
MCL	Mantle Cell Lymphoma
MDS	Myelodysplastic syndrome
MDSC	Myeloid-derived suppressor cell

MDS/MPN	myelodysplastic/myeloproliferative neoplasias
MDS/MPN-RS-T	MDS/MPN with ring sideroblasts and thrombocytosis
MDS/MPNu	MDS/MPN unclassifiable
MedDRA	Medical Dictionary for Regulatory Activities
Mg	Milligram
MRI	Magnetic resonance imaging
MTD	Maximum tolerated dose
MYD88	Myeloid differentiation primary response 88
NCI	National Cancer Institute
NF-κB	Nuclear factor kappa light chain enhancer of activated B-cells
nM	Nanomolar
NOAEL	No observed adverse effect level
NOS	Not otherwise specified
NTD	Non transfusion dependent
NYHA	New York Heart Association
ORR	Overall response rate
OS	Overall survival
PBMC	Peripheral blood mononuclear cells
PD	Progressive disease
PDX	Patient-derived xenograft
PEI	Paul-Ehrlich-Institut (Agency of the German Federal Ministry of Health. Its research and control activities promote the quality, efficacy and safety of biological medicinal products.)
PET	Positron emission tomography
PFS	Progression free survival
P-gp	P-glycoprotein
PIA	Plasma inhibitory assay
PK	Pharmacokinetics
PR	Partial response
PT	Prothrombin time
PTT	Partial thromboplastin time
QD	Quaque die (once daily)
QTc	Q-T interval, corrected
QTcF	Q-T interval with Fridericia’s correction
RAEB	Refractory anemia with excess blasts
RFS	Relapse free survival
RP2D	Recommended Phase 2 dose

RR	Relapsed/Refractory
SAE	Serious Adverse Event
SAP	Statistical analysis plan
SAR	Serious Adverse Reaction
SD	Stable disease
SEAP	Secreted embyonic alkaline phosphatase
SOC	System organ class
SPD	Sum of the product of the diameters
STAT3	Signal transducer and activator of transcription 3
STD	Severely toxic dose
STD10	Severely toxic dose in 10% of animals
SUSAR	Suspected Unexpected Serious Adverse Reaction
T1/2	Plasma terminal elimination half-life
TBD	To be determined
TEAE	Treatment-emergent adverse event
TGI	Tumor growth inhibition
TIR	Toll/IL-1 receptor
TLR	Toll-like receptor
TLS	Tumor lysis syndrome
Tmax	Time to maximum plasma concentration
TNF	Tumor necrosis factor
TRAE	Treatment related adverse event
TRAF6	TNF receptor associated factor 6
TSH	Thyroid stimulating hormone
ULN	Upper limit of normal
US	United States
Vdss	Volume of distribution at steady state
VGPR	Very good partial response
WHO	World Health Organization
WHO-UMC	World Health Organization – Uppsala Monitoring Centre
WM/LPL	Waldenström macroglobulinemia/ lymphoplasmacytic lymphoma
ZKS Leipzig	Zentrum für klinische Studien Leipzig
µM	Micromolar

18.4 Labelling

The German Label will include the following information:

Nur zur klinischen Prüfung bestimmt!

Protokoll LUCAS (EudraCT 2020-003986-20)

CA-4948 Tabletten, 50 mg

25 Tabletten zum Einnehmen

Ch.-B.: <LOT-Nr.> verwendbar bis: <MON/JJJJ>

Patienten-ID: _______-___

Außerhalb der Reichweite von Kindern aufbewahren!

Dosierungsanweisung: Einnahme gemäß Studienprotokoll!

Bei Raumtemperatur (15-25 °C) lagern.

Sponsor: Universität Leipzig - Medizinische Fakultät, Liebigstraße 22, 04103 Leipzig, Germany, Prof. Dr. Uwe Platzbecker, Medizinische Klinik und Poliklinik I – Hämatologie und Zelltherapie, Internistische Onkologie, Hämostaseologie, Telefon: +49 (0)341 97 13050

18.5 Template trial protocol

This trial protocol was written based on a template by the ZKS Leipzig based on the SOPs of the ZKS Leipzig.

The used template version is: Final 9.0 from 20-JAN-2020

18.6 Supplemental Materials

These documents are relevant to the protocol, but they are not considered part of the protocol. They are stored and modified separately. As such, modifications to these documents do not require protocol amendments.

18.6.1 ECOG PERFORMANCE STATUS SCALE

ECOG Performance Status	
**Grade**	**Description**
0	Fully active, able to carry on all pre-disease performance without restriction
1	Restricted in physically strenuous activity but ambulatory and able to carry out work of a light or sedentary nature, e.g., light house work, office work
2	Ambulatory and capable of all self-care but unable to carry out any work activities. Up and about more than 50% of waking hours

ECOG Performance Status	
3	Capable of only limited self-care, confined to bed or chair more than 50% of waking hours
4	Completely disabled. Cannot carry on any self-care. Totally confined to bed or chair
5	Dead

Source: Oken_1982

18.6.2 2018 INTERNATIONAL WORKING GROUP RESPONSE CRITERIA FOR MYELODYSPLASTIC SYNDROMES

The following response criteria are based on the International MDS Working Group (IWG) Criteria 2018 by *Platzbecker et al.*

Complete remission (CR)

Bone marrow evaluation: Bone marrow showing < 5% myeloblasts with normal maturation of all cell lines*. Persistent dysplasia will be noted*.

Peripheral blood‡ evaluation:

- Hemoglobin > 11 g/dL (untransfused, patient not on erythropoietin)
- Neutrophils ≥ 1.000/mm^3 (not on a myeloid growth factor)
- Platelets ≥ 100.000/mm^3 (untransfused, not on a thrombopoetic agent)
- Blasts – 0%

Partial remission (PR)

All of the CR criteria (if abnormal prior to treatment), except:

Bone marrow evaluation: Blasts decreased by ≥ 50% over pretreatment but still > 5%. Cellularity and morphology are not relevant.

Marrow CR

- Bone marrow: ≤ 5% myeloblasts and decrease by ≥ 50% over pretreatment
- Peripheral blood: if HI responses, they will be noted in addition to marrow CR

Stable disease

Failure to achieve at least a PR, but with no evidence of progression for > 8 weeks.

Failure

Death during treatment or disease progression characterized by worsening of cytopenias, increase in percentage of bone marrow blasts, or progression to a more advanced MDS FAB subtype than pretreatment.

Relapse (following a CR or PR)

One or more of the following:

- Return to pretreatment bone marrow blast percentage.
- Decrement of ≥ 50% from maximum remission response levels in granulocytes or platelets.
- Reduction in Hb concentration by ≥ 1.5 g/dL or transfusion dependence.

Disease progression

For patients with:

- Less than 5% blasts: ≥ 50% increase in blasts to > 5% blasts
- 5%-10% blasts: ≥ 50% increase to > 10% blasts
- 10%-20% blasts: ≥ 50% increase to > 20% blasts
- 20%-30% blasts: ≥ 50% increase to > 30% blasts

Any of the following:

- At least 50% decrement from maximum remission/response in granulocytes or platelets
- Reduction in Hb by ≥ 2 g/dL
- Transfusion dependence

Cytogenetic response

Requires 20 analyzable metaphases when using conventional cytogenetic techniques.

- Complete Cytogenetic Response: Disappearance of the chromosomal abnormality without appearance of new ones.
- Partial Cytogenetic Response: At least 50% reduction of the chromosomal abnormality.

Survival

Endpoints:

Overall: death from any cause
Event free: failure or death from any cause
PFS: disease progression or death from MDS
DFS: time to relapse
Cause-specific death: death related to MDS

To convert hemoglobin from grams per deciliter to grams per liter, multiply grams per deciliter by 10.
MDS indicates myelodysplastic syndromes; Hb, hemoglobin; CR, complete remission; HI, hematologic improvement; PR, partial remission; FAB, French-American-British; AML, acute myeloid leukemia; PFS, progression-free survival; DFS, disease-free survival.
*Dysplastic changes should consider the normal range of dysplastic changes.
‡In some circumstances, protocol therapy may require the initiation of further treatment (eg, consolidation, maintenance) before the 4-week period. Such patients can be included in the response category into which they fit at the time the therapy is started. Transient cytopenias during repeated chemotherapy courses should not be considered as interrupting durability of response, as long as they recover to the improved counts of the previous course.

Hematologic improvement criteria for MDS:

Hematologic Improvement (HI)	Response Criteria
Erythroid response (HI-E) (pretreatment Hb < 10 g/dL)	
**NTD** patients at baseline (<3 RBCs† in 16 weeks)	Absence of any transfusion and at least 2 consecutive Hb measurements ≥1.5 g/dL for a period of minimum 8 weeks in an observation period of 16-24* weeks compared with the lowest mean of 2 Hb measurements (≥7 days apart from any RBC transfusion event) within 16 weeks before treatment onset.
**LTB** patients at baseline (3-7 RBCs† in 16 weeks in at least 2 transfusion episodes)	HI-E in LTB patients corresponds to transfusion independence, defined by the absence of any transfusions for at least 8 weeks in an observation period of 16-24* weeks with the same transfusion policy compared with 16 weeks prior to treatment
**HTB** patients at baseline (≥8 RBCs† in 16 weeks)	<u>Major response</u>: Major HI-E response in HTB patients corresponds to transfusion independence, defined by the absence of any transfusions over a period of minimum 8 weeks in an observation

Hematologic Improvement (HI)	Response Criteria
	period of 16-24* weeks with the same transfusion policy† compared with 16 weeks prior to treatment<br><br>Minor response: Minor HI-E response in HTB patients is defined as a reduction by at least 50% of RBCs over a minimum of 16 weeks with the same transfusion policy† compared with 16 weeks prior to treatment
Platelet response (HI-P) (pretreatment < 100 x 10^9/L)	• Absolute increase of 30 x 10^9/L for patients starting with >20 x 10^9/L PLTs or<br>• Increase from <20 x 10^9/L to >20 x 10^9/L and by at least 100%.<br>In addition:<br>• Evolution of bleeding symptoms to be taken into account.<br>• Increments of platelets for patients with a pretreatment PLT count of >100 x 10^9/L are to be reported.
Neutrophil response (HI-N) (pretreatment < 1.0 x 10^9/L)	At least 100% increase and an absolute increase of >0.5 x 10^9/L. Increments of neutrophils for patients with a pretreatment ANC of >1.0 x 10^9/L are to be reported.
Progression or relapse after HI‡	**HI-E:**<br>*NTD patients with previous reponse according to IWG 2018 criteria*:<br>• Reduction in Hb by ≥ 1.5 g/dL or<br>• New transfusion dependence (i.e. ≥3 RBCs within a period of 16 weeks)<br>*LTB patients with previous reponse according to IWG 2018 criteria*:<br>• Reduction in Hb by ≥ 1.5 g/dL or<br>• New Transfusion dependence (i.e. ≥3 RBCs within a period of 16 weeks)<br>*HTB patients with previous reponse according to IWG 2018 criteria*::<br>• Increase in transfusion burden by at least 50%<br><br>**HI-N**:<br>• At least 50% decrement from maximum response levels in neutrophils and<br>• IWG 2018 response criteria are not met any more plus absence of dose interruptions<br><br>**HI-P**:<br>• At least 50% decrement from maximum response levels in platelets and<br>• IWG 2018 response criteria are not met any more plus absence of dose interruptions

To convert hemoglobin levels from grams per deciliter to grams per liter, multiply grams per deciliter by 10.
Hb indicates hemoglobin; RBC: red blood cell; HI: hematologic improvement.
*Observation period is per definition (according to IWG 2018) 16-24 weeks. In the LUCAS trial the primary endpoint is measured after 16 weeks of treatment so the observation period is of 16 weeks here.
†Only RBC transfusions administered for an Hb level below 9 g/dL are taken into account

Duration of response

Defined as time to disease progression (as per bone marrow response) or progression/relapse following hematologic improvement (as per hematologic improvement)

Source: (*Cheson et al. 2006*; *Platzbecker 2018*)

*Dysplastic changes should consider the normal range of dysplastic changes (modification).41

†Modification to IWG response criteria.

‡In some circumstances, protocol therapy may require the initiation of further treatment (eg, consolidation, maintenance) before the 4-week period. Such patients can be included in the response category into which they fit at the time the therapy is started. Transient cytopenias during repeated chemotherapy courses should not be considered as interrupting durability of response, as long as they recover to the improved counts of the previous course.

18.6.3 TUMOR LYSIS SYNDROME IDENTIFICATION AND MANAGEMENT RECOMMENDATIONS

Recommendations for Initial Management of Electrolyte Abnormalities and Prevention of Tumor Lysis Syndrome (TLS).

Section 1: First Dose of CA-4948 (within 24 hours of first dose)

- Within the first 24 hours after the first dose, if any laboratory criteria below are met, the patient should be hospitalized for monitoring and the investigator notified. No additional CA-4948 doses should be administered until resolution. A rapidly rising serum potassium is a medical emergency.
- Nephrology (or other acute dialysis service) should be consulted/contacted (per institutional standards to ensure emergency dialysis is available) on admission for any subject hospitalized prophylactically or in response to laboratory changes.
- IV fluids (e.g., D5 1/2 normal saline) should be initiated at a rate of at least 1 mL/kg/hr rounded to the nearest 10 mL (target 150 to 200 mL/hr; not < 50 mL/hr). Modification of fluid rate should also be considered for individuals with specific medical needs.
- Rasburicase must be administered/considered per regional standards or institutional guidelines for subjects with rapidly rising uric acid level.
- Monitor for symptoms or signs of TLS (e.g., fever, chills, tachycardia, nausea, vomiting, diarrhea, diaphoresis, hypotension, muscle aches, weakness, paresthesias, mental status changes, confusion, and seizures). If any clinical features are observed, recheck potassium, inorganic phosphate, uric acid, calcium and creatinine within 1 hour.
- Vital signs should be taken at time of all blood draws or any intervention.
- The management recommendations below focus on the minimum initial responses required. If a diagnosis of TLS is established, ongoing intensive monitoring and multi-disciplinary management will be per institutional protocols.

In addition to the recommendations in the table below, for patients receiving the first dose of CA-4948:

- For potassium increase ≥ 0.5 mmol/L from baseline, or any value > 5.0 mmol/L, recheck potassium, inorganic phosphates, uric acid, calcium, and creatinine within 1 hour and follow first guideline.
- For inorganic phosphate increase of > 0.5 mg/dL AND > 4.5 mg/dL, administer phosphate binder and recheck potassium, inorganic phosphate, uric acid, calcium, and creatinine within 1 hour.

Abnormality	Management Recommendations
**Hyperkalemia (including rapidly rising potassium)**	
Potassium ≥0.5 mmol/L increase from prior value (even if potassium within normal limits [WNL])	• Recheck potassium, phosphorus, uric acid, calcium and creatinine in 1 hour. If further ≥ 0.2 mmol/L increase in potassium, but still < upper limit of normal (ULN), manage as per potassium ≥ ULN. Otherwise recheck in 1 hour.<br>• Resume per protocol testing if change in potassium is < 0.2 mmol/L, and potassium < ULN, and no other evidence of tumor lysis.<br>• At discretion of investigator, may recheck prior to hospitalization.<br>• If stable or decreased, and still WNL, hospitalization is at the discretion of the investigator. Potassium, phosphorus, uric acid, calcium, and creatinine must be rechecked within 24 hours.
Potassium >ULN	• Perform STAT ECG and commence telemetry.<br>• Nephrology (or other acute dialysis service) notification with consideration of initiating dialysis.<br>• Administer Kayexalate 60 g (or Resonium A 60 g).<br>• Administer furosemide 20 mg IV × 1.<br>• Administer calcium gluconate 100 to 200 mg/kg IV slowly if there is ECG/telemetry evidence of life-threatening arrhythmias.<br>• Recheck potassium, phosphorus, uric acid, calcium, and creatinine in 1 hour STAT.<br>• If potassium < ULN 1 hour later, repeat potassium, phosphorus, uric acid, calcium, and creatin
Potassium ≥6.0 mmol/L (6.0 mEq/L) and/or symptomatic (e.g., muscle cramps, weakness, paresthesias, nausea, vomiting, diarrhea)	• Perform STAT ECG and commence telemetry.<br>• Nephrology (or other acute dialysis service) assessment with consideration of initiating dialysis.<br>• Administer Kayexalate 60 g (or Resonium A 60 g).<br>• Administer furosemide 20 mg IV × 1.<br>• Administer insulin 0.1 U/kg IV + D25 2 mL/kg IV.<br>• Administer sodium bicarbonate 1 to 2 mEq IV push.<br>*NOTE: If sodium bicarbonate is used, rasburicase should not be used as this may exacerbate calcium phosphate precipitation.*

	• Administer calcium gluconate 100 to 200 mg/kg IV slowly if there is ECG/telemetry evidence of life-threatening arrhythmias. Do not administer in same IV line as sodium bicarbonate.<br>• Recheck potassium, phosphorus, uric acid, calcium, and creatinine every hour STAT.
**Hyperuricemia**	
Uric acid ≥8.0 mg/dL (476 µmol/L)	• Consider rasburicase (0.2 mg/kg as an intravenous infusion over 30 minutes, or a dose following institutional guidelines).<br>*NOTE: If rasburicase is used, sodium bicarbonate should not be used as this may exacerbate calcium phosphate precipitation.*<br>• Recheck potassium, phosphorus, uric acid, calcium, and creatinine in 1 hr STAT.
Uric acid ≥10 mg/Dl (595 µmol/L)<br>OR<br>Uric acid ≥8.0 mg/dL (476 µmol/L) with 25% increase and creatinine increase ≥0.3 mg/dL (≥0.027 mmol/L) from pre-dose level	• Administer rasburicase (0.2 mg/kg as an intravenous infusion over 30 minutes, or a dose following institutional guidelines).<br>*NOTE: When rasburicase is used, sodium bicarbonate should not be used as this may exacerbate calcium phosphate precipitation.*<br>• Notify nephrology (or other acute dialysis service).<br>• Recheck potassium, phosphorus, uric acid, calcium and creatinine in 1 hour STAT.<br>• If uric acid < 8.0 mg/dL 1 hour later, repeat potassium, phosphorus, uric acid, calcium, and creatinine 2 and 4 hours later, if no other evidence of tumor lysis.
**Hypocalcemia**	
Calcium ≤7.0 mg/dL (1.75 mmol/L)<br>AND<br>Patient symptomatic (e.g., muscle cramps, hypotension, tetany, cardiac arrhythmias)	• Administer calcium gluconate 50 to 100 mg/kg IV slowly with ECG monitoring.<br>• Telemetry.<br>• Recheck potassium, phosphorus, uric acid, calcium, and creatinine in 1 hour STAT.<br>• If calcium normalized 1 hour later, repeat potassium, phosphorus, uric acid, calcium, and creatinine 2 and 4 hours later, if no other evidence of tumor lysis.<br>• Calculate corrected calcium and check ionized calcium if albumin low.
**Hyperphosphatemia**	
Phosphorus ≥5.0 mg/Dl (1.615 mmol/L) with ≥0.5 mg/dL (0.16 mmol/L) increase	• Administer a phosphate binder (e.g., aluminum hydroxide, calcium carbonate, sevelamer hydroxide, or lanthanum carbonate).<br>• Nephrology (or other acute dialysis service) notification (dialysis required for phosphorus ≥ 10 mg/dL).<br>• Recheck potassium, phosphorus, uric acid, calcium, and creatinine in 1 hour STAT.<br>• If phosphorus < 5.0 mg/dL 1 hour later, repeat potassium, phosphorus, uric acid, calcium, and creatinine 2 and 4 hours later, if no other evidence of tumor lysis.

Creatinine	
Increase ≥25% from baseline	• Start or increase rate of IV fluids.<br>• Recheck potassium, phosphorus, uric acid, calcium, and creatinine in 1 to 2 hours STAT.

Section 2: Ongoing Dosing of CA-4948 (beyond 24 hours of first dose)

Management of electrolyte changes from last value at intervals >24 hours after the first dose (e.g., 48 or 72 hours) are as below. *NOTE: If the patient is hospitalized, no additional CA-4948 doses should be administered until resolution.*

- For potassium, admit patient for any increase ≥ 1.0 mmol/L (1.0 mEq/L), or any level >ULN.
- Refer to the management guidelines for electrolyte changes observed within the first 24 hours after the first dose (see prior table).
- If a smaller potassium increase is observed that does not meet the criteria for admission above, recheck potassium, inorganic phosphate, uric acid, calcium, and creatinine in 24 hours and confirm no evidence of tumor lysis prior to further CA-4948 dosing.
- For uric acid, calcium, inorganic phosphate, and creatinine, refer to the management guidelines for electrolyte changes observed within the first 24 hours after the first dose (see prior table).

18.6.4 HOWARD DEFINITIONS OF LABORATORY AND CLINICAL TUMOR LYSIS SYNDROME

Metabolic Abnormality	Criteria for Classification of Laboratory TLS	Criteria for Classification of Clinical TLS
Hyperuricemia	Uric acid > 8.0 mg/dl (475.8 µmol/liter) in adults or above the upper limit of the normal range for age in children	
Hyperphosphatemia	Phosphorus > 4.5 mg/dl (1.5 mmol/liter) in adults or > 6.5 mg/dl (2.1 mmol/liter) in children	
Hyperkalemia	Potassium > 6.0 mmol/liter	Cardiac dysrhythmia or sudden death probably or definitely caused by hyperkalemia

Hypocalcemia	Corrected calcium < 7.0 mg/dl (1.75 mmol/liter) or ionized calcium < 1.12 (0.3 mmol/liter)†	Cardiac dysrhythmia, sudden death, seizure, neuromuscular irritability (tetany, paresthesias, muscle twitching, carpopedal spasm, Trousseau's sign, Chvostek's sign, laryngospasm, or bronchospasm), hypotension, or heart failure probably or definitely caused by hypocalcemia
Acute kidney injury††		Increase in the serum creatinine level of 0.3 mg/dl (26.5 µmol/liter) (or a single value > 1.5×ULN of the age-appropriate normal range if no baseline creatinine measurement is available) or the presence of oliguria, defined as an average urine output < 0.5 ml/kg/hr for 6 hrs

† The corrected calcium level in milligrams per deciliter = measured calcium level in milligrams per deciliter + 0.8 × (4-albumin in grams per deciliter).

†† Acute kidney injury is defined as an increase in the creatinine level of at least 0.3 mg per deciliter (26.5 µmol per liter) or a period of oliguria lasting 6 hours or more. By definition, if acute kidney injury is present, the patient has clinical TLS.

NOTE: In laboratory TLS, two or more metabolic abnormalities must be present during the same 24-hour period within 3 days before the start of therapy or up to 7 afterward. Clinical TLS requires the presence of laboratory tumor lysis syndrome plus an increased creatinine level, seizures, cardiac dysrhythmia, or death.

18.6.5 REVISED INTERNATIONAL PROGNOSTIC SCORING SYSTEM (IPSS-R)

IPSS-R Prognostic Score Values

**Prognostic Variable**	**0**	**0.5**	**1**	**1.5**	**2**	**3**	**4**
Cytogenetics	Very good	-	Good	-	Intermediate	Poor	Very poor
Bone marrow blast, %	≤2	-	>2% - <5%	-	5%-10%	>10%	-
Hemoglobin	≥10	-	8 - <10	<8	-	-	-
Platelets	≥100	50 - <100	<50	-	-	-	-
Absolute neutrophil count	≥0.8	<0.8	-	-	-	-	-

- indicates not applicable.

Reference: Greenberg PL, Tuechler H, Schanz J, et al. Revised international prognostic scoring system for myelodysplastic syndromes. Blood. 2012;120(12):2454-65. Website for accessing the IPSS-R calculator tool: *https://www.mds-foundation.org/calculator/index.php*. A mobile app for the calculator tool is also available.

IPSS-R Cytogenetic Risk Groups

Cytogenetic Prognostic Subgroups	Cytogenetic Abnormalities
Very good	-Y, del(11q)
Good	Normal, del(5q), del(12p), del(20q), double including del(5q)
Intermediate	del(7q), +8, +19, i(17q), any other single or double independent clones
Poor	-7, inv(3)/t(3q)/del(3q), double including -7/del(7q), complex: 3 abnormalities
Very poor	Complex: >3 abnormalities

Reference: Greenberg PL, Tuechler H, Schanz J, et al. Revised international prognostic scoring system for myelodysplastic syndromes. Blood. 2012;120(12):2454-65.

IPSS-R Prognostic Risk Categories and Clinical Outcomes

IPSS-R Risk Category (% IPSS-R pop.)	Overall Score	Median Survival (y) in the Absence of Therapy	25% AML Progression (y) in the Absence of Therapy
Very low (19)	≤1.5	8.8	Not reached
Low (38)	>1.5-3	5.3	10.8
Intermediate[a] (20)	>3-4.5	3.0	3.2
High (13)	>4.5-6	1.6	1.4
Very high (10)	>6	0.8	0.7

a Malcovati L, Della Porta MG, Strupp C, et al. Impact of the degree of anemia on the outcome of patients with myelodysplastic syndrome and its integration into the WHO classification-based Prognostic Scoring System (WPSS). Haematologica. 2011 Oct;96(10):1433-40.

Reference: Greenberg PL, Tuechler H, Schanz J, et al. Revised international prognostic scoring system for myelodysplastic syndromes. Blood. 2012;120(12):2454-65.

18.6.6 DOSE ADJUSTMENT FOR HEMATOLOGIC TOXICITIES DURING MDS TREATMENT

<u>The following general dose modification rules, based on ANC or platelets, are applied:</u>

- Subjects that experience a drop in the ANC (grad ≥4) or platelets (grad ≥3) during a cycle will hold the dose for the remaining cycle
- If dose was held, the start of next cycle is based on ANC or platelet recovery

<u>The following specific dose modification rules, based on ANC or platelets, are applied:</u>

<u>Platelet counts</u>
Subjects experiencing worsening of Thrombocytopenia to Grade 3 under IP treatment

**If baseline is at least 100,000/mcL**	
**When Platelets**	**Recommended Course**
Fall below 50,000/mcL	Interrupt treatment
Return to at least 100,000/mcL	Resume next cycle with last dose level
	If the subject continues to experience thrombocytopenia Grade 3 during 2 consecutive cycles, the treatment may be reduced by one dose level

Pre-existing Thrombocytopenia

**If baseline is below 100,000/mcL**
If the subject continues to experience thrombocytopenia Grade 3 or higher during 2 consecutive cycles, the treatment schedule may be reduced by one dose level<br>If dose was held, the start of next cycle is based on platelet recovery

ANC counts
Subjects experiencing worsening of Neutropenia to Grade 4 under IP treatment

**When Neutrophils**	**Recommended Course**
Fall below 500/mcL	Interrupt treatment, start antibiotic and antifungal prophylaxis per institutional guidelines
Return to at least 1000/mcl	Resume next cycle with last dose level
	If the subject continues to experience neutropenia Grade 4 during 2 consecutive cycles, the treatment may be reduced by one dose level

Pre-existing Neutropenia Grade 4

**If baseline is below 500/mcL**
If the subject continues to experience neutropenia Grade 4 during 2 consecutive cycles, the treatment may be reduced by one dose level<br>If dose was held, the start of next cycle is based on ANC recovery

18.6.7 EORTC QLQ-C30

EORTC QLQ-C30 (version 3)

We are interested in some things about you and your health. Please answer all of the questions yourself by circling the number that best applies to you. There are no "right" or "wrong" answers. The information that you provide will remain strictly confidential.

Please fill in your initials: |_|_|_|_|
Your birthdate (Day, Month, Year): |_|_|_|_|_|_|_|_|
Today's date (Day, Month, Year): 31 |_|_|_|_|_|_|_|_|

	Not at All	A Little	Quite a Bit	Very Much
1. Do you have any trouble doing strenuous activities, like carrying a heavy shopping bag or a suitcase?	1	2	3	4
2. Do you have any trouble taking a <u>long</u> walk?	1	2	3	4
3. Do you have any trouble taking a <u>short</u> walk outside of the house?	1	2	3	4
4. Do you need to stay in bed or a chair during the day?	1	2	3	4
5. Do you need help with eating, dressing, washing yourself or using the toilet?	1	2	3	4

**During the past week:**	Not at All	A Little	Quite a Bit	Very Much
6. Were you limited in doing either your work or other daily activities?	1	2	3	4
7. Were you limited in pursuing your hobbies or other leisure time activities?	1	2	3	4
8. Were you short of breath?	1	2	3	4
9. Have you had pain?	1	2	3	4
10. Did you need to rest?	1	2	3	4
11. Have you had trouble sleeping?	1	2	3	4
12. Have you felt weak?	1	2	3	4
13. Have you lacked appetite?	1	2	3	4
14. Have you felt nauseated?	1	2	3	4
15. Have you vomited?	1	2	3	4
16. Have you been constipated?	1	2	3	4

<u>Please go on to the next page</u>

SPECIMEN

During the past week:	Not at All	A Little	Quite a Bit	Very Much
17. Have you had diarrhea?	1	2	3	4
18. Were you tired?	1	2	3	4
19. Did pain interfere with your daily activities?	1	2	3	4
20. Have you had difficulty in concentrating on things, like reading a newspaper or watching television?	1	2	3	4
21. Did you feel tense?	1	2	3	4
22. Did you worry?	1	2	3	4
23. Did you feel irritable?	1	2	3	4
24. Did you feel depressed?	1	2	3	4
25. Have you had difficulty remembering things?	1	2	3	4
26. Has your physical condition or medical treatment interfered with your <u>family</u> life?	1	2	3	4
27. Has your physical condition or medical treatment interfered with your <u>social</u> activities?	1	2	3	4
28. Has your physical condition or medical treatment caused you financial difficulties?	1	2	3	4

For the following questions please circle the number between 1 and 7 that best applies to you

29. How would you rate your overall <u>health</u> during the past week?

1	2	3	4	5	6	7
Very poor						Excellent

30. How would you rate your overall <u>quality of life</u> during the past week?

1	2	3	4	5	6	7
Very poor						Excellent

SPECIMEN

18.6.8 EORTC QLQ-FA12

EORTC QLQ-FA12

Patients sometimes report that they have the following symptoms or problems. Please indicate the extent to which you have experienced these symptoms or problems during the past week. Please answer by circling the number that best applies to you.

**During the past week:**	**Not at all**	**A little**	**Quite a bit**	**Very much**
1. Have you lacked energy?	1	2	3	4
2. Have you felt exhausted?	1	2	3	4
3. Have you felt slowed down?	1	2	3	4
4. Did you feel sleepy during the day?	1	2	3	4
5. Did you have trouble getting things started?	1	2	3	4
6. Did you feel discouraged?	1	2	3	4
7. Did you feel helpless?	1	2	3	4
8. Did you feel frustrated?	1	2	3	4
9. Did you have trouble thinking clearly?	1	2	3	4
10. Did you feel confused?	1	2	3	4
11. Did tiredness interfere with your daily activities?	1	2	3	4
12. Did you feel that your tiredness is (was) not understood by the people who are close to you ?	1	2	3	4

SPECIMEN

19 REFERENCES

[1] U. Platzbecker, Treatment of MDS, Blood 133 (2019) 1096–1107. https://doi.org/10.1182/blood-2018-10-844696.

[2] A.D. Ricart, Drug-induced liver injury in Oncology, Ann. Oncol. 28 (2017) 2013–2020. https://doi.org/10.1093/annonc/mdx158.

[3] G.W. Rhyasen, D.T. Starczynowski, IRAK signalling in cancer, Br. J. Cancer 112 (2015) 232–237. https://doi.org/10.1038/bjc.2014.513.

[4] P.N. Kelly, D.L. Romero, Y. Yang, A.L. Shaffer, D. Chaudhary, S. Robinson, W. Miao, L. Rui, W.F. Westlin, R. Kapeller, L.M. Staudt, Selective interleukin-1 receptor-associated kinase 4 inhibitors for the treatment of autoimmune disorders and lymphoid malignancy, J. Exp. Med. 212 (2015) 2189–2201. https://doi.org/10.1084/jem.20151074.

[5] G.S. Choudhary, T.D. Bhagat, M.E.S. Samson, S. Gordon, D. von Ahrens, K. Pradhan, A. Shastri, A. Pellagatti, J. Boutlwood, R.N. Booher, U. Steidl, A. Verma, Abstract 127: Efficacy of novel IRAK4 inhibitor CA4948 in AML and MDS, in: Experimental and Molecular Therapeutics, American Association for Cancer Research, 07012017, p. 127.

[6] N.E. Genung, K.M. Guckian, Small Molecule Inhibition of Interleukin-1 Receptor-Associated Kinase 4 (IRAK4), Prog. Med. Chem. 56 (2017) 117–163. https://doi.org/10.1016/bs.pmch.2016.11.004.

[7] C.R. Cogle, Incidence and Burden of the Myelodysplastic Syndromes, Curr. Hematol. Malig. Rep. 10 (2015) 272–281. https://doi.org/10.1007/s11899-015-0269-y.

[8] U. Germing, C. Strupp, A. Kündgen, D. Bowen, C. Aul, R. Haas, N. Gattermann, No increase in age-specific incidence of myelodysplastic syndromes, Haematologica 89 (2004) 905–910.

[9] X. Ma, M. Does, A. Raza, S.T. Mayne, Myelodysplastic syndromes: incidence and survival in the United States, Cancer 109 (2007) 1536–1542. https://doi.org/10.1002/cncr.22570.

[10] P. Greenberg, Hematology: Basic Principles and Practice (2000).

[11] D.T. Starczynowski, A. Karsan, Deregulation of innate immune signaling in myelodysplastic syndromes is associated with deletion of chromosome arm 5q, Cell Cycle 9 (2010) 855–856. https://doi.org/10.4161/cc.9.5.11156.

[12] L.P. Gondek, R. Tiu, C.L. O'Keefe, M.A. Sekeres, K.S. Theil, J.P. Maciejewski, Chromosomal lesions and uniparental disomy detected by SNP arrays in MDS, MDS/MPD, and MDS-derived AML, Blood 111 (2008) 1534–1542. https://doi.org/10.1182/blood-2007-05-092304.

[13] D.T. Starczynowski, A. Karsan, Innate immune signaling in the myelodysplastic syndromes, Hematol. Oncol. Clin. North Am. 24 (2010) 343–359. https://doi.org/10.1016/j.hoc.2010.02.008.

[14] M.P. Boldin, K.D. Taganov, D.S. Rao, L. Yang, J.L. Zhao, M. Kalwani, Y. Garcia-Flores, M. Luong, A. Devrekanli, J. Xu, G. Sun, J. Tay, P.S. Linsley, D. Baltimore, miR-146a is a significant brake on autoimmunity, myeloproliferation, and cancer in mice, J. Exp. Med. 208 (2011) 1189–1201. https://doi.org/10.1084/jem.20101823.

[15] G.W. Rhyasen, L. Bolanos, J. Fang, A. Jerez, M. Wunderlich, C. Rigolino, L. Mathews, M. Ferrer, N. Southall, R. Guha, J. Keller, C. Thomas, L.J. Beverly, A. Cortelezzi, E.N. Oliva, M. Cuzzola, J.P. Maciejewski, J.C. Mulloy, D.T. Starczynowski, Targeting IRAK1 as a therapeutic approach for myelodysplastic syndrome, Cancer Cell 24 (2013) 90–104. https://doi.org/10.1016/j.ccr.2013.05.006.

[16] G.W. Rhyasen, L. Bolanos, D.T. Starczynowski, Differential IRAK signaling in hematologic malignancies, Exp. Hematol. 41 (2013) 1005–1007. https://doi.org/10.1016/j.exphem.2013.09.008.
[17] S. Dimicoli, Y. Wei, C. Bueso-Ramos, H. Yang, C. Dinardo, Y. Jia, H. Zheng, Z. Fang, M. Nguyen, S. Pierce, R. Chen, H. Wang, C. Wu, G. Garcia-Manero, Overexpression of the toll-like receptor (TLR) signaling adaptor MYD88, but lack of genetic mutation, in myelodysplastic syndromes, PLoS ONE 8 (2013) e71120. https://doi.org/10.1371/journal.pone.0071120.
[18] Y. Wei, S. Dimicoli, C. Bueso-Ramos, R. Chen, H. Yang, D. Neuberg, S. Pierce, Y. Jia, H. Zheng, H. Wang, X. Wang, M. Nguyen, S.A. Wang, B. Ebert, R. Bejar, R. Levine, O. Abdel-Wahab, M. Kleppe, I. Ganan-Gomez, H. Kantarjian, G. Garcia-Manero, Toll-like receptor alterations in myelodysplastic syndrome, Leukemia 27 (2013) 1832–1840. https://doi.org/10.1038/leu.2013.180.
[19] A. Carey, D.K. Edwards, C.A. Eide, L. Newell, E. Traer, B.C. Medeiros, D.A. Pollyea, M.W. Deininger, R.H. Collins, J.W. Tyner, B.J. Druker, G.C. Bagby, S.K. McWeeney, A. Agarwal, Identification of Interleukin-1 by Functional Screening as a Key Mediator of Cellular Expansion and Disease Progression in Acute Myeloid Leukemia, Cell Rep. 18 (2017) 3204–3218. https://doi.org/10.1016/j.celrep.2017.03.018.
[20] M.M. Hosseini, S.E. Kurtz, S. Abdelhamed, S. Mahmood, M.A. Davare, A. Kaempf, J. Elferich, J.E. McDermott, T. Liu, S.H. Payne, U. Shinde, K.D. Rodland, M. Mori, B.J. Druker, J.W. Singer, A. Agarwal, Inhibition of interleukin-1 receptor-associated kinase-1 is a therapeutic strategy for acute myeloid leukemia subtypes, Leukemia 32 (2018) 2374–2387. https://doi.org/10.1038/s41375-018-0112-2.
[21] D. Chaudhary, S. Robinson, D.L. Romero, Recent advances in the discovery of small molecule inhibitors of interleukin-1 receptor-associated kinase 4 (IRAK4) as a therapeutic target for inflammation and oncology disorders, J. Med. Chem. 58 (2015) 96–110. https://doi.org/10.1021/jm5016044.
[22] M.A. Smith, G.S. Choudhary, A. Pellagatti, K. Choi, L.C. Bolanos, T.D. Bhagat, S. Gordon-Mitchell, D. von Ahrens, K. Pradhan, V. Steeples, S. Kim, U. Steidl, M. Walter, I.D.C. Fraser, A. Kulkarni, N. Salomonis, K. Komurov, J. Boultwood, A. Verma, D.T. Starczynowski, U2AF1 mutations induce oncogenic IRAK4 isoforms and activate innate immune pathways in myeloid malignancies, Nat. Cell Biol. 21 (2019) 640–650. https://doi.org/10.1038/s41556-019-0314-5.
[23] B. Saez, M.J. Walter, T.A. Graubert, Splicing factor gene mutations in hematologic malignancies, Blood 129 (2017) 1260–1269. https://doi.org/10.1182/blood-2016-10-692400.
[24] M.E. Varney, K. Melgar, M. Niederkorn, M. Smith, L. Barreyro, D.T. Starczynowski, Deconstructing innate immune signaling in myelodysplastic syndromes, Exp. Hematol. 43 (2015) 587–598. https://doi.org/10.1016/j.exphem.2015.05.016.
[25] A. Jain, S. Kaczanowska, E. Davila, IL-1 Receptor-Associated Kinase Signaling and Its Role in Inflammation, Cancer Progression, and Therapy Resistance, Front. Immunol. 5 (2014) 553. https://doi.org/10.3389/fimmu.2014.00553.
[26] K. Newton, V.M. Dixit, Signaling in innate immunity and inflammation, Cold Spring Harb. Perspect. Biol. 4 (2012). https://doi.org/10.1101/cshperspect.a006049.
[27] A.A. Basiorka, K.L. McGraw, E.A. Eksioglu, X. Chen, J. Johnson, L. Zhang, Q. Zhang, B.A. Irvine, T. Cluzeau, D.A. Sallman, E. Padron, R. Komrokji, L. Sokol, R.C. Coll, A.A.B. Robertson, M.A. Cooper, J.L. Cleveland, L.A. O'Neill, S. Wei, A.F. List, The

NLRP3 inflammasome functions as a driver of the myelodysplastic syndrome phenotype, Blood 128 (2016) 2960–2975. https://doi.org/10.1182/blood-2016-07-730556.

[28] C.I. Maratheftis, E. Andreakos, H.M. Moutsopoulos, M. Voulgarelis, Toll-like receptor-4 is up-regulated in hematopoietic progenitor cells and contributes to increased apoptosis in myelodysplastic syndromes, Clin. Cancer Res. 13 (2007) 1154–1160. https://doi.org/10.1158/1078-0432.CCR-06-2108.

[29] R.K. Schneider, M. Schenone, M.V. Ferreira, R. Kramann, C.E. Joyce, C. Hartigan, F. Beier, T.H. Brümmendorf, U. Germing, U. Platzbecker, G. Büsche, R. Knüchel, M.C. Chen, C.S. Waters, E. Chen, L.P. Chu, C.D. Novina, R.C. Lindsley, S.A. Carr, B.L. Ebert, Rps14 haploinsufficiency causes a block in erythroid differentiation mediated by S100A8 and S100A9, Nat. Med. 22 (2016) 288–297. https://doi.org/10.1038/nm.4047.

[30] A. Tefferi, J.W. Vardiman, Myelodysplastic syndromes, N. Engl. J. Med. 361 (2009) 1872–1885. https://doi.org/10.1056/NEJMra0902908.

[31] D.A. Sallman, A. List, The central role of inflammatory signaling in the pathogenesis of myelodysplastic syndromes, Blood 133 (2019) 1039–1048. https://doi.org/10.1182/blood-2018-10-844654.

[32] C.E.M. de Mooij, M.G. Netea, W.J.F.M. van der Velden, N.M.A. Blijlevens, Targeting the interleukin-1 pathway in patients with hematological disorders, Blood 129 (2017) 3155–3164. https://doi.org/10.1182/blood-2016-12-754994.

[33] J.W. Singer, A. Fleischman, S. Al-Fayoumi, J.O. Mascarenhas, Q. Yu, A. Agarwal, Inhibition of interleukin-1 receptor-associated kinase 1 (IRAK1) as a therapeutic strategy, Oncotarget 9 (2018) 33416–33439. https://doi.org/10.18632/oncotarget.26058.

[34] L.S. Fridericia, The duration of systole in an electrocardiogram in normal humans and in patients with heart disease. 1920, Ann. Noninvasive Electrocardiol. 8 (2003) 343–351. https://doi.org/10.1046/j.1542-474x.2003.08413.x.

[35] Y.S. Jeelall, K. Horikawa, Oncogenic MYD88 mutation drives Toll pathway to lymphoma, Immunol. Cell Biol. 89 (2011) 659–660. https://doi.org/10.1038/icb.2011.31.

[36] D. Podsiadlo, S. Richardson, The timed "Up & Go": a test of basic functional mobility for frail elderly persons, J. Am. Geriatr. Soc. 39 (1991) 142–148. https://doi.org/10.1111/j.1532-5415.1991.tb01616.x.

[37] R. Starkman, S. Alibhai, R.A. Wells, M. Geddes, N. Zhu, M.M. Keating, B. Leber, L. Chodirker, M. Sabloff, G. Christou, H.A. Leitch, E. St-Hilaire, N. Finn, A. Shamy, K. Yee, J. Storring, T. Nevill, R. Delage, M. Elemary, V. Banerji, M. Lenis, A. Kirubananthaan, A. Mamedov, L. Zhang, K. Rockwood, R. Buckstein, An MDS-specific frailty index based on cumulative deficits adds independent prognostic information to clinical prognostic scoring, Leukemia 34 (2020) 1394–1406. https://doi.org/10.1038/s41375-019-0666-7.

[38] U. Platzbecker, P. Fenaux, L. Adès, A. Giagounidis, V. Santini, A.A. van de Loosdrecht, D. Bowen, T. de Witte, G. Garcia-Manero, E. Hellström-Lindberg, U. Germing, R. Stauder, L. Malcovati, M.A. Sekeres, D.P. Steensma, S. Gloaguen, Proposals for revised IWG 2018 hematological response criteria in patients with MDS included in clinical trials, Blood 133 (2019) 1020–1030. https://doi.org/10.1182/blood-2018-06-857102.

[39] CHMP/ICH, E 2 A Clinical Safety Data Management: Definitions and Standards for Expedited Reporting.

[40] Verordnung über die Anwendung der Guten Klinischen Praxis bei der Durchführung von klinischen Prüfungen mit Arzneimitteln zur Anwendung am Menschen (GCP-Verordnung - GCP-V) 2004.
[41] R. Simon, Optimal two-stage designs for phase II clinical trials, Controlled Clinical Trials 10 (1989) 1–10. https://doi.org/10.1016/0197-2456(89)90015-9.
[42] European Medicines Agency, E6 (R2) Step 5 Addendum – Good clinical practice.

Statistical Analysis Plan
(SAP)

for the trial

A Phase II, Single-Arm, Open-Label Study to Assess the Efficacy and Safety of Canakinumab for the Treatment of Anemia in Patients with IPSS-R Very Low, Low, or Intermediate Risk Myelodysplastic Syndromes or MDS/MPN (CANFIRE)

NCT05237713

EU CT Number: 2024-515886-33-00

Coordinating Investigator

Dr. Dominic Brauer

Klinik und Poliklinik für

Hämatologie, Zelltherapie, Hämostaseologie und Infektiologie

Universitätsklinikum Leipzig

Liebigstraße 22, D -04103 Leipzig

Responsible trial biometrician

Dr. Jens Przybilla and Dr. Dirk Hasenclever

Zentrum für Klinische Studien, Universität Leipzig

Härtelstrasse 16-18, D - 04107 Leipzig

jens.przybilla@zks.uni-leipzig.de

Date of the document: 2026-03-25

Version of the document: Final 1.0

We hereby approve the Statistical Analysis Plan in its final version:

Coordinating Investigator

Dr. Dominic Brauer

Date	Signature

Dr. Jens Przybilla

01.04.2026

X Jens Przybilla

Biometrician(1)

Signiert von: Jens Przybilla

Dr. Dirk Hasenclever

02.04.2026

X

Biometrician(2)

Signiert von: Dirk Hasenclever

Table of contents

1 Introduction 4

2 Changes in the planned analysis and sample size calculation 4

3 Endpoints and further variables 4

3.1 Primary Endpoint 4

3.2 Main Secondary Endpoints 5

3.3 Descriptive Secondary Endpoints 5

3.4 Safety Endpoints 5

3.5 Endpoints in add-on studies mentioned in the protocol 5

3.6 Further Variables 6

3.6.1 Baseline characteristics 6

3.6.2 Treatment delivery 7

4 General Analysis Definitions 7

4.1 Analysis Populations 7

4.2 Subgroups 7

4.3 Handling of center effects 7

5 Planned analysis 7

5.1 Demographic and other baseline parameter 7

5.2 Concomitant Diseases and Medication 7

5.3 Compliance with regard to the Study Intervention 8

5.4 Primary efficacy endpoints 8

5.5 Secondary efficacy endpoints 8

5.5.1 Treatment continuation after eight cycles due to perceived clinical benefit 8

5.5.2 Duration of HI-E response 8

Duration of HI-E response will be described only in patients with documented response. 8

5.5.3 Time to HI-E response 8

Time to HI-E response will be described only in patients with documented response. 8

5.5.4 Frequency of RBC transfusions in transfusion dependent patients 8

5.5.5 Neutrophil and platelet (HI-N and HI-P) responses according to IWG 2018 criteria 9

This analysis is restricted to the few patients that enter the study with low neutrophils and/or platelets. Individual cases are listed. 9

5.5.6 Quality of life (QoL) 9

5.5.7 Other laboratory parameters 9

5.6 Safety end points 9

6 Appendix 10

6.1 Abbreviations 10

1 Introduction

The purpose of this document is to provide a detailed elaboration of the statistical analysis described in the protocol, including detailed procedures for the confirmatory analysis of the primary and secondary endpoints and other variables.

The Statistical Analysis Plan (SAP) assumes familiarity with the Study Protocol (Version final 5.0 dated 28.03.2025), including Protocol Amendments. If in doubt the study protocol formulation takes precedence.

The SAP is based on the planned analysis specification as written in the study protocol Section 8 “Biometry”. SAP readers may consult the study protocol for more background information on the study, e.g., on study objectives, study design and population, trial intervention, definition of measurements and variables, planning of sample size, and randomization.

R 4.5 or higher, (Particular R-packages named below) will be used for statistical analyses.

2 Changes in the planned analysis and sample size calculation

The primary endpoint is an erythroid response (HI-E) according to International Working Group (IWG) 2018 criteria as implemented in the in-house R package *HbResponse*.

The protocol envisages that patients without a HI-E after eight cycles stop Canakinumab treatment. There are cases in whom the local investigator chooses to continue the treatment due to perceived clinical benefit despite lack of objective response.

We therefore add a further secondary endpoint, “treatment continuation after eight cycles due to perceived clinical benefit” in order to document these cases.

Quality of life endpoints were listed in the protocols synopsis as secondary endpoints and under 2.2 Secondary objectives, but not mentioned in the biometric section. The both questionnaires EORTC QLQC30 and QUALMS will be analysed as secondary endpoints.

Analysis of the secondary endpoint disease progression is restricted to bone marrow analysis. Additional parts of the IWG 2018 criteria (concerning granulocyte, platelet response, Hb reduction and transfusion dependence) don’t apply to the CANFIRE study population.

3 Endpoints and further variables

3.1 Primary Endpoint

The primary efficacy endpoint is HI-E according to IWG 2018 criteria.

Implementing IWG 2018 HI-E-response criteria in a clinical trial requires some modifications. Mostly, IWG 2018 rules are only specified further to make them technically operational, unambiguous, and to handle data problems expected in real world trial data.

The main deviation from IWG 2018 is the introduction of so-called sliding time windows over which Hb-measurements can be averaged or transfusion requirements assessed. These are used both to determine the baseline Hb-level (in NTD) and the baseline transfusion burden in (LTB/HTB) as well as determine Hb-response for Non-transfusion dependent (NTD) patients and transfusion independence in (LTB/HTB). This deviation makes the response definitions statistically robust and markedly increases the power to detect a true response (Hasenclever et al in preparation).

Only a documented response counts as success. In determining the response rate, patients who do not complete the treatment (without documented early response) or patients not assessable for response after treatment count as failures.

3.2 Main Secondary Endpoints

- Additional endpoint: Treatment continuation after eight cycles due to perceived clinical benefit
- HI-E response duration
- Time to HI-E
- Disease progression according to IWG 2018 criteria (restricted to bone marrow analysis)

3.3 Descriptive Secondary Endpoints

- Hemoglobin changes from baseline
- Red blood cell transfusion (RBC) independence ≥ 8 and ≥ 12 weeks
- Frequency of RBC transfusions in transfusion dependent patients

These endpoints will be mainly described by time course plots.

- Neutrophil (HI-N) response (pretreatment < 1.0 x 10^9/L)
    - At least 100% increase and an absolute increase of >0.5 x 10^9/L.
    - Increments of neutrophils for patients with a pretreatment ANC of >1.0 x 10^9/L are to be reported.

- Platelet (HI-P) response (pretreatment < 100 x 10^9/L)
    - Absolute increase of 30 x 10^9/L for patients starting with >20 x 10^9/L PLTs or
    - Increase from <20 x 10^9/L to >20 x 10^9/L and by at least 100%.
    - In addition:
    - Evolution of bleeding symptoms to be taken into account.
    - Increments of platelets for patients with a pretreatment PLT count of >100 x 10^9/L are to be reported.

Neutrophil and platelet counts will be described by time course plots.

- Quality of life (QoL) will be mainly described by time course plots.

3.4 Safety Endpoints

- Safety and toxicity profile of Canakinumab

3.5 Endpoints in add-on studies mentioned in the protocol

- Mutational pattern and burden of selected genes and their influence on response

This is not part of this SAP.

3.6 Further Variables

3.6.1 Baseline characteristics

Age is calculated from the **Birth Year**, recorded on patient medical history CRF.

Sex is recorded on patient medical history CRF.

Weight is recorded on physical examination CRF.

<u>MDS-/MDS/MPN-/CMML- groups at baseline</u>

- **MDS type** is recorded on central morphology CRF as (MDS-MLD / MDS-SLD / MDS-MLD-RS / MDR-SLD-RS / MDS-EB1 / MDS-EB2 / MDS del(5q) / MDS-U)
- **MDS/MPN type** is recorded on central morphology CRF as MDS/MPN-RS-T / MDS/MPNu, aCML
- **CMML type** is recorded on central morphology CRF as Type 0 / Type I / Type II

If a diagnosis at the central lab is not possible, the result from the local laboratory will be used.

<u>Classification regarding RBC transfusion status</u>

- Transfusion Burden is recorded at HB/TRANS before inclusion CRF as NTD / LTB / HTB and calculated with data base implementation of the *HbResponse* algorithm.

MDS, MDS/MPN prognostic scores are recorded at Disease risk score CRF as IPSS-R / CPSS

IPSS-R score is recorded at Disease risk score CRF

- **Bone marrow blasts** are recorded at Disease risk score CRF
- **Cytogenetic category** is recorded at Disease risk score CRF
- **Hemoglobin** is recorded at Disease risk score CRF
- **Thrombocytes** is recorded at Disease risk score CRF
- **Absolut neutrophile count** is recorded at Disease risk score CRF

CPSS is recorded at Disease risk score CRF

- **WHO type** is recorded at Disease risk score CRF as (CMML-1/CMML-2)
- **Leukocyte count** is recorded at Disease risk score CRF as (0= < 13 GPT/L; 1= ≥ 13 GPT/L)
- **Cytogenetic category** is recorded at Disease risk score CRF as low / intermediate / high
- **Transfusion needed** is recorded at Disease risk score CRF as no / yes

3.6.2 Treatment delivery

Number of cycles received. Each intervention cycle is documented with start time. The number of cycles for which a start time is recorded is calculated.

Cumulative dose received over all given cycles up to end of cycle 8.

Time from start of therapy to end of cycle 8.

4 General Analysis Definitions

4.1 Analysis Populations

The **Full Analysis Sets (FAS)** consists of all formally included patients that have taken any study medication.

A Per-Protocol analysis is not meaningful.

The **Safety Analysis Set (SAS)** consists of all patients who received the intervention.

4.2 Subgroups

No subgroup analysis is planned.

4.3 Handling of center effects

Center effects cannot be accounted for in the analysis because of small patient numbers.

5 Planned analysis

A flowchart according to the CONSORT statement will describe the disposition of all patients registered to the trial detailing screening failure before final inclusion, withdrawals, drop-outs and inclusion in the analyses sets defined above. Respective listing will be provided.

Standard methods of descriptive statistics will be used always indicating the number of valid and missing values. Summary statistic will be reasonably rounded to avoid pseudo-precision.

5.1 Demographic and other baseline parameter

Demographic and other baseline parameter will be described for the respective FAS.

5.2 Concomitant Diseases and Medication

Frequencies of concomitant diseases and medication will be described for the respective FAS.

5.3 Compliance with regard to the Study Intervention

Patients will be listed who deviated from the planned treatment. Percentage of received dose of intended dose will be described by and plotted as an empirical cumulative distribution function.

5.4 Primary efficacy endpoints

The response rate will be estimated with an exact two-sided 95% confidence interval.

The response rate will be described by initial transfusion dependency status.

The erythroid response (HI-E) is operationalized using the in-house R-package *HbResponse* version ≥ 0.2.6.

The formal time course of Hb and transfusions will be plotted for all patients.

5.5 Secondary efficacy endpoints

5.5.1 Treatment continuation after eight cycles due to perceived clinical benefit

The rate of treatment continuation after eight cycles due to perceived clinical benefit will be estimated with an exact two-sided 95% confidence interval. The rate will be described by initial transfusion dependency status.

Patients with treatment continuation after eight cycles due to perceived clinical benefit will be described in detail, in blood and lab parameter, transfusion rate, and quality of life assessments.

Investigators will be asked to provide a narrative to describe the perceived clinical benefit.

5.5.2 Duration of HI-E response

Duration of HI-E response will be described only in patients with documented response.

5.5.3 Time to HI-E response

Time to HI-E response will be described only in patients with documented response.

5.5.4 Frequency of RBC transfusions in transfusion dependent patients

We will describe the RBC transfusion frequency with individual cumulative transfusion plots covering both baseline and treatment periods. The slope of the curve shows the change in rate of transfusion per time interval.

We calculate transfusion rates dividing RBCs counts during the screening phase and during the treatment period by their respective period lengths in days. We illustrate the data by a boxplot and test the null hypothesis that there is no change using the paired Wilcoxon-test.

We also include initially NTD patients, as they too could become transfusion dependent.

5.5.5 Neutrophil and platelet (HI-N and HI-P) responses according to IWG 2018 criteria

This analysis is restricted to the few patients that enter the study with low neutrophils and/or platelets. Individual cases are listed.

5.5.6 Quality of life (QoL)

Quality of life is described and plotted as time course for each patient. For calculations we use the in-house R-package *qscorer* version >= 0.035.

Average time course will be analysed and plotted based on a linear mixed model for repeated measurements with variance heterogeneity.

5.5.7 Other laboratory parameters

Average time course will be analysed and plotted based on a linear mixed model for repeated measurements with variance heterogeneity.

5.6 Safety end points

The Safety profile of Canakinumab will be described. All documented adverse events and serious adverse events and their respective CTCAE grades will be tabulated by study-cohort with MedDRA-coding.

In addition, the maximum CTCAE grade per patient for specific AE types will be tabulated.

A list of deaths with causes of death will be provided.

6 Appendix

6.1 Abbreviations

AE	Adverse event
CRF	Case Report Form
CTCAE	Common Terminology Criteria for Adverse Events
FAS	Full analysis set
Hb	Hemoglobin
HI-E	Hematologic Improvement - Erythroid
HI-N	Hematologic Improvement – Neutrophils
HI-P	Hematologic Improvement – Platelets
HTB	High transfusion burden
LTB	Low transfusion burden
MedDRA	Medical Dictionary for Regulatory Activities
MeddRA-PT	MedDRA Preferred Term
MeddRA-SOC	MedDRA System Organ Class
NTD	Non transfusion dependent
PPS	Per protocol set
QoL	Quality of Life
RBC	Red blood cells
SAE	Serious adverse event
SAP	Statistical Analysis Plan
SAS	Safety analysis set

Statistical Analysis Plan
(SAP)

for the trial

A Phase II, Open-Label, Multicenter Study of Orally Administered CA-4948 for the Treatment of Anemia in Patients with Very Low, Low or Intermediate Risk Myelodysplastic Syndromes (MDS)
(LUCAS)

NCT05178342
EudraCT-No.: 2020-003986-20

Coordinating Investigator

Prof. Dr. Uwe Platzbecker

Klinik und Poliklinik für Hämatologie,

Zelltherapie und Hämostaseologie

Universitätsklinikum Leipzig

Liebigstraße 22, D -04103 Leipzig

Responsible trial biometrician

Dr. Dirk Hasenclever and Dr. Jens Przybilla

Zentrum für Klinische Studien, Universität Leipzig

Härtelstrasse 16-18, D - 04107 Leipzig

jens.przybilla@zks.uni-leipzig.de

Date of the document: 2025-04-02

Version of the document: Final 3.0

We hereby approve the Statistical Analysis Plan in its final version:

Coordinating Investigator	Date	Signature
Biometrician(1)	Date	Signature
Biometrician(2)	Date	Signature

Table of contents

1 Introduction 5

2 Changes in the planned analysis and sample size calculation 5

2.1 Changes in the planned analysis relative to the protocol 5

2.2 Changes in the planned analysis relative to the SAP version 1.0 5

3 Endpoints and further variables 6

3.1 Primary Endpoint 6

3.2 Main Secondary Endpoints 6

3.3 Descriptive Secondary Endpoints 6

3.4 Safety Endpoints..7

3.5 Endpoints in add-on studies mentioned in the protocol 7

3.6 Further Variables 7

3.6.1 Baseline characteristics.. 7

3.6.2 Treatment delivery 8

4 General Analysis Definitions 8

4.1 Analysis Populations..8

4.2 Subgroups 8

4.3 Handling of center effects 8

5 Planned analysis 9

5.1 Demographic and other baseline parameter 9

5.2 Concomitant Diseases and Medication 9

5.3 Compliance with regard to the Study Intervention 9

5.4 Primary efficacy endpoints 9

5.5 Secondary efficacy endpoints 9

5.5.1 Treatment continuation after four cycles due to perceived clinical benefit 9

5.5.2 Duration of HI-E response...10

Duration of HI-E response will be described only in patients with documented response. 10

5.5.3 Time to HI-E response 10

Time to HI-E response will be described only in patients with documented response. ..10

5.5.4 Frequency of RBC transfusions...10

5.5.5 Neutrophil and platelet (HI-N and HI-P) responses according to IWG 2018 criteria 10

This analysis is restricted to the few patients that enter the study with low neutrophils and/or platelets. Individual cases are listed. 10
5.5.6 Quality of life (QoL) 10
5.6 Safety end points 10
6 Appendix 11
6.1 Abbreviations 11

1 Introduction

The purpose of this document is to provide a detailed elaboration of the statistical analysis described in the protocol, including detailed procedures for the confirmatory analysis of the primary and secondary endpoints and other variables.

The Statistical Analysis Plan (SAP) assumes familiarity with the Study Protocol (Version final 4.0 dated 31.05.2022), including Protocol Amendments. If in doubt the study protocol formulation takes precedence.

The SAP is based on the planned analysis specification as written in the study protocol Section 9 “Biometry”. SAP readers may consult the study protocol for more background information on the study, e.g., on study objectives, study design and population, trial intervention, definition of measurements and variables, planning of sample size, and randomization.

R 4.4, (Particular R packages named below) will be used for statistical analyses.

2 Changes in the planned analysis and sample size calculation

2.1 Changes in the planned analysis relative to the protocol

The primary endpoint is an erythroid response (HI-E) according to IWG 2018 criteria as implemented in the in-house R package *HbResponse*.

The protocol envisages that patients without an erythroid response (HI-E) after four cycles stop CA-4948 treatment. There are cases in whom the local investigator and the patient choose to continue the treatment due to perceived clinical benefit despite lack of objective response.

We therefore add a further secondary endpoint, “treatment continuation after four cycles due to perceived clinical benefit” in order to document these protocol deviations.

Quality of life endpoints EORTC FA-12 and QLQC30 were listed in the protocols synopsis as secondary endpoints, but not mentioned in the biometric section. They will be analyzed as secondary endpoints.

Analysis of the secondary endpoint disease progression is restricted to bone marrow analysis. Additional parts of the IWG 2018 criteria (concerning granulocyte, platelet response, Hb reduction and transfusion dependence) are not applicable in LUCAS.

2.2 Changes in the planned analysis relative to the SAP version 1.0

The analysis of the first 21 patients in Cohort A triggered the stopping rule of the Simon design – (0/21 HI-E responses). Thus, the accrual to the Cohort A was stopped.

The results in Cohort A triggered a clinical reassessment of the risk-benefit consideration of the sub-study in Cohort B, although the sub-studies in Cohort A and B are formally independent as per study protocol. Eventually, also the accrual to the Cohort B was stopped.
We describe all patients that started treatment for HI-E response from both cohorts.

For all evaluable patients we provide individual synopses for each patient detailing baseline, medical history, treatment received, AEs experienced, and plots on the time course of Hb/transfusions, neutrophils, platelets and Quality of life scores.

In addition, we try to provide informative summary statistics concerning the following clinical questions:

- What is the toxicity profile of the drug?

- Description of AE types in general and by cohort
- Toxicity in laboratory parameters during treatment

- Does the rate of premature treatment discontinuations and toxicity differ by cohort?
- Is the evidence of an effect on the malignant clone in the bone marrow (as reported in other trials with the drug)?
- Do patients with perceived clinical benefit (treatment continues after 4 cycles) show any clinical or quality of live improvements that substantiate the clinical impression.

3 Endpoints and further variables

3.1 Primary Endpoint

The primary efficacy endpoint is erythroid response (HI-E) according to IWG 2018 criteria.

Implementing IWG 2018 Hb-response criteria in a clinical trial requires some modifications. Mostly, IWG 2018 rules are only specified further to make them technically operational, unambiguous, and to handle data problems expected in real world trial data.

The main deviation from IWG 2018 is the introduction of so-called sliding time windows over which Hb-measurements can be averaged not only for the determination of the baseline Hb-level, but also to determine Hb-response for NTD patients. This deviation makes the response definitions statistically robust and markedly increases the power to detect a true response (Hasenclever et al in preparation).

Only a documented response counts as success. In determining the response rate, patients who do not complete the treatment (without documented early response) or patients not assessable for response after treatment count as failures.

3.2 Main Secondary Endpoints

- Additional endpoint: Treatment continuation after four cycles due to perceived clinical benefit
- HI-E response duration
- Time to HI-E
- Disease progression according to IWG 2018 criteria (restricted to bone marrow analysis)

3.3 Descriptive Secondary Endpoints

- Hemoglobin changes from baseline
- Red blood cell transfusion (RBC) independence ≥ 8 and ≥ 12 weeks
- Frequency of RBC transfusions in transfusion dependent patients

These endpoints will be mainly described by time course plots.

- Neutrophil (HI-N) response ((pretreatment < 1.0 x 10^9/L)
  - At least 100% increase and an absolute increase of >0.5 x 10^9/L.
  - Increments of neutrophils for patients with a pretreatment ANC of >1.0 x 10^9/L are to be reported.

- Platelet (HI-P) response (pretreatment < 100 x 10^9/L)

- Absolute increase of 30 x 10^9/L for patients starting with >20 x 10^9/L PLTs or
- Increase from <20 x 10^9/L to >20 x 10^9/L and by at least 100%.
- In addition:
- Evolution of bleeding symptoms to be taken into account.
- Increments of platelets for patients with a pretreatment PLT count of >100 x 10^9/L are to be reported.

- Quality of life (QoL)

3.4 Safety Endpoints

- Safety and toxicity profile of CA-4948

3.5 Endpoints in add-on studies mentioned in the protocol

- Mutational pattern and burden of selected genes and their influence on response

This is not part of this SAP.

3.6 Further Variables

3.6.1 Baseline characteristics

Age is calculated from the **Birth Year**, recorded on patient medical history CRF.

Sex is recorded on patient medical history CRF.

Weight is recorded on physical examination CRF.

Height is recorded on physical examination CRF.

Preexisting conditions

MDS-/CMML- related medical history

- **Disease type** is recorded on medical history CRF as MDS or MDS/MPN
- **MDS type** is recorded on medical history CRF as (MDS-MLD / MDS-SLD / MDS-MLD-RS / MDR-SLD-RS / MDS-EB1 / MDS-EB2 / MDS del(5q) / MDS-U)
- **MDS/MPN type** is recorded on medical history CRF as MDS/MPN-RS-T / MDS/MPNu / CMML /aCML
- **CMML type** is recorded on medical history CRF as Type0 / Type I / Type II

Classification regarding RBC transfusion status

- **Transfusion Burden** is recorded at HB/TRANS before inclusion CRF as NTD / LTB / HTB and calculated with DB implementation of the *HbResponse* algorithm.

MDS, MDS/MPN prognostic scores are recorded at Disease risk score CRF as IPSS-R / CPSS

IPSS-R score is recorded at Disease risk score CRF

- **Bone marrow blasts** are recorded at Disease risk score CRF
- **Cytogenetic category** is recorded at Disease risk score CRF
- **Hemoglobin** is recorded at Disease risk score CRF
- **Thrombocytes** is recorded at Disease risk score CRF
- **Absolut neutrophile count** is recorded at Disease risk score CRF

CPSS is recorded at Disease risk score CRF

- **WHO type** is recorded at Disease risk score CRF as (CMML-1/CMML-2)
- **Leukocyte count** is recorded at Disease risk score CRF as (0= < 13 GPT/L; 1= ≥ 13 GPT/L)
- **Cytogenetic category** is recorded at Disease risk score CRF as low / intermediate / high
- **Transfusion needed** is recorded at Disease risk score CRF as no / yes

3.6.2 Treatment delivery

Number of cycles received. Each intervention cycle is documented with start and end time. The number of cycles for which a start time is recorded is calculated.

Cumulative dose received until Response assessment (end of cycle 4) in percent of the individually planned dose.

Time to treatment discontinuation.

4 General Analysis Definitions

4.1 Analysis Populations

The **Full Analysis Sets (FAS)** consists of all formally included patients in the respective sub-study that have taken any treatment medication.

A Per-Protocol analysis is not meaningful.

The **Safety Analysis Set (SAS)** consists of all patients who received the intervention in the respective sub-study. A safety analysis pooling sub-studies may also be performed.

4.2 Subgroups

Response rates will be described by the initial transfusion burden within each sub-study.

4.3 Handling of center effects

Center effects cannot be accounted for in the analysis because of small patient numbers.

5 Planned analysis

A flowchart according to the CONSORT statement will describe the disposition of all patients registered to the trial detailing screening failure before final inclusion, withdrawals, drop-outs and inclusion in the analyses sets defined above. Respective listing will be provided.

Standard methods of descriptive statistics will be used always indicating the number of valid and missing values. Summary statistic will be reasonably rounded to avoid pseudo-precision.

Standard methods of descriptive statistics will be used always indicating the number of valid and missing values. Summary statistics will be reasonably rounded to avoid pseudo-precision.

The two sub-studies are analysed separately.

5.1 Demographic and other baseline parameter

Demographic and other baseline parameter will be described for the respective FAS.

5.2 Concomitant Diseases and Medication

Frequencies of concomitant diseases and medication will be described for the respective FAS.

5.3 Compliance with regard to the Study Intervention

Patients will be listed who deviated from the planned treatment. Percentage of received dose of intended dose will be described by sub-study and plotted as an empirical cumulative distribution function.

5.4 Primary efficacy endpoints

The test of the null hypotheses – separately by cohort - is determined by the Simon design.

The response rate in each cohort will be estimated with an exact two-sided 95% confidence interval.

The response rate will be described by initial transfusion dependency status.

The erythroid response (HI-E) is operationalized using the in-house R-package *HbResponse*.

The formal time course of Hb and transfusions will be plotted for all patients.

5.5 Secondary efficacy endpoints

5.5.1 Treatment continuation after four cycles due to perceived clinical benefit

The rate of treatment continuation after four cycles due to perceived clinical benefit in each cohort will be estimated with an exact two-sided 95% confidence interval. The rate will be described by initial transfusion dependency status.

Patients with treatment continuation after four cycles due to perceived clinical benefit will be described in detail, in blood and lab parameter, transfusion rate, and quality of life assessments.

Investigators will be asked to provide a narrative to describe the perceived clinical benefit.

5.5.2 Duration of HI-E response

Duration of HI-E response will be described only in patients with documented response.

5.5.3 Time to HI-E response

Time to HI-E response will be described only in patients with documented response.

5.5.4 Frequency of RBC transfusions

We will describe the RBC transfusion frequency with individual cumulative transfusion plots covering both baseline and treatment periods. The slope of the curve shows the change in rate of transfusion per time interval.

We calculate transfusion rates dividing RBCs counts during the screening phase and during the treatment period by their respective period lengths in days. We illustrate the data by a boxplot and test the null hypothesis that there is no change using the paired Wilcoxon-test.

We include also initially NTD patients since some became transfusion dependent.

5.5.5 Neutrophil and platelet (HI-N and HI-P) responses according to IWG 2018 criteria

This analysis is restricted to the few patients that enter the study with low neutrophils and/or platelets. Individual cases are listed.

5.5.6 Quality of life (QoL)

Quality of life is described and analysed as time course. For calculation of the scores, we use the in-house R-package *qscorer* version >= 0.0.35.

5.6 Safety end points

The Safety profile of CA-4948 will be described. All documented adverse events and serious adverse events and their respective CTCAE grades will be tabulated by study-cohort with MedDRA-coding.

In addition, the maximum CTCAE toxicity grade per patient for given AE types will be tabulated.

A list of deaths with causes of death will be provided.

6 Appendix

6.1 Abbreviations

AE	Adverse event
CRF	Case Report Form
CTCAE	Common Terminology Criteria for Adverse Events
EC	Ethics Committee
EOS	End of study
EoT	End of therapy
FAS	Full analysis set
GCP	Good Clinical Practice
ICH	International Conference on Harmonisation
MedDRA	Medical Dictionary for Regulatory Activities
MeddRA-LLT	MedDRA Low Level Term
MeddRA-PT	MedDRA Preferred Term
MeddRA-SOC	MedDRA System Organ Class
QoL	Quality of life
PPS	Per protocol set
SAE	Serious adverse event
SAP	Statistical Analysis Plan
SAS	Safety analysis set

LUCAS study locations

Investigator	Study Centre(s)
Prof. Dr. Uwe Platzbecker	Universitätsklinikum Leipzig<br>Klinik und Poliklinik für Hämatologie, Zelltherapie, Hämostaseologie und Infektiologie<br>Liebigstraße 22, Haus 7, 04103 Leipzig
Prof. Dr. Katharina Götze	Klinikum rechts der Isar der TU München<br>III. Medizinische Klinik - Hämatologie und Onkologie<br>Ismaninger Straße 22<br>81675 München
Dr. Stefan Mahlmann	Friedrich-Ebert-Krankenhaus GmbH<br>Klinik für Hämatologie, Onkologie und Nephrologie<br>Friesenstrasse 44<br>24534 Neumünster
Dr. Kathrin Rieger	Charité Berlin - Campus Benjamin Franklin<br>Med. Klinik m. S. Hämatologie, Onkologie, Tumorimmunologie<br>Hindenburgdamm 30<br>12200 Berlin<br><br>Charité Berlin Campus Virchow Klinikum Med. Klinik m. S. Hämatologie, Onkologie und Tumorimmunologie

Investigator	Study Centre(s)
	Augustenburger Platz 1<br>13353 Berlin
PD Dr. Martin Schmidt-Hieber	Carl-Thiem-Klinikum Cottbus gGmbH<br>2. Med. Klinik<br>Thiemstraße 111<br>03048 Cottbus
PD Dr. Thomas Illmer	Gemeinschaftspraxis Dr. Jacobasch<br>Dresden<br>Arnoldstraße 18<br>01307 Dresden
Prof. Dr. Aristoteles Giagounidis	Marienhospital Düsseldorf<br>Klinik für Onkologie und Hämatologie,<br>Palliativmedizin<br>Rochusstraße 2<br>40479 Düsseldorf
Dr. Friederike Wortmann	Universitätsklinikum Schleswig-<br>Holstein<br>Klinik für Hämatologie und Onkologie<br>Campus Lübeck<br>Ratzeburger Allee 160<br>23538 Lübeck
Prof. Dr. Rudolf Weide	InVO-Institut für Versorgungsforschung in<br>der Onkologie

Investigator	Study Centre(s)
	Neversstraße 5<br>56068 Koblenz
Dr. Daniel Sasca	Universitätsmedizin der Johannes<br>Gutenberg-Universität Mainz<br>III. Medizinische Klinik und Poliklinik -<br>Hämatologie, Internistische Onkologie und<br>Pneumologie<br>Langenbeckstraße 1<br>55131 Mainz
PD Dr. Mohamad Jawhar	Universitätsklinikum Mannheim<br>III. Medizinische Klinik - Hämatologie und<br>Onkologie<br>Theodor-Kutzer-Ufer 1-3<br>68167 Mannheim
Dr. Martina Haibach	ONCOSEARCH<br>Institut für Klin. Studien GbR<br>Nägselbachstr. 49 c<br>91052 Erlangen
Dr. Ursula Vehling-Kaiser	VK & K Studien GbR<br>Studienzentrum<br>Achdorfer Weg 5<br>84036 Landshut

Investigator	Study Centre(s)
Dr. Mohammad Amen Wattad	Klinikum Hochsauerland GmbH<br>Klinik f. Hämatologie, Onkologie,<br>Palliativmedizin, Stammzelltransplantation<br>Schederweg 12<br>59872 Meschede
Dr. Julia Glück-Wolf	Rems-Murr-Kliniken gGmbH<br>Hämatologie, Onkologie und Palliativmedizin<br>Am Jakobsweg 1<br>71364 Winnenden

CANFIRE study locations

Investigator	Study Centre(s)
Active (date: 07/2026)	
Dr. Dominic Brauer	Universitätsklinikum Leipzig<br>Klinik und Poliklinik für Hämatologie, Zelltherapie, Hämostaseologie und Infektiologie<br>Liebigstraße 22, Haus 7, 04103 Leipzig
Dr. Philipp Kiewe	Medizinisches Versorgungszentrum<br>"Onkologischer Schwerpunkt am Oskar-Helene-Heim"<br>Clayallee 225 a<br>14195 Berlin
closed	
Prof. Dr. Katharina Götze	Klinikum rechts der Isar der TU München<br>III. Medizinische Klinik - Hämatologie und Onkologie<br>Ismaninger Straße 22<br>81675 München
PD Dr. Alexander Höllein	Rotkreuzklinikum München<br>III. Medizinische Abteilung<br>Nymphenburger Str. 163<br>80634 München
Dr. Katja Sockel	Universitätsklinikum Carl Gustav Carus an der Technischen Universität Dresden

Investigator	Study Centre(s)
	Medizinischen Klinik und Poliklinik I / Hämatologie<br>Fetscherstraße 74<br>01307 Dresden
Dr. Karin Meyer	Universitätsklinikum Bonn<br>Med. Klinik III / ZIM, Hämatologie/Onkologie<br>Venusberg - Campus 1<br>53127 Bonn
