# Supplementary material for: Canakinumab or emavusertib for anemia in lower-risk MDS: results of the CANFIRE and LUCAS phase 2, open-label, multicenter trials
Source: Commun Med (Lond). 2026 Sep 16;6:486. doi: 10.1038/s43856-026-01885-z (PMC13583006; doi:10.1038/s43856-026-01885-z)
Supplement: Supplementary file 2 [file 43856_2026_1885_MOESM2_ESM.docx]

Description of additional supplementary file

File name: Supplementary Data 1

Description: The source data underlying Figure 3 can be found in Supplementary Data 1

File name: Supplementary Data 2

Description: The source data underlying Figure 4 can be found in Supplementary Data 2

File name: Supplementary Data 3

Description: The source data underlying Figure 5 can be found in Supplementary Data 3

File name: Supplementary Data 4

Description: The source data underlying Figure 6 can be found in Supplementary Data 4.
